# Examining the measurement equivalence of the Maslach Burnout Inventory across age, gender, and specialty groups in US physicians

**DOI:** 10.1186/s41687-021-00312-2

**Published:** 2021-06-05

**Authors:** Keri J. S. Brady, R. Christopher Sheldrick, Pengsheng Ni, Mickey T. Trockel, Tait D. Shanafelt, Susannah G. Rowe, Lewis E. Kazis

**Affiliations:** 1 Department of Health Law, Policy & Management, Boston University School of Public Health, Boston, MA 02118 USA; 2 Biostatistics & Epidemiology Data Analytic Center, Boston University School of Public Health, Boston, MA USA; 3 Department of Psychiatry and Behavioral Sciences, Stanford School of Medicine, Stanford, CA USA; 4 Stanford Medicine WellMD Center, Stanford School of Medicine, Stanford, CA USA; 5 Boston Medical Center, Boston, MA USA; 6 Department of Ophthalmology, Boston University School of Medicine, Boston, MA USA

**Keywords:** Physician burnout, Health outcome measurement, Person-centered outcome measurement, Differential item functioning, Differential test functioning, Measurement equivalence, Measurement invariance

## Abstract

**Background:**

Disparities in US physician burnout rates across age, gender, and specialty groups as measured by the Maslach Burnout Inventory-Human Services Survey for Medical Personnel (MBI) are well documented. We evaluated whether disparities in US physician burnout are explained by differences in the MBI’s functioning across physician age, gender, and specialty groups.

**Methods:**

We assessed the measurement equivalence of the MBI across age, gender, and specialty groups in multi-group item response theory- (IRT-) based differential item functioning (DIF) analyses using secondary, cross-sectional survey data from US physicians (*n* = 6577). We detected DIF using two IRT-based methods and assessed its impact by estimating the overall average difference in groups’ subscale scores attributable to DIF. We assessed DIF’s practical significance by comparing differences in individuals’ subscale scores and burnout prevalence estimates from models unadjusted and adjusted for DIF.

**Results:**

We detected statistically significant age-, gender-, and specialty- DIF in all but one MBI item. However, in all cases, average differences in expected subscale-level scores due to DIF were < 0.10 SD on each subscale. Differences in physicians’ individual-level subscale scores and burnout symptom prevalence estimates across DIF- adjusted and unadjusted IRT models were also small (in all cases, mean absolute differences in individual subscale scores were < 0.04 z-score units; prevalence estimates differed by < 0.70%).

**Conclusions:**

Age-, gender-, and specialty-related disparities in US physician burnout are not explained by differences in the MBI’s functioning across these demographic groups. Our findings support the use of the MBI as a valid tool to assess age-, gender-, and specialty-related disparities in US physician burnout.

**Supplementary Information:**

The online version contains supplementary material available at 10.1186/s41687-021-00312-2.

Disparities in US physician burnout rates across age, gender, and specialty groups as measured by the Maslach Burnout Inventory-Human Services Survey for Medical Personnel (MBI-HSS) are well documented [1–6]. Physicians who are younger, female, and practicing in front-line specialties (e.g., emergency medicine, family medicine, and general internal medicine) have generally reported higher rates of burnout compared to their older, male colleagues practicing in non-front-line specialties [1]. In response, the National Academy of Medicine has recommended that healthcare organizations monitor and intervene on demographic disparities within their institutions [4]. However, it is unclear whether the observed disparities are explained by differences in the MBI-HSS’s functioning, or what is known as a lack of measurement equivalence, across demographic groups [4].

A measure is equivalent when it functions the same way across groups of respondents who might differ in gender, age, or other personal characteristics that may influence their responses to a self-reported measure. However, when a measure lacks equivalence across respondents who differ demographically, subscale score differences may actually reflect systematic differences in the way the demographic groups interpret items or in their willingness to endorse items, as opposed to true differences in the groups’ latent (unobserved) burnout symptom levels [7]. For example, female physicians may have higher observed burnout scores than male physicians because they are more willing than male physicians to report their symptoms, despite both groups having the same latent burnout levels. Establishing the measurement equivalence of an instrument is a key aspect of construct validity; and, consequently, is required for the unbiased comparison of physician burnout across demographic groups [8, 9]. However, no studies, to our knowledge, have evaluated the demographic measurement equivalence of the MBI-HSS in US physicians [10].

The aim of this study was to examine whether demographic disparities in US physician burnout are explained by differences in the MBI-HSS’s functioning across physician age, gender, and specialty groups.

## Methods

### Design and sample

This study used secondary, cross-sectional survey data from a national study on the prevalence of physician burnout conducted by Shanafelt et al. [2]. Data were collected in 2014 from physicians of all specialties sampled via email from the American Medical Association Physician Master File. Further sampling design details are reported in Shanafelt et al. [2]. From this dataset, we excluded physicians who were not practicing in the US or were retired.

### Measures

The MBI-HSS is an outcome assessment of job burnout containing three subscales: emotional exhaustion (EE) (9 items), depersonalization (DP) (5 items), and personal accomplishment (PA) (8 items). All MBI-HSS items have a 7-point Likert-type, frequency response scale (0 = *never*, 1 = *a few times a year or less*, 2 = *once a month or less*, 3 = *a few times a month*, 4 = *once a week*, 5 = *a few times a week*, 6 = *every day*). Higher scores on each subscale indicate more of each construct. Burnout symptoms are indicated by high scores on the EE and DP subscales and low scores on the PA subscale. Demographic variables included age group (< 35, 35–44, 45–54, 55–64, and ≥ 65 years), gender (male and female), and specialty.

### Statistical analyses

We evaluated the demographic measurement equivalence of the MBI-HSS subscales in a series of multi-group item response theory- (IRT-) based differential item functioning (DIF) analyses (Additional file 1: Appendix 1). IRT represents a class of generalized linear mixed effect models for relating observed item responses to latent constructs. Within an IRT framework, a lack of measurement equivalence in an item is called differential item functioning (DIF). For a particular item under investigation, DIF occurred when the probability of endorsing one or more item response significantly differed across reference and focal groups (e.g., males versus females) for physicians with the same latent burnout symptom (EE, DP, or PA) level.

IRT-based DIF analyses require that all IRT model assumptions, such as essential unidimensionality, have been met prior to analysis. These assumptions were evaluated and met in a previous IRT calibration of the MBI-HSS in US physicians using the same dataset by Brady et al. [11]. In Brady et al. [11], each scale demonstrated essential unidimensionality in unidimensional or bifactor confirmatory factor analyses. In following Brady et al. [11], we summed items EE4 (“people real strain”) and EE8 (“people too much stress”) to form a single, combined scale (EE4EE8) to meet IRT model assumptions.

Our statistical analyses proceeded in two main steps: 1) DIF detection and 2) DIF impact assessment. Our analyses were informed by the scientific standards for instrument development and validation developed by the National Institutes of Health Patient-Reported Outcomes Measurement Information System (NIH PROMIS) [8].

#### DIF detection (item-level)

Following best practices [8, 12], we employed two IRT-based approaches to detecting DIF in each subscale item: log-likelihood ratio tests (LRTs) and Chalmers et al.’s (2018) signed differential response functioning (sDRF) statistic [13]. These approaches have shown to be robust detection methods in previous studies [12–14]. Both DIF detection approaches require the selection of anchor items that have little to no DIF, which are used to estimate the reference and focal groups’ latent burnout symptom levels in multi-group IRT model estimation [8, 15]. Specialty groups with < 200 respondents were excluded from the DIF specialty analysis to ensure adequate sample size for DIF detection [14].

In the first DIF detection approach, we estimated an unconstrained baseline multi-group IRT model where all item parameters (except anchor items) were estimated freely across reference and focal groups and, for each item, compared its fit using a LRT against a more restrictive model where the item parameters for the studied item were constrained to be equal across groups. In the second DIF detection approach, we detected DIF in each subscale item using the sDRF statistic at the item-level, computed from the unconstrained baseline multi-group IRT model [13]. The item-level sDRF statistic estimates the overall average difference (bias) in the reference and focal groups’ expected item scores (i.e., raw item scores) across the latent burnout symptom continuum due to DIF in an item, after matching physicians on their latent burnout symptoms levels [13]. Items showing a significant Benjamini-Hochberg adjusted LRT statistic (*p* < 0.05) or a Benjamini-Hochberg adjusted item-level sDRF statistic were flagged as displaying statistically significant DIF in one or more item parameters.

DIF magnitude, or the degree of DIF present in an item, was captured by size of the item-level sDRF statistic, which is in the same raw score metric as item scores [8, 13]. For example, a negative item-level sDRF statistic of − 1.0 for a particular item indicates that the focal group’s item scores will be, on average, one score point higher than the reference group’s item scores due to DIF; whereas, a positive item-level sDRF statistic of 1.0 for a particular item indicates that the focal group’s item scores will be, on average, one score point lower than the reference group’s item scores due to DIF. To aid in the interpretation of DIF magnitude, we converted absolute item-level sDRF estimates to SD units based on their respective item score distributions.

#### DIF impact assessment (subscale-level)

Although items may display statistically significant DIF, the effect of the DIF on subscale scores across reference and focal groups may be negligible [16]. Therefore, an essential part of assessing measurement equivalence is to evaluate the impact of the statistically significant DIF identified [8, 12]. DIF impact relates to the aggregate effect of DIF across all subscale items on group- and individual-level *subscale scores* [8]. To evaluate DIF impact, we evaluated the size of the sDRF statistics at the subscale-level for all statistically significant DIF identified [13]. The subscale-level sDRF statistic estimates the overall average difference (bias) in the reference and focal groups’ expected *subscale scores* (i.e., raw total scores) across the underlying burnout symptom continuum due to the aggregate effects of DIF across all subscale items, after matching physicians on their underlying burnout symptoms levels [13]. For example, a negative subscale-level sDRF statistic of − 1.0 indicates that the focal group will have total scores that are, on average, one raw score point higher than the reference group’s total scores due to the aggregate effects of DIF in the subscale; whereas, a positive item-level sDRF statistic of 1.0 indicates that the focal group will have total scores that are, on average, one raw score point lower than the reference group’s total scores due to the aggregate effects of DIF in the subscale. A significant subscale-level sDRF statistic (*p* < 0.05) indicated that the aggregate effects of DIF across all subscale items resulted in significant differences in the subscale’s functioning across reference and focal groups. To aid in the interpretation of the DIF impact, we converted absolute subscale-level sDRF estimates to SD units based on their respective total score distributions.

For aggregate DIF that resulted in a significant subscale-level sDRF statistic (*p* < 0.05), we assessed its practical impact by comparing differences in individuals’ IRT-estimated subscale scores and burnout symptom prevalence estimates produced from multi-group IRT models that were unadjusted and adjusted for DIF [8, 12].

All statistical analyses were conducted in R (v3.5.1) using the *mirt* package (v1.31.4) [17, 18]. This study was approved by the Boston University Medical Campus Institutional Review Board (H-37414).

## Results

The overall sample included 6577 multi-specialty US physicians (Table 1). The majority of the sample was male, ≥ 55 years of age, and a non-primary care physician. We used physicians who were ≥ 65 years, male, and practicing in general internal medicine (GIM) as the reference group in respective age, gender, and specialty DIF analyses. Physicians in dermatology, neurosurgery, otolaryngology, pathology, radiation oncology, and urology were excluded from the specialty DIF analysis due to inadequate sample sizes.

**Table 1:** Overall and group-level sample characteristics

Characteristic	Overall Sample, n (%) ^a^	Emotional exhaustion subscale (*n* = 6264), mean total score (SD)	Depersonalization subscale (*n* = 6403), mean total score (SD)	Personal accomplishment subscale (*n* = 6201), mean total score (SD)
Overall	6577 (100)	25.74 (13.45)	8.07 (6.55)	40.03 (6.67)
Age group				
< 35 years	319 (5.0)	26.60 (12.77)	10.28 (7.06)	38.83 (6.35)
35–44 years	1201 (18.8)	27.98 (12.34)	9.90 (6.67)	38.54 (6.65)
45–54 years	1379 (21.6)	28.58 (12.93)	9.07 (6.78)	39.60 (6.57)
55–64 years	2129 (33.4)	27.15 (12.93)	7.90 (6.36)	40.35 (6.55)
***≥*** **65 years**	**1349 (21.2)**	**17.97 (13.09)**	**5.23 (5.27)**	**41.86 (6.23)**
Missing	200 (3.1)	--	--	--
Gender				
**Male**	**4287 (67.2)**	**24.66 (13.55)**	**7.99 (6.60)**	**40.36 (6.64)**
Female	2090 (32.8)	27.79 (12.98)	8.28 (6.45)	39.49 (6.47)
Missing	200 (3.1)	--	--	--
Specialty				
Anesthesiology	227 (3.5)	25.14 (12.63)	7.75 (5.97)	38.75 (7.78)
Dermatology	160 (2.5)	26.18 (12.95)	7.12 (5.75)	42.06 (5.41)
Emergency Medicine	342 (5.2)	29.09 (12.89)	12.86 (7.65)	37.95 (7.38)
Family Medicine	516 (7.9)	28.73 (13.72)	9.20 (6.66)	40.66 (5.93)
**General Internal Medicine**	**444 (6.8)**	**28.06 (13.87)**	**8.63 (6.86)**	**39.89 (6.74)**
General Pediatrics	354 (5.4)	23.81 (13.41)	6.49 (5.61)	40.18 (6.59)
General Surgery	244 (3.7)	24.17 (12.90)	8.26 (6.40)	40.11 (5.97)
General surgery subspecialty	363 (5.6)	23.87 (13.27)	7.49 (5.92)	40.34 (6.46)
Internal Medicine-subspecialty	750 (11.5)	26.08 (12.93)	7.40 (6.18)	40.40 (6.15)
Neurology	235 (3.6)	27.90 (13.63)	8.67 (6.60)	39.93 (6.44)
Neurosurgery	55 (0.8)	27.08 (12.36)	8.17 (5.59)	38.59 (7.68)
Obstetrics and gynecology (OBGYN)	281 (4.3)	25.18 (13.59)	8.06 (6.07)	40.64 (6.20)
Ophthalmology	230 (3.5)	24.27 (13.88)	7.70 (6.81)	40.92 (6.29)
Orthopedic surgery	232 (3.6)	25.95 (13.23)	9.47 (6.95)	41.21 (6.17)
Other	223 (3.4)	22.54 (14.09)	7.14 (6.86)	40.03 (6.62)
Otolaryngology	160 (2.5)	25.58 (13.18)	7.92 (5.71)	41.04 (5.60)
Pathology	155 (2.4)	24.71 (14.21)	6.82 (6.34)	35.02 (9.19)
Pediatric subspecialty	309 (4.7)	24.39 (12.49)	6.40 (5.49)	39.91 (6.45)
Physical medicine and rehabilitation/Preventive medicine, occupational medicine, or environmental medicine	272 (4.2)	24.15 (13.86)	8.03 (6.89)	39.40 (7.42)
Psychiatry	545 (8.4)	23.46 (13.27)	7.52 (6.32)	40.90 (6.47)
Radiation Oncology	63 (1.0)	23.57 (12.69)	5.84 (5.02)	42.23 (5.32)
Radiology	246 (3.8)	27.92 (12.86)	7.31 (6.29)	37.93 (7.41)
Urology	115 (1.8)	29.08 (14.23)	9.72 (7.41)	39.79 (7.06)
Missing	56 (0.9)	--	--	--

^a^ Percentages may not add to 100 due to rounding; bolded group is reference group in DIF analyses. *“--” =* not applicable

### Detection of DIF in subscale items

We detected statistically significant DIF (via one or both detection methods) across age, gender, and specialty groups in all MBI-HSS items except EE5 (“burned out from work”) (Tables 2, 3 and 4). Statistically significant age DIF was detected in five EE items (EE1, EE3, EE6, EE7, EE9), three DP items (DP2-DP4), and seven PA items (PA1-PA7) (Tables 2, 3 and 4). Statistically significant gender DIF was detected in four EE items (EE1, EE2, EE6, EE7), one DP item (DP1), and three PA items (PA1, PA4, PA6). Statistically significant specialty DIF was detected in five EE items (EE2, EE6, EE7, EE4EE8, EE9), all DP items (DP1-DP5), and five PA items (PA1, PA3, PA4, PA7, PA8). See Additional file 1: Appendices 2–3 for additional DIF detection results.

**Table 2:** DIF detection and magnitude results by item and demographic variable – Emotional Exhaustion subscale

		***Item-Level sDRF Estimate (95% CI) [absolute item-level sDRF estimate expressed in item SD units]***^a^							
Demographic Variable (Reference group)	Focal group	EE1 (emotionally drained)	EE2 (used up)	EE3 (fatigued)	EE5 (burned out)	EE6 (frustrated)	EE7 (working too hard)	EE4-EE8 (people a strain and/or too much stress)	EE9 (end of rope)
**Gender** (Male)	Female	−0.01 (− 0.06, 0.04) [< 0.01]^¥^	− 0.01 (− 0.04, 0.07) [< 0.01]^¥^	*--*	*--*	0.13 (0.07, 0.19) [0.07]^§^	0.20 (0.13, 0.27) [0.10]^§^	*--*	*--*
**Age Category** (≥ 65 years)	< 35 years	0.04 (− 0.09, 0.16) [0.02]^¥^	*--*	− 0.31 (− 0.44, − 0.18) [0.16]^§^	*--*	0.31 (0.18, 0.45) [0.17]^§^	0.18 (0.03, 0.34) [0.09]^§^	*--*	*--*
	35–44 years	< 0.01 (− 0.08, 0.08) [< 0.01]^¥^	*--*	− 0.33 (− 0.42, − 0.23) [0.17]^§^	*--*	0.13 (0.03, 0.23) [0.07]^§^	0.03 (−0.08, 0.14) [0.02]^¥^	*--*	*--*
	45–54 years	*--*	*--*	−0.13 (− 0.22, − 0.04) [0.07]^§^	*--*	0.11 (0.01, 0.20) [0.06]^§^	−0.16 (− 0.27, − 0.06) [0.08]^§^	*--*	0.16 (0.06, 0.26) [0.09]^§^
	55–64 years	*--*	*--*	*--*	*--*	*--*	− 0.18 (− 0.28, 0.08) [0.09]^§^	*--*	*--*
**Specialty** (General Internal Medicine)	Anesthesiology	*--*	*--*	*--*	*--*	*--*	0.10 (− 0.10, 0.29) [0.05]^¥^	*--*	*--*
	Emergency medicine	*--*	*--*	*--*	*--*	*--*	0.01 (− 0.16, 0.18) [< 0.01]^¥^	− 0.43 (− 0.60, − 0.24) [0.13]^§^	*--*
	Family medicine	*--*	*--*	*--*	*--*	*--*	--	*--*	*--*
	General pediatrics	*--*	*--*	*--*	*--*	0.33 (0.16, 0.50) [0.18]^†^	0.26 (0.09, 0.45) [0.13]^†^	*--*	*--*
	General surgery	*--*	0.15 (0.01, 0.30) [0.08]^†^	*--*	*--*	*--*	*--*	*--*	−0.25 (− 0.43, − 0.05) [0.13]^†^
	General surgery subspecialty	*--*	*--*	*--*	*--*	*--*	*--*	*--*	*--*
	Internal medicine subspecialty	*--*	*--*	*--*	*--*	*--*	*--*	*--*	*--*
	Neurology	*--*	*--*	*--*	*--*	*--*	*--*	*--*	*--*
	Obstetrics and gynecology	*--*	*--*	*--*	*--*	*--*	*--*	*--*	*--*
	Ophthalmology	*--*	*--*	*--*	*--*	*--*	*--*	*--*	*--*
	Orthopedic surgery	*--*	*--*	*--*	*--*	*--*	*--*	*--*	*--*
	Pediatric subspecialty	*--*	*--*	*--*	*--*	0.21 (0.05, 0.38) [0.11]^§^	*--*	*--*	*--*
	Physical medicine and rehabilitation/preventive medicine/occupational medicine	*--*	*--*	*--*	*--*	0.15 (− 0.01, 0.30) [0.08]^¥^	*--*	*--*	*--*
	Psychiatry	*--*	*--*	*--*	*--*	0.12 (− 0.03, 0.27) [0.06]^¥^	*--*	*--*	*--*
	Radiology	*--*	*--*	*--*	*--*	*--*	0.08 (− 0.12, 0.30) [0.04]^¥^	*--*	*--*

^a^ “--” = no DIF detected.^§^ DIF significant (Benjamini-Hochberg adjusted *p* < 0.05) in both the Likelihood Ratio Test (LRT) and item-level signed Differential Response Functioning (sDRF) statistic detection method;^¥^ DIF significant (Benjamini-Hochberg adjusted *p* < 0.05) in the Likelihood Ratio Test (LRT) detection method only;^†^ DIF significant (Benjamini-Hochberg adjusted *p* < 0.05) in the item-level sDRF statistic detection method only. Mean (raw) item scores from the EE calibration sample (*n* = 6264) in Brady et al. [11] were: EE1 = 3.54 (1.75), EE2 = 3.83 (SD = 1.77), EE3 = 3.02 (SD = 1.93), EE5 = 2.84 (2.01), EE6 = 3.69 (SD = 1.85), EE7 = 3.66 (SD = 1.95), EE4EE8 = 3.48 (SD = 3.20), EE9 = 1.67 (SD = 1.88)

**Table 3:** DIF detection and magnitude results by item and demographic variable – Depersonalization subscale

		***Item-Level sDRF Estimate (95% CI) [absolute item-level sDRF estimate expressed in item SD units]*** ^a^				
Demographic Variable (Reference group)	Focal group	DP1 (treat patients as objects)	DP2 (more callous)	DP3 (job hardening me)	DP4 (don’t care)	DP5 (patients blame me)
**Gender** (Male)	Female	0.15 (0.08, 0.22) [0.13] ^§^	*--*	*--*	*--*	*--*
**Age Category** (≥ 65 years)	< 35 years	*--*	*--*	0.02 (− 0.14, 0.16) [< 0.01] ^¥^	0.04 (− 0.11, 0.14) [0.03] ^¥^	*--*
	35–44 years	*--*	0.15 (0.00, 0.27) [0.08] ^¥^	0.03 (− 0.12, 0.17) [< 0.01] ^¥^	*--*	*--*
	45–54 years	*--*	*--*	− 0.20 (− 0.31, − 0.08) [0.10] ^§^	0.08 (− 0.01, 0.18) [0.06] ^¥^	*--*
	55–64 years	*--*	*--*	− 0.14 (− 0.24, 0.03) [0.07] ^§^	0.04 (− 0.04, 0.12) [0.03] ^¥^	*--*
**Specialty** (General Internal Medicine)	Anesthesiology	− 0.31 (− 0.52, − 0.09) [0.19] ^§^	*--*	*--*	0.40 (0.19, 0.56) [0.30] ^§^	*--*
	Emergency medicine	− 0.27 (− 0.48, − 0.07) [0.17] ^†^	*--*	0.38 (0.17, 0.59) [0.19] ^§^	*--*	--
	Family medicine	*--*	*--*	0.40 (0.21, 0.58) [0.20] ^§^	*--*	*--*
	General pediatrics	*--*	− 0.27 (− 0.46, − 0.06) [0.15] ^†^	*--*	*--*	*--*
	General surgery	*--*	*--*	*--*	*--*	*--*
	General surgery subspecialty	*--*	*--*	*--*	0.35 (0.20, 0.49) [0.26] ^†^	−0.28 (− 0.52, − 0.05) [0.15] ^†^
	Internal medicine subspecialty	*--*	*--*	*--*	*--*	*--*
	Neurology	*--*	*--*	0.41 (0.19, 0.63) [0.20] ^§^	*--*	*--*
	Obstetrics and gynecology	*--*	− 0.29 (− 0.49, − 0.08) [0.16] ^†^	*--*	*--*	*--*
	Ophthalmology	*--*	*--*	*--*	*--*	*--*
	Orthopedic surgery	*--*	*--*	*--*	0.23 (0.08, 0.42) [0.17] ^†^	*--*
	Pediatric subspecialty	*--*	*--*	*--*	*--*	*--*
	Physical medicine and rehabilitation/preventive medicine/occupational medicine	*--*	*--*	*--*	*--*	*--*
	Psychiatry	*--*	*--*	0.42 (0.24, 0.61) [0.21] ^§^	*--*	− 0.33 (− 0.54, − 0.12) [0.18] ^†^
	Radiology	−0.31 (− 0.52, − 0.07) [0.19] ^§^	*--*	*--*	*--*	0.53 (0.29, 0.78) [0.28] ^§^

^a^ “--” = no DIF detected. ^§^ DIF significant (Benjamini-Hochberg adjusted *p* < 0.05) in both the Likelihood Ratio Test (LRT) and item-level signed Differential Response Functioning (sDRF) statistic detection method; ^¥^ DIF significant (Benjamini-Hochberg adjusted *p* < 0.05) in the Likelihood Ratio Test (LRT) detection method only; ^†^ DIF significant (Benjamini-Hochberg adjusted *p* < 0.05) in the item-level sDRF statistic detection method only. Mean (raw) item scores from the DP calibration sample (*n* = 6403) in Brady et al. [11] were: DP1 = 1.19 (SD = 1.59), DP2 = 1.79 (SD = 1.81), DP3 = 2.05 (SD = 2.05), DP4 = 0.76 (SD = 1.33), DP5 = 2.28 (SD = 1.87)

**Table 4:** DIF detection and magnitude results by item and demographic variable – Personal Accomplishment subscale

		***Item-Level sDRF Estimate (95% CI) [absolute item-level sDRF estimate expressed in item SD units]*** ^a^							
Demographic Variable (Reference group)	Focal group	PA1 (easily understand patients)	PA2 (deal effectively with patient problems)	PA3 (positively influencing others)	PA4 (feel energetic)	PA5 (can create relaxed atmosphere)	PA6 (exhilarated)	PA7 (accomplished many things)	PA8 (deal with problems calmly)
**Gender** (Male)	Female	−0.21 (− 0.26, − 0.16) [0.20] ^§^	*--*	*--*	0.13 (0.06, 0.20) [0.09] ^§^	*--*	− 0.10 (− 0.17, − 0.02) [0.06] ^†^	*--*	*--*
**Age Category** (≥ 65 years)	< 35 years	− 0.14 (− 0.24, − 0.02) [0.13] ^§^	− 0.11 (− 0.18, − 0.03) [0.11] ^§^	0.05 (− 0.05, 0.16) [0.04] ^¥^	0.16 (0.02, 0.31) [0.11] ^§^	*--*	--	0.25 (0.14, 0.38) [0.19] ^†^	*--*
	35–44 years	− 0.21 (− 0.30, − 0.12) [0.20] ^§^	−0.20 (− 0.29, − 0.13) [0.19] ^§^	*--*	0.20 (0.08, 0.31) [0.13] ^§^	*--*	0.05 (− 0.07, 0.16) [0.03] ^¥^	0.23 (0.14, 0.32) [0.17] ^§^	*--*
	45–54 years	−0.27 (− 0.35, − 0.18) [0.24] ^§^	−0.26 (− 0.34, − 0.18) [0.25] ^§^	*--*	0.15 (0.03, 0.26) [0.10] ^§^	−0.12 (− 0.21, − 0.02) [0.09] ^§^	--	*--*	*--*
	55–64 years	−0.22 (− 0.31, − 0.13) [0.20] ^§^	−0.17 (− 0.25, − 0.10) [0.17] ^†^	*--*	0.29 (0.20, 0.39) [0.19] ^§^	--	0.19 (0.09, 0.28) [0.12] ^§^	*--*	*--*
**Specialty** (General Internal Medicine)	Anesthesiology								
	Emergency medicine	*--*	*--*	*--*	− 0.30 (− 0.46, − 0.14) [0.20] ^§^	*--*	*--*	--	*--*
	Family medicine	*--*	*--*	*--*	*--*	*--*	*--*	*--*	*--*
	General pediatrics	*--*	*--*	*--*	*--*	*--*	*--*	*--*	*--*
	General surgery	*--*	*--*	*--*	*--*	*--*	*--*	*--*	*--*
	General surgery subspecialty	0.23 (0.10, 0.36) [0.21] ^†^	*--*	*--*	*--*	*--*	*--*	*--*	0.30 (0.14, 0.46) [0.26] ^§^
	Internal medicine subspecialty	--	*--*	*--*	*--*	*--*	*--*	*--*	*--*
	Neurology	--	--	− 0.19 (− 0.33, − 0.05) [0.19] ^†^	*--*	*--*	*--*	*--*	*--*
	Obstetrics and gynecology	*--*	*--*	*--*	*--*	*--*	*--*	--	*--*
	Ophthalmology	*--*	*--*	*--*	*--*	*--*	*--*	− 0.33 (− 0.47, − 0.17) [0.25] ^§^	0.25 (0.09, 0.41) [0.21] ^§^
	Orthopedic surgery	*--*	*--*	*--*	*--*	*--*	*--*	*--*	*--*
	Pediatric subspecialty	*--*	*--*	*--*	*--*	*--*	*--*	*--*	*--*
	Physical medicine and rehabilitation/preventive medicine/occupational medicine	*--*	*--*	*--*	*--*	*--*	*--*	*--*	*--*
	Psychiatry	*--*	*--*	*--*	*--*	*--*	*--*	*--*	− 0.24 (− 0.33, − 0.14) [0.21] ^§^
	Radiology	*--*	*--*	*--*	*--*	*--*	*--*	*--*	--

^a^ “--” = no DIF detected. ^§^ DIF significant (Benjamini-Hochberg adjusted *p* < 0.05) in both the Likelihood Ratio Test (LRT) and item-level signed Differential Response Functioning (sDRF) statistic detection method; ^¥^ DIF significant (Benjamini-Hochberg adjusted *p* < 0.05) in the Likelihood Ratio Test (LRT) detection method only; ^†^ DIF significant (Benjamini-Hochberg adjusted *p* < 0.05) in the item-level sDRF statistic detection method only. Mean (raw) item scores from the PA calibration sample (*n* = 6201) in Brady et al. [11] were: PA1 = 5.41 (SD = 1.10), PA2 = 5.51 (SD = 1.03), PA3 = 5.22 (SD = 1.20), PA4 = 4.30 (SD = 1.50), PA5 = 5.26 (SD = 1.29), PA6 = 4.24 (SD = 1.62), PA7 = 4.91 (SD = 1.33), PA8 = 5.17 (SD = 1.17)

Most EE items that had statistically significant age, gender, or specialty DIF were of a small magnitude, representing less than 0.10 SD of a given item’s score (Table 2). The DP and PA subscales had several items demonstrating larger age, gender, or specialty DIF, representing greater than 0.20 SD of a given item’s score (Tables 3 and 4). Within the EE, DP, and PA subscales, the largest DIF was observed in item: EE6 across GIM and general pediatrics specialty groups; DP4 across GIM and anesthesiology specialty groups; and PA8 across GIM and general surgery subspecialty groups, respectively. On average, general pediatricians, anesthesiologists, and general surgery subspecialists had respective item scores on EE6, DP4, and PA8 that were 0.33 points (0.18 SD), 0.40 points (0.30 SD), and 0.30 (0.26 SD) lower than general internists due to DIF.

### Impact of DIF on subscale scores

A subset of the statistically significant DIF produced significant overall average differences in expected subscale scores across demographic groups (Table 5). However, in all cases, the overall average differences in total scores due to DIF amounted to less than 0.10 SD on each subscale (Table 5, also see Additional file 1: Appendix 4). Age DIF impacted both the PA and EE subscales, but had no significant impact on the DP subscale (Table 5). Compared to physicians ≥ 65 years, physicians 45–54 and 55–64 years had respective total scores on the PA and EE subscales that were, on average, 0.49 score points (0.07 SD) and 0.18 score points (0.01 SD) higher due to the aggregate effects of age DIF. Gender DIF impacted the EE subscale, but had no significant effect on the DP and PA subscales (Table 5). Compared to male physicians, female physicians had EE total scores that were, on average, 0.34 score points (0.03 SD) lower due to gender DIF. This was primarily caused by gender DIF in items EE6 and EE7, where female physicians were systematically less likely to endorse feeling she is “frustrated with work” and “working too hard” than male physicians, respectively.

**Table 5:** Summary of statistically significant subscale-level signed differential response functioning (sDRF) estimates - by demographic variable and MBI-HSS subscale

Grouping Variable (Reference Group)	Focal group	Subscale-level sDRF estimate (95% CI) [absolute subscale-level sDRF estimate expressed in subscale SD units] ^a^		
		Emotional Exhaustion (EE) subscale	Depersonalization (DP) subscale	Personal Accomplishment (PA) subscale
**Sex** (Male)	Female	0.34 (0.19, 0.49) [0.03]	--	--
**Age Category **(≥ 65 years)	< 35 years	--	--	--
	35–44 years	--	--	--
	45–54 years	--	--	− 0.49 (− 0.70, − 0.29) [0.07]
	55–64 years	− 0.18 (− 0.27, − 0.08) [0.01]	--	--
**Specialty** (General Internal Medicine)	Anesthesiology	--	--	− 0.60 (− 1.10, − 0.05) [0.09]
	Emergency medicine	− 0.42 (− 0.69, − 0.16) [0.03]	--	− 0.30 (− 0.44, − 0.14) [0.04]
	Family medicine	--	0.40 (0.22, 0.58) [0.06]	--
	General pediatrics	0.63 (0.26, 1.03) [0.05]	−0.35 (− 0.66, − 0.03) [0.05]	--
	General surgery	--	--	--
	General surgery subspecialty	--	--	0.53 (0.34, 0.74) [0.08]
	Internal medicine subspecialty	--	--	--
	Neurology	− 0.46 (− 0.91, 0.01) [0.03]	0.41 (0.18, 0.61) [0.06]	−0.55 (− 1.05, − 0.11) [0.08]
	Obstetrics and gynecology	--	− 0.38 (− 0.72, 0.06) [0.06]	--
	Ophthalmology	--	--	--
	Orthopedic surgery	--	--	--
	Pediatric subspecialty	0.21 (0.05, 0.37) [0.02]	--	--
	Physical medicine and rehabilitation/ preventive medicine/ occupational medicine	--	--	--
	Psychiatry	--	--	− 0.24 (− 0.33, − 0.14) [0.04]
	Radiology	--	--	--

^a^ “--” indicates no significant subscale-level sDRF detected

Specialty DIF impacted all three MBI-HSS subscales (Table 5). On the EE subscale: emergency medicine physicians and neurologists had total scores that were, on average, 0.42 score points (0.03 SD) and 0.46 score points (0.03 SD) higher than general internists due to specialty DIF, respectively; and general pediatricians and pediatric subspecialists had total scores that were, on average, 0.63 score points (0.05 SD) and 0.21 score points (0.02 SD) lower than general internists due to specialty DIF, respectively. On the DP subscale: family physicians and neurologists had total scores that were, on average, 0.40 score points (0.06 SD) and 0.41 score points (0.06 SD) lower than general internists due to specialty DIF, respectively; and general pediatricians and OBGYN physicians had total scores that were, on average, 0.35 score points (0.05 SD) and 0.38 score points (0.06 SD) higher than general internists due to specialty DIF, respectively. On the PA subscale: anesthesiologists, emergency medicine, neurologists, and psychiatrists had total scores that were, on average, 0.60 score points (0.09 SD), 0.30 score points (0.04 SD), 0.55 score points (0.08 SD), and 0.24 score points (0.04 SD) higher than general internists due to specialty DIF, respectively; and general surgery subspecialists had 0.53 score points (0.08 SD) lower than general internists due to specialty DIF.

Among the subscales with significant subscale-level sDRF, differences produced from DIF- unadjusted and adjusted models in physicians’ individual-level subscale scores and in symptom prevalence estimates were also very small (Table 6). In all cases, mean absolute differences in individual subscale scores and correlations between individual physicians’ subscale scores produced between DIF- unadjusted and adjusted models were < 0.04 z-score units and > 0.99, respectively. The absolute differences between physicians’ scores produced from EE, DP, and low PA prevalence estimates all differed by 0.00 to < 0.70%.

**Table 6:** Impact of aggregate DIF within the EE, DP, and PA subscales on individual physicians’ subscale scores and burnout symptom prevalence estimates ^a^

Scale- DIF Grouping variable	Reference (R) group; Focal (F) group (Sample n)	Mean absolute difference (SD) between individual physicians’ latent burnout symptom score from DIF unadjusted and adjusted multi-group IRT models	Correlation between individual physicians’ latent burnout symptom score from DIF unadjusted and adjusted multi-group IRT models	Prevalence of EE/DP/low PA from DIF-unadjusted Multi-group IRT Model, % (n)	Prevalence of EE/DP/low PA from DIF-adjusted Multi-group IRT Model, % (n)	Absolute difference in EE/DP/low PA prevalence estimates between DIF- unadjusted and adjusted multi-group IRT model
EE – Sex	Male (R); Female (F) (*n* = 6083)	0.00 (0.01)	0.9999	46.7% (2842)	46.6% (2835)	0.1%
EE – Age Group	≥65 years (R); 55–64 years (F) (*n* = 3271)	0.00 (0.01)	0.9999	40.8% (1336)	40.7% (1330)	0.1%
EE – Specialty	GIM (R); Emergency medicine (F) (*n* = 744)	0.01 (0.02)	0.9997	49.2% (366)	49.1% (365)	0.1%
	GIM (R); General pediatrics (F) (*n* = 762)	0.01 (0.02)	0.9998	47.6% (363)	47.9% (365)	0.3%
	GIM (R); Neurology (F)	0.01 (0.03)	0.9995	49.3% (318)	49.1% (317)	0.2%
	GIM (R); Pediatric subspecialty (F) (*n* = 717)	0.01 (0.01)	0.9999	43.5% (312)	43.2% (310)	0.3%
DP – Specialty	GIM (R); Family medicine (F) (*n* = 946)	0.03 (0.04)	0.9991	39.5% (374)	40.0% (378)	0.5%
	GIM (R); General pediatrics (F) (*n* = 784)	0.05 (0.07)	0.9977	33.7% (264)	33.7% (264)	0.0%
	GIM (R); Neurology (F) (*n* = 672)	0.02 (0.05)	0.9986	37.8% (254)	38.1% (256)	0.7%
	GIM (R); Obstetrics and gynecology (F) (*n* = 708)	0.04 (0.05)	0.9984	37.4% (265)	37.7% (267)	0.3%
PA – Age Group	≥65 years (R); 45–54 years (F) (*n* = 2540)	0.02 (0.03)	0.9996	13.6% (346)	13.6% (346)	0.0%
PA – Specialty	GIM (R); Anesthesiology (F) *n* = 642)	0.03 (0.05)	0.9987	18.5% (119)	18.2% (117)	0.3%
	GIM (R); Emergency Medicine (*n* = 761)	0.01 (0.03)	0.9996	19.2% (146)	18.9% (144)	0.3%
	GIM (R); General surgery subspecialty (F) (*n* = 766)	0.03 (0.03)	0.9993	14.9% (114)	14.9% (114)	0.0%
	GIM (R); Neurology (F) (*n* = 653)	0.02 (0.06)	0.9981	14.1% (92)	14.4% (94)	0.3%
	GIM (R); Psychiatry (F) (*n* = 923)	0.02 (0.02)	0.9998	13.2% (123)	13.0% (121)	0.2%

^a^ Calculated for all analyses resulting in significant subscale-level signed DRF; DIF-unadjusted multi-group IRT models were those where item parameters for studied items were constrained to equality; DIF-adjusted multi-group IRT models were those where item parameters for studied items were freely estimated. Prevalence of EE, DP, and low PA was obtained by finding the theta score corresponding with the commonly used respective cut offs of ≥ 27, ≥ 10, and ≤ 33 on each scale based on EAP sum scoring produced from the DIF-unadjusted and DIF-adjusted multi-group IRT models

## Discussion

Studies have consistently demonstrated disparities in physician burnout by age, gender, and specialty on the MBI-HSS [4, 19, 20]. However, the extent to which disparities are explained by differences in the MBI-HSS’s functioning across demographic subgroups of US physicians has been unclear. In this study, we evaluated the measurement equivalence of the MBI-HSS subscales across age, gender, and specialty groups in a sample of US physicians. We found a lack of measurement equivalence across demographic groups in all *items* except EE5 (“feel burned out from work”), demonstrating that physicians’ age group, gender, or specialty biased nearly all item scores to some degree. However, in all cases, the overall average aggregate effects of DIF on biasing the total *subscale scores* were small (< 0.10 SD). Furthermore, DIF had very little practical impact on individual-level physicians’ scores and burnout symptom prevalence estimates. Overall, our findings demonstrate that age-, gender-, and specialty-related disparities in US physician burnout are not explained by differences in the MBI’s functioning across these demographic groups.

Our study has several important implications for federal agencies and healthcare organizations aiming to monitor demographic disparities in physician burnout using the MBI-HSS [20–22]. First, our findings support the use of the MBI-HSS as a valid tool to assess age-, gender-, and specialty-related disparities in US physician burnout. Second, our research underscores the importance of using the full MBI-HSS subscales to assess demographic disparities in burnout versus using individual items. At the subscale level, the effects of DIF often cancelled out. Subscale-level cancellation effects occur when DIF causes bias of similar magnitude but in opposing direction (e.g., one item upward biases total scores and another downward biases total scores of the same magnitude). Therefore, the subscale scores generally showed less bias due to DIF than item scores. If researchers are interested in using individual items or subsets of items, however, our analyses can be used to select the items with the least DIF. Furthermore, since the item-level and subscale-level sDRF statistics represent the degree of item- and subscale level bias in the same raw score metric as item scores and total scores, researchers can use our findings to 1) assess the impact that DIF may have on a particular analysis and 2) adjust cross-group comparisons of raw item and total scores for DIF.

This study has several main limitations. First, DIF analyses can be prone to Type I due to multiplicity or if the wrong anchor items are selected. We mitigated this by not only applying multiplicity adjustment but by also thoroughly evaluating whether statistically significant DIF impacted group-level subscale scores, individual-level subscale scores, and burnout symptom prevalence. Second, our analysis computed the overall average difference in item and total scores due to DIF across a range of latent burnout symptom scores. As these differences are *overall average differences* across the latent metric*,* the bias in reference and focal groups’ scores at a particular point on the latent metric may be larger or smaller than the overall average [13]. Third, there is a paucity of literature on what constitutes “small” item-level DIF. However, our methods of examining the impact of DIF on subscale scores and burnout symptom prevalence are reasonable solutions. Fourth, although early and late responder analyses by Shanafelt et al. [2] support the demographic representativeness of the sample, it is possible that the this sample is not entirely representative of the current US physician population. However, assuming that the items in this sample function the same as in the US physician population, the findings of this study would not be different. Finally, although the MBI-HSS subscales demonstrated measurement equivalence across age, gender, and specialty, they may lack equivalence across other groups that we did not evaluate (e.g., race/ethnicity groups). Future studies are needed to evaluate whether the MBI-HSS functions equivalently across other demographic groups.

## Conclusions

As the MBI-HSS is increasingly employed in research and practice to monitor disparities in US physician burnout, it is important to understand its performance across demographic groups. Our findings demonstrate that differences in the way the MBI-HSS subscales function across groups do not account for the observed disparities in US physician burnout across age, gender, and specialty groups. Our findings support the use of the MBI-HSS as a valid tool to assess disparities in burnout across age, gender, and specialty groups in US physicians. Further research is needed to understand how these measures function across other physician subgroups.

## Supplementary Information

**Additional file 1 **: **Appendix 1**: Additional detail on DIF analysis methods. **Appendix 2**: Anchor item identification results and detailed LRT and item-level sDRF statistic DIF detection results. **Appendix** 3: Plots of expected item and test score functions and multi-group IRT item parameter estimates. **Appendix 4**: Detailed DIF impact results – subscale-level signed DRF (sDRF) and unsigned DRF (uDRF) statistics.

## Data Availability

This study was a re-analysis of existing data obtained upon request from the author of the original study (10.1016/j.mayocp.2015.08.023).

## Abbreviations

DIF: Differential item functioning
DP: Depersonalization
EE: Emotional Exhaustion
IRT: Item response theory
LRT: Likelihood Ratio Test
MBI-HSS: Maslach Burnout Inventory-Human Services Survey for Medical Personnel
PA: Personal Accomplishment
NIH PROMIS: National Institutes of Health Patient-Reported Outcomes Measurement Information System
sDRF: Signed differential response functioning
US: United States
