# Supplementary material for: Examining the measurement equivalence of the Maslach Burnout Inventory across age, gender, and specialty groups in US physicians
Source: J Patient Rep Outcomes. 2021 Jun 5;5:43. doi: 10.1186/s41687-021-00312-2 (PMC8179856; doi:10.1186/s41687-021-00312-2)
Supplement: Supplementary file 1 — Additional file 1 : Appendix 1: Additional detail on DIF analysis methods. Appendix 2: Anchor item identification results and detailed LRT and item-level sDRF statistic DIF detection results. Appendix 3: Plots of expected item and test score functions and multi-group IRT item parameter estimates. Appendix 4: Detailed DIF impact results – subscale-level signed DRF (sDRF) and unsigned DRF (uDRF) statistics. [file 41687_2021_312_MOESM1_ESM.pdf]

Online Supplemental Appendices:
Brady et al. (2021). Examining the Measurement Equivalence of the Maslach Burnout Inventory Across Age, Gender, and Specialty Groups in US Physicians. *Journal of Patient Reported Outcomes.*

Supplemental Appendix 1: Additional detail on DIF analysis methods

Following PROMIS scientific standards for evaluating differential item functioning (DIF) [8], we employed two IRT-based approaches to evaluate DIF in each MBI-HSS subscale across age, sex, and specialty groups: the IRT Log-Likelihood Ratio Test (IRTLR) and Chalmers et al. (2018) Differential Response Functioning (DRF) statistics [13]. The IRTLR approach was used to select anchor items and detect DIF. The IRTLR approach requires testing DIF in two groups at a time, i.e., a reference and focal group [12]. The DRF statistics were also used to detect DIF and as a primary method for quantifying DIF magnitude and impact.

The IRTLR and DRF approaches are robust methods [13, 14]. Compared to other DIF detection methods, the IRTLR approach has demonstrated improved power to detect uniform and non-uniform DIF [13, 14, 22], improved power in small samples [13], and less susceptibility to type I errors caused by non-normality of groups' latent distributions [12, 22]. The DRF statistics also offer a powerful and flexible approach to detecting DIF and quantifying its magnitude and impact (i.e., the impact of DIF on scale scores) [13]. Both approaches also rely upon the IRT-estimated latent score for determining respondents' latent trait levels, which is more accurate than the use of an observed score as is used in several other DIF detection methods [22]. Finally, while the IRTLR approach offers the ability to evaluate DIF magnitude and impact visually through the evaluation of differences in each group's expected item and test scores, the DRF approach creates a statistic to quantify these differences while taking into account the sampling variability of expected item and test scores [13]. These differences are quantified in the metric of the scale, aiding in the interpretability of DIF magnitude and impact.

Anchor item selection

Both the IRTLR and DRF approaches require the selection of anchor items that are used to estimate each group's underlying trait levels and link both groups onto the same latent metric in multi-group IRT estimation [8, 15]. Anchor items are constrained to equality in the IRT multi-group estimation and, importantly, are assumed to have no or minimal DIF. The presence of DIF in anchor items ("contamination") may cause inaccurate estimates of individuals' standing on the latent construct, and, therefore, may lead to inflated Type I error rates in DIF detection [12, 13]. To identify a set of anchor items with little to no DIF, a purification process whereby items are iteratively tested for DIF and removed from the set of candidate anchor items is recommended [12]. We employed a purification process known as an iterative, backward all-other approach, which does not require prior knowledge regarding which items may have DIF [12, 16]. This approach compares the fit of a constrained baseline model to a less constrained, nested model in a likelihood ratio test (LRT). Specifically, we estimated a baseline multi-group IRT model where all item parameters were constrained to equality across groups and, for each item, compared the baseline model's fit against a less restrictive model where all items except the studied item was constrained to equality across groups. In both models, the reference group's latent mean and

variance was set to 0 and 1, respectively, and the focal group's latent mean and variance was estimated freely. Items showing a significant Benjamini-Hochberg adjusted LRT statistic ($p < 0.05$) were flagged as potentially displaying DIF in one or more item parameters across the reference and focal groups. If one or more items was flagged for DIF, the item parameters for flagged item(s) were allowed to vary across groups (in both the baseline and less restrictive models) and the items that showed invariance were re-tested for DIF. This process was repeated until no new DIF items were identified. This process was implemented using the "drop sequential" option in the R *mirt* package's DIF function. Items that did not show DIF (LRT p-value < 0.05) in the final iteration of this purification process were selected as anchor items. We aimed to select a minimum of three anchor items for all DIF tests, which has demonstrated adequate power to detect DIF using the IRT-based detection approach in prior studies [12]. In cases where fewer than three items were identified as DIF-free, we identified additional anchors by selecting items that produced the smallest Akaike Information Criteria (AIC) differences in nested model comparisons made in the final round of the iterative, backward all-other DIF testing. In cases where no DIF was detected in the iterative, drop sequential purification approach, an initial anchor item was selected, and we conducted a LRT using a forward DIF testing approach (comparing an unconstrained model to a model with each study item constrained to equality) with the single anchor. Items with the lowest AIC difference in the forward LRT were selected as anchors in the final DIF detection process.

Final DIF detection

We used both the IRTLR and DRF statistics to detect DIF. In the IRTLR approach, we compared nested models using the LRT to detect DIF in each item using a forward approach. In this approach, the fit of an unconstrained baseline model was compared to a partially constrained, nested model using a LRT. Specifically, we estimated a baseline multi-group IRT model where all item parameters (except anchor items) were estimated freely across groups and, for each item, compared its fit against a more restrictive model where the item parameters for the studied item were constrained to equality across groups. In both the unconstrained and partially constrained models, the reference group's latent mean and variance were set to 0 and 1, respectively, the focal group's latent mean and variance were estimated freely, and anchor items were constrained to equality across both groups. In accordance with best practices [16, 22], items that were identified as DIF-free in this final DIF detection stage were not added the anchor set for subsequent DIF testing. The forward approach was selected for final DIF detection based on research suggesting the backward approach may result in inflated Type I error rates [23].

In the DRF approach, we detected DIF in each subscale item using the signed DRF statistic at the item-level (i.e., item-level signed DRF [sDRF] statistic), which was computed from the unconstrained baseline multi-group IRT model [13]. The item-level sDRF statistic estimates the overall average difference (bias) in the reference and focal groups' expected item scores across the underlying (latent) burnout symptom continuum due to DIF in an item [13].

Items showing a significant Benjamini-Hochberg adjusted LRT statistic ($p < 0.05$) *or* a Benjamini-Hochberg adjusted item-level sDRF statistic were flagged as displaying statistically significant DIF in one or more item parameters.

Following DIF detection, we plotted expected item scores across both groups for each item to evaluate whether statistically significant LRTs were due to the presence of uniform or non-uniform DIF, which were visually identified based on the presence of non-crossing and crossing expected item score functions, respectively. We also identified the direction(s) in which the DIF was occurring, i.e., at what latent trait levels does the focal group have a higher or lower probability of item endorsement than the reference group?

Evaluation of DIF Magnitude and Impact

Although items may display statistically significant DIF, the effect of the DIF on item and scale scores across reference and focal groups may be negligible [17]. This is particularly likely for large samples where the statistical power to detect even very small effects is high. Therefore, an essential part of assessing measurement equivalence is to evaluate the magnitude and impact of the statistically significant DIF identified [8, 12]. DIF magnitude relates to the degree of DIF present in *an item*; whereas, DIF impact relates to the aggregate effect of DIF across all subscale items on group- and individual-level *subscale scores* [8].

To evaluate DIF magnitude and impact, we first visually inspected plots of expected item and test score functions across groups, respectively. At any particular latent trait level, the distance between expected item/test score functions provided an estimate of the differences between each group's expected item/test scores due to DIF. These differences are in the metric of the item or scale. At the scale level, differences in the expected test scores across groups for respondents with the same latent trait levels is called differential test function (DTF) [17, 24]. Although visual inspection of differences in the expected item/test score functions is helpful, quantifying DIF magnitude and impact depends on several factors, including: whether compensatory or non-compensatory measures of DIF/DTF are used, where on the latent trait distribution the DIF/DTF is observed, and the sampling variability of the expected item/test score functions. We used the signed Differential Response Functioning (sDRF) effect size statistic at the item- and subscale-level developed by Chalmers et al. (2018), which take these factors into account when quantifying DIF magnitude and impact, respectively. The sDRF statistic is a compensatory differential response functioning (i.e., DIF or DTF) statistic in that it accounts for the cancellation effects that occur in non-uniform DIF or DTF. That is, when DIF or DTF occurs in opposing directions across the latent trait, the overall average differences in expected item or test scores may cancel each other out resulting in no to little differences in expected item/test scores on average across the latent trait range. Whereas, non-compensatory differential response functioning statistics take the overall average absolute differences in expected item/test score functions across groups across the latent trait range, not allowing for cancellation effects. Non-compensatory DRF is important in a computer-adaptive setting where it is not known in advance what items will be administered to each respondent. However, for a fixed-form instrument such as the MBI-HSS subscales, all subscale items are administered to each respondent. As such, we considered the compensatory effects of the items in our DIF impact estimates. The sDRF statistic applied at the item- and subscale-level estimates the overall average difference in respective expected item and test scores across the latent trait. A *positive* item-level sDRF statistic indicates that due to DIF, 1) the focal group will be on average *less* likely to endorse the item than the reference group across the latent trait and 2) will have *lower expected item scores* (raw item scores) on average compared to the reference group across the latent trait; a *positive* subscale-

level sDRF statistic indicates that due to aggregate DIF effects in the scale, the focal group will have *lower expected test scores* (raw total scores) on average than the reference group across the latent trait. A *negative* item-level sDRF statistic indicates that due to DIF, 1) the focal group will be on average *more* likely to endorse the item than the reference group across the latent trait and 2) will have *higher expected item scores* on average compared to the reference group across the latent trait; a *negative* subscale-level sDRF statistic indicates that due to aggregate DIF effects in the scale, the focal group will have *higher expected test scores* on average than the reference group across the latent trait.

Supplemental Appendix 2: Anchor Item Identification Results and Detailed LRT and Item-Level sDRF statistic DIF Detection Results

Table 2.1 Anchor item identification results: EE Scale

DIF Grouping Variable	*Reference group (n); focal group (n)*	*Item*	**Likelihood-ratio test for anchor item identification using a backward sequential approach [a]**			
			AIC difference	*X^2 (df)*	*p-value*	*B-H adjusted p-value*
**Sex**	Reference: male (n = 4078)<br><br>Focal: female (n = 2005)	EE1	-10.56	22.56 (6)	**0.0010**	**0.0014**
		EE2	-11.06	23.06 (6)	**0.0008**	**0.0014**
		EE3	-9.26	21.26 (6)	**0.0017**	**0.0020**
		EE5	--	--	--	--
		EE6	-20.03	32.03 (6)	**<0.0000**	**<0.0000**
		EE7	-39.71	51.71 (6)	**<0.0000**	**<0.0000**
		EE4EE8	--	--	--	--
		EE9	-7.06	19.06 (6)	**0.0041**	**0.0041**
**Age Category**	Reference: ≥65 years (n = 1258)<br><br>Focal: <35 years (n = 313)	EE1	-12.66	24.66 (6)	**0.0004**	**0.0007**
		EE2	-5.25	17.25 (6)	**0.0084**	**0.0118**
		EE3	-46.37	58.37 (6)	**0.0000**	**0.0000**
		EE5	--	--	--	--
		EE6	-25.00	37.00 (6)	**0.0000**	**0.0000**
		EE7	-29.33	41.33 (6)	**0.0000**	**0.0000**
		EE4EE8	2.00	22.00 (12)	**0.0376**	**0.0376**
		EE9	-2.49	14.49 (6)	**0.0246**	**0.0287**
	Reference: ≥65 years (n = 1258)<br><br>Focal: 35-44 years (n = 1167)	EE1	-6.47	18.47 (6)	**0.0051**	**0.0064**
		EE2	--	--	--	--
		EE3	-75.27	87.27 (6)	**<0.0000**	**<0.0000**
		EE5	--	--	--	--
		EE6	-46.27	58.27 (6)	**<0.0000**	**<0.0000**
		EE7	-27.65	39.65 (6)	**<0.0000**	**<0.0000**
		EE4EE8	--	--	--	--
		EE9	-4.87	16.87 (6)	**0.0098**	**0.0098**
	Reference: ≥65 years (n = 1258)<br><br>Focal: 45-54 years (n = 1328)	EE1	--	--	--	--
		EE2	--	--	--	--
		EE3	-11.54	23.54 (6)	**0.0006**	**0.0008**
		EE5	--	--	--	--
		EE6	-12.98	24.98 (6)	**0.0003**	**0.0007**
		EE7	-39.41	51.41 (6)	**<0.0000**	**<0.0000**
		EE4EE8	--	--	--	--
		EE9	-9.99	21.99 (6)	**0.0012**	**0.0012**
	Reference: ≥65 years (n = 1258)<br><br>Focal: 55-64 years (n = 2013)	EE1	--	--	--	--
		EE2	--	--	--	--
		EE3	--	--	--	--
		EE5	--	--	--	--
		EE6	--	--	--	--
		EE7	-14.25	26.25 (6)	**0.0002**	**0.0002**
		EE4EE8	--	--	--	--
		EE9	--	--	--	--

**Specialty**	GIM (n = 424) (R); Anesthesiology (n = 219) (F)	EE1	--	--	--	--
		EE2	--	--	--	--
		EE3	--	--	--	--
		EE5	--	--	--	--
		EE6	--	--	--	--
		EE7	--	--	--	--
		EE4EE8	--	--	--	--
		EE9	--	--	--	--
	GIM (R) (n = 424); Emergency medicine (F) (n = 320)	EE1	--	--	--	--
		EE2	--	--	--	--
		EE3	--	--	--	--
		EE5	--	--	--	--
		EE6	--	--	--	--
		EE7	-33.50	45.50 (6)	**<0.0000**	**<0.0000**
		EE4EE8	-8.51	20.51 (6)	**0.0022**	**0.0022**
		EE9	--	--	--	--
	GIM (R) (n = 424); Family medicine (F) (n = 494)	EE1	--	--	--	--
		EE2	--	--	--	--
		EE3	--	--	--	--
		EE5	--	--	--	--
		EE6	--	--	--	--
		EE7	--	--	--	--
		EE4EE8	--	--	--	--
		EE9	--	--	--	--
	GIM (R) (n = 424); General Pediatrics (n = 338) (F)	EE1	--	--	--	--
		EE2	--	--	--	--
		EE3	--	--	--	--
		EE5	--	--	--	--
		EE6	--	--	--	--
		EE7	--	--	--	--
		EE4EE8	--	--	--	--
		EE9	--	--	--	--
	GIM (R) (n = 424); General Surgery (F) (n = 230)	EE1	--	--	--	--
		EE2	-17.72	29.72 (6)	**<0.0000**	**<0.0000**
		EE3	--	--	--	--
		EE5	--	--	--	--
		EE6	--	--	--	--
		EE7	--	--	--	--
		EE4EE8	--	--	--	--
		EE9	-9.64	21.64 (6)	**0.0014**	**0.0014**
	GIM (R) (n = 424); General surgery subspecialty (F) (n = 350)	EE1	--	--	--	--
		EE2	--	--	--	--
		EE3	--	--	--	--
		EE5	--	--	--	--
		EE6	--	--	--	--
		EE7	--	--	--	--
		EE4EE8	--	--	--	--
		EE9	--	--	--	--
	GIM (R) (n = 424); Internal medicine subspecialty (F) (n = 711)	EE1	--	--	--	--
		EE2	--	--	--	--
		EE3	--	--	--	--
		EE5	--	--	--	--

		EE6	--	--	--	--
		EE7	--	--	--	--
		EE4EE8	--	--	--	--
		EE9	--	--	--	--
	GIM (R) (n = 424); Neurology(F) (n = 221)	EE1	--	--	--	--
		EE2	--	--	--	--
		EE3	--	--	--	--
		EE5	--	--	--	--
		EE6	--	--	--	--
		EE7	--	--	--	--
		EE4EE8	--	--	--	--
		EE9	--	--	--	--
	GIM (R) (n = 424); Obstetrics and gynecology (F) (n = 267)	EE1	--	--	--	--
		EE2	--	--	--	--
		EE3	--	--	--	--
		EE5	--	--	--	--
		EE6	--	--	--	--
		EE7	--	--	--	--
		EE4EE8	--	--	--	--
		EE9	--	--	--	--
	GIM (R) (n = 424); Ophthalmology (F) (n = 219)	EE1	--	--	--	--
		EE2	--	--	--	--
		EE3	--	--	--	--
		EE5	--	--	--	--
		EE6	--	--	--	--
		EE7	--	--	--	--
		EE4EE8	--	--	--	--
		EE9	--	--	--	--
	GIM (R); Orthopedic surgery) (n = 219)	EE1	--	--	--	--
		EE2	--	--	--	--
		EE3	--	--	--	--
		EE5	--	--	--	--
		EE6	--	--	--	--
		EE7	--	--	--	--
		EE4EE8	--	--	--	--
		EE9	--	--	--	--
	GIM (R) (n = 424); Pediatric subspecialty (F) (n = 293)	EE1	--	--	--	--
		EE2	--	--	--	--
		EE3	--	--	--	--
		EE5	--	--	--	--
		EE6	-6.16	18.16 (6)	**0.0058**	**0.0058**
		EE7	--	--	--	--
		EE4EE8	--	--	--	--
		EE9	--	--	--	--
	GIM (R) (n =424); Physical medicine and rehabilitation/preventive medicine/occupational medicine (F) (n = 267)	EE1	--	--	--	--
		EE2	--	--	--	--
		EE3	--	--	--	--
		EE5	--	--	--	--
		EE6	-10.22	22.22 (6)	**0.0011**	**0.0011**
		EE7	--	--	--	--
		EE4EE8	--	--	--	--
		EE9	--	--	--	--

Brady et al. (2021)

	GIM (n = 424) (R); Psychiatry (n = 525) (F)	EE1	--	--	--	--
		EE2	--	--	--	--
		EE3	--	--	--	--
		EE5	--	--	--	--
		EE6	-7.84	19.84 (6)	**0.0030**	**0.0030**
		EE7	--	--	--	--
		EE4EE8	--	--	--	--
		EE9	--	--	--	--
	GIM (R) (n =424) ; Radiology (F) (n = 230)	EE1	--	--	--	--
		EE2	--	--	--	--
		EE3	--	--	--	--
		EE5	--	--	--	--
		EE6	--	--	--	--
		EE7	--	--	--	--
		EE4EE8	--	--	--	--
		EE9	--	--	--	--

[a] "--" indicates that no DIF was detected in the backward sequential approach. Bolded p-values are significant at p < 0.05.

Table 2.2 Anchor item identification results: DP Scale

DIF Grouping Variable	***Reference group (n); focal group (n)***	***Item***	**Likelihood-ratio test for anchor item identification using a backward sequential approach [a]**			
			AIC difference	***X^2 (df)***	***p-value***	***B-H adjusted p-value***
**Sex**	Reference: Male (n = 4178); Focal: male (n = 2032)	mbi_dp1	-30.79	42.79 (6)	**<0.0000**	**<0.0000**
		mbi_dp2	-17.27	29.27 (6)	**<0.0000**	**<0.0000**
		mbi_dp3	--	--	--	--
		mbi_dp4	--	--	--	--
		mbi_dp5	--	--	--	--
**Age Category**	Reference: : ≥65 years (n = 1303); Focal: <35 years (n = 309)	mbi_dp1	--	--	--	--
		mbi_dp2	-3.25	15.25 (6)	**0.0184**	**0.0184**
		mbi_dp3	-9.81	21.81 (6)	**0.0013**	**0.0042**
		mbi_dp4	-8.69	20.69 (6)	**0.0021**	**0.0042**
		mbi_dp5	-4.25	16.25 (6)	**0.0125**	**0.0166**
	Reference: ≥65 years (n = 1303); Focal: 35-44 years (n = 1170)	mbi_dp1	--	--	--	--
		mbi_dp2	-10.89	22.89 (6)	**0.0008**	**0.0013**
		mbi_dp3	-15.27	27.27 (6)	**0.0001**	**0.0004**
		mbi_dp4	-9.38	21.38 (6)	**0.0016**	**0.0016**
		mbi_dp5	--	--	--	--
	Reference: ≥65 years (n = 1303); Focal: 45-54 years (n = 1345)	mbi_dp1	--	--	--	--
		mbi_dp2	-18.07	30.07 (6)	**<0.0000**	**<0.0000**
		mbi_dp3	-26.59	38.59 (6)	**<0.0000**	**<0.0000**
		mbi_dp4	-21.82	33.82 (6)	**<0.0000**	**<0.0000**
		mbi_dp5	--	--	--	--
	Reference: ≥65 years (n = 1303); Focal: 55-64 years (n = 2083)	mbi_dp1	--	--	--	--
		mbi_dp2	-3.56	15.56 (6)	**0.0163**	**0.0163**
		mbi_dp3	-14.54	26.54 (6)	**0.0002**	**0.0005**
		mbi_dp4	-6.08	18.08 (6)	**0.0060**	**0.0090**
		mbi_dp5	--	--	--	--
**Specialty**		mbi_dp1	-23.83	35.83 (6)	**<0.0000**	**<0.0000**

	GIM (R) (n = 438); Anesthesiology (F) (n = 224)	mbi_dp2	--	--	--	--
		mbi_dp3	--	--	--	--
		mbi_dp4	-25.10	37.10 (6)	**<0.0000**	**<0.0000**
		mbi_dp5	--	--	--	--
	GIM (R) (n = 438); Emergency medicine (F) (n = 331)	mbi_dp1	-13.15	25.15 (6)	**0.0003**	**0.0003**
		mbi_dp2	--	--	--	--
		mbi_dp3	-16.96	28.96 (6)	**<0.0000**	**0.0001**
		mbi_dp4	--	--	--	--
		mbi_dp5	--	--	--	--
	GIM (R) (n = 438); Family Medicine (F) (n = 508)	mbi_dp1	--	--	--	--
		mbi_dp2	--	--	--	--
		mbi_dp3	5.54	6.46 (6)	0.3738	0.3738
		mbi_dp4	--	--	--	--
		mbi_dp5	--	--	--	--
	General pediatrics (R); General internal medicine (F)	mbi_dp1	--	--	--	--
		mbi_dp2	--	--	--	--
		mbi_dp3	--	--	--	--
		mbi_dp4	--	--	--	--
		mbi_dp5	--	--	--	--
	General internal medicine (R) General surgery (F) (n = 239)	mbi_dp1	--	--	--	--
		mbi_dp2	--	--	--	--
		mbi_dp3	--	--	--	--
		mbi_dp4	--	--	--	--
		mbi_dp5	--	--	--	--
	GIM (R) (n = 438); General surgery subspecialty (F) (n = 355)	mbi_dp1	--	--	--	--
		mbi_dp2	--	--	--	--
		mbi_dp3	--	--	--	--
		mbi_dp4	-12.73	24.73 (6)	**0.0004**	**0.0004**
		mbi_dp5	-19.69	31.69 (6)	**<0.0000**	**<0.0000**
	GIM (R) (n = 438); Internal medicine subspecialty (F) (n = 730)	mbi_dp1	--	--	--	--
		mbi_dp2	--	--	--	--
		mbi_dp3	--	--	--	--
		mbi_dp4	--	--	--	--
		mbi_dp5	--	--	--	--
	GIM (R) (n = 438); Neurology (F) (n = 234)	mbi_dp1	--	--	--	--
		mbi_dp2	--	--	--	--
		mbi_dp3	2.04	9.96 (6)	0.1265	0.1265
		mbi_dp4	--	--	--	--
		mbi_dp5	--	--	--	--
	GIM (R) (n = 438); Obstetrics and gynecology (F) (n = 270)	mbi_dp1	--	--	--	--
		mbi_dp2	--	--	--	--
		mbi_dp3	--	--	--	--
		mbi_dp4	--	--	--	--
		mbi_dp5	--	--	--	--
	GIM (R) (n = 438); Ophthalmology (F) (n = 226)	mbi_dp1	--	--	--	--
		mbi_dp2	--	--	--	--
		mbi_dp3	--	--	--	--
		mbi_dp4	--	--	--	--
		mbi_dp5	--	--	--	--
	GIM (R) (n = 438); Orthopedic surgery (F) (n = 224)	mbi_dp1	--	--	--	--
		mbi_dp2	--	--	--	--
		mbi_dp3	--	--	--	--

		mbi_dp4	--	--	--	--
		mbi_dp5	--	--	--	--
	GIM (R) (n = 438); Pediatric subspecialty (F) (n = 301)	mbi_dp1	--	--	--	--
		mbi_dp2	--	--	--	--
		mbi_dp3	--	--	--	--
		mbi_dp4	--	--	--	--
		mbi_dp5	--	--	--	--
	GIM (R) (n = 438); Physical medicine and rehabilitation/preventiv e medicine/occupational medicine (F) (n = 263)	mbi_dp1	--	--	--	--
		mbi_dp2	--	--	--	--
		mbi_dp3	--	--	--	--
		mbi_dp4	--	--	--	--
		mbi_dp5	--	--	--	--
	GIM (R) (n = 438); Psychiatry (F) (n = 531)	mbi_dp1	--	--	--	--
		mbi_dp2	--	--	--	--
		mbi_dp3	-18.78	30.78 (6)	**<0.0000**	**<0.0000**
		mbi_dp4	--	--	--	--
		mbi_dp5	-19.04	31.04 (6)	**<0.0000**	**<0.0000**
	GIM (R) (n = 438); Radiology (F) (n = 240)	mbi_dp1	-22.90	32.90 (5)	**<0.0000**	**<0.0000**
		mbi_dp2	-18.79	30.79 (6)	**<0.0000**	**<0.0000**
		mbi_dp3	--	--	--	--
		mbi_dp4	--	--	--	--
		mbi_dp5	--	--	--	--

[a] *“--” indicates that no DIF was detected in the backward sequential approach. Bolded p-values are significant at p < 0.05.*

Table 2.3 Anchor item identification results: PA Scale

DIF Grouping Variable	***Reference group (n); focal group (n)***	***Item***	**Likelihood-ratio test for anchor item identification using a backward sequential approach** [a]			
			AIC difference	***X^2 (df)***	***p-value***	***B-H adjusted p-value***
**Sex**	Reference: male (n = 4048)<br><br>Focal: female (n = 1973)	mbi_pa1	-60.09	72.09 (6)	**<0.0000**	**<0.0000**
		mbi_pa2	--	--	--	--
		mbi_pa3	--	--	--	--
		mbi_pa4	-25.29	37.29 (6)	**<0.0000**	**<0.0000**
		mbi_pa5	--	--	--	--
		mbi_pa6	-5.60	17.60 (6)	**0.0073**	**0.0073**
		mbi_pa7	-12.03	24.03 (6)	**0.0005**	**0.0007**
		mbi_pa8	--	--	--	--
**Age Category**	Reference: ≥65 years (n = 1222)<br><br>Focal: <35 years (n = 307)	mbi_pa1	-17.34	25.34 (4)	**<0.0000**	**0.0001**
		mbi_pa2	-16.68	24.68 (4)	**<0.0000**	**0.0001**
		mbi_pa3	-11.56	21.56 (5)	**0.0006**	**0.0010**
		mbi_pa4	-21.88	31.88 (5)	**<0.0000**	**<0.0000**
		mbi_pa5	-7.56	15.56 (4)	**0.0037**	**0.0037**
		mbi_pa6	--	--	--	--
		mbi_pa7	-8.35	18.35 (5)	**0.0025**	**0.0030**
		mbi_pa8	--	--	--	--
	Reference: ≥65 years (n = 1222)<br><br>Focal: 35-44 years (n = 1158)	mbi_pa1	-40.05	52.05 (6)	**<0.0000**	**<0.0000**
		mbi_pa2	-42.30	52.30 (5)	**<0.0000**	**<0.0000**
		mbi_pa3	-9.64	21.64 (6)	**0.0014**	**0.0016**
		mbi_pa4	-27.35	39.35 (6)	**<0.0000**	**<0.0000**
		mbi_pa5	-28.48	40.48 (6)	**<0.0000**	**<0.0000**

		mbi_pa6	-5.81	17.81 (6)	**0.0067**	**0.0067**
		mbi_pa7	-20.21	32.21 (6)	**<0.0000**	**<0.0000**
		mbi_pa8	-12.45	22.45 (5)	**0.0004**	**0.0006**
	Reference: ≥65 years (n = 1222)<br><br>Focal: 45-54 years (n = 1318)	mbi_pa1	-41.99	51.99 (5)	**<0.0000**	**<0.0000**
		mbi_pa2	-36.22	48.22 (6)	**<0.0000**	**<0.0000**
		mbi_pa3	-4.12	16.12 (6)	**0.0131**	**0.0153**
		mbi_pa4	-18.11	30.11 (6)	**<0.0000**	**<0.0000**
		mbi_pa5	-17.54	29.54 (6)	**<0.0000**	**<0.0000**
		mbi_pa6	-2.89	14.89 (6)	**0.0211**	**0.0211**
		mbi_pa7	-5.57	17.57 (6)	**0.0074**	**0.0103**
		mbi_pa8	--	--	--	--
	Reference: ≥65 years (n = 1222)<br><br>Focal: 55-64 years (n = 2013)	mbi_pa1	-28.06	40.06 (6)	**<0.0000**	**<0.0000**
		mbi_pa2	-22.89	34.89 (6)	**<0.0000**	**<0.0000**
		mbi_pa3	-7.47	19.47 (6)	**0.0034**	**0.0046**
		mbi_pa4	-48.40	60.40 (6)	**<0.0000**	**<0.0000**
		mbi_pa5	-7.81	19.81 (6)	**0.0030**	**0.0046**
		mbi_pa6	-15.37	27.37 (6)	**0.0001**	**0.0002**
		mbi_pa7	-2.37	14.37 (6)	**0.0257**	**0.0294**
		mbi_pa8	-1.43	13.43 (6)	**0.0367**	**0.0367**
**Specialty**	GIM (R) (n = 427); Anesthesiology (n =215)	mbi_pa1	--	--	--	--
		mbi_pa2	--	--	--	--
		mbi_pa3	--	--	--	--
		mbi_pa4	--	--	--	--
		mbi_pa5	--	--	--	--
		mbi_pa6	--	--	--	--
		mbi_pa7	--	--	--	--
		mbi_pa8	--	--	--	--
	GIM (R) (n = 427); Emergency medicine (n=334) (F)	mbi_pa1	--	--	--	--
		mbi_pa2	--	--	--	--
		mbi_pa3	--	--	--	--
		mbi_pa4	-4.84	14.84 (5)	**0.0110**	**0.0110**
		mbi_pa5	--	--	--	--
		mbi_pa6	--	--	--	--
		mbi_pa7	--	--	--	--
		mbi_pa8	--	--	--	--
	GIM (R) (n = 427); Family Medicine (F) (n = 497)	mbi_pa1	--	--	--	--
		mbi_pa2	--	--	--	--
		mbi_pa3	--	--	--	--
		mbi_pa4	--	--	--	--
		mbi_pa5	--	--	--	--
		mbi_pa6	--	--	--	--
		mbi_pa7	--	--	--	--
		mbi_pa8	--	--	--	--
	GIM (n = 427) (R); General pediatrics (n = 337) (F)	mbi_pa1	--	--	--	--
		mbi_pa2	--	--	--	--
		mbi_pa3	--	--	--	--
		mbi_pa4	--	--	--	--
		mbi_pa5	--	--	--	--
		mbi_pa6	--	--	--	--
		mbi_pa7	--	--	--	--
		mbi_pa8	--	--	--	--
		mbi_pa1	--	--	--	--

	GIM (R) (n = 427); General surgery (F) (n = 240)	mbi_pa2	--	--	--	--
		mbi_pa3	--	--	--	--
		mbi_pa4	--	--	--	--
		mbi_pa5	--	--	--	--
		mbi_pa6	--	--	--	--
		mbi_pa7	--	--	--	--
		mbi_pa8	--	--	--	--
	GIM (R) (n = 427); General surgery subspecialty (F) (n = 339)	mbi_pa1	4.71	1.29 (3)	0.7311	0.7762
		mbi_pa2	--	--	--	--
		mbi_pa3	--	--	--	--
		mbi_pa4	--	--	--	--
		mbi_pa5	--	--	--	--
		mbi_pa6	--	--	--	--
		mbi_pa7	--	--	--	--
		mbi_pa8	7.50	2.50 (5)	0.7311	0.7762
	GIM (R) (n = 427); Internal medicine subspecialty (F) (n = 712)	mbi_pa1	--	--	--	--
		mbi_pa2	--	--	--	--
		mbi_pa3	--	--	--	--
		mbi_pa4	--	--	--	--
		mbi_pa5	--	--	--	--
		mbi_pa6	--	--	--	--
		mbi_pa7	--	--	--	--
		mbi_pa8	--	--	--	--
	GIM (R) (n = 427); Neurology (F) (n = 226)	mbi_pa1	--	--	--	--
		mbi_pa2	--	--	--	--
		mbi_pa3	--	--	--	--
		mbi_pa4	--	--	--	--
		mbi_pa5	--	--	--	--
		mbi_pa6	--	--	--	--
		mbi_pa7	--	--	--	--
		mbi_pa8	--	--	--	--
	GIM (R) (n = 427); Obstetrics and gynecology (F) (n = 270)	mbi_pa1	--	--	--	--
		mbi_pa2	--	--	--	--
		mbi_pa3	--	--	--	--
		mbi_pa4	--	--	--	--
		mbi_pa5	--	--	--	--
		mbi_pa6	--	--	--	--
		mbi_pa7	--	--	--	--
		mbi_pa8	--	--	--	--
	GIM (R); Ophthalmology (F) (n = 218)	mbi_pa1	--	--	--	--
		mbi_pa2	--	--	--	--
		mbi_pa3	--	--	--	--
		mbi_pa4	--	--	--	--
		mbi_pa5	--	--	--	--
		mbi_pa6	--	--	--	--
		mbi_pa7	-25.13	33.13 (4)	**<0.0000**	**<0.0000**
		mbi_pa8	-26.48	34.48 (4)	**<0.0000**	**<0.0000**
	GIM (R) (n = 427); Orthopedic Surgery (F) (n = 217)	mbi_pa1	--	--	--	--
		mbi_pa2	--	--	--	--
		mbi_pa3	--	--	--	--
		mbi_pa4	--	--	--	--
		mbi_pa5	--	--	--	--

		mbi_pa6	--	--	--	--
		mbi_pa7	--	--	--	--
		mbi_pa8	--	--	--	--
	GIM (R) (n = 427); Pediatric subspecialty (F) (n = 291)	mbi_pa1	--	--	--	--
		mbi_pa2	--	--	--	--
		mbi_pa3	--	--	--	--
		mbi_pa4	--	--	--	--
		mbi_pa5	--	--	--	--
		mbi_pa6	--	--	--	--
		mbi_pa7	--	--	--	--
		mbi_pa8	--	--	--	--
	GIM (R) (n = 427); Physical medicine and rehabilitation/preventive medicine/occupational medicine (F) (n = 250)	mbi_pa1	--	--	--	--
		mbi_pa2	--	--	--	--
		mbi_pa3	--	--	--	--
		mbi_pa4	--	--	--	--
		mbi_pa5	--	--	--	--
		mbi_pa6	--	--	--	--
		mbi_pa7	--	--	--	--
		mbi_pa8	--	--	--	--
	GIM (R) (n = 427); Psychiatry (F) (n = 505)	mbi_pa1	--	--	--	--
		mbi_pa2	--	--	--	--
		mbi_pa3	--	--	--	--
		mbi_pa4	--	--	--	--
		mbi_pa5	--	--	--	--
		mbi_pa6	--	--	--	--
		mbi_pa7	--	--	--	--
		mbi_pa8	4.76	1.24 (3)	0.7428	0.7428
	GIM (R) (n = 427); Radiology (F) (n = 229)	mbi_pa1	--	--	--	--
		mbi_pa2	--	--	--	--
		mbi_pa3	--	--	--	--
		mbi_pa4	--	--	--	--
		mbi_pa5	--	--	--	--
		mbi_pa6	--	--	--	--
		mbi_pa7	--	--	--	--
		mbi_pa8	--	--	--	--

[a] "--" indicates that no DIF was detected in the backward sequential approach. Bolded p-values are significant at $p < 0.05$.

Supplemental Appendix 3: Plots of Expected Item and Test Score Functions and Multi-Group IRT Item Parameter Estimates

Figure 3.1 Differential item and test functioning by gender – EE subscale

a) Expected total score functions for reference (male = 0) and focal (female = 1) groups

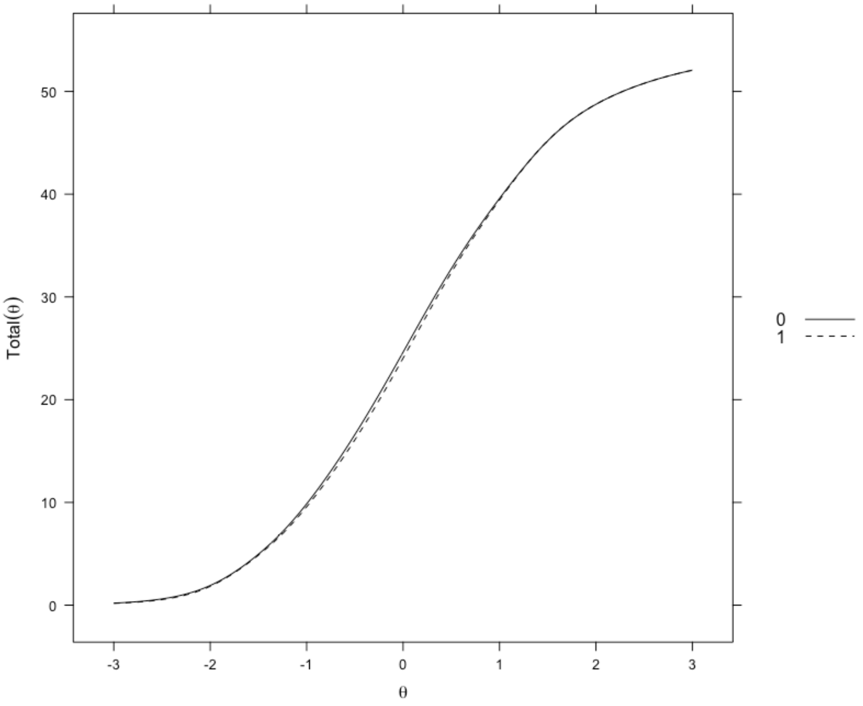

b) Expected item score functions for reference (male = 0) and focal (female = 1) groups

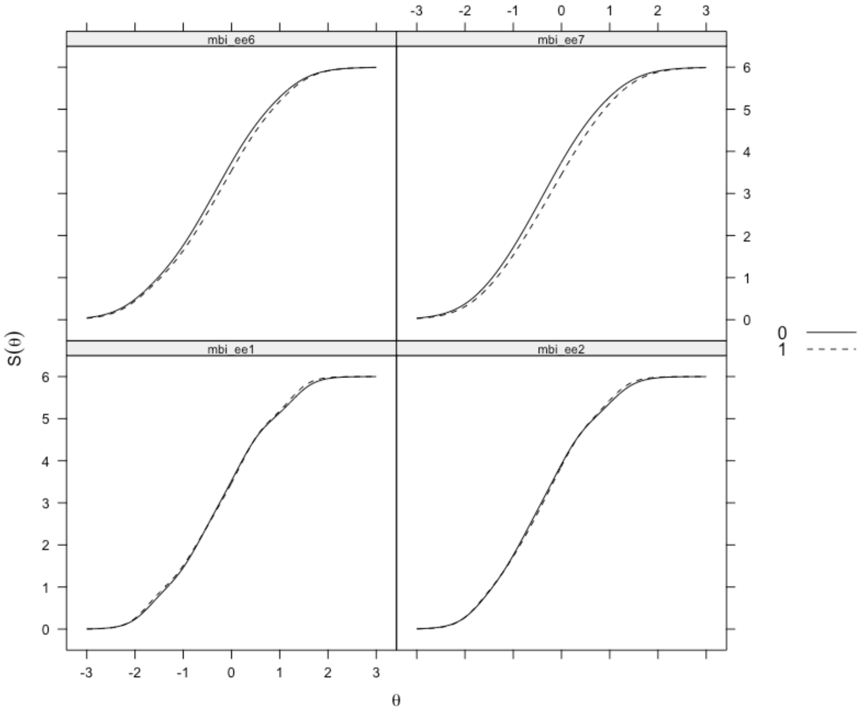

Table 3.1 Multi-group IRT item parameter estimates and standard errors (SE) by gender group (reference: male; focal: female) – EE subscale

	**Reference group item parameter estimates**	**Reference group SE**	**Focal group item parameter estimates**	**Focal group SE**
**ee1.a**	4.26	0.11	4.75	0.18
**ee1.b1**	-1.71	0.04	-1.77	0.06
**ee1.b2**	-0.89	0.02	-0.96	0.04
**ee1.b3**	-0.50	0.02	-0.48	0.03
**ee1.b4**	0.02	0.02	0.05	0.02
**ee1.b5**	0.37	0.02	0.39	0.02
**ee1.b6**	1.29	0.03	1.23	0.03
**ee2.a**	3.85	0.10	4.23	0.16
**ee2.b1**	-1.71	0.04	-1.75	0.06
**ee2.b2**	-1.05	0.03	-1.08	0.04
**ee2.b3**	-0.66	0.02	-0.59	0.03
**ee2.b4**	-0.16	0.02	-0.15	0.03
**ee2.b5**	0.17	0.02	0.21	0.02
**ee2.b6**	1.08	0.03	1.02	0.03
**ee3.a**	3.18	0.07	3.18	0.07

**ee3.b1**	-1.22	0.03	-1.22	0.03
**ee3.b2**	-0.59	0.02	-0.59	0.02
**ee3.b3**	-0.17	0.02	-0.17	0.02
**ee3.b4**	0.30	0.02	0.30	0.02
**ee3.b5**	0.66	0.02	0.66	0.02
**ee3.b6**	1.47	0.03	1.47	0.03
**ee5.a**	4.44	0.10	4.44	0.10
**ee5.b1**	-1.07	0.02	-1.07	0.02
**ee5.b2**	-0.34	0.02	-0.34	0.02
**ee5.b3**	0.00	0.02	0.00	0.02
**ee5.b4**	0.36	0.02	0.36	0.02
**ee5.b5**	0.69	0.02	0.69	0.02
**ee5.b6**	1.31	0.03	1.31	0.03
**ee6.a**	2.74	0.07	2.96	0.10
**ee6.b1**	-1.85	0.04	-1.85	0.07
**ee6.b2**	-1.00	0.03	-0.95	0.04
**ee6.b3**	-0.59	0.02	-0.51	0.03
**ee6.b4**	-0.12	0.02	-0.01	0.03
**ee6.b5**	0.24	0.02	0.37	0.03
**ee6.b6**	1.06	0.03	1.15	0.04
**ee7.a**	2.51	0.06	2.57	0.09
**ee7.b1**	-1.59	0.04	-1.53	0.06
**ee7.b2**	-0.98	0.03	-0.91	0.04
**ee7.b3**	-0.58	0.02	-0.46	0.03
**ee7.b4**	-0.13	0.02	0.04	0.03
**ee7.b5**	0.20	0.02	0.40	0.03
**ee7.b6**	1.00	0.03	1.11	0.04
**ee4ee8.a**	1.55	0.04	1.55	0.04
**ee4ee8.b1**	-1.12	0.03	-1.12	0.03
**ee4ee8.b2**	-0.58	0.03	-0.58	0.03
**ee4ee8.b3**	-0.03	0.02	-0.03	0.02
**ee4ee8.b4**	0.37	0.03	0.37	0.03
**ee4ee8.b5**	0.71	0.03	0.71	0.03
**ee4ee8.b6**	1.04	0.03	1.04	0.03
**ee4ee8.b7**	1.38	0.04	1.38	0.04
**ee4ee8.b8**	1.64	0.04	1.64	0.04
**ee4ee8.b9**	1.97	0.05	1.97	0.05
**ee4ee8.b10**	2.28	0.06	2.28	0.06

Brady et al. (2021)

**ee4ee8.b11**	2.80	0.07	2.80	0.07
**ee4ee8.b12**	3.39	0.09	3.39	0.09
**ee9.a**	2.48	0.06	2.48	0.06
**ee9.b1**	-0.30	0.02	-0.30	0.02
**ee9.b2**	0.40	0.02	0.40	0.02
**ee9.b3**	0.73	0.02	0.73	0.02
**ee9.b4**	1.08	0.03	1.08	0.03
**ee9.b5**	1.42	0.03	1.42	0.03
**ee9.b6**	2.12	0.04	2.12	0.04
**Latent Mean**	0.00	NA	0.27	0.03
**Latent Variance**	1.00	NA	0.86	0.04

Figure 3.2 Differential item and test functioning by age group (≥65 years and < 35 years) – EE subscale

a) Expected total score functions for reference (≥65 years = 0) and focal groups (< 35 years = 1)

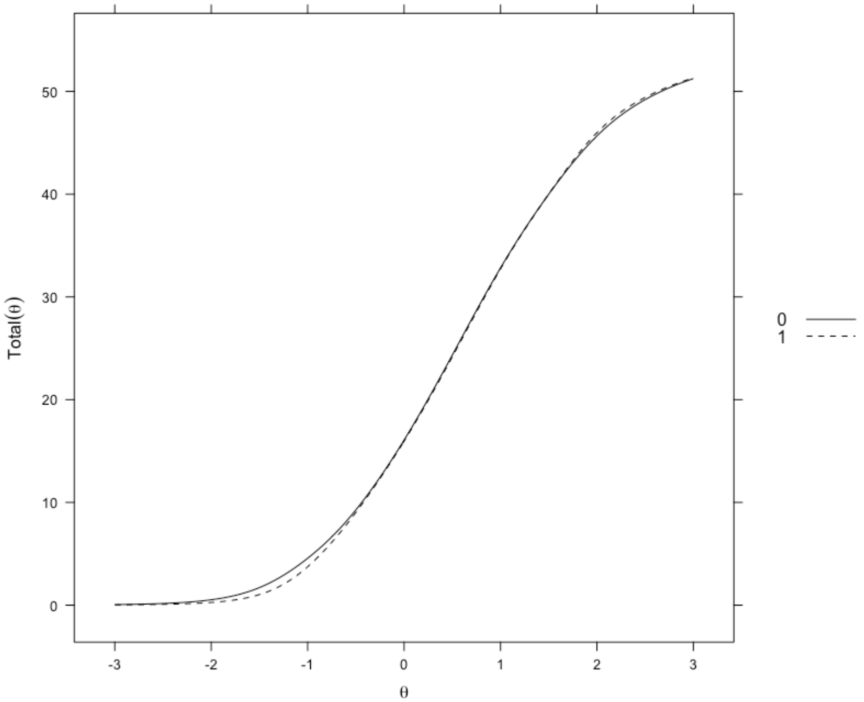

a) Expected item score functions for reference (≥65 years = 0) and focal groups (< 35 years = 1)

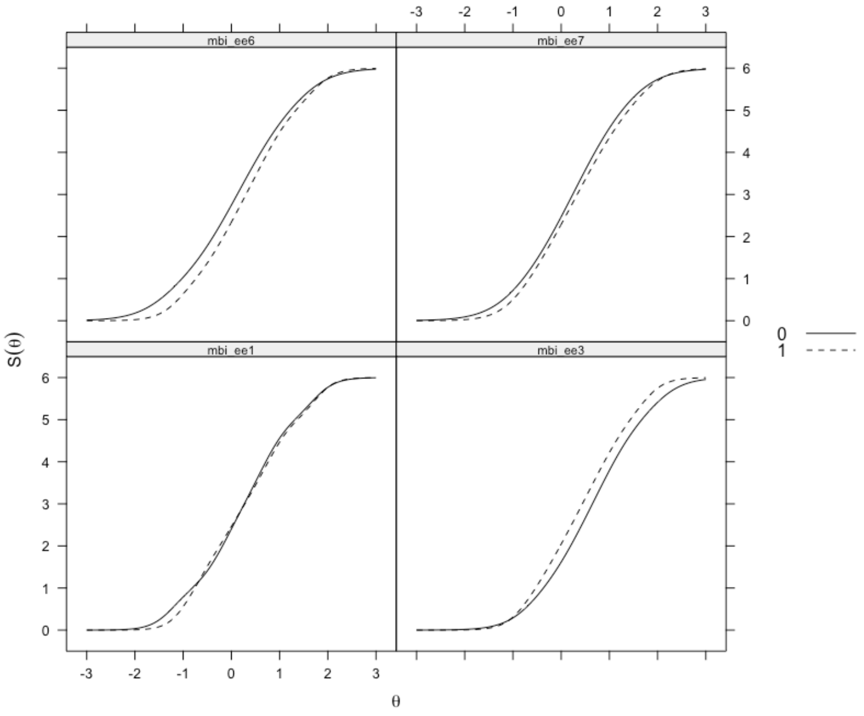

Table 3.2 Multi-group IRT item parameter estimates and standard errors (SE) by age group (reference: ≥65 years; focal: < 35 years) – EE subscale

	**Reference group item parameter estimates**	**Reference group SE**	**Focal group item parameter estimates**	**Focal group SE**
**ee1.a**	4.23	0.20	4.84	0.43
**ee1.b1**	-1.22	0.05	-0.96	0.11
**ee1.b2**	-0.34	0.04	-0.52	0.08
**ee1.b3**	0.03	0.04	0.03	0.06
**ee1.b4**	0.52	0.04	0.55	0.05
**ee1.b5**	0.86	0.04	0.96	0.06
**ee1.b6**	1.70	0.06	1.75	0.09
**ee2.a**	3.80	0.16	3.80	0.16
**ee2.b1**	-1.17	0.05	-1.17	0.05
**ee2.b2**	-0.52	0.04	-0.52	0.04
**ee2.b3**	-0.10	0.04	-0.10	0.04
**ee2.b4**	0.37	0.04	0.37	0.04
**ee2.b5**	0.72	0.04	0.72	0.04
**ee2.b6**	1.55	0.06	1.55	0.06
**ee3.a**	3.05	0.14	4.27	0.36

**ee3.b1**	-0.62	0.04	-0.76	0.10
**ee3.b2**	0.06	0.04	-0.24	0.07
**ee3.b3**	0.46	0.04	0.23	0.06
**ee3.b4**	0.87	0.05	0.66	0.06
**ee3.b5**	1.18	0.05	1.09	0.06
**ee3.b6**	1.96	0.08	1.71	0.09
**ee5.a**	4.10	0.18	4.10	0.18
**ee5.b1**	-0.55	0.04	-0.55	0.04
**ee5.b2**	0.16	0.04	0.16	0.04
**ee5.b3**	0.49	0.04	0.49	0.04
**ee5.b4**	0.86	0.04	0.86	0.04
**ee5.b5**	1.17	0.05	1.17	0.05
**ee5.b6**	1.82	0.06	1.82	0.06
**ee6.a**	2.57	0.11	3.92	0.34
**ee6.b1**	-1.32	0.06	-1.05	0.12
**ee6.b2**	-0.47	0.04	-0.35	0.07
**ee6.b3**	-0.08	0.04	0.08	0.06
**ee6.b4**	0.35	0.04	0.54	0.06
**ee6.b5**	0.69	0.05	0.91	0.06
**ee6.b6**	1.46	0.06	1.68	0.09
**ee7.a**	2.48	0.11	3.33	0.28
**ee7.b1**	-0.93	0.05	-0.84	0.11
**ee7.b2**	-0.31	0.04	-0.35	0.08
**ee7.b3**	0.06	0.04	0.12	0.06
**ee7.b4**	0.44	0.04	0.54	0.06
**ee7.b5**	0.75	0.05	1.00	0.07
**ee7.b6**	1.42	0.06	1.67	0.09
**ee4ee8.a**	1.75	0.08	1.75	0.08
**ee4ee8.b1**	-0.59	0.05	-0.59	0.05
**ee4ee8.b2**	-0.10	0.04	-0.10	0.04
**ee4ee8.b3**	0.42	0.05	0.42	0.05
**ee4ee8.b4**	0.83	0.05	0.83	0.05
**ee4ee8.b5**	1.15	0.06	1.15	0.06
**ee4ee8.b6**	1.43	0.07	1.43	0.07
**ee4ee8.b7**	1.79	0.08	1.79	0.08
**ee4ee8.b8**	1.98	0.09	1.98	0.09
**ee4ee8.b9**	2.32	0.10	2.32	0.10

Brady et al. (2021)

**ee4ee8.b10**	2.63	0.12	2.63	0.12
**ee4ee8.b11**	3.04	0.14	3.04	0.14
**ee4ee8.b12**	3.64	0.20	3.64	0.20
**ee9.a**	2.30	0.11	2.30	0.11
**ee9.b1**	0.17	0.04	0.17	0.04
**ee9.b2**	0.88	0.05	0.88	0.05
**ee9.b3**	1.19	0.05	1.19	0.05
**ee9.b4**	1.54	0.06	1.54	0.06
**ee9.b5**	1.88	0.08	1.88	0.08
**ee9.b6**	2.58	0.11	2.58	0.11
**Latent Mean**	0.00	NA	0.67	0.06
**Latent Variance**	1.00	NA	0.72	0.08

Figure 3.3 Differential item and test functioning by age group (≥65 years and 35-44 years) – EE subscale

a) Expected total score functions for reference (≥65 years = 0) and focal (35-44 years = 1) groups

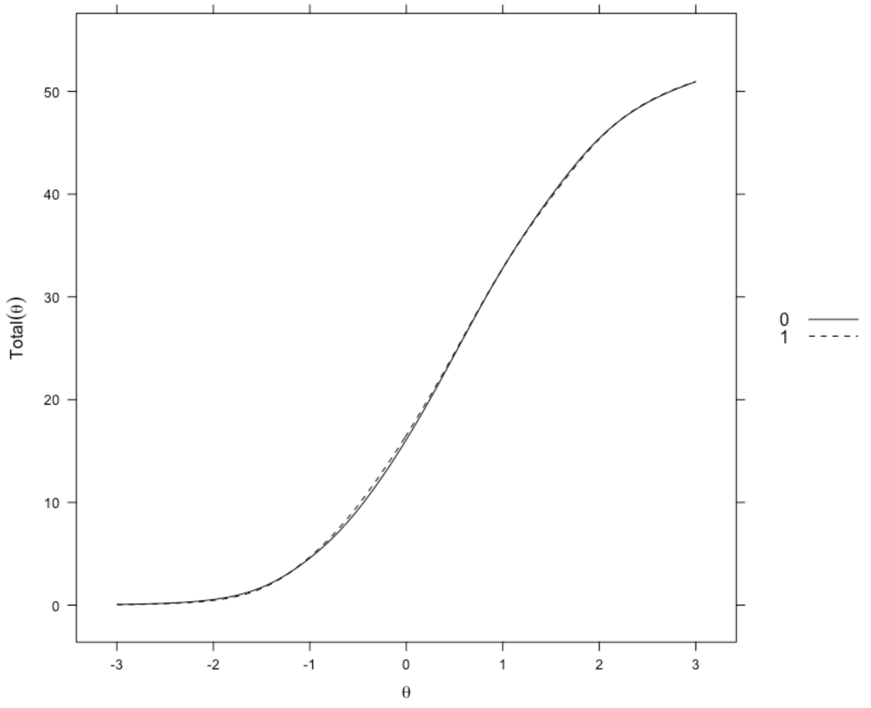

b) Expected item score functions for reference (≥65 years = 0) and focal (35-44 years = 1) groups

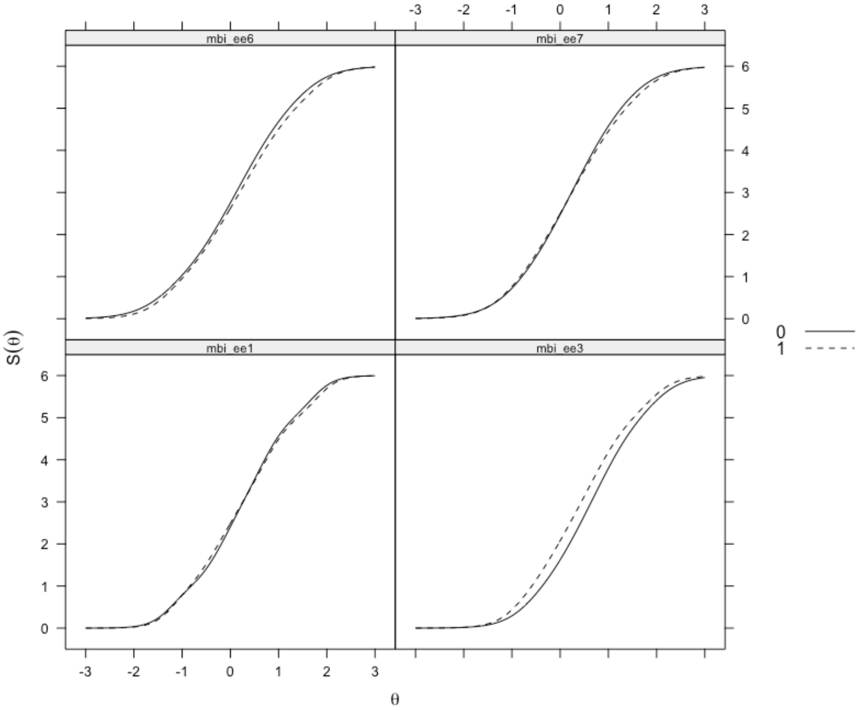

Table 3.3 Multi-group IRT item parameter estimates and standard errors (SE) by age group (reference: ≥65 years; focal: 35-44 years)

	**Reference group item parameter estimates**	**Reference group SE**	**Focal group item parameter estimates**	**Focal group SE**
**ee1.a**	4.23	0.20	4.44	0.23
**ee1.b1**	-1.21	0.05	-1.17	0.09
**ee1.b2**	-0.34	0.04	-0.47	0.05
**ee1.b3**	0.02	0.04	-0.01	0.04
**ee1.b4**	0.51	0.04	0.54	0.04
**ee1.b5**	0.85	0.04	0.92	0.04
**ee1.b6**	1.71	0.06	1.81	0.06
**ee2.a**	4.05	0.15	4.05	0.15
**ee2.b1**	-1.16	0.05	-1.16	0.05
**ee2.b2**	-0.53	0.04	-0.53	0.04
**ee2.b3**	-0.13	0.03	-0.13	0.03
**ee2.b4**	0.35	0.03	0.35	0.03
**ee2.b5**	0.68	0.04	0.68	0.04
**ee2.b6**	1.56	0.05	1.56	0.05

Brady et al. (2021)

**ee3.a**	3.04	0.14	3.52	0.17
**ee3.b1**	-0.62	0.04	-0.83	0.07
**ee3.b2**	0.06	0.04	-0.24	0.05
**ee3.b3**	0.45	0.04	0.22	0.04
**ee3.b4**	0.86	0.05	0.69	0.04
**ee3.b5**	1.18	0.05	1.04	0.05
**ee3.b6**	1.96	0.08	1.89	0.07
**ee5.a**	4.36	0.16	4.36	0.16
**ee5.b1**	-0.54	0.04	-0.54	0.04
**ee5.b2**	0.17	0.03	0.17	0.03
**ee5.b3**	0.49	0.03	0.49	0.03
**ee5.b4**	0.83	0.04	0.83	0.04
**ee5.b5**	1.19	0.04	1.19	0.04
**ee5.b6**	1.83	0.06	1.83	0.06
**ee6.a**	2.56	0.11	3.12	0.15
**ee6.b1**	-1.32	0.06	-1.30	0.10
**ee6.b2**	-0.47	0.04	-0.51	0.06
**ee6.b3**	-0.09	0.04	-0.06	0.05
**ee6.b4**	0.34	0.04	0.46	0.04
**ee6.b5**	0.69	0.05	0.86	0.04
**ee6.b6**	1.46	0.06	1.70	0.06
**ee7.a**	2.47	0.11	2.75	0.14
**ee7.b1**	-0.93	0.05	-1.02	0.08
**ee7.b2**	-0.31	0.04	-0.42	0.06
**ee7.b3**	0.06	0.04	0.01	0.05
**ee7.b4**	0.44	0.04	0.49	0.04
**ee7.b5**	0.75	0.05	0.86	0.05
**ee7.b6**	1.42	0.06	1.65	0.06
**ee4ee8.a**	1.61	0.06	1.61	0.06
**ee4ee8.b1**	-0.59	0.05	-0.59	0.05
**ee4ee8.b2**	-0.06	0.04	-0.06	0.04
**ee4ee8.b3**	0.46	0.04	0.46	0.04
**ee4ee8.b4**	0.86	0.05	0.86	0.05
**ee4ee8.b5**	1.16	0.05	1.16	0.05
**ee4ee8.b6**	1.48	0.06	1.48	0.06
**ee4ee8.b7**	1.85	0.07	1.85	0.07
**ee4ee8.b8**	2.09	0.08	2.09	0.08

Brady et al. (2021)

**ee4ee8.b9**	2.39	0.09	2.39	0.09
**ee4ee8.b10**	2.72	0.10	2.72	0.10
**ee4ee8.b11**	3.28	0.13	3.28	0.13
**ee4ee8.b12**	3.83	0.17	3.83	0.17
**ee9.a**	2.43	0.10	2.43	0.10
**ee9.b1**	0.16	0.04	0.16	0.04
**ee9.b2**	0.87	0.04	0.87	0.04
**ee9.b3**	1.20	0.05	1.20	0.05
**ee9.b4**	1.56	0.06	1.56	0.06
**ee9.b5**	1.93	0.07	1.93	0.07
**ee9.b6**	2.57	0.09	2.57	0.09
**Latent Mean**	0.00	NA	0.76	0.04
**Latent Variance**	1.00	NA	0.73	0.05

Figure 3.4 Differential item and test functioning by age group (≥65 years and 45-54 years) – EE subscale

a) Expected total score functions for reference (≥65 years = 0) and focal (45-54 years = 1) groups

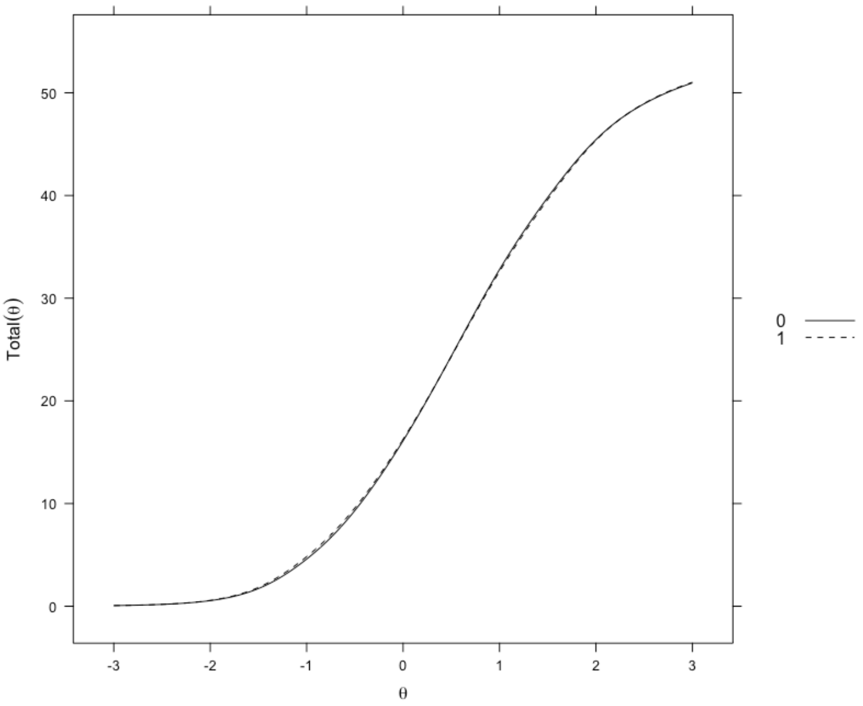

a) Expected item score functions for reference (≥65 years = 0) and focal (45-54) groups

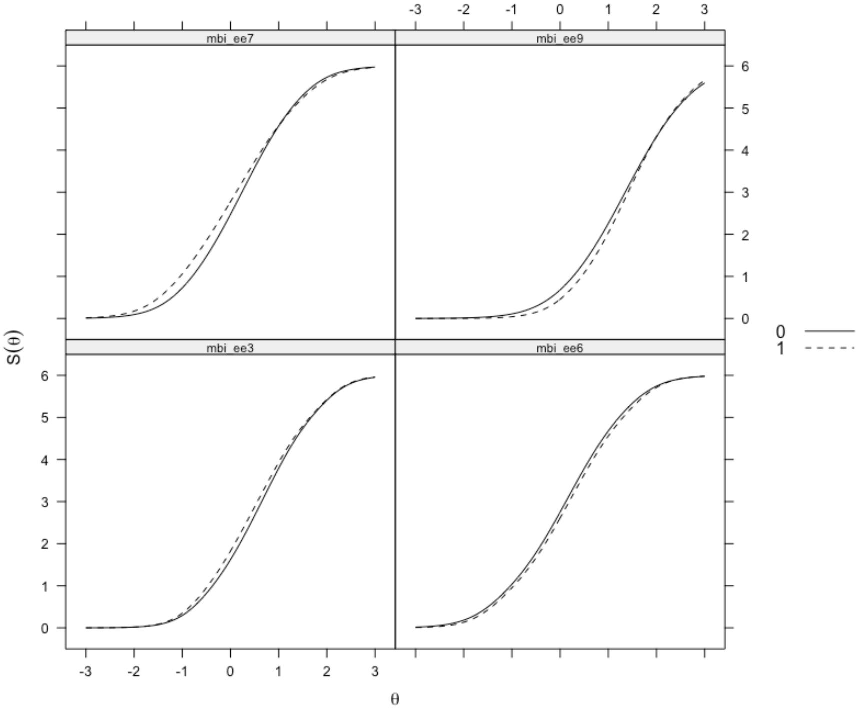

Table 3.4 Multi-group IRT item parameter estimates and standard errors (SE) by age groups (reference: ≥65 years; focal: 45-54 years)

	**Reference group item parameter estimates**	**Reference group SE**	**Focal group item parameter estimates**	**Focal group SE**
**ee1.a**	4.40	0.16	4.40	0.16
**ee1.b1**	-1.20	0.05	-1.20	0.05
**ee1.b2**	-0.36	0.04	-0.36	0.04
**ee1.b3**	0.03	0.03	0.03	0.03
**ee1.b4**	0.53	0.03	0.53	0.03
**ee1.b5**	0.87	0.04	0.87	0.04
**ee1.b6**	1.72	0.05	1.72	0.05
**ee2.a**	3.98	0.14	3.98	0.14
**ee2.b1**	-1.17	0.05	-1.17	0.05
**ee2.b2**	-0.50	0.04	-0.50	0.04
**ee2.b3**	-0.12	0.03	-0.12	0.03
**ee2.b4**	0.35	0.03	0.35	0.03
**ee2.b5**	0.69	0.04	0.69	0.04
**ee2.b6**	1.57	0.05	1.57	0.05

Brady et al. (2021)

**ee3.a**	3.04	0.14	3.22	0.15
**ee3.b1**	-0.62	0.04	-0.71	0.06
**ee3.b2**	0.06	0.04	-0.08	0.04
**ee3.b3**	0.46	0.04	0.33	0.04
**ee3.b4**	0.87	0.05	0.79	0.04
**ee3.b5**	1.19	0.05	1.17	0.05
**ee3.b6**	1.96	0.08	1.98	0.07
**ee5.a**	4.38	0.16	4.38	0.16
**ee5.b1**	-0.55	0.04	-0.55	0.04
**ee5.b2**	0.16	0.03	0.16	0.03
**ee5.b3**	0.50	0.03	0.50	0.03
**ee5.b4**	0.84	0.04	0.84	0.04
**ee5.b5**	1.17	0.04	1.17	0.04
**ee5.b6**	1.81	0.06	1.81	0.06
**ee6.a**	2.55	0.11	2.91	0.14
**ee6.b1**	-1.33	0.06	-1.31	0.09
**ee6.b2**	-0.47	0.04	-0.44	0.05
**ee6.b3**	-0.09	0.04	-0.05	0.04
**ee6.b4**	0.35	0.04	0.43	0.04
**ee6.b5**	0.69	0.05	0.81	0.04
**ee6.b6**	1.46	0.06	1.61	0.06
**ee7.a**	2.46	0.11	2.48	0.12
**ee7.b1**	-0.93	0.05	-1.24	0.09
**ee7.b2**	-0.31	0.04	-0.60	0.06
**ee7.b3**	0.06	0.04	-0.13	0.05
**ee7.b4**	0.44	0.04	0.40	0.04
**ee7.b5**	0.75	0.05	0.75	0.04
**ee7.b6**	1.42	0.06	1.56	0.06
**ee4ee8.a**	1.63	0.06	1.63	0.06
**ee4ee8.b1**	-0.59	0.05	-0.59	0.05
**ee4ee8.b2**	-0.05	0.04	-0.05	0.04
**ee4ee8.b3**	0.51	0.04	0.51	0.04
**ee4ee8.b4**	0.90	0.05	0.90	0.05
**ee4ee8.b5**	1.20	0.05	1.20	0.05
**ee4ee8.b6**	1.51	0.06	1.51	0.06
**ee4ee8.b7**	1.86	0.07	1.86	0.07
**ee4ee8.b8**	2.09	0.08	2.09	0.08

Brady et al. (2021)

**ee4ee8.b9**	2.43	0.09	2.43	0.09
**ee4ee8.b10**	2.72	0.10	2.72	0.10
**ee4ee8.b11**	3.17	0.12	3.17	0.12
**ee4ee8.b12**	3.75	0.15	3.75	0.15
**ee9.a**	2.17	0.11	2.67	0.13
**ee9.b1**	0.16	0.04	0.28	0.04
**ee9.b2**	0.87	0.05	0.95	0.05
**ee9.b3**	1.17	0.06	1.22	0.05
**ee9.b4**	1.51	0.07	1.59	0.06
**ee9.b5**	1.87	0.08	1.86	0.07
**ee9.b6**	2.51	0.12	2.61	0.10
**Latent Mean**	0.00	NA	0.81	0.04
**Latent Variance**	1.00	NA	0.82	0.05

Figure 3.5 Differential item and test functioning by age group (≥65 years and 55-64 years)– EE subscale

a) Expected total score functions for reference (≥65 years = 0) and focal groups (55-64 years = 1)

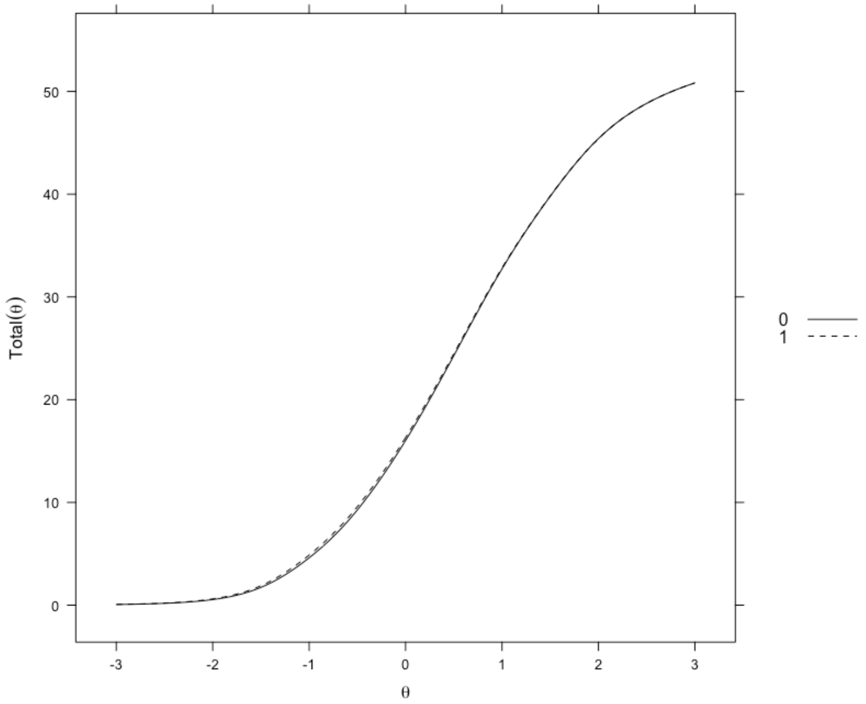

b) Expected item score functions for reference (≥65 years = 0) and focal groups (55-64 years = 1)

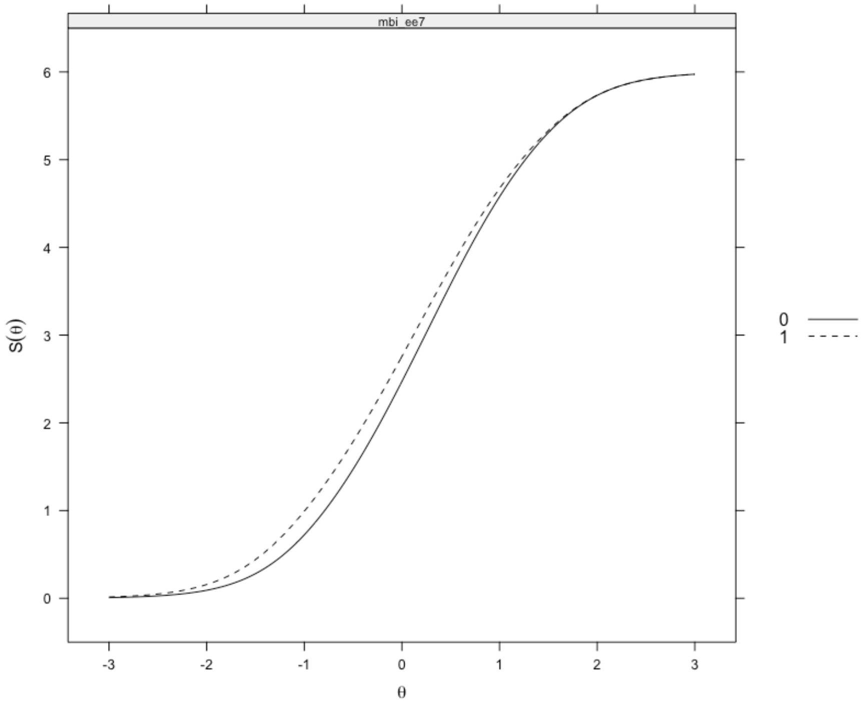

Table 3.5 Multi-group IRT item parameter estimates and standard errors (SE) by age group (reference: ≥65 years; focal: 55-64 years)

	**Reference group item parameter estimates**	**Reference group SE**	**Focal group item parameter estimates**	**Focal group SE**
**ee1.a**	4.52	0.15	4.52	0.15
**ee1.b1**	-1.21	0.05	-1.21	0.05
**ee1.b2**	-0.35	0.03	-0.35	0.03
**ee1.b3**	0.03	0.03	0.03	0.03
**ee1.b4**	0.52	0.03	0.52	0.03
**ee1.b5**	0.84	0.04	0.84	0.04
**ee1.b6**	1.73	0.05	1.73	0.05
**ee2.a**	3.93	0.13	3.93	0.13
**ee2.b1**	-1.17	0.05	-1.17	0.05
**ee2.b2**	-0.52	0.04	-0.52	0.04
**ee2.b3**	-0.10	0.03	-0.10	0.03
**ee2.b4**	0.36	0.03	0.36	0.03
**ee2.b5**	0.67	0.04	0.67	0.04

Brady et al. (2021)

**ee2.b6**	1.52	0.05	1.52	0.05
**ee3.a**	3.13	0.10	3.13	0.10
**ee3.b1**	-0.65	0.04	-0.65	0.04
**ee3.b2**	0.00	0.03	0.00	0.03
**ee3.b3**	0.40	0.03	0.40	0.03
**ee3.b4**	0.87	0.04	0.87	0.04
**ee3.b5**	1.20	0.04	1.20	0.04
**ee3.b6**	1.97	0.06	1.97	0.06
**ee5.a**	4.19	0.14	4.19	0.14
**ee5.b1**	-0.53	0.04	-0.53	0.04
**ee5.b2**	0.19	0.03	0.19	0.03
**ee5.b3**	0.51	0.03	0.51	0.03
**ee5.b4**	0.87	0.04	0.87	0.04
**ee5.b5**	1.15	0.04	1.15	0.04
**ee5.b6**	1.77	0.05	1.77	0.05
**ee6.a**	2.72	0.09	2.72	0.09
**ee6.b1**	-1.34	0.05	-1.34	0.05
**ee6.b2**	-0.45	0.04	-0.45	0.04
**ee6.b3**	-0.05	0.03	-0.05	0.03
**ee6.b4**	0.40	0.03	0.40	0.03
**ee6.b5**	0.72	0.04	0.72	0.04
**ee6.b6**	1.48	0.05	1.48	0.05
**ee7.a**	2.47	0.11	2.46	0.10
**ee7.b1**	-0.93	0.05	-1.18	0.07
**ee7.b2**	-0.31	0.04	-0.51	0.05
**ee7.b3**	0.06	0.04	-0.10	0.04
**ee7.b4**	0.44	0.04	0.37	0.04
**ee7.b5**	0.75	0.05	0.68	0.04
**ee7.b6**	1.41	0.06	1.44	0.05
**ee4ee8.a**	1.58	0.06	1.58	0.06
**ee4ee8.b1**	-0.63	0.05	-0.63	0.05
**ee4ee8.b2**	-0.08	0.04	-0.08	0.04
**ee4ee8.b3**	0.48	0.04	0.48	0.04
**ee4ee8.b4**	0.87	0.04	0.87	0.04
**ee4ee8.b5**	1.24	0.05	1.24	0.05
**ee4ee8.b6**	1.54	0.06	1.54	0.06
**ee4ee8.b7**	1.89	0.07	1.89	0.07

Brady et al. (2021)

**ee4ee8.b8**	2.16	0.07	2.16	0.07
**ee4ee8.b9**	2.47	0.08	2.47	0.08
**ee4ee8.b10**	2.78	0.10	2.78	0.10
**ee4ee8.b11**	3.36	0.12	3.36	0.12
**ee4ee8.b12**	3.86	0.15	3.86	0.15
**ee9.a**	2.39	0.08	2.39	0.08
**ee9.b1**	0.19	0.04	0.19	0.04
**ee9.b2**	0.88	0.04	0.88	0.04
**ee9.b3**	1.21	0.05	1.21	0.05
**ee9.b4**	1.53	0.05	1.53	0.05
**ee9.b5**	1.87	0.06	1.87	0.06
**ee9.b6**	2.53	0.08	2.53	0.08
**Latent Mean**	0.00	NA	0.70	0.04
**Latent Variance**	1.00	NA	0.82	0.05

Figure 3.6 Differential item and test functioning by specialty group (General Internal Medicine and Anesthesiology) – EE subscale

a) Expected total score functions for reference (General Internal Medicine) and focal (Anesthesiology) groups

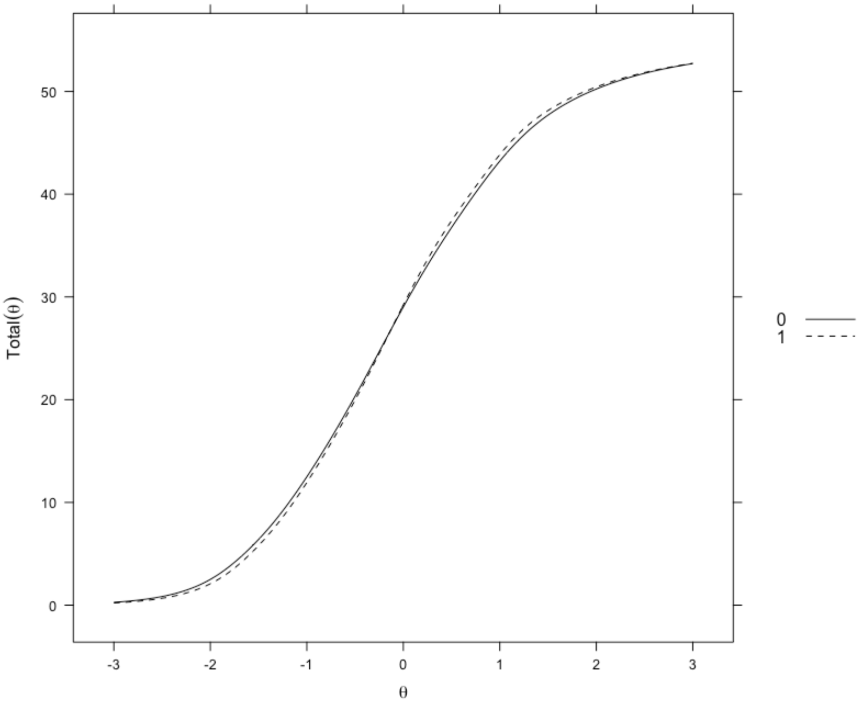

b) Expected item score functions for reference (General Internal Medicine) and focal (Anesthesiology) groups

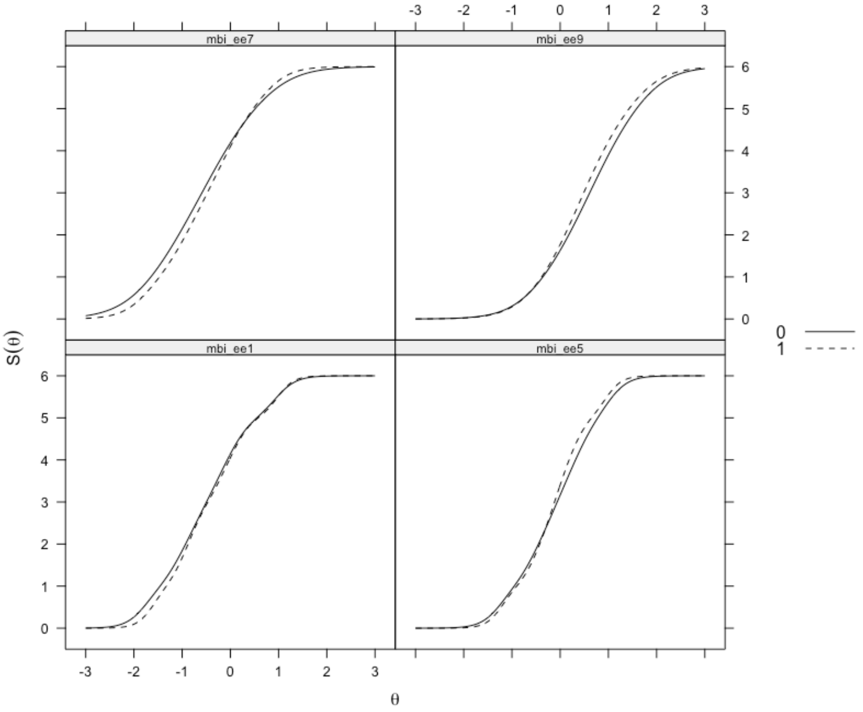

Table 3.6 Multi-group IRT item parameter estimates and standard errors (SE) by specialty group (reference: General Internal Medicine; focal: Anesthesiology) – EE subscale

	**Reference group item parameter estimates**	**Reference group SE**	**Focal group item parameter estimates**	**Focal group SE**
**ee1.a**	4.69	0.38	5.84	0.67
**ee1.b1**	-1.76	0.11	-1.61	0.13
**ee1.b2**	-1.10	0.08	-1.01	0.09
**ee1.b3**	-0.73	0.07	-0.74	0.08
**ee1.b4**	-0.26	0.06	-0.21	0.07
**ee1.b5**	0.09	0.06	0.15	0.07
**ee1.b6**	0.95	0.08	0.98	0.11
**ee2.a**	5.06	0.37	5.06	0.37
**ee2.b1**	-1.73	0.10	-1.73	0.10
**ee2.b2**	-1.23	0.08	-1.23	0.08
**ee2.b3**	-0.84	0.07	-0.84	0.07
**ee2.b4**	-0.43	0.06	-0.43	0.06
**ee2.b5**	-0.12	0.06	-0.12	0.06
**ee2.b6**	0.68	0.07	0.68	0.07

Brady et al. (2021)

**ee3.a**	3.14	0.20	3.14	0.20
**ee3.b1**	-1.48	0.10	-1.48	0.10
**ee3.b2**	-0.88	0.07	-0.88	0.07
**ee3.b3**	-0.52	0.06	-0.52	0.06
**ee3.b4**	-0.03	0.06	-0.03	0.06
**ee3.b5**	0.28	0.06	0.28	0.06
**ee3.b6**	1.17	0.09	1.17	0.09
**ee5.a**	4.64	0.37	5.90	0.66
**ee5.b1**	-1.24	0.09	-1.21	0.10
**ee5.b2**	-0.60	0.07	-0.55	0.07
**ee5.b3**	-0.24	0.06	-0.23	0.07
**ee5.b4**	0.13	0.06	0.00	0.07
**ee5.b5**	0.46	0.07	0.30	0.08
**ee5.b6**	1.02	0.08	0.95	0.10
**ee6.a**	2.79	0.18	2.79	0.18
**ee6.b1**	-2.11	0.14	-2.11	0.14
**ee6.b2**	-1.21	0.09	-1.21	0.09
**ee6.b3**	-0.80	0.07	-0.80	0.07
**ee6.b4**	-0.29	0.06	-0.29	0.06
**ee6.b5**	0.05	0.06	0.05	0.06
**ee6.b6**	0.74	0.08	0.74	0.08
**ee7.a**	2.28	0.18	3.43	0.36
**ee7.b1**	-1.70	0.13	-1.74	0.16
**ee7.b2**	-1.20	0.10	-1.06	0.10
**ee7.b3**	-0.79	0.09	-0.71	0.08
**ee7.b4**	-0.36	0.08	-0.21	0.08
**ee7.b5**	-0.08	0.07	0.05	0.08
**ee7.b6**	0.69	0.09	0.72	0.11
**ee4ee8.a**	1.64	0.12	1.64	0.12
**ee4ee8.b1**	-1.21	0.10	-1.21	0.10
**ee4ee8.b2**	-0.73	0.08	-0.73	0.08
**ee4ee8.b3**	-0.24	0.07	-0.24	0.07
**ee4ee8.b4**	0.15	0.08	0.15	0.08
**ee4ee8.b5**	0.45	0.08	0.45	0.08
**ee4ee8.b6**	0.75	0.09	0.75	0.09
**ee4ee8.b7**	1.18	0.11	1.18	0.11
**ee4ee8.b8**	1.39	0.12	1.39	0.12

Brady et al. (2021)

**ee4ee8.b9**	1.65	0.13	1.65	0.13
**ee4ee8.b10**	1.91	0.15	1.91	0.15
**ee4ee8.b11**	2.38	0.19	2.38	0.19
**ee4ee8.b12**	3.13	0.27	3.13	0.27
**ee9.a**	2.58	0.20	2.68	0.31
**ee9.b1**	-0.49	0.08	-0.46	0.09
**ee9.b2**	0.11	0.07	0.11	0.09
**ee9.b3**	0.45	0.07	0.33	0.10
**ee9.b4**	0.78	0.08	0.55	0.11
**ee9.b5**	1.10	0.09	0.96	0.13
**ee9.b6**	1.72	0.12	1.60	0.20
**Latent Mean**	0.00	NA	-0.19	0.08
**Latent Variance**	1.00	NA	0.65	0.09

Figure 3.7 Differential item and test functioning by specialty group (General Internal Medicine and Emergency Medicine) – EE subscale

a) Expected total score functions for reference (General Internal Medicine) and focal (Emergency Medicine) groups

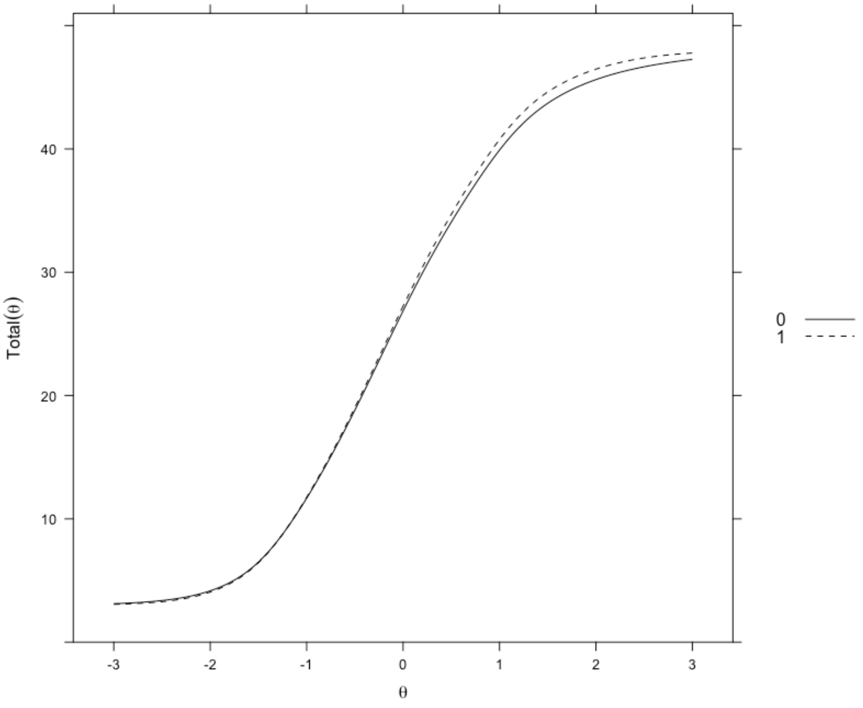

b) Expected item score functions for reference (General Internal Medicine) and focal (Anesthesiology) groups

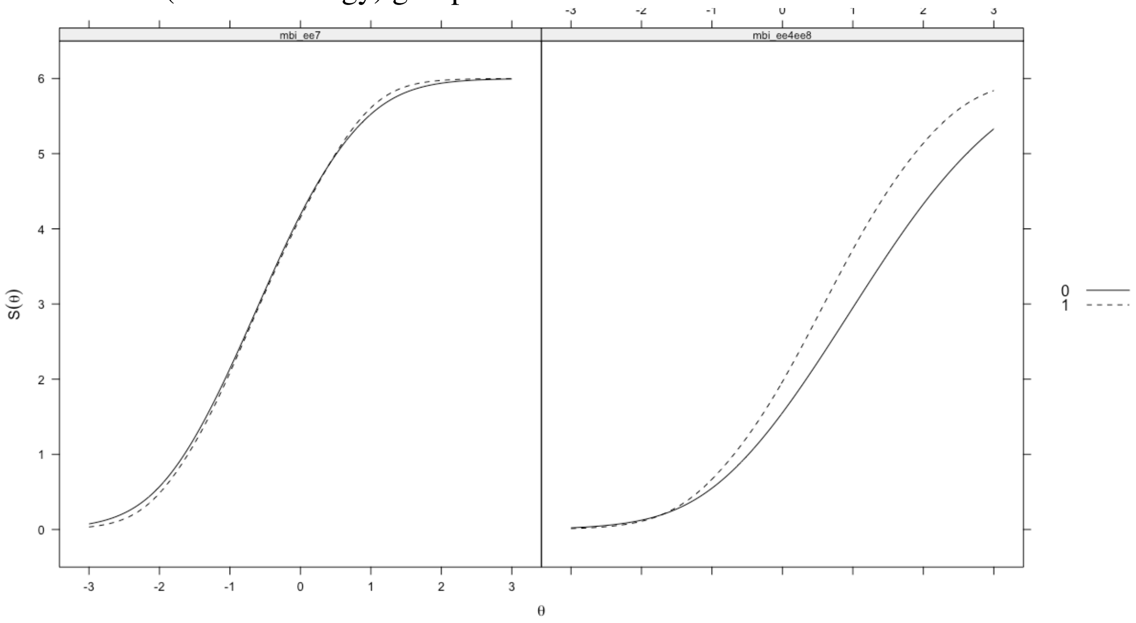

Table 3.7 Multi-group IRT item parameter estimates and standard errors (SE) by specialty group (reference: General Internal Medicine; focal: Emergency Medicine) – EE subscale

	**Reference group item parameter estimates**	**Reference group SE**	**Focal group item parameter estimates**	**Focal group SE**
**ee1.a**	4.86	0.33	4.86	0.33
**ee1.b1**	-1.17	0.08	-1.17	0.08
**ee1.b2**	-0.72	0.06	-0.72	0.06
**ee1.b3**	-0.25	0.06	-0.25	0.06
**ee1.b4**	0.11	0.06	0.11	0.06
**ee1.b5**	0.97	0.07	0.97	0.07
**ee2.a**	4.54	0.31	4.54	0.31
**ee2.b1**	-1.21	0.08	-1.21	0.08
**ee2.b2**	-0.82	0.07	-0.82	0.07
**ee2.b3**	-0.42	0.06	-0.42	0.06
**ee2.b4**	-0.10	0.06	-0.10	0.06
**ee2.b5**	0.66	0.06	0.66	0.06
**ee3.a**	3.11	0.19	3.11	0.19
**ee3.b1**	-1.50	0.10	-1.50	0.10
**ee3.b2**	-0.88	0.07	-0.88	0.07
**ee3.b3**	-0.44	0.06	-0.44	0.06

**ee3.b4**	0.03	0.06	0.03	0.06
**ee3.b5**	0.39	0.06	0.39	0.06
**ee3.b6**	1.20	0.08	1.20	0.08
**ee5.a**	4.56	0.29	4.56	0.29
**ee5.b1**	-1.28	0.08	-1.28	0.08
**ee5.b2**	-0.57	0.06	-0.57	0.06
**ee5.b3**	-0.21	0.06	-0.21	0.06
**ee5.b4**	0.14	0.06	0.14	0.06
**ee5.b5**	0.46	0.06	0.46	0.06
**ee5.b6**	1.00	0.07	1.00	0.07
**ee6.a**	2.86	0.18	2.86	0.18
**ee6.b1**	-1.23	0.09	-1.23	0.09
**ee6.b2**	-0.84	0.07	-0.84	0.07
**ee6.b3**	-0.36	0.06	-0.36	0.06
**ee6.b4**	-0.01	0.06	-0.01	0.06
**ee6.b5**	0.73	0.07	0.73	0.07
**ee7.a**	2.54	0.16	2.54	0.16
**ee7.b1**	-1.76	0.12	-1.76	0.12
**ee7.b2**	-1.19	0.09	-1.19	0.09
**ee7.b3**	-0.81	0.07	-0.81	0.07
**ee7.b4**	-0.36	0.06	-0.36	0.06
**ee7.b5**	0.04	0.06	0.04	0.06
**ee7.b6**	0.72	0.07	0.72	0.07
**ee4ee8.a**	1.87	0.13	1.87	0.13
**ee4ee8.b1**	-0.82	0.08	-0.82	0.08
**ee4ee8.b2**	0.03	0.07	0.03	0.07
**ee4ee8.b3**	0.56	0.08	0.56	0.08
**ee4ee8.b4**	1.12	0.09	1.12	0.09
**ee4ee8.b5**	1.67	0.12	1.67	0.12
**ee4ee8.b6**	2.62	0.19	2.62	0.19
**ee9.a**	2.55	0.17	2.55	0.17
**ee9.b1**	-0.52	0.07	-0.52	0.07
**ee9.b2**	0.17	0.06	0.17	0.06
**ee9.b3**	0.48	0.07	0.48	0.07
**ee9.b4**	0.82	0.07	0.82	0.07
**ee9.b5**	1.15	0.09	1.15	0.09
**ee9.b6**	1.77	0.11	1.77	0.11

Brady et al. (2021)

**Latent Mean**	0.00	NA	0.04	0.07
**Latent Variance**	1.00	NA	0.73	0.09

Figure 3.8 Differential item and test functioning by specialty group (General Internal Medicine and Family Medicine) – EE subscale

a) Expected total score functions for reference (General Internal Medicine) and focal (Family Medicine) groups

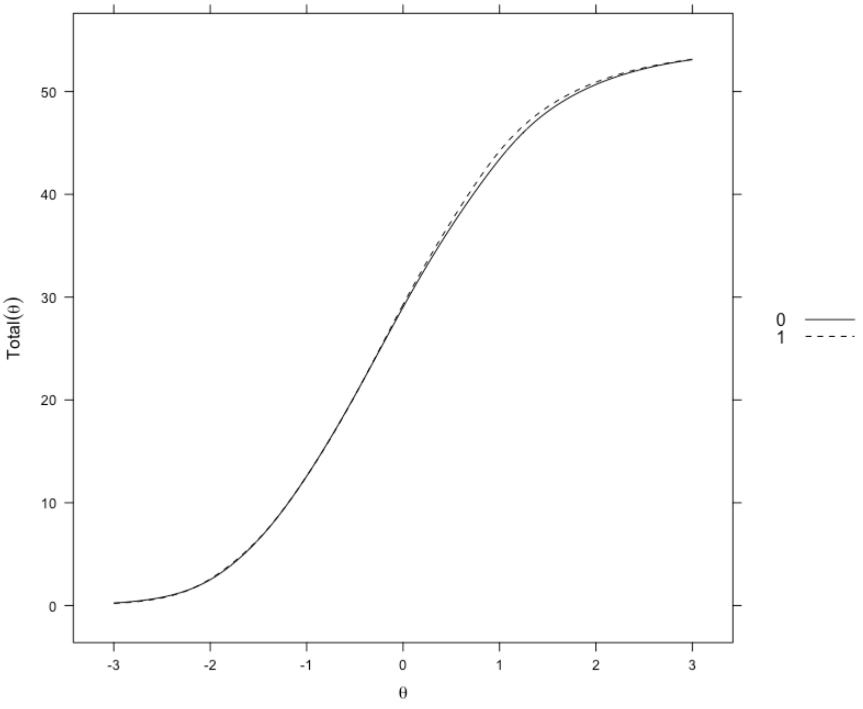

b) Expected item score functions for reference (General Internal Medicine) and focal (Family Medicine) groups

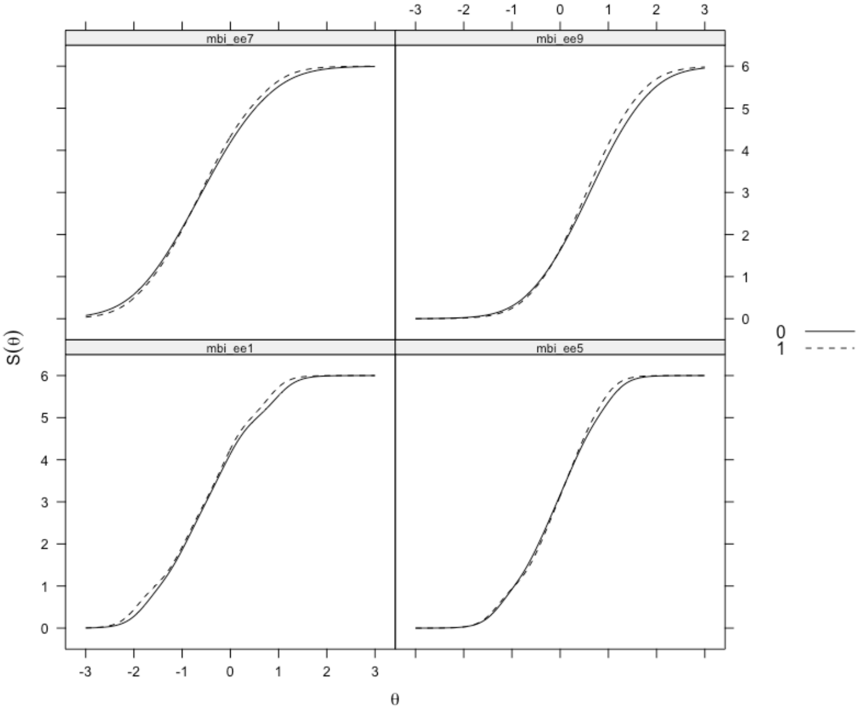

,

Table 3.8 Multi-group IRT item parameter estimates and standard errors (SE) by specialty group (reference: General Internal Medicine; focal: Family Medicine) – EE subscale

	**Reference group item parameter estimates**	**Reference group SE**	**Focal group item parameter estimates**	**Focal group SE**
**ee1.a**	4.55	0.37	5.05	0.42
**ee1.b1**	-1.77	0.11	-1.94	0.14
**ee1.b2**	-1.11	0.08	-1.14	0.09
**ee1.b3**	-0.73	0.07	-0.77	0.07
**ee1.b4**	-0.27	0.06	-0.26	0.06
**ee1.b5**	0.09	0.06	0.02	0.06
**ee1.b6**	0.96	0.08	0.80	0.08
**ee2.a**	4.17	0.26	4.17	0.26
**ee2.b1**	-1.76	0.10	-1.76	0.10
**ee2.b2**	-1.26	0.08	-1.26	0.08
**ee2.b3**	-0.90	0.07	-0.90	0.07
**ee2.b4**	-0.49	0.06	-0.49	0.06
**ee2.b5**	-0.12	0.06	-0.12	0.06
**ee2.b6**	0.67	0.07	0.67	0.07

Brady et al. (2021)

**ee3.a**	3.10	0.18	3.10	0.18
**ee3.b1**	-1.37	0.09	-1.37	0.09
**ee3.b2**	-0.78	0.07	-0.78	0.07
**ee3.b3**	-0.41	0.06	-0.41	0.06
**ee3.b4**	0.07	0.06	0.07	0.06
**ee3.b5**	0.43	0.06	0.43	0.06
**ee3.b6**	1.22	0.08	1.22	0.08
**ee5.a**	4.74	0.38	5.10	0.42
**ee5.b1**	-1.24	0.09	-1.30	0.09
**ee5.b2**	-0.60	0.07	-0.54	0.07
**ee5.b3**	-0.25	0.06	-0.23	0.06
**ee5.b4**	0.13	0.06	0.14	0.06
**ee5.b5**	0.46	0.07	0.44	0.07
**ee5.b6**	1.03	0.08	0.88	0.08
**ee6.a**	2.84	0.17	2.84	0.17
**ee6.b1**	-2.05	0.12	-2.05	0.12
**ee6.b2**	-1.27	0.08	-1.27	0.08
**ee6.b3**	-0.90	0.07	-0.90	0.07
**ee6.b4**	-0.38	0.06	-0.38	0.06
**ee6.b5**	-0.04	0.06	-0.04	0.06
**ee6.b6**	0.72	0.07	0.72	0.07
**ee7.a**	2.27	0.18	2.91	0.22
**ee7.b1**	-1.71	0.13	-1.82	0.14
**ee7.b2**	-1.20	0.10	-1.15	0.10
**ee7.b3**	-0.79	0.09	-0.81	0.08
**ee7.b4**	-0.36	0.08	-0.43	0.07
**ee7.b5**	-0.08	0.07	-0.07	0.07
**ee7.b6**	0.70	0.09	0.68	0.08
**ee4ee8.a**	1.78	0.11	1.78	0.11
**ee4ee8.b1**	-1.26	0.10	-1.26	0.10
**ee4ee8.b2**	-0.77	0.08	-0.77	0.08
**ee4ee8.b3**	-0.29	0.07	-0.29	0.07
**ee4ee8.b4**	0.09	0.07	0.09	0.07
**ee4ee8.b5**	0.39	0.07	0.39	0.07
**ee4ee8.b6**	0.67	0.08	0.67	0.08
**ee4ee8.b7**	1.02	0.09	1.02	0.09
**ee4ee8.b8**	1.27	0.10	1.27	0.10

Brady et al. (2021)

**ee4ee8.b9**	1.55	0.11	1.55	0.11
**ee4ee8.b10**	1.77	0.12	1.77	0.12
**ee4ee8.b11**	2.19	0.15	2.19	0.15
**ee4ee8.b12**	2.70	0.18	2.70	0.18
**ee9.a**	2.61	0.21	2.87	0.23
**ee9.b1**	-0.49	0.08	-0.47	0.07
**ee9.b2**	0.11	0.07	0.09	0.07
**ee9.b3**	0.45	0.07	0.41	0.07
**ee9.b4**	0.78	0.08	0.70	0.08
**ee9.b5**	1.10	0.09	0.99	0.09
**ee9.b6**	1.71	0.12	1.54	0.12
**Latent Mean**	0.00	NA	0.02	0.07
**Latent Variance**	1.00	NA	0.91	0.10

Figure 3.9 Differential item and test functioning by specialty group (General Internal Medicine and General Pediatrics) – EE subscale

a) Expected total score functions for reference (General Internal Medicine) and focal (General Pediatrics) groups

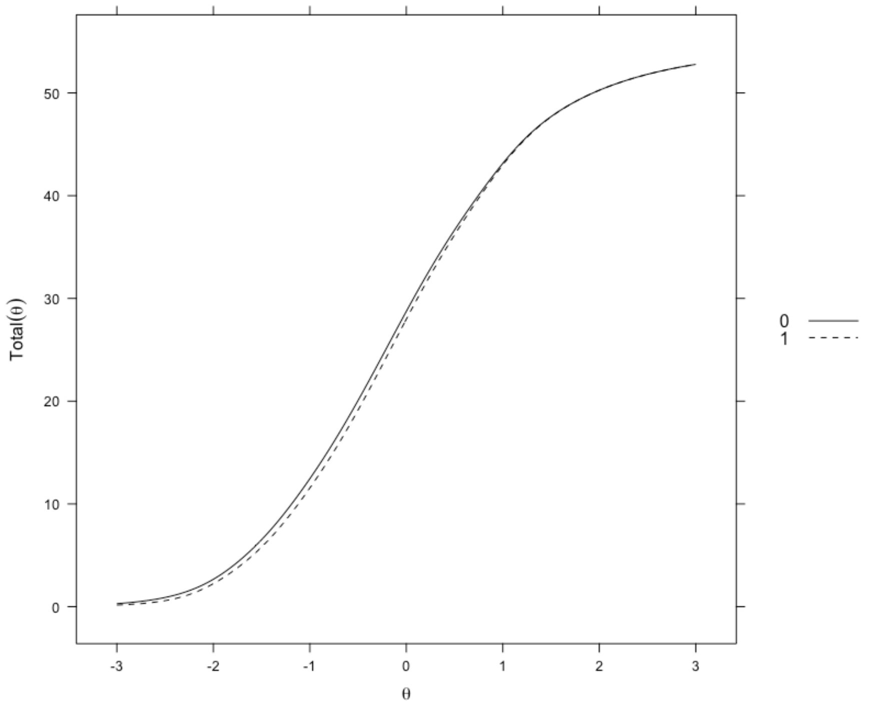

b) Expected item score functions for reference (General Internal Medicine) and focal (General Pediatrics) groups

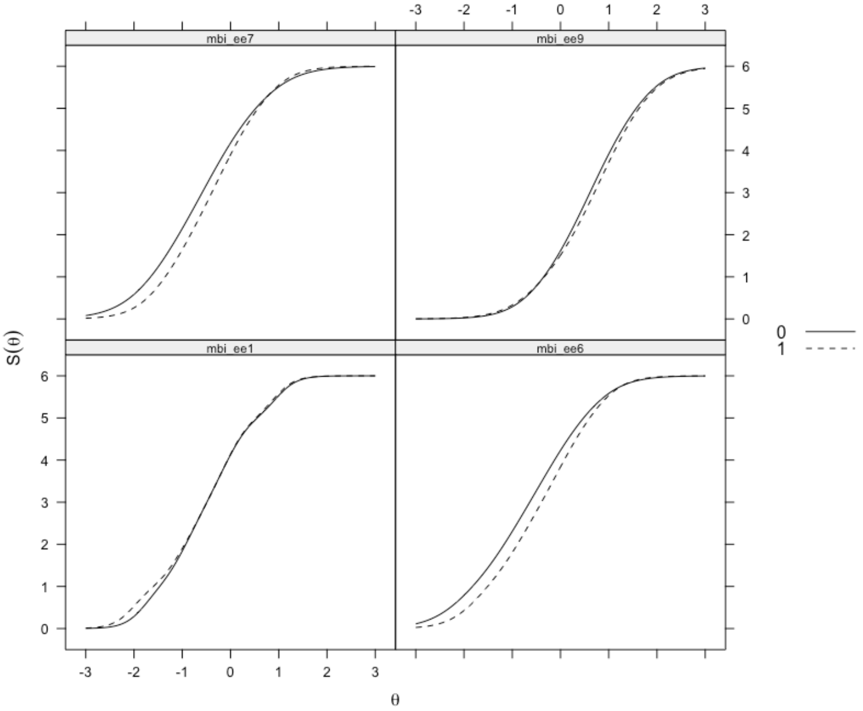

Table 3.9 Multi-group IRT item parameter estimates and standard errors (SE) by specialty group (reference: General Internal Medicine; focal: General Pediatrics) – EE subscale

	**Reference group item parameter estimates**	**Reference group SE**	**Focal group item parameter estimates**	**Focal group SE**
**ee1.a**	4.57	0.37	4.59	0.42
**ee1.b1**	-1.78	0.11	-2.01	0.14
**ee1.b2**	-1.10	0.08	-1.15	0.09
**ee1.b3**	-0.72	0.07	-0.70	0.07
**ee1.b4**	-0.25	0.06	-0.25	0.07
**ee1.b5**	0.10	0.06	0.08	0.07
**ee1.b6**	0.96	0.08	0.91	0.09
**ee2.a**	4.16	0.27	4.16	0.27
**ee2.b1**	-1.84	0.11	-1.84	0.11
**ee2.b2**	-1.30	0.08	-1.30	0.08
**ee2.b3**	-0.82	0.07	-0.82	0.07
**ee2.b4**	-0.44	0.06	-0.44	0.06
**ee2.b5**	-0.09	0.06	-0.09	0.06
**ee2.b6**	0.70	0.07	0.70	0.07

Brady et al. (2021)

**ee3.a**	3.28	0.20	3.28	0.20
**ee3.b1**	-1.39	0.09	-1.39	0.09
**ee3.b2**	-0.82	0.07	-0.82	0.07
**ee3.b3**	-0.41	0.06	-0.41	0.06
**ee3.b4**	0.01	0.06	0.01	0.06
**ee3.b5**	0.35	0.06	0.35	0.06
**ee3.b6**	1.14	0.08	1.14	0.08
**ee5.a**	4.88	0.32	4.88	0.32
**ee5.b1**	-1.28	0.08	-1.28	0.08
**ee5.b2**	-0.56	0.06	-0.56	0.06
**ee5.b3**	-0.22	0.06	-0.22	0.06
**ee5.b4**	0.11	0.06	0.11	0.06
**ee5.b5**	0.42	0.06	0.42	0.06
**ee5.b6**	1.00	0.07	1.00	0.07
**ee6.a**	2.55	0.20	3.14	0.27
**ee6.b1**	-2.11	0.16	-1.82	0.14
**ee6.b2**	-1.30	0.10	-1.07	0.09
**ee6.b3**	-0.90	0.09	-0.63	0.08
**ee6.b4**	-0.34	0.07	-0.10	0.07
**ee6.b5**	-0.01	0.07	0.21	0.08
**ee6.b6**	0.68	0.08	0.81	0.10
**ee7.a**	2.26	0.18	2.89	0.25
**ee7.b1**	-1.71	0.13	-1.50	0.12
**ee7.b2**	-1.20	0.10	-0.98	0.09
**ee7.b3**	-0.79	0.09	-0.59	0.08
**ee7.b4**	-0.35	0.08	-0.13	0.07
**ee7.b5**	-0.08	0.07	0.10	0.08
**ee7.b6**	0.70	0.09	0.75	0.10
**ee4ee8.a**	1.68	0.11	1.68	0.11
**ee4ee8.b1**	-1.15	0.10	-1.15	0.10
**ee4ee8.b2**	-0.70	0.08	-0.70	0.08
**ee4ee8.b3**	-0.18	0.07	-0.18	0.07
**ee4ee8.b4**	0.19	0.07	0.19	0.07
**ee4ee8.b5**	0.48	0.08	0.48	0.08
**ee4ee8.b6**	0.79	0.09	0.79	0.09
**ee4ee8.b7**	1.18	0.10	1.18	0.10
**ee4ee8.b8**	1.40	0.11	1.40	0.11

Brady et al. (2021)

**ee4ee8.b9**	1.67	0.13	1.67	0.13
**ee4ee8.b10**	1.95	0.15	1.95	0.15
**ee4ee8.b11**	2.36	0.18	2.36	0.18
**ee4ee8.b12**	3.01	0.24	3.01	0.24
**ee9.a**	2.63	0.21	2.46	0.23
**ee9.b1**	-0.48	0.08	-0.55	0.08
**ee9.b2**	0.12	0.07	0.23	0.08
**ee9.b3**	0.45	0.07	0.59	0.09
**ee9.b4**	0.78	0.08	0.91	0.11
**ee9.b5**	1.10	0.09	1.14	0.12
**ee9.b6**	1.71	0.12	1.65	0.17
**Latent Mean**	0.00	NA	-0.24	0.07
**Latent Variance**	1.00	NA	0.84	0.10

Figure 3.10 Differential item and test functioning by specialty group (General Internal Medicine and General Surgery) – EE subscale

a) Expected total score functions for reference (General Internal Medicine) and focal (General Surgery) groups

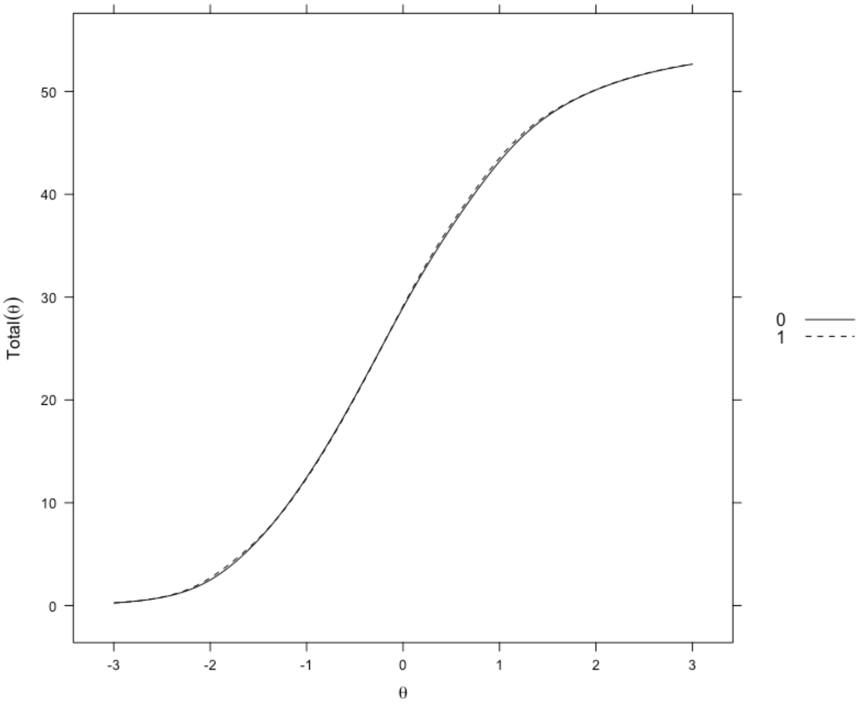

b) Expected item score functions for reference (General Internal Medicine) and focal (General Surgery) groups

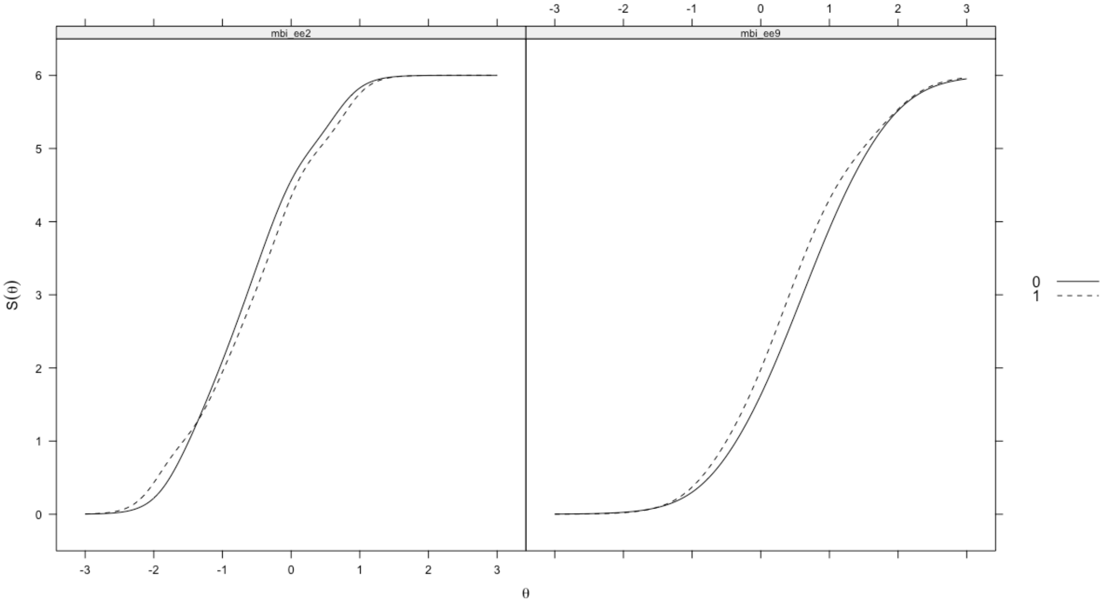

Table 3.10 Multi-group IRT item parameter estimates and standard errors (SE) by specialty group (reference: General Internal Medicine; focal: General Surgery) – EE subscale

	**Reference group item parameter estimates**	**Reference group SE**	**Focal group item parameter estimates**	**Focal group SE**
**ee1.a**	4.76	0.33	4.76	0.33
**ee1.b1**	-1.84	0.11	-1.84	0.11
**ee1.b2**	-1.08	0.07	-1.08	0.07
**ee1.b3**	-0.69	0.06	-0.69	0.06
**ee1.b4**	-0.23	0.06	-0.23	0.06
**ee1.b5**	0.12	0.06	0.12	0.06
**ee1.b6**	0.96	0.07	0.96	0.07
**ee2.a**	4.65	0.38	5.23	0.59
**ee2.b1**	-1.68	0.11	-1.94	0.15
**ee2.b2**	-1.24	0.09	-1.18	0.09
**ee2.b3**	-0.81	0.07	-0.74	0.08
**ee2.b4**	-0.46	0.06	-0.31	0.07
**ee2.b5**	-0.13	0.06	-0.01	0.07
**ee2.b6**	0.65	0.07	0.78	0.09
**ee3.a**	3.09	0.20	3.09	0.20
**ee3.b1**	-1.49	0.10	-1.49	0.10

**ee3.b2**	-0.90	0.07	-0.90	0.07
**ee3.b3**	-0.48	0.06	-0.48	0.06
**ee3.b4**	0.03	0.06	0.03	0.06
**ee3.b5**	0.38	0.07	0.38	0.07
**ee3.b6**	1.26	0.09	1.26	0.09
**ee5.a**	4.50	0.30	4.50	0.30
**ee5.b1**	-1.25	0.08	-1.25	0.08
**ee5.b2**	-0.59	0.06	-0.59	0.06
**ee5.b3**	-0.26	0.06	-0.26	0.06
**ee5.b4**	0.08	0.06	0.08	0.06
**ee5.b5**	0.43	0.06	0.43	0.06
**ee5.b6**	0.98	0.07	0.98	0.07
**ee6.a**	2.69	0.18	2.69	0.18
**ee6.b1**	-2.06	0.13	-2.06	0.13
**ee6.b2**	-1.31	0.09	-1.31	0.09
**ee6.b3**	-0.90	0.08	-0.90	0.08
**ee6.b4**	-0.34	0.06	-0.34	0.06
**ee6.b5**	0.00	0.06	0.00	0.06
**ee6.b6**	0.68	0.07	0.68	0.07
**ee7.a**	2.54	0.17	2.54	0.17
**ee7.b1**	-1.66	0.11	-1.66	0.11
**ee7.b2**	-1.15	0.09	-1.15	0.09
**ee7.b3**	-0.75	0.07	-0.75	0.07
**ee7.b4**	-0.34	0.07	-0.34	0.07
**ee7.b5**	-0.05	0.06	-0.05	0.06
**ee7.b6**	0.69	0.08	0.69	0.08
**ee4ee8.a**	1.64	0.12	1.64	0.12
**ee4ee8.b1**	-1.24	0.10	-1.24	0.10
**ee4ee8.b2**	-0.72	0.08	-0.72	0.08
**ee4ee8.b3**	-0.22	0.07	-0.22	0.07
**ee4ee8.b4**	0.13	0.08	0.13	0.08
**ee4ee8.b5**	0.44	0.08	0.44	0.08
**ee4ee8.b6**	0.74	0.09	0.74	0.09
**ee4ee8.b7**	1.16	0.11	1.16	0.11
**ee4ee8.b8**	1.38	0.12	1.38	0.12
**ee4ee8.b9**	1.69	0.14	1.69	0.14
**ee4ee8.b10**	2.02	0.16	2.02	0.16

Brady et al. (2021)

**ee4ee8.b11**	2.43	0.19	2.43	0.19
**ee4ee8.b12**	3.15	0.27	3.15	0.27
**ee9.a**	2.59	0.21	3.12	0.33
**ee9.b1**	-0.49	0.08	-0.71	0.09
**ee9.b2**	0.11	0.07	-0.06	0.08
**ee9.b3**	0.45	0.07	0.24	0.09
**ee9.b4**	0.78	0.08	0.56	0.10
**ee9.b5**	1.10	0.09	0.91	0.12
**ee9.b6**	1.72	0.12	1.89	0.22
**Latent Mean**	0.00	NA	-0.29	0.08
**Latent Variance**	1.00	NA	0.77	0.10

Figure 3.11 Differential item and test functioning by specialty group (General Internal Medicine and General Surgery Subspecialty) – EE subscale

a) Expected total score functions for reference (General Internal Medicine) and focal (General Surgery Subspecialty) groups

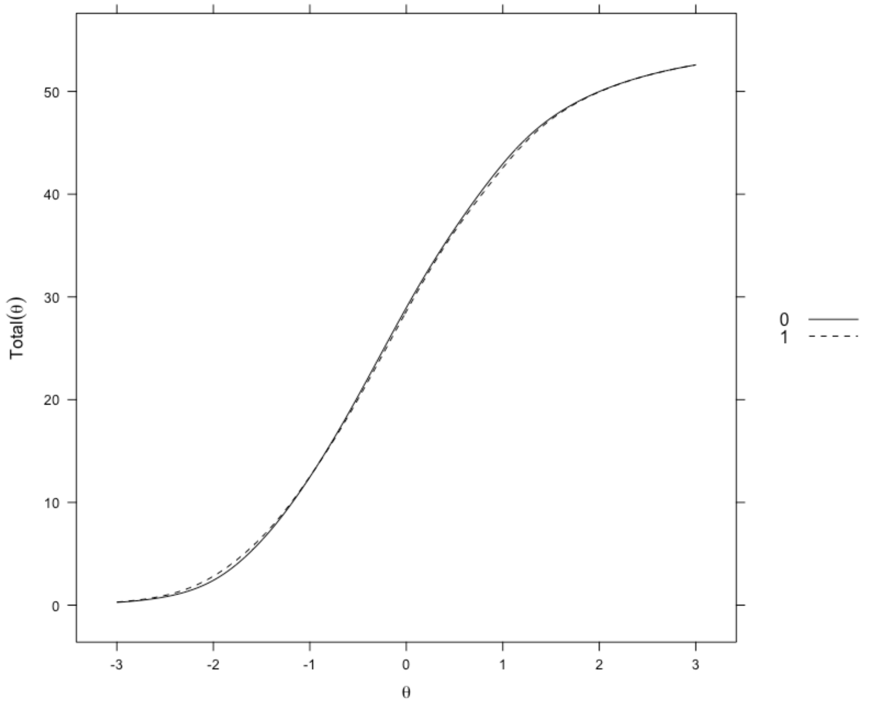

b) Expected item score functions for reference (General Internal Medicine) and focal (General Surgery Subspecialty) groups

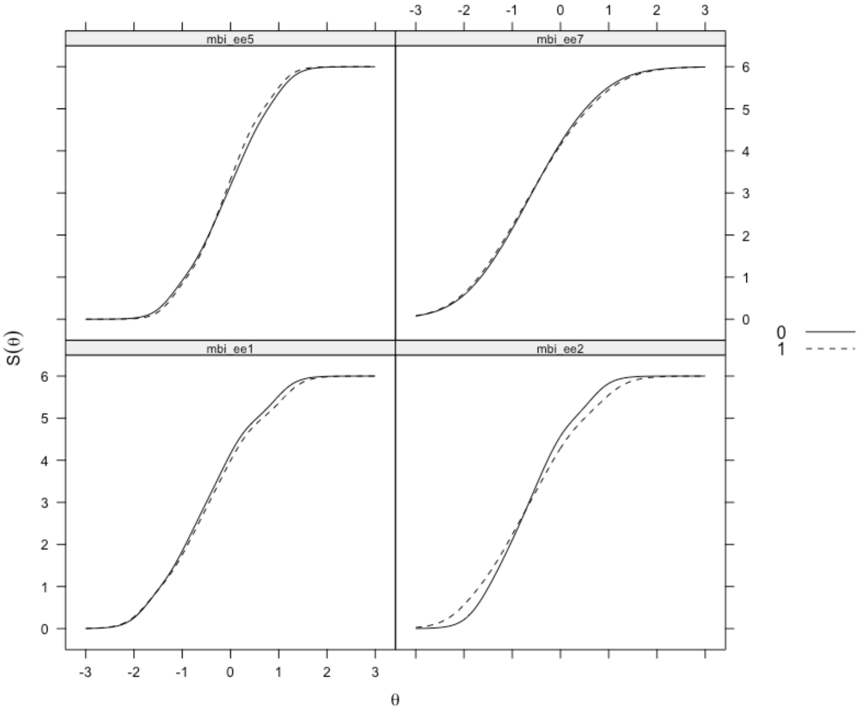

Table 3.11 Multi-group IRT item parameter estimates and standard errors (SE) by specialty group (reference: General Internal Medicine; focal: General Surgery Subspecialty) – EE subscale

	**Reference group item parameter estimates**	**Reference group SE**	**Focal group item parameter estimates**	**Focal group SE**
**ee1.a**	4.64	0.38	4.47	0.41
**ee1.b1**	-1.75	0.11	-1.77	0.13
**ee1.b2**	-1.10	0.08	-1.06	0.09
**ee1.b3**	-0.73	0.07	-0.67	0.08
**ee1.b4**	-0.27	0.06	-0.22	0.07
**ee1.b5**	0.08	0.06	0.17	0.07
**ee1.b6**	0.96	0.08	1.10	0.11
**ee2.a**	4.61	0.38	3.56	0.32
**ee2.b1**	-1.67	0.11	-1.97	0.15
**ee2.b2**	-1.24	0.09	-1.28	0.10
**ee2.b3**	-0.82	0.07	-0.89	0.09
**ee2.b4**	-0.47	0.06	-0.37	0.07
**ee2.b5**	-0.14	0.06	-0.01	0.07
**ee2.b6**	0.65	0.07	0.90	0.10

Brady et al. (2021)

**ee3.a**	3.21	0.20	3.21	0.20
**ee3.b1**	-1.45	0.09	-1.45	0.09
**ee3.b2**	-0.85	0.07	-0.85	0.07
**ee3.b3**	-0.44	0.06	-0.44	0.06
**ee3.b4**	0.01	0.06	0.01	0.06
**ee3.b5**	0.32	0.06	0.32	0.06
**ee3.b6**	1.17	0.08	1.17	0.08
**ee5.a**	4.73	0.38	5.29	0.52
**ee5.b1**	-1.24	0.09	-1.19	0.09
**ee5.b2**	-0.60	0.07	-0.56	0.07
**ee5.b3**	-0.25	0.06	-0.27	0.07
**ee5.b4**	0.12	0.06	0.07	0.07
**ee5.b5**	0.46	0.07	0.34	0.07
**ee5.b6**	1.03	0.08	0.97	0.10
**ee6.a**	2.62	0.17	2.62	0.17
**ee6.b1**	-2.00	0.12	-2.00	0.12
**ee6.b2**	-1.25	0.09	-1.25	0.09
**ee6.b3**	-0.87	0.07	-0.87	0.07
**ee6.b4**	-0.41	0.06	-0.41	0.06
**ee6.b5**	0.01	0.06	0.01	0.06
**ee6.b6**	0.74	0.07	0.74	0.07
**ee7.a**	2.27	0.18	2.31	0.21
**ee7.b1**	-1.70	0.13	-1.75	0.14
**ee7.b2**	-1.20	0.10	-1.28	0.11
**ee7.b3**	-0.79	0.09	-0.87	0.09
**ee7.b4**	-0.36	0.08	-0.35	0.08
**ee7.b5**	-0.08	0.07	-0.01	0.08
**ee7.b6**	0.69	0.09	0.82	0.11
**ee4ee8.a**	1.62	0.11	1.62	0.11
**ee4ee8.b1**	-1.21	0.10	-1.21	0.10
**ee4ee8.b2**	-0.68	0.08	-0.68	0.08
**ee4ee8.b3**	-0.15	0.07	-0.15	0.07
**ee4ee8.b4**	0.23	0.07	0.23	0.07
**ee4ee8.b5**	0.53	0.08	0.53	0.08
**ee4ee8.b6**	0.84	0.09	0.84	0.09
**ee4ee8.b7**	1.23	0.11	1.23	0.11
**ee4ee8.b8**	1.46	0.12	1.46	0.12

Brady et al. (2021)

**ee4ee8.b9**	1.73	0.13	1.73	0.13
**ee4ee8.b10**	2.06	0.16	2.06	0.16
**ee4ee8.b11**	2.51	0.19	2.51	0.19
**ee4ee8.b12**	3.14	0.26	3.14	0.26
**ee9.a**	2.61	0.17	2.61	0.17
**ee9.b1**	-0.56	0.07	-0.56	0.07
**ee9.b2**	0.06	0.06	0.06	0.06
**ee9.b3**	0.41	0.07	0.41	0.07
**ee9.b4**	0.78	0.07	0.78	0.07
**ee9.b5**	1.15	0.09	1.15	0.09
**ee9.b6**	1.74	0.11	1.74	0.11
**Latent Mean**	0.00	NA	-0.27	0.08
**Latent Variance**	1.00	NA	0.91	0.11

Figure 3.12 Differential item and test functioning by specialty group (General Internal Medicine and Internal Medicine Subspecialty) – EE subscale

a) Expected total score functions for reference (General Internal Medicine) and focal (Internal Medicine Subspecialty) groups

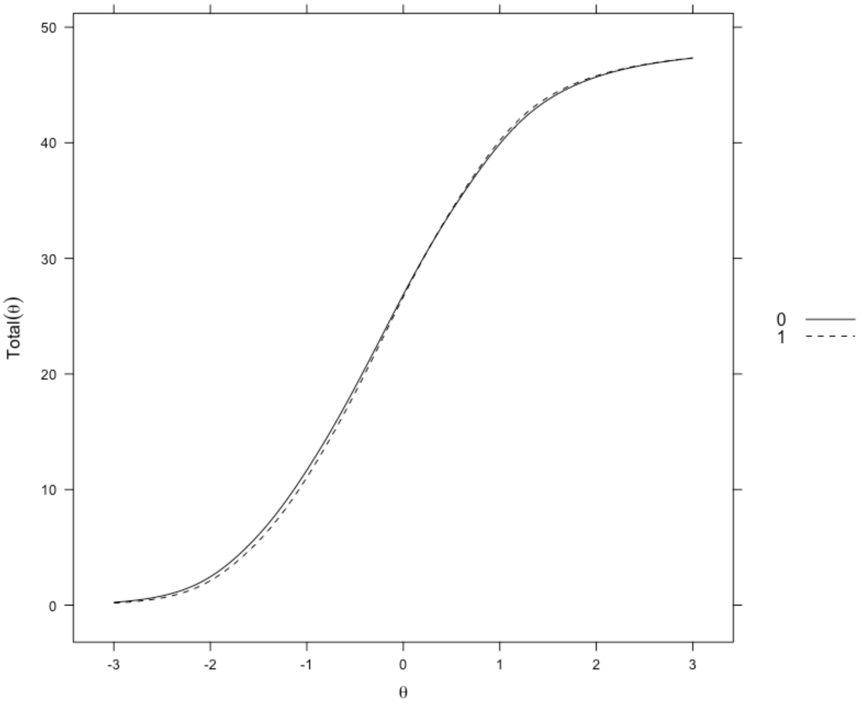

b) Expected item score functions for reference (General Internal Medicine) and focal (Internal Medicine Subspecialty) groups

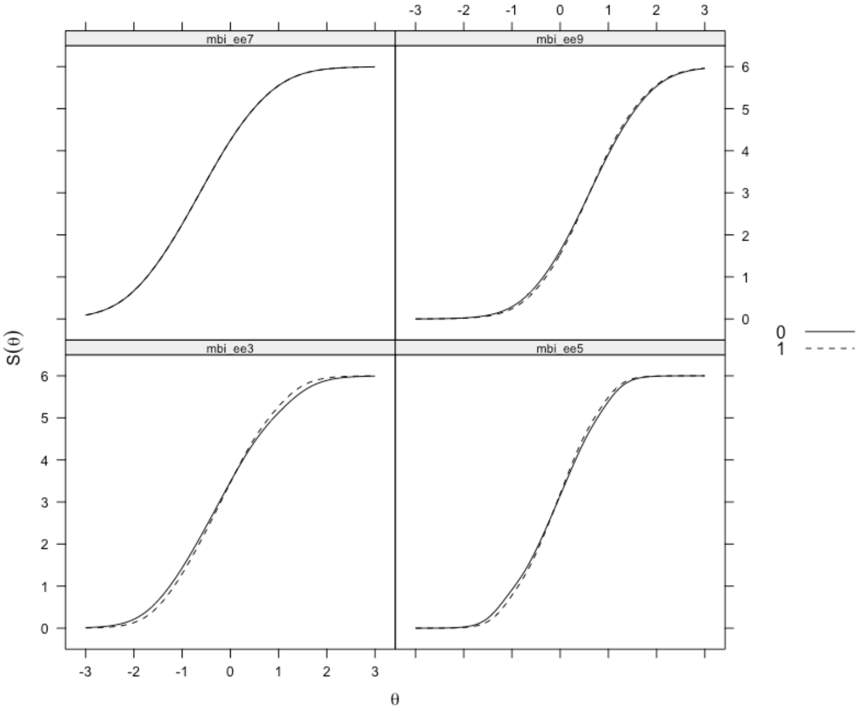

Table 3.12 Multi-group IRT item parameter estimates and standard errors (SE) by specialty group (reference: General Internal Medicine; focal: Internal Medicine Subspecialty) – EE subscale

	**Reference group item parameter estimates**	**Reference group SE**	**Focal group item parameter estimates**	**Focal group SE**
**ee1.a**	4.54	0.27	4.54	0.27
**ee1.b1**	-1.80	0.10	-1.80	0.10
**ee1.b2**	-1.07	0.07	-1.07	0.07
**ee1.b3**	-0.69	0.06	-0.69	0.06
**ee1.b4**	-0.21	0.06	-0.21	0.06
**ee1.b5**	0.08	0.06	0.08	0.06
**ee1.b6**	0.90	0.07	0.90	0.07
**ee2.a**	4.25	0.25	4.25	0.25
**ee2.b1**	-1.76	0.10	-1.76	0.10
**ee2.b2**	-1.25	0.08	-1.25	0.08
**ee2.b3**	-0.84	0.07	-0.84	0.07
**ee2.b4**	-0.42	0.06	-0.42	0.06
**ee2.b5**	-0.11	0.06	-0.11	0.06

**ee2.b6**	0.68	0.06	0.68	0.06
**ee3.a**	2.94	0.22	3.33	0.23
**ee3.b1**	-1.44	0.11	-1.36	0.09
**ee3.b2**	-0.87	0.08	-0.83	0.07
**ee3.b3**	-0.47	0.07	-0.41	0.06
**ee3.b4**	0.02	0.07	0.03	0.06
**ee3.b5**	0.36	0.07	0.35	0.07
**ee3.b6**	1.25	0.10	1.09	0.09
**ee5.a**	4.78	0.38	4.81	0.35
**ee5.b1**	-1.24	0.09	-1.13	0.08
**ee5.b2**	-0.59	0.07	-0.54	0.06
**ee5.b3**	-0.24	0.06	-0.23	0.06
**ee5.b4**	0.13	0.06	0.11	0.06
**ee5.b5**	0.46	0.06	0.39	0.06
**ee5.b6**	1.02	0.08	0.95	0.08
**ee6.a**	2.57	0.20	2.82	0.19
**ee6.b1**	-2.10	0.16	-1.89	0.13
**ee6.b2**	-1.30	0.10	-1.11	0.08
**ee6.b3**	-0.90	0.09	-0.75	0.07
**ee6.b4**	-0.34	0.07	-0.27	0.06
**ee6.b5**	-0.01	0.07	0.04	0.06
**ee6.b6**	0.67	0.08	0.77	0.08
**ee7.a**	2.32	0.14	2.32	0.14
**ee7.b1**	-1.86	0.11	-1.86	0.11
**ee7.b2**	-1.25	0.08	-1.25	0.08
**ee7.b3**	-0.83	0.07	-0.83	0.07
**ee7.b4**	-0.41	0.06	-0.41	0.06
**ee7.b5**	-0.08	0.06	-0.08	0.06
**ee7.b6**	0.67	0.07	0.67	0.07
**ee4ee8.a**	1.78	0.11	1.78	0.11
**ee4ee8.b1**	-0.68	0.07	-0.68	0.07
**ee4ee8.b2**	0.14	0.06	0.14	0.06
**ee4ee8.b3**	0.75	0.08	0.75	0.08
**ee4ee8.b4**	1.33	0.10	1.33	0.10
**ee4ee8.b5**	1.83	0.12	1.83	0.12
**ee4ee8.b6**	2.84	0.20	2.84	0.20
**ee9.a**	2.63	0.21	2.71	0.20

**ee9.b1**	-0.48	0.08	-0.42	0.07
**ee9.b2**	0.12	0.07	0.18	0.07
**ee9.b3**	0.45	0.07	0.48	0.07
**ee9.b4**	0.78	0.08	0.77	0.08
**ee9.b5**	1.10	0.09	0.98	0.09
**ee9.b6**	1.71	0.12	1.72	0.13
**Latent Mean**	0.00	NA	-0.13	0.06
**Latent Variance**	1.00	NA	0.71	0.07

Figure 3.13 Differential item and test functioning by specialty group (General Internal Medicine and Neurology) – EE subscale

a) Expected total score functions for reference (General Internal Medicine) and focal (Neurology) groups

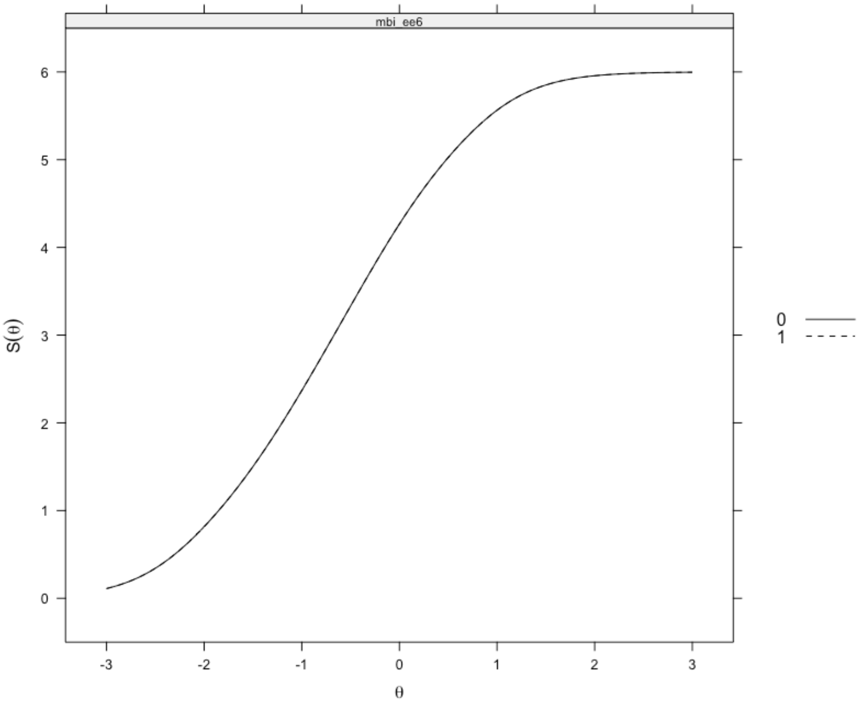

b) Expected item score functions for reference (General Internal Medicine) and focal (Neurology) groups

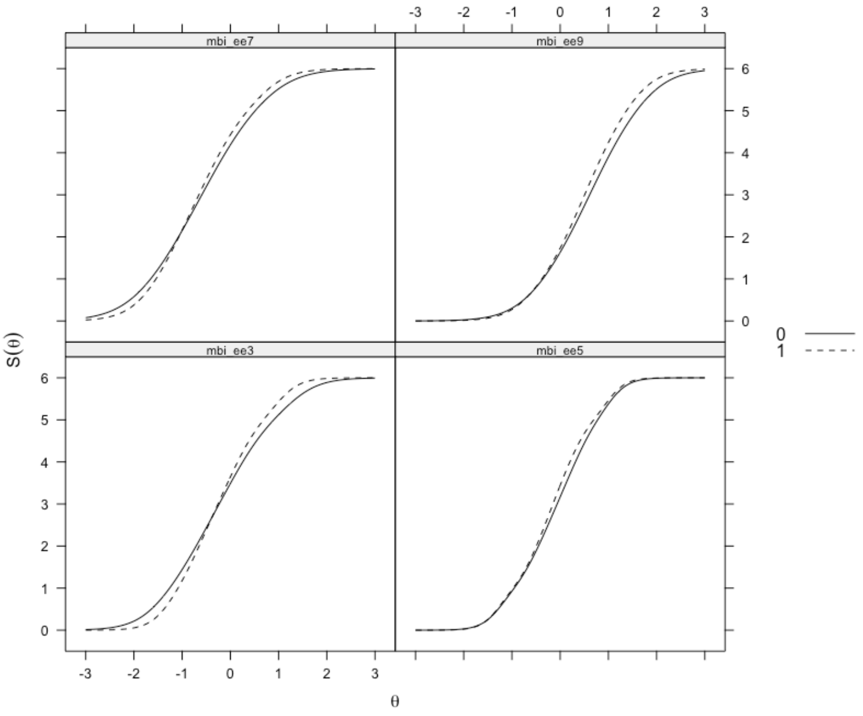

Table 3.13 Multi-group IRT item parameter estimates and standard errors (SE) by specialty group (reference: General Internal Medicine; focal: Neurology) – EE subscale

	**Reference group item parameter estimates**	**Reference group SE**	**Focal group item parameter estimates**	**Focal group SE**
**ee1.a**	4.92	0.35	4.92	0.35
**ee1.b1**	-1.81	0.11	-1.81	0.11
**ee1.b2**	-1.07	0.07	-1.07	0.07
**ee1.b3**	-0.71	0.06	-0.71	0.06
**ee1.b4**	-0.23	0.06	-0.23	0.06
**ee1.b5**	0.08	0.06	0.08	0.06
**ee1.b6**	0.95	0.07	0.95	0.07
**ee2.a**	4.79	0.34	4.79	0.34
**ee2.b1**	-1.69	0.10	-1.69	0.10
**ee2.b2**	-1.23	0.08	-1.23	0.08
**ee2.b3**	-0.82	0.07	-0.82	0.07
**ee2.b4**	-0.43	0.06	-0.43	0.06
**ee2.b5**	-0.12	0.06	-0.12	0.06
**ee2.b6**	0.65	0.07	0.65	0.07

**ee3.a**	2.92	0.22	4.15	0.43
**ee3.b1**	-1.44	0.11	-1.27	0.11
**ee3.b2**	-0.88	0.08	-0.79	0.09
**ee3.b3**	-0.47	0.07	-0.47	0.08
**ee3.b4**	0.02	0.07	-0.05	0.07
**ee3.b5**	0.35	0.07	0.28	0.08
**ee3.b6**	1.25	0.10	1.00	0.11
**ee5.a**	4.65	0.37	5.13	0.56
**ee5.b1**	-1.24	0.09	-1.28	0.11
**ee5.b2**	-0.60	0.07	-0.62	0.08
**ee5.b3**	-0.24	0.06	-0.30	0.07
**ee5.b4**	0.13	0.06	-0.01	0.07
**ee5.b5**	0.46	0.07	0.34	0.08
**ee5.b6**	1.02	0.08	1.00	0.10
**ee6.a**	2.62	0.17	2.62	0.17
**ee6.b1**	-2.13	0.14	-2.13	0.14
**ee6.b2**	-1.36	0.09	-1.36	0.09
**ee6.b3**	-0.91	0.08	-0.91	0.08
**ee6.b4**	-0.39	0.07	-0.39	0.07
**ee6.b5**	-0.03	0.06	-0.03	0.06
**ee6.b6**	0.75	0.08	0.75	0.08
**ee7.a**	2.27	0.18	2.96	0.31
**ee7.b1**	-1.71	0.13	-1.62	0.15
**ee7.b2**	-1.20	0.10	-1.20	0.12
**ee7.b3**	-0.79	0.09	-0.87	0.10
**ee7.b4**	-0.36	0.08	-0.47	0.09
**ee7.b5**	-0.08	0.07	-0.14	0.08
**ee7.b6**	0.69	0.09	0.64	0.10
**ee4ee8.a**	1.70	0.13	1.70	0.13
**ee4ee8.b1**	-0.76	0.09	-0.76	0.09
**ee4ee8.b2**	0.11	0.07	0.11	0.07
**ee4ee8.b3**	0.67	0.09	0.67	0.09
**ee4ee8.b4**	1.26	0.11	1.26	0.11
**ee4ee8.b5**	1.84	0.14	1.84	0.14
**ee4ee8.b6**	2.84	0.23	2.84	0.23
**ee9.a**	2.58	0.20	3.06	0.33
**ee9.b1**	-0.49	0.08	-0.57	0.09

**ee9.b2**	0.11	0.07	0.07	0.08
**ee9.b3**	0.45	0.07	0.36	0.09
**ee9.b4**	0.78	0.08	0.63	0.10
**ee9.b5**	1.10	0.09	1.00	0.12
**ee9.b6**	1.72	0.12	1.51	0.16
**Latent Mean**	0.00	NA	-0.09	0.08
**Latent Variance**	1.00	NA	0.81	0.11

Figure 3.14 Differential item and test functioning by specialty group (General Internal Medicine and Obstetrics and Gynecology) – EE subscale

a) Expected total score functions for reference (General Internal Medicine) and focal (Obstetrics and Gynecology) groups

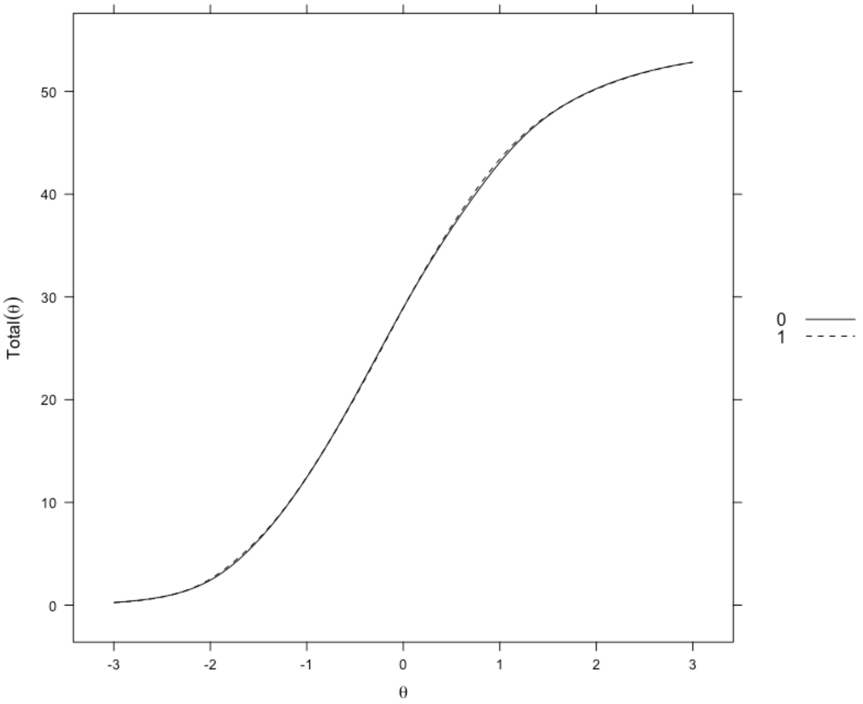

b) Expected item score functions for reference (General Internal Medicine) and focal (Obstetrics and Gynecology) groups

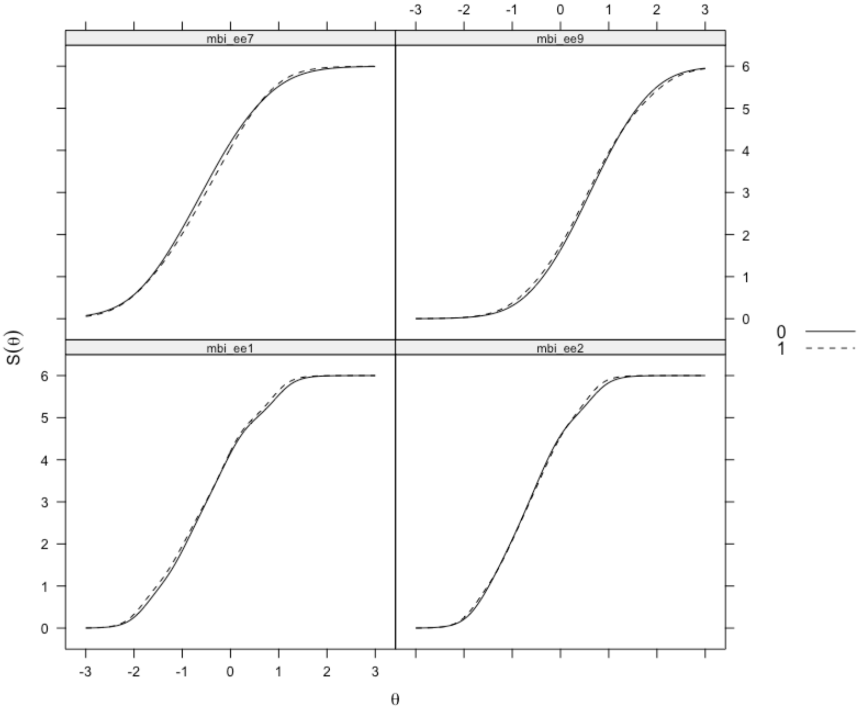

Table 3.14 Multi-group IRT item parameter estimates and standard errors (SE) by specialty group (reference: General Internal Medicine; focal: Obstetrics and Gynecology) – EE subscale

	**Reference group item parameter estimates**	**Reference group SE**	**Focal group item parameter estimates**	**Focal group SE**
**ee1.a**	4.72	0.39	5.11	0.56
**ee1.b1**	-1.75	0.11	-1.84	0.14
**ee1.b2**	-1.10	0.08	-1.18	0.10
**ee1.b3**	-0.73	0.07	-0.76	0.08
**ee1.b4**	-0.27	0.06	-0.24	0.07
**ee1.b5**	0.08	0.06	0.05	0.07
**ee1.b6**	0.96	0.08	0.87	0.10
**ee2.a**	4.69	0.39	5.31	0.59
**ee2.b1**	-1.67	0.11	-1.78	0.13
**ee2.b2**	-1.24	0.09	-1.20	0.10
**ee2.b3**	-0.82	0.07	-0.84	0.08
**ee2.b4**	-0.47	0.06	-0.43	0.07
**ee2.b5**	-0.14	0.06	-0.08	0.07
**ee2.b6**	0.65	0.07	0.57	0.08

Brady et al. (2021)

**ee3.a**	3.17	0.20	3.17	0.20
**ee3.b1**	-1.45	0.09	-1.45	0.09
**ee3.b2**	-0.84	0.07	-0.84	0.07
**ee3.b3**	-0.48	0.06	-0.48	0.06
**ee3.b4**	0.02	0.06	0.02	0.06
**ee3.b5**	0.39	0.06	0.39	0.06
**ee3.b6**	1.21	0.09	1.21	0.09
**ee5.a**	4.02	0.26	4.02	0.26
**ee5.b1**	-1.28	0.08	-1.28	0.08
**ee5.b2**	-0.64	0.06	-0.64	0.06
**ee5.b3**	-0.28	0.06	-0.28	0.06
**ee5.b4**	0.10	0.06	0.10	0.06
**ee5.b5**	0.43	0.06	0.43	0.06
**ee5.b6**	1.02	0.08	1.02	0.08
**ee6.a**	2.70	0.17	2.70	0.17
**ee6.b1**	-2.09	0.13	-2.09	0.13
**ee6.b2**	-1.25	0.09	-1.25	0.09
**ee6.b3**	-0.79	0.07	-0.79	0.07
**ee6.b4**	-0.32	0.06	-0.32	0.06
**ee6.b5**	0.03	0.06	0.03	0.06
**ee6.b6**	0.71	0.07	0.71	0.07
**ee7.a**	2.29	0.18	2.75	0.27
**ee7.b1**	-1.70	0.13	-1.90	0.16
**ee7.b2**	-1.20	0.10	-1.14	0.11
**ee7.b3**	-0.79	0.09	-0.76	0.09
**ee7.b4**	-0.36	0.08	-0.22	0.08
**ee7.b5**	-0.08	0.07	0.12	0.08
**ee7.b6**	0.69	0.09	0.66	0.10
**ee4ee8.a**	1.69	0.12	1.69	0.12
**ee4ee8.b1**	-1.18	0.10	-1.18	0.10
**ee4ee8.b2**	-0.70	0.08	-0.70	0.08
**ee4ee8.b3**	-0.22	0.07	-0.22	0.07
**ee4ee8.b4**	0.18	0.07	0.18	0.07
**ee4ee8.b5**	0.48	0.08	0.48	0.08
**ee4ee8.b6**	0.79	0.09	0.79	0.09
**ee4ee8.b7**	1.19	0.10	1.19	0.10
**ee4ee8.b8**	1.39	0.11	1.39	0.11

Brady et al. (2021)

**ee4ee8.b9**	1.66	0.13	1.66	0.13
**ee4ee8.b10**	1.94	0.14	1.94	0.14
**ee4ee8.b11**	2.36	0.18	2.36	0.18
**ee4ee8.b12**	2.87	0.22	2.87	0.22
**ee9.a**	2.57	0.20	2.73	0.27
**ee9.b1**	-0.50	0.08	-0.67	0.09
**ee9.b2**	0.11	0.07	0.08	0.08
**ee9.b3**	0.45	0.07	0.41	0.09
**ee9.b4**	0.78	0.08	0.69	0.10
**ee9.b5**	1.11	0.09	1.10	0.13
**ee9.b6**	1.72	0.12	1.93	0.20
**Latent Mean**	0.00	NA	-0.19	0.08
**Latent Variance**	1.00	NA	0.91	0.12

Figure 3.15 Differential item and test functioning by specialty group (General Internal Medicine and Ophthalmology) – EE subscale

a) Expected total score functions for reference (General Internal Medicine) and focal (Ophthalmology) groups

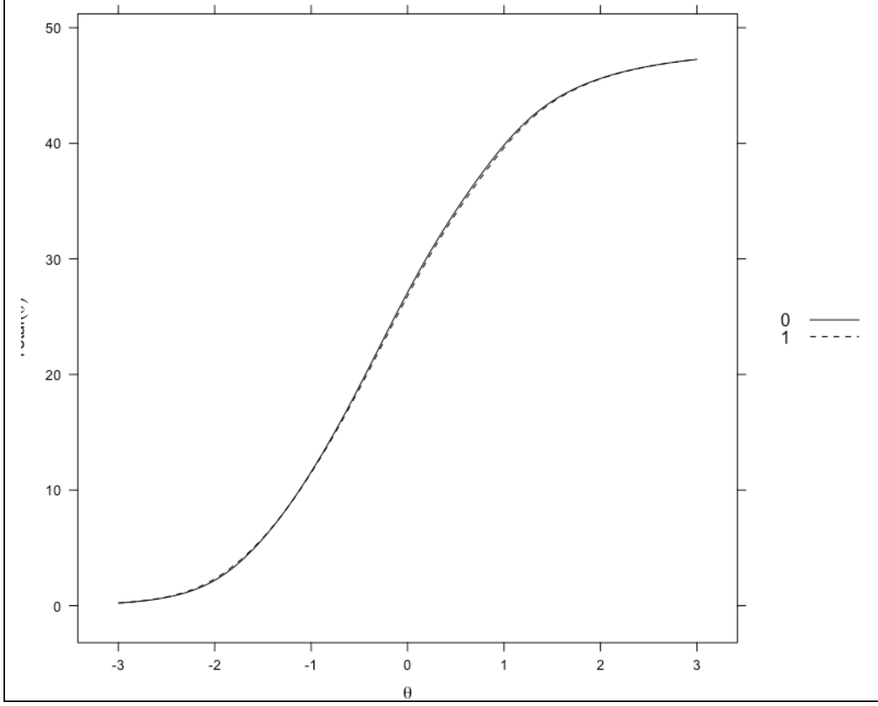

b) Expected item score functions for reference (General Internal Medicine) and focal (Ophthalmology) groups

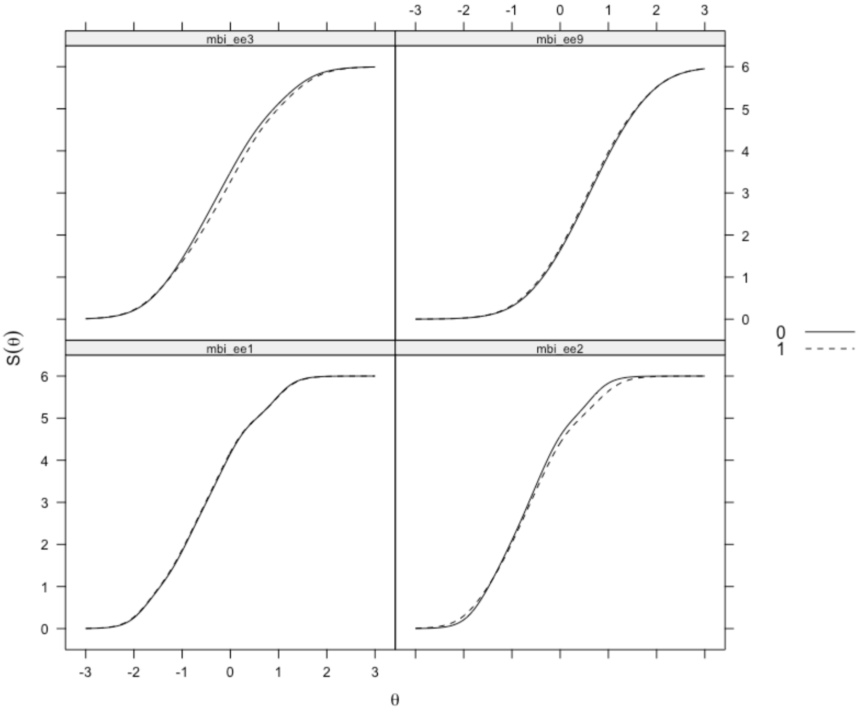

Table 3.15 Multi-group IRT item parameter estimates and standard errors (SE) by specialty group (reference: General Internal Medicine; focal: Ophthalmology) – EE subscale

	**Reference group item parameter estimates**	**Reference group SE**	**Focal group item parameter estimates**	**Focal group SE**
**ee1.a**	4.68	0.38	4.52	0.51
**ee1.b1**	-1.75	0.11	-1.76	0.14
**ee1.b2**	-1.10	0.08	-1.12	0.10
**ee1.b3**	-0.73	0.07	-0.74	0.08
**ee1.b4**	-0.27	0.06	-0.28	0.08
**ee1.b5**	0.08	0.06	0.05	0.08
**ee1.b6**	0.96	0.08	0.97	0.11
**ee2.a**	4.68	0.39	3.90	0.43
**ee2.b1**	-1.67	0.11	-1.72	0.14
**ee2.b2**	-1.24	0.09	-1.16	0.10
**ee2.b3**	-0.81	0.07	-0.80	0.09
**ee2.b4**	-0.47	0.06	-0.42	0.08
**ee2.b5**	-0.14	0.06	-0.09	0.08
**ee2.b6**	0.65	0.07	0.83	0.11

Brady et al. (2021)

**ee3.a**	2.93	0.22	3.00	0.31
**ee3.b1**	-1.44	0.11	-1.51	0.13
**ee3.b2**	-0.88	0.08	-0.84	0.10
**ee3.b3**	-0.48	0.07	-0.33	0.09
**ee3.b4**	0.02	0.07	0.15	0.09
**ee3.b5**	0.35	0.07	0.43	0.10
**ee3.b6**	1.25	0.10	1.34	0.16
**ee5.a**	4.58	0.32	4.58	0.32
**ee5.b1**	-1.25	0.08	-1.25	0.08
**ee5.b2**	-0.58	0.06	-0.58	0.06
**ee5.b3**	-0.26	0.06	-0.26	0.06
**ee5.b4**	0.10	0.06	0.10	0.06
**ee5.b5**	0.43	0.06	0.43	0.06
**ee5.b6**	1.04	0.08	1.04	0.08
**ee6.a**	2.58	0.17	2.58	0.17
**ee6.b1**	-2.14	0.14	-2.14	0.14
**ee6.b2**	-1.23	0.09	-1.23	0.09
**ee6.b3**	-0.88	0.08	-0.88	0.08
**ee6.b4**	-0.37	0.07	-0.37	0.07
**ee6.b5**	-0.01	0.06	-0.01	0.06
**ee6.b6**	0.70	0.08	0.70	0.08
**ee7.a**	2.42	0.16	2.42	0.16
**ee7.b1**	-1.69	0.11	-1.69	0.11
**ee7.b2**	-1.15	0.09	-1.15	0.09
**ee7.b3**	-0.74	0.07	-0.74	0.07
**ee7.b4**	-0.33	0.07	-0.33	0.07
**ee7.b5**	-0.07	0.07	-0.07	0.07
**ee7.b6**	0.73	0.08	0.73	0.08
**ee4ee8.a**	1.66	0.12	1.66	0.12
**ee4ee8.b1**	-0.77	0.09	-0.77	0.09
**ee4ee8.b2**	0.09	0.08	0.09	0.08
**ee4ee8.b3**	0.69	0.09	0.69	0.09
**ee4ee8.b4**	1.34	0.11	1.34	0.11
**ee4ee8.b5**	1.90	0.15	1.90	0.15
**ee4ee8.b6**	2.88	0.24	2.88	0.24
**ee9.a**	2.58	0.21	2.69	0.30
**ee9.b1**	-0.50	0.08	-0.58	0.09

**ee9.b2**	0.11	0.07	0.12	0.09
**ee9.b3**	0.44	0.07	0.36	0.10
**ee9.b4**	0.78	0.08	0.82	0.12
**ee9.b5**	1.10	0.09	1.00	0.13
**ee9.b6**	1.73	0.12	1.79	0.21
**Latent Mean**	0.00	NA	-0.27	0.09
**Latent Variance**	1.00	NA	0.96	0.14

Figure 3.16 Differential item and test functioning by specialty group (General Internal Medicine and Orthopedic Surgery) – EE subscale

a) Expected total score functions for reference (General Internal Medicine) and focal (Orthopedic Surgery) groups

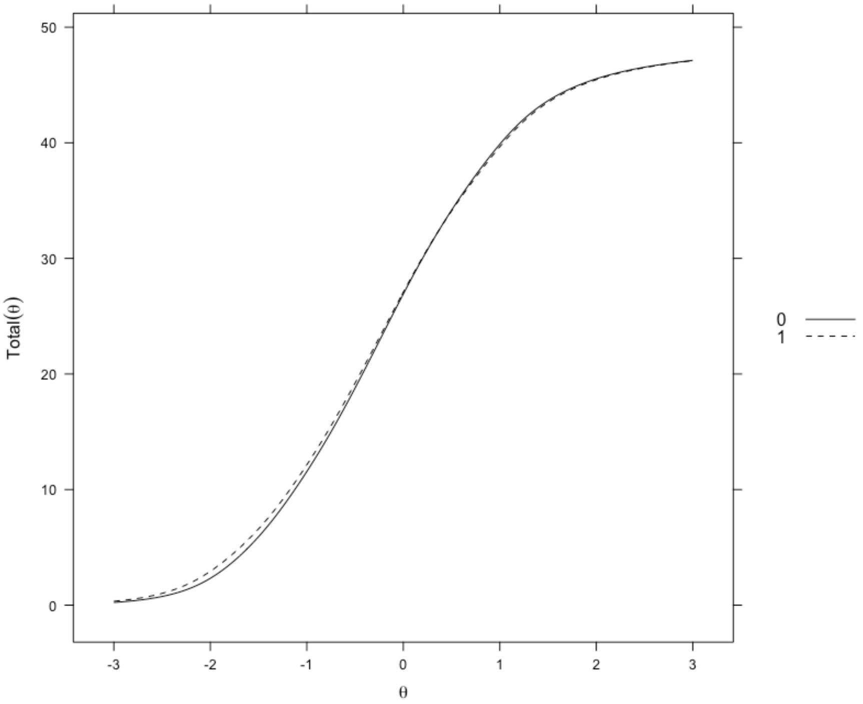

b) Expected item score functions for reference (General Internal Medicine) and focal (Orthopedic Surgery) groups

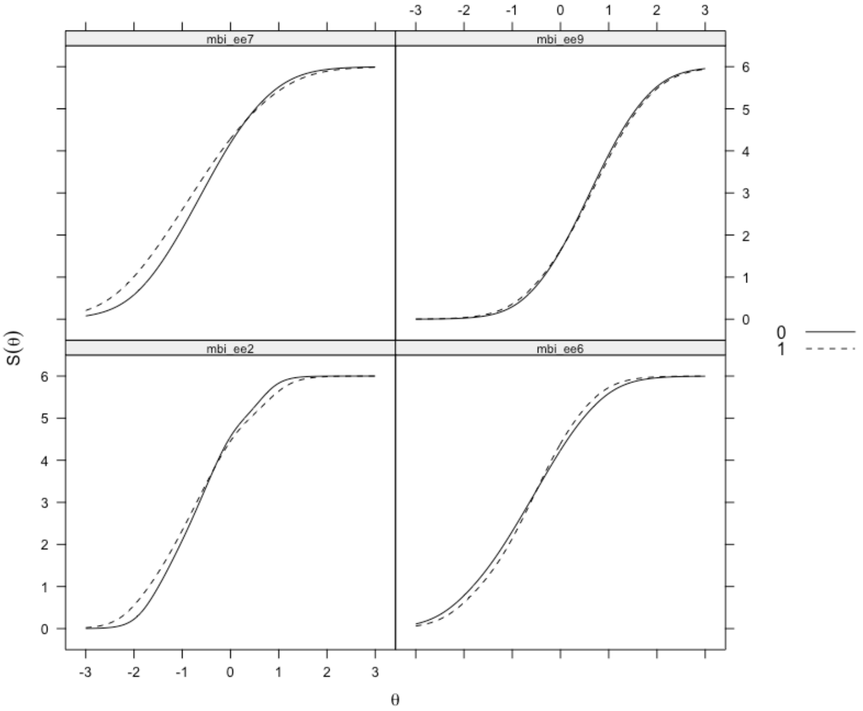

Table 3.16 Multi-group IRT item parameter estimates and standard errors (SE) by specialty group (reference: General Internal Medicine; focal: Orthopedic Surgery) – EE subscale

	**Reference group item parameter estimates**	**Reference group SE**	**Focal group item parameter estimates**	**Focal group SE**
**ee1.a**	4.57	0.32	4.57	0.32
**ee1.b1**	-1.87	0.11	-1.87	0.11
**ee1.b2**	-1.12	0.08	-1.12	0.08
**ee1.b3**	-0.70	0.07	-0.70	0.07
**ee1.b4**	-0.22	0.06	-0.22	0.06
**ee1.b5**	0.08	0.06	0.08	0.06
**ee1.b6**	0.96	0.07	0.96	0.07
**ee2.a**	4.64	0.39	3.69	0.40
**ee2.b1**	-1.69	0.11	-1.94	0.16
**ee2.b2**	-1.24	0.09	-1.34	0.11
**ee2.b3**	-0.81	0.07	-0.91	0.09
**ee2.b4**	-0.46	0.06	-0.48	0.08
**ee2.b5**	-0.13	0.06	-0.10	0.08
**ee2.b6**	0.65	0.07	0.82	0.10

Brady et al. (2021)

**ee3.a**	3.13	0.20	3.13	0.20
**ee3.b1**	-1.37	0.09	-1.37	0.09
**ee3.b2**	-0.80	0.07	-0.80	0.07
**ee3.b3**	-0.42	0.06	-0.42	0.06
**ee3.b4**	0.05	0.06	0.05	0.06
**ee3.b5**	0.37	0.06	0.37	0.06
**ee3.b6**	1.20	0.09	1.20	0.09
**ee5.a**	4.80	0.34	4.80	0.34
**ee5.b1**	-1.29	0.08	-1.29	0.08
**ee5.b2**	-0.59	0.06	-0.59	0.06
**ee5.b3**	-0.23	0.06	-0.23	0.06
**ee5.b4**	0.12	0.06	0.12	0.06
**ee5.b5**	0.39	0.06	0.39	0.06
**ee5.b6**	1.02	0.08	1.02	0.08
**ee6.a**	2.56	0.20	2.83	0.30
**ee6.b1**	-2.10	0.16	-1.99	0.18
**ee6.b2**	-1.30	0.10	-1.14	0.11
**ee6.b3**	-0.90	0.09	-0.79	0.10
**ee6.b4**	-0.35	0.07	-0.40	0.09
**ee6.b5**	-0.01	0.07	-0.13	0.08
**ee6.b6**	0.67	0.08	0.52	0.10
**ee7.a**	2.26	0.18	2.02	0.23
**ee7.b1**	-1.71	0.13	-2.06	0.21
**ee7.b2**	-1.20	0.10	-1.55	0.16
**ee7.b3**	-0.79	0.09	-1.04	0.13
**ee7.b4**	-0.36	0.08	-0.50	0.10
**ee7.b5**	-0.08	0.07	-0.12	0.10
**ee7.b6**	0.69	0.09	0.83	0.13
**ee4ee8.a**	1.66	0.12	1.66	0.12
**ee4ee8.b1**	-0.69	0.09	-0.69	0.09
**ee4ee8.b2**	0.12	0.08	0.12	0.08
**ee4ee8.b3**	0.69	0.09	0.69	0.09
**ee4ee8.b4**	1.34	0.12	1.34	0.12
**ee4ee8.b5**	2.00	0.16	2.00	0.16
**ee4ee8.b6**	3.15	0.27	3.15	0.27
**ee9.a**	2.61	0.21	2.42	0.27
**ee9.b1**	-0.49	0.08	-0.56	0.10

**ee9.b2**	0.11	0.07	0.11	0.09
**ee9.b3**	0.44	0.07	0.54	0.10
**ee9.b4**	0.77	0.08	0.82	0.12
**ee9.b5**	1.10	0.09	1.10	0.14
**ee9.b6**	1.71	0.12	1.70	0.19
**Latent Mean**	0.00	NA	-0.18	0.08
**Latent Variance**	1.00	NA	0.87	0.12

Figure 3.17 Differential item and test functioning by specialty group (General Internal Medicine and Pediatric Subspecialty) – EE subscale

a) Expected total score functions for reference (General Internal Medicine) and focal (Pediatric Subspecialty) groups

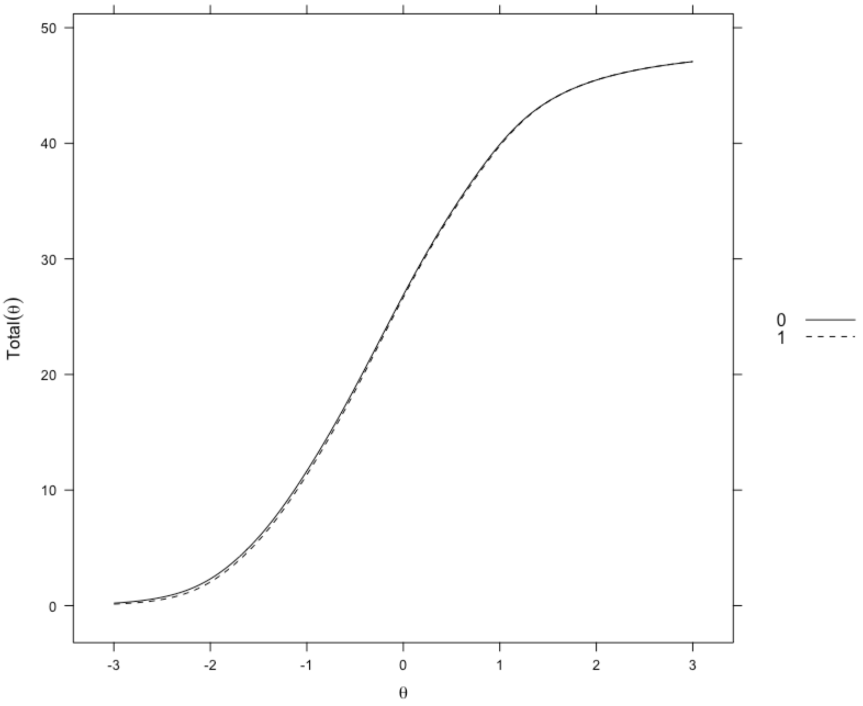

b) Expected item score functions for reference (General Internal Medicine) and focal (Pediatric Subspecialty) groups

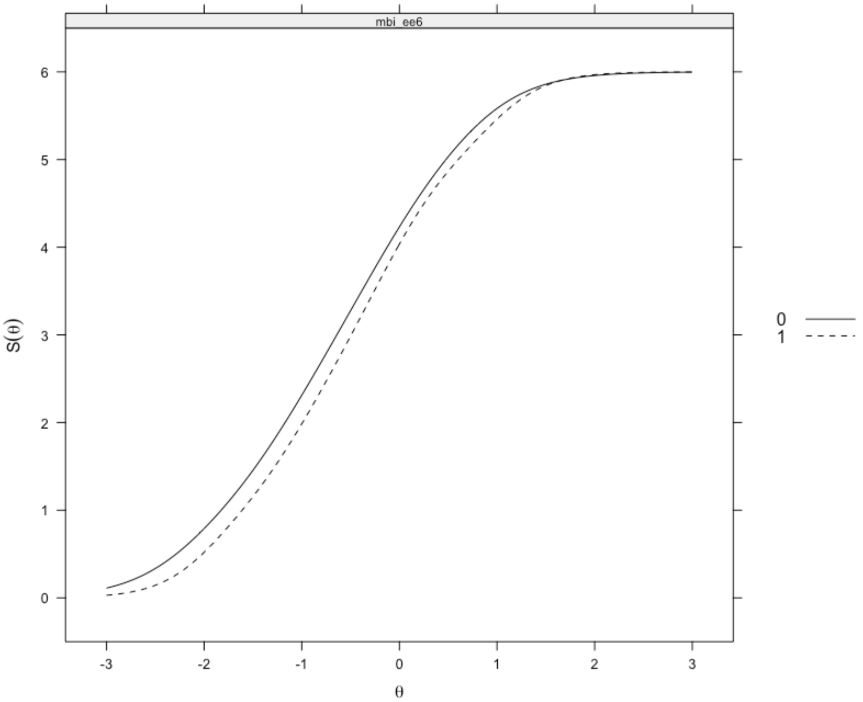

Table 3.17 Multi-group IRT item parameter estimates and standard errors (SE) by specialty group (reference: General Internal Medicine; focal: Pediatric Subspecialty) – EE subscale

	**Reference group item parameter estimates**	**Reference group SE**	**Focal group item parameter estimates**	**Focal group SE**
**ee1.a**	4.48	0.29	4.48	0.29
**ee1.b1**	-1.89	0.11	-1.89	0.11
**ee1.b2**	-1.11	0.07	-1.11	0.07
**ee1.b3**	-0.76	0.06	-0.76	0.06
**ee1.b4**	-0.26	0.06	-0.26	0.06
**ee1.b5**	0.13	0.06	0.13	0.06
**ee1.b6**	0.92	0.07	0.92	0.07
**ee2.a**	4.79	0.33	4.79	0.33
**ee2.b1**	-1.69	0.10	-1.69	0.10
**ee2.b2**	-1.23	0.08	-1.23	0.08
**ee2.b3**	-0.82	0.07	-0.82	0.07
**ee2.b4**	-0.42	0.06	-0.42	0.06
**ee2.b5**	-0.08	0.06	-0.08	0.06
**ee2.b6**	0.68	0.07	0.68	0.07

Brady et al. (2021)

**ee3.a**	3.32	0.20	3.32	0.20
**ee3.b1**	-1.39	0.09	-1.39	0.09
**ee3.b2**	-0.86	0.07	-0.86	0.07
**ee3.b3**	-0.46	0.06	-0.46	0.06
**ee3.b4**	0.03	0.06	0.03	0.06
**ee3.b5**	0.35	0.06	0.35	0.06
**ee3.b6**	1.18	0.08	1.18	0.08
**ee5.a**	4.91	0.33	4.91	0.33
**ee5.b1**	-1.26	0.08	-1.26	0.08
**ee5.b2**	-0.58	0.06	-0.58	0.06
**ee5.b3**	-0.25	0.06	-0.25	0.06
**ee5.b4**	0.13	0.06	0.13	0.06
**ee5.b5**	0.46	0.06	0.46	0.06
**ee5.b6**	0.98	0.07	0.98	0.07
**ee6.a**	2.56	0.20	3.36	0.30
**ee6.b1**	-2.11	0.16	-1.93	0.15
**ee6.b2**	-1.30	0.10	-1.19	0.10
**ee6.b3**	-0.90	0.09	-0.72	0.08
**ee6.b4**	-0.34	0.07	-0.23	0.07
**ee6.b5**	-0.01	0.07	0.10	0.07
**ee6.b6**	0.68	0.08	0.97	0.11
**ee7.a**	2.58	0.17	2.58	0.17
**ee7.b1**	-1.77	0.11	-1.77	0.11
**ee7.b2**	-1.25	0.09	-1.25	0.09
**ee7.b3**	-0.79	0.07	-0.79	0.07
**ee7.b4**	-0.33	0.06	-0.33	0.06
**ee7.b5**	-0.04	0.06	-0.04	0.06
**ee7.b6**	0.70	0.07	0.70	0.07
**ee4ee8.a**	1.59	0.12	1.59	0.12
**ee4ee8.b1**	-0.70	0.08	-0.70	0.08
**ee4ee8.b2**	0.17	0.07	0.17	0.07
**ee4ee8.b3**	0.73	0.09	0.73	0.09
**ee4ee8.b4**	1.46	0.12	1.46	0.12
**ee4ee8.b5**	2.08	0.16	2.08	0.16
**ee4ee8.b6**	3.17	0.27	3.17	0.27
**ee9.a**	2.55	0.17	2.55	0.17
**ee9.b1**	-0.52	0.07	-0.52	0.07

**ee9.b2**	0.12	0.06	0.12	0.06
**ee9.b3**	0.44	0.07	0.44	0.07
**ee9.b4**	0.78	0.08	0.78	0.08
**ee9.b5**	1.13	0.09	1.13	0.09
**ee9.b6**	1.70	0.11	1.70	0.11
**Latent Mean**	0.00	NA	-0.24	0.07
**Latent Variance**	1.00	NA	0.71	0.09

Figure 3.18 Differential item and test functioning by specialty group (General Internal Medicine and Physical Medicine and Rehabilitation/Preventive Medicine/Occupational Medicine) – EE subscale

a) Expected total score functions for reference (General Internal Medicine) and focal (Physical Medicine and Rehabilitation/Preventive Medicine/Occupational Medicine) groups

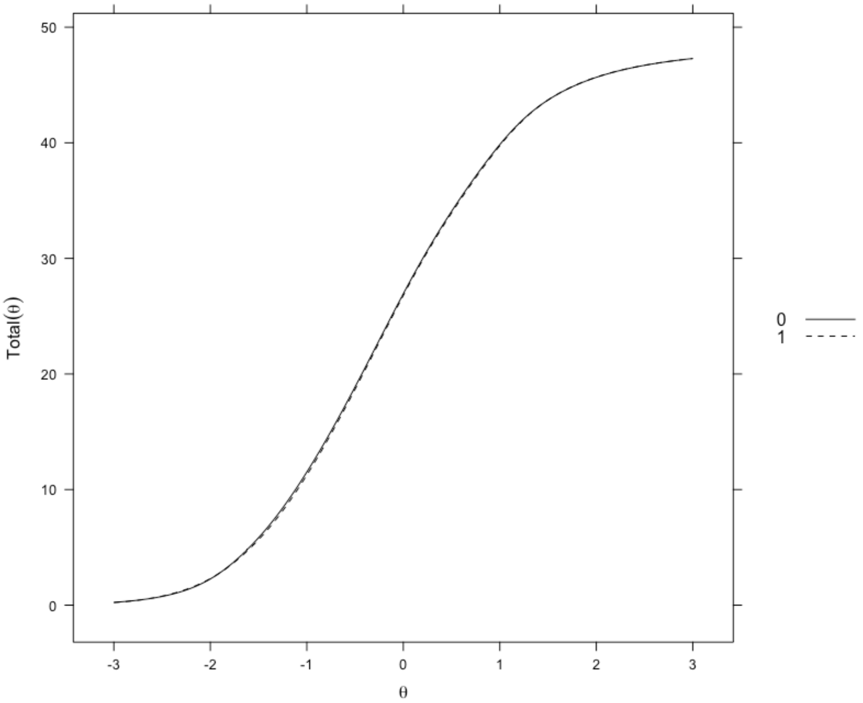

b) Expected item score functions for reference (General Internal Medicine) and focal (Physical Medicine and Rehabilitation/Preventive Medicine/Occupational Medicine) groups

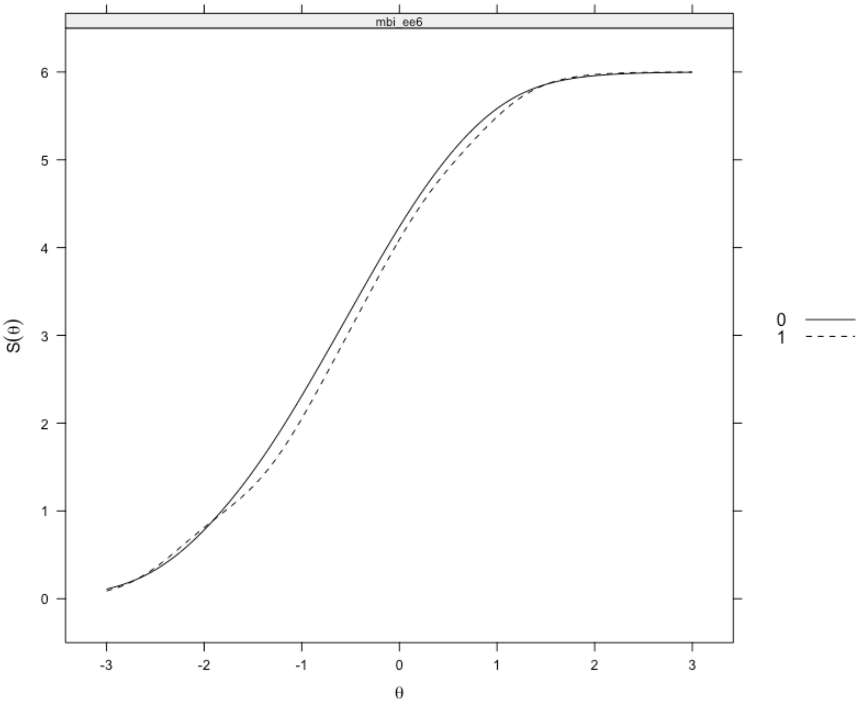

Table 3.18 Multi-group IRT item parameter estimates and standard errors (SE) by specialty group (reference: General Internal Medicine; focal: Physical Medicine and Rehabilitation/Preventive Medicine/Occupational Medicine) – EE subscale

	**Reference group item parameter estimates**	**Reference group SE**	**Focal group item parameter estimates**	**Focal group SE**
**ee1.a**	4.64	0.31	4.64	0.31
**ee1.b1**	-1.77	0.10	-1.77	0.10
**ee1.b2**	-1.06	0.07	-1.06	0.07
**ee1.b3**	-0.75	0.06	-0.75	0.06
**ee1.b4**	-0.28	0.06	-0.28	0.06
**ee1.b5**	0.07	0.06	0.07	0.06
**ee1.b6**	0.96	0.07	0.96	0.07
**ee2.a**	4.65	0.32	4.65	0.32
**ee2.b1**	-1.71	0.10	-1.71	0.10
**ee2.b2**	-1.19	0.08	-1.19	0.08
**ee2.b3**	-0.81	0.07	-0.81	0.07
**ee2.b4**	-0.46	0.06	-0.46	0.06

**ee2.b5**	-0.11	0.06	-0.11	0.06
**ee2.b6**	0.69	0.07	0.69	0.07
**ee3.a**	3.11	0.19	3.11	0.19
**ee3.b1**	-1.42	0.09	-1.42	0.09
**ee3.b2**	-0.86	0.07	-0.86	0.07
**ee3.b3**	-0.44	0.06	-0.44	0.06
**ee3.b4**	0.01	0.06	0.01	0.06
**ee3.b5**	0.35	0.06	0.35	0.06
**ee3.b6**	1.20	0.09	1.20	0.09
**ee5.a**	4.79	0.32	4.79	0.32
**ee5.b1**	-1.26	0.08	-1.26	0.08
**ee5.b2**	-0.61	0.06	-0.61	0.06
**ee5.b3**	-0.26	0.06	-0.26	0.06
**ee5.b4**	0.10	0.06	0.10	0.06
**ee5.b5**	0.42	0.06	0.42	0.06
**ee5.b6**	1.02	0.07	1.02	0.07
**ee6.a**	2.55	0.20	3.38	0.31
**ee6.b1**	-2.09	0.16	-2.30	0.18
**ee6.b2**	-1.30	0.10	-1.15	0.10
**ee6.b3**	-0.90	0.09	-0.79	0.08
**ee6.b4**	-0.35	0.07	-0.28	0.08
**ee6.b5**	-0.01	0.07	0.10	0.08
**ee6.b6**	0.68	0.08	0.94	0.11
**ee7.a**	2.19	0.14	2.19	0.14
**ee7.b1**	-1.75	0.11	-1.75	0.11
**ee7.b2**	-1.19	0.09	-1.19	0.09
**ee7.b3**	-0.74	0.07	-0.74	0.07
**ee7.b4**	-0.28	0.07	-0.28	0.07
**ee7.b5**	0.01	0.07	0.01	0.07
**ee7.b6**	0.82	0.08	0.82	0.08
**ee4ee8.a**	1.73	0.12	1.73	0.12
**ee4ee8.b1**	-0.75	0.08	-0.75	0.08
**ee4ee8.b2**	0.06	0.07	0.06	0.07
**ee4ee8.b3**	0.62	0.08	0.62	0.08
**ee4ee8.b4**	1.28	0.11	1.28	0.11
**ee4ee8.b5**	1.91	0.14	1.91	0.14
**ee4ee8.b6**	2.88	0.23	2.88	0.23

Brady et al. (2021)

**ee9.a**	2.73	0.18	2.73	0.18
**ee9.b1**	-0.52	0.07	-0.52	0.07
**ee9.b2**	0.13	0.06	0.13	0.06
**ee9.b3**	0.45	0.07	0.45	0.07
**ee9.b4**	0.79	0.07	0.79	0.07
**ee9.b5**	1.10	0.08	1.10	0.08
**ee9.b6**	1.74	0.11	1.74	0.11
**Latent Mean**	0.00	NA	-0.29	0.08
**Latent Variance**	1.00	NA	0.94	0.12

Figure 3.19 Differential item and test functioning by specialty group (General Internal Medicine and Psychiatry) – EE subscale

a) Expected total score functions for reference (General Internal Medicine) and focal (Psychiatry) groups

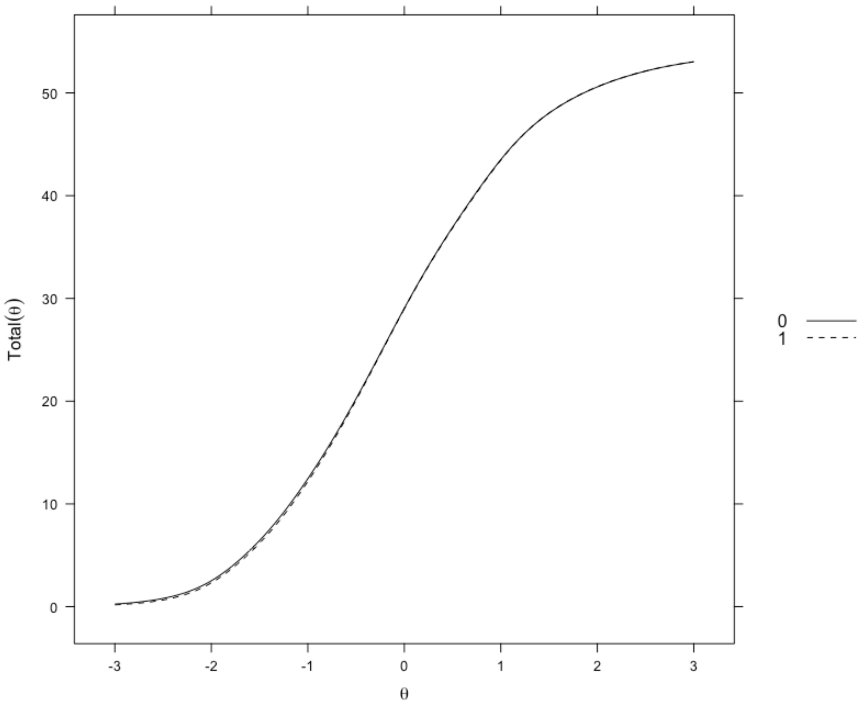

b) Expected item score functions for reference (General Internal Medicine) and focal (Psychiatry) groups

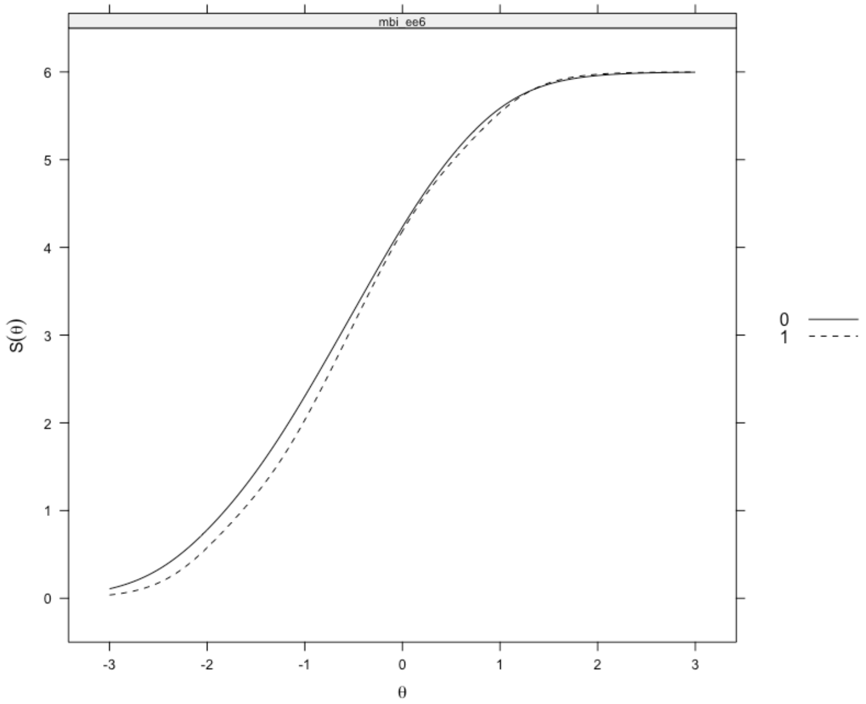

Table 3.19 Multi-group IRT item parameter estimates and standard errors (SE) by specialty group (reference: General Internal Medicine; focal: Psychiatry) – EE subscale

	Reference group item parameter estimates	Reference group SE	Focal group item parameter estimates	Focal group SE
**ee1.a**	4.90	0.30	4.90	0.30
**ee1.b1**	-1.82	0.10	-1.82	0.10
**ee1.b2**	-1.10	0.07	-1.10	0.07
**ee1.b3**	-0.70	0.06	-0.70	0.06
**ee1.b4**	-0.27	0.06	-0.27	0.06
**ee1.b5**	0.07	0.06	0.07	0.06
**ee1.b6**	0.92	0.07	0.92	0.07
**ee2.a**	4.53	0.28	4.53	0.28
**ee2.b1**	-1.78	0.10	-1.78	0.10
**ee2.b2**	-1.26	0.08	-1.26	0.08
**ee2.b3**	-0.81	0.06	-0.81	0.06
**ee2.b4**	-0.40	0.06	-0.40	0.06
**ee2.b5**	-0.10	0.06	-0.10	0.06
**ee2.b6**	0.71	0.06	0.71	0.06

**ee3.a**	3.07	0.17	3.07	0.17
**ee3.b1**	-1.42	0.09	-1.42	0.09
**ee3.b2**	-0.81	0.07	-0.81	0.07
**ee3.b3**	-0.42	0.06	-0.42	0.06
**ee3.b4**	0.05	0.06	0.05	0.06
**ee3.b5**	0.38	0.06	0.38	0.06
**ee3.b6**	1.18	0.08	1.18	0.08
**ee5.a**	4.55	0.27	4.55	0.27
**ee5.b1**	-1.27	0.08	-1.27	0.08
**ee5.b2**	-0.59	0.06	-0.59	0.06
**ee5.b3**	-0.25	0.06	-0.25	0.06
**ee5.b4**	0.11	0.06	0.11	0.06
**ee5.b5**	0.43	0.06	0.43	0.06
**ee5.b6**	0.99	0.07	0.99	0.07
**ee6.a**	2.57	0.20	3.32	0.24
**ee6.b1**	-2.10	0.15	-2.00	0.13
**ee6.b2**	-1.30	0.10	-1.15	0.08
**ee6.b3**	-0.90	0.09	-0.75	0.07
**ee6.b4**	-0.34	0.07	-0.35	0.06
**ee6.b5**	-0.01	0.07	0.04	0.06
**ee6.b6**	0.68	0.08	0.89	0.09
**ee7.a**	2.54	0.15	2.54	0.15
**ee7.b1**	-1.63	0.10	-1.63	0.10
**ee7.b2**	-1.13	0.08	-1.13	0.08
**ee7.b3**	-0.75	0.07	-0.75	0.07
**ee7.b4**	-0.34	0.06	-0.34	0.06
**ee7.b5**	-0.02	0.06	-0.02	0.06
**ee7.b6**	0.72	0.07	0.72	0.07
**ee4ee8.a**	1.79	0.11	1.79	0.11
**ee4ee8.b1**	-1.30	0.09	-1.30	0.09
**ee4ee8.b2**	-0.84	0.08	-0.84	0.08
**ee4ee8.b3**	-0.33	0.07	-0.33	0.07
**ee4ee8.b4**	0.06	0.07	0.06	0.07
**ee4ee8.b5**	0.36	0.07	0.36	0.07
**ee4ee8.b6**	0.60	0.08	0.60	0.08
**ee4ee8.b7**	0.96	0.09	0.96	0.09
**ee4ee8.b8**	1.19	0.10	1.19	0.10

Brady et al. (2021)

**ee4ee8.b9**	1.44	0.11	1.44	0.11
**ee4ee8.b10**	1.80	0.13	1.80	0.13
**ee4ee8.b11**	2.22	0.16	2.22	0.16
**ee4ee8.b12**	2.83	0.21	2.83	0.21
**ee9.a**	2.48	0.15	2.48	0.15
**ee9.b1**	-0.47	0.06	-0.47	0.06
**ee9.b2**	0.18	0.06	0.18	0.06
**ee9.b3**	0.53	0.07	0.53	0.07
**ee9.b4**	0.85	0.08	0.85	0.08
**ee9.b5**	1.18	0.09	1.18	0.09
**ee9.b6**	1.86	0.12	1.86	0.12
**Latent Mean**	0.00	NA	-0.33	0.06
**Latent Variance**	1.00	NA	0.79	0.08

Figure 3.20 Differential item and test functioning by specialty group (General Internal Medicine and Radiology) – EE subscale

a) Expected total score functions for reference (General Internal Medicine) and focal (Radiology) groups

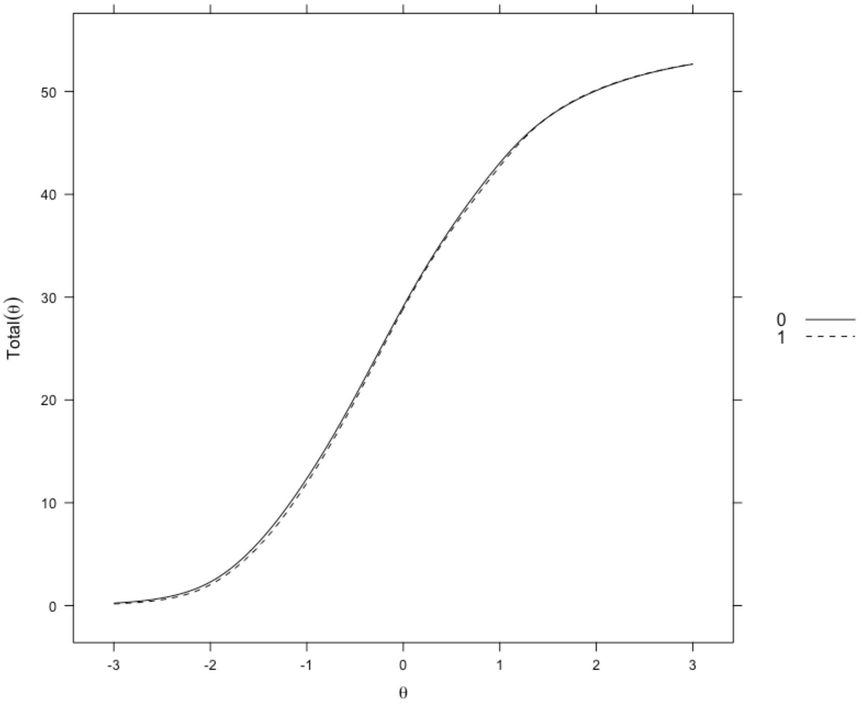

b) Expected item score functions for reference (General Internal Medicine) and focal (Radiology) groups

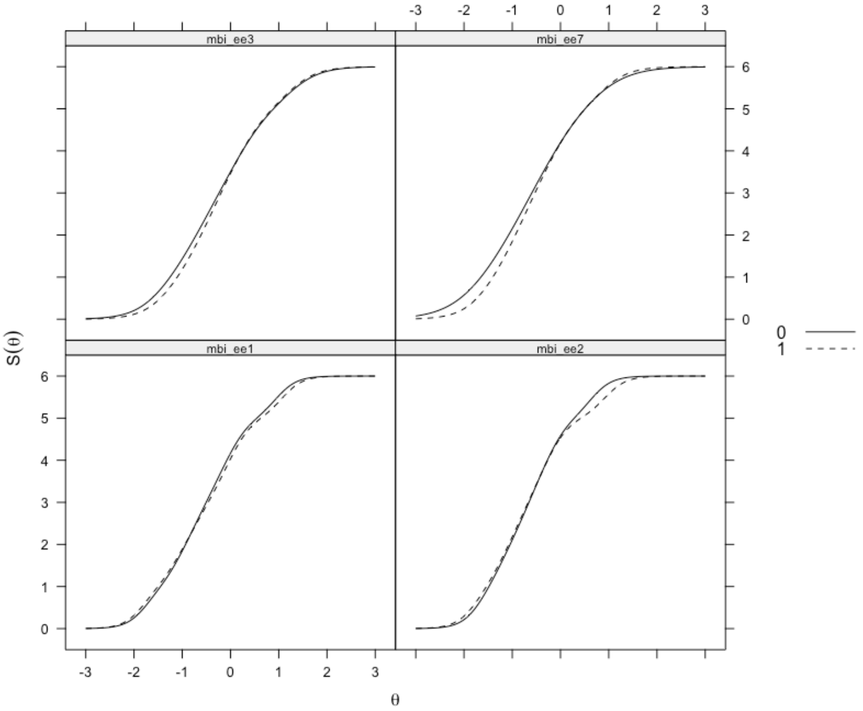

Table 3.20 Multi-group IRT item parameter estimates and standard errors (SE) by specialty group (reference: General Internal Medicine; focal: Radiology) – EE subscale

	Reference group item parameter estimates	Reference group SE	Focal group item parameter estimates	Focal group SE
**ee1.a**	4.60	0.37	4.68	0.52
**ee1.b1**	-1.74	0.11	-1.81	0.17
**ee1.b2**	-1.10	0.08	-1.16	0.11
**ee1.b3**	-0.73	0.07	-0.71	0.09
**ee1.b4**	-0.28	0.06	-0.19	0.07
**ee1.b5**	0.07	0.06	0.12	0.07
**ee1.b6**	0.96	0.08	1.08	0.10
**ee2.a**	4.59	0.38	4.48	0.52
**ee2.b1**	-1.67	0.11	-1.76	0.17
**ee2.b2**	-1.24	0.09	-1.24	0.12
**ee2.b3**	-0.82	0.07	-0.88	0.09
**ee2.b4**	-0.47	0.06	-0.45	0.08
**ee2.b5**	-0.15	0.06	-0.15	0.07
**ee2.b6**	0.65	0.07	0.93	0.10

Brady et al. (2021)

**ee3.a**	2.95	0.22	3.15	0.32
**ee3.b1**	-1.43	0.11	-1.27	0.13
**ee3.b2**	-0.88	0.08	-0.72	0.10
**ee3.b3**	-0.48	0.07	-0.41	0.09
**ee3.b4**	0.01	0.07	-0.02	0.08
**ee3.b5**	0.35	0.07	0.34	0.08
**ee3.b6**	1.25	0.10	1.23	0.13
**ee5.a**	5.04	0.36	5.04	0.36
**ee5.b1**	-1.27	0.08	-1.27	0.08
**ee5.b2**	-0.60	0.06	-0.60	0.06
**ee5.b3**	-0.28	0.06	-0.28	0.06
**ee5.b4**	0.07	0.06	0.07	0.06
**ee5.b5**	0.41	0.06	0.41	0.06
**ee5.b6**	1.05	0.08	1.05	0.08
**ee6.a**	2.76	0.18	2.76	0.18
**ee6.b1**	-1.95	0.13	-1.95	0.13
**ee6.b2**	-1.20	0.09	-1.20	0.09
**ee6.b3**	-0.83	0.07	-0.83	0.07
**ee6.b4**	-0.33	0.06	-0.33	0.06
**ee6.b5**	-0.01	0.06	-0.01	0.06
**ee6.b6**	0.74	0.07	0.74	0.07
**ee7.a**	2.27	0.18	3.25	0.34
**ee7.b1**	-1.70	0.13	-1.54	0.15
**ee7.b2**	-1.20	0.10	-1.04	0.11
**ee7.b3**	-0.79	0.09	-0.76	0.10
**ee7.b4**	-0.37	0.08	-0.35	0.08
**ee7.b5**	-0.09	0.07	0.01	0.08
**ee7.b6**	0.69	0.09	0.85	0.10
**ee4ee8.a**	1.66	0.12	1.66	0.12
**ee4ee8.b1**	-1.07	0.10	-1.07	0.10
**ee4ee8.b2**	-0.64	0.08	-0.64	0.08
**ee4ee8.b3**	-0.21	0.07	-0.21	0.07
**ee4ee8.b4**	0.18	0.08	0.18	0.08
**ee4ee8.b5**	0.51	0.08	0.51	0.08
**ee4ee8.b6**	0.79	0.09	0.79	0.09
**ee4ee8.b7**	1.23	0.11	1.23	0.11
**ee4ee8.b8**	1.44	0.12	1.44	0.12

Brady et al. (2021)

**ee4ee8.b9**	1.70	0.13	1.70	0.13
**ee4ee8.b10**	1.95	0.15	1.95	0.15
**ee4ee8.b11**	2.50	0.19	2.50	0.19
**ee4ee8.b12**	3.10	0.25	3.10	0.25
**ee9.a**	2.69	0.18	2.69	0.18
**ee9.b1**	-0.53	0.07	-0.53	0.07
**ee9.b2**	0.06	0.06	0.06	0.06
**ee9.b3**	0.35	0.07	0.35	0.07
**ee9.b4**	0.74	0.07	0.74	0.07
**ee9.b5**	1.10	0.08	1.10	0.08
**ee9.b6**	1.76	0.12	1.76	0.12
**Latent Mean**	0.00	NA	0.03	0.08
**Latent Variance**	1.00	NA	0.75	0.10

Figure 3.21 Differential item and test functioning by gender (Male and Female) – DP subscale

a) Expected total score functions for reference (Male) and focal (Female) groups

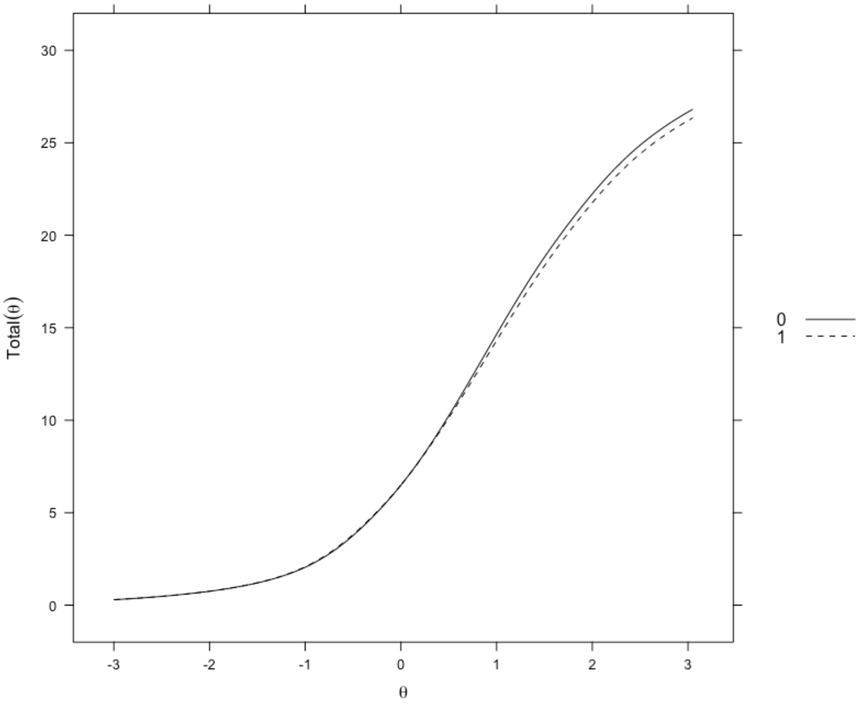

b) Expected item score functions for reference (Male) and focal (Female) groups

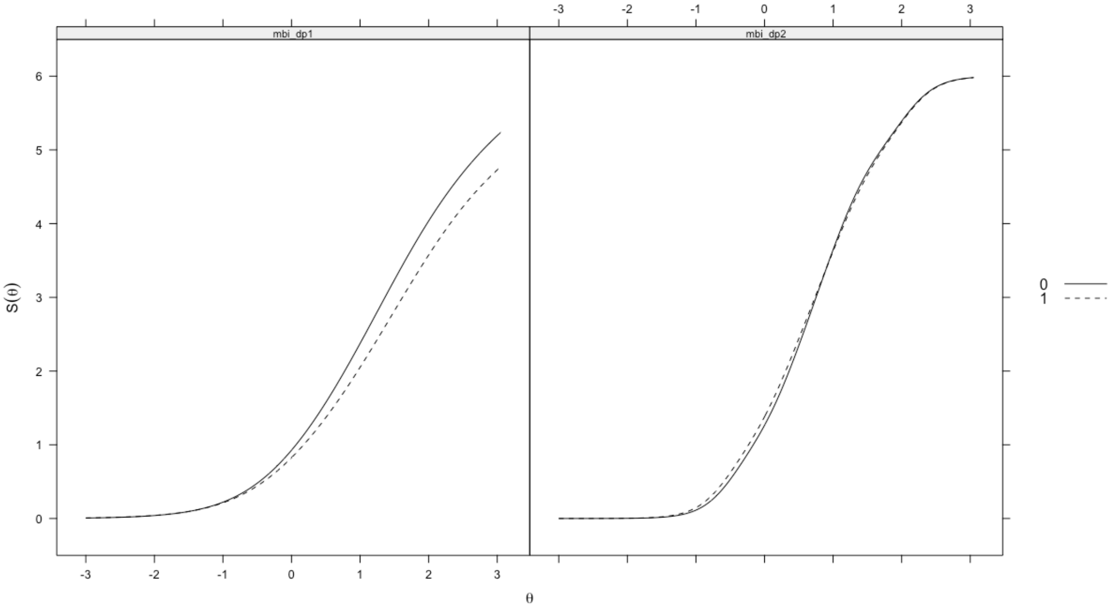

Table 3.21 Multi-group IRT item parameter estimates and standard errors (SE) by gender group (reference: Male; focal: Female) – DP subscale

	**Reference group item parameter estimates**	**Reference group SE**	**Focal group item parameter estimates**	**Focal group SE**
mbi_dp1.a	1.81	0.06	1.74	0.09
mbi_dp1.b1	-0.01	0.03	0.01	0.04
mbi_dp1.b2	0.69	0.03	0.86	0.05
mbi_dp1.b3	1.12	0.04	1.34	0.06
mbi_dp1.b4	1.56	0.04	1.84	0.08
mbi_dp1.b5	1.98	0.05	2.31	0.10
mbi_dp1.b6	3.11	0.10	3.94	0.24
mbi_dp2.a	4.01	0.17	3.99	0.25
mbi_dp2.b1	-0.46	0.02	-0.53	0.04
mbi_dp2.b2	0.24	0.02	0.16	0.03
mbi_dp2.b3	0.57	0.02	0.53	0.03
mbi_dp2.b4	0.93	0.03	0.95	0.04
mbi_dp2.b5	1.26	0.03	1.31	0.05
mbi_dp2.b6	2.04	0.05	2.05	0.07
mbi_dp3.a	2.70	0.07	2.70	0.07
mbi_dp3.b1	-0.52	0.02	-0.52	0.02
mbi_dp3.b2	0.14	0.02	0.14	0.02

mbi_dp3.b3	0.48	0.02	0.48	0.02
mbi_dp3.b4	0.81	0.02	0.81	0.02
mbi_dp3.b5	1.13	0.03	1.13	0.03
mbi_dp3.b6	1.66	0.04	1.66	0.04
mbi_dp4.a	1.58	0.05	1.58	0.05
mbi_dp4.b1	0.50	0.03	0.50	0.03
mbi_dp4.b2	1.33	0.04	1.33	0.04
mbi_dp4.b3	1.83	0.05	1.83	0.05
mbi_dp4.b4	2.27	0.06	2.27	0.06
mbi_dp4.b5	2.70	0.08	2.70	0.08
mbi_dp4.b6	3.61	0.12	3.61	0.12
mbi_dp5.a	1.08	0.03	1.08	0.03
mbi_dp5.b1	-1.67	0.06	-1.67	0.06
mbi_dp5.b2	-0.18	0.03	-0.18	0.03
mbi_dp5.b3	0.46	0.03	0.46	0.03
mbi_dp5.b4	1.08	0.04	1.08	0.04
mbi_dp5.b5	1.69	0.06	1.69	0.06
mbi_dp5.b6	2.98	0.09	2.98	0.09
Latent Mean	0.00	NA	0.08	0.03
Latent Variance	1.00	NA	0.95	0.06

Figure 3.22 Differential item and test functioning by age group (≥65 years and <35 years) – DP subscale

a) Expected total score functions for reference (≥65 years) and focal (<35 years) groups

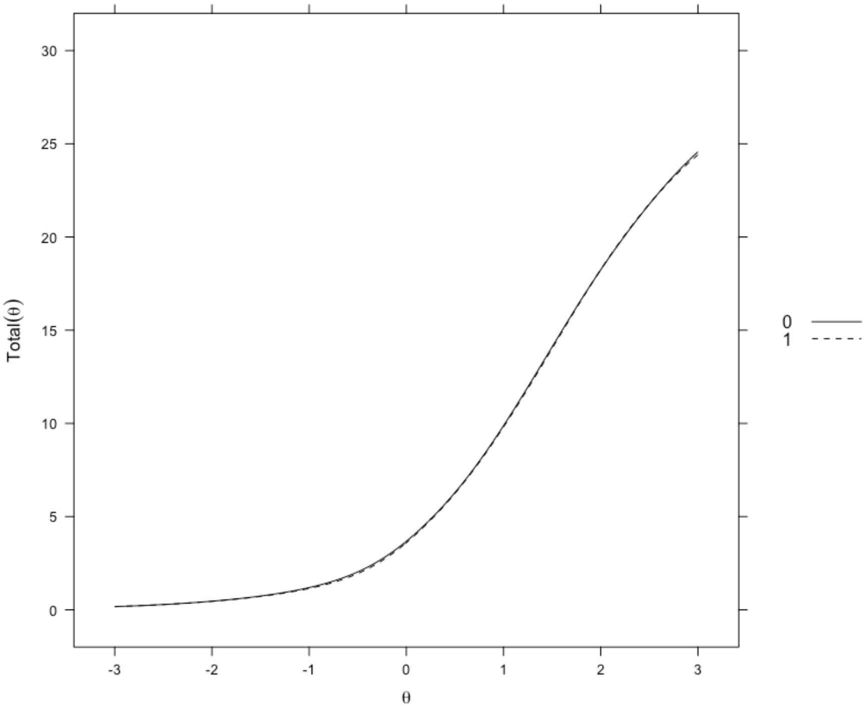

b) Expected item score functions for reference (≥65 years) and focal (<35 years) groups

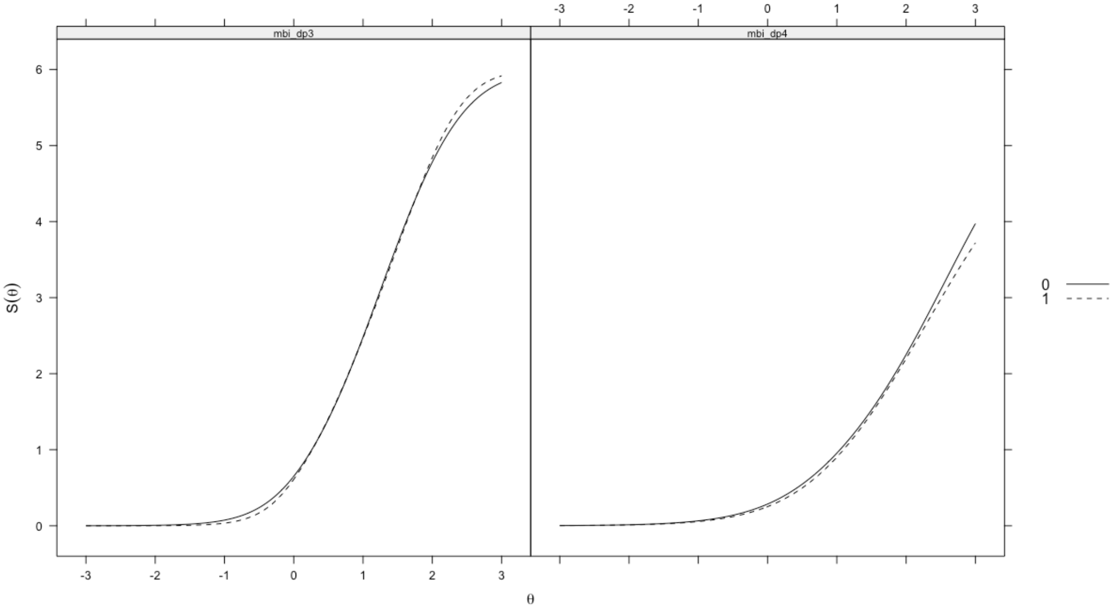

Table 3.22 Multi-group IRT item parameter estimates and standard errors (SE) by age group (reference: ≥65 years; focal: <35 years) – DP subscale

	**Reference group item parameter estimates**	**Reference group SE**	**Focal group item parameter estimates**	**Focal group SE**
mbi_dp1.a	1.69	0.10	1.69	0.10
mbi_dp1.b1	0.48	0.05	0.48	0.05
mbi_dp1.b2	1.28	0.06	1.28	0.06
mbi_dp1.b3	1.82	0.08	1.82	0.08
mbi_dp1.b4	2.33	0.11	2.33	0.11
mbi_dp1.b5	2.83	0.13	2.83	0.13
mbi_dp1.b6	3.99	0.24	3.99	0.24
mbi_dp2.a	3.50	0.24	3.50	0.24
mbi_dp2.b1	0.02	0.04	0.02	0.04
mbi_dp2.b2	0.73	0.04	0.73	0.04
mbi_dp2.b3	1.07	0.05	1.07	0.05
mbi_dp2.b4	1.49	0.06	1.49	0.06
mbi_dp2.b5	1.78	0.07	1.78	0.07
mbi_dp2.b6	2.60	0.11	2.60	0.11
mbi_dp3.a	2.53	0.16	3.35	0.41
mbi_dp3.b1	0.14	0.04	0.04	0.09
mbi_dp3.b2	0.77	0.05	0.63	0.07
mbi_dp3.b3	1.07	0.05	1.03	0.07
mbi_dp3.b4	1.38	0.06	1.47	0.08
mbi_dp3.b5	1.66	0.07	1.77	0.10
mbi_dp3.b6	2.18	0.10	2.18	0.12
mbi_dp4.a	1.60	0.12	1.60	0.21
mbi_dp4.b1	0.89	0.06	1.08	0.10
mbi_dp4.b2	1.89	0.11	1.83	0.14
mbi_dp4.b3	2.38	0.14	2.27	0.17
mbi_dp4.b4	2.70	0.16	2.60	0.20
mbi_dp4.b5	2.92	0.18	3.17	0.27
mbi_dp4.b6	3.61	0.25	4.86	0.59
mbi_dp5.a	1.09	0.06	1.09	0.06
mbi_dp5.b1	-1.14	0.09	-1.14	0.09
mbi_dp5.b2	0.34	0.06	0.34	0.06
mbi_dp5.b3	1.00	0.07	1.00	0.07
mbi_dp5.b4	1.67	0.10	1.67	0.10

Brady et al. (2021)

mbi_dp5.b5	2.29	0.12	2.29	0.12
mbi_dp5.b6	3.53	0.20	3.53	0.20
Latent Mean	0.00	NA	0.93	0.07
Latent Variance	1.00	NA	0.94	0.12

Figure 3.23 Differential item and test functioning by age group (≥65 years and 35-44 years) – DP subscale

a) Expected total score functions for reference (≥65 years) and focal (35-44 years) groups

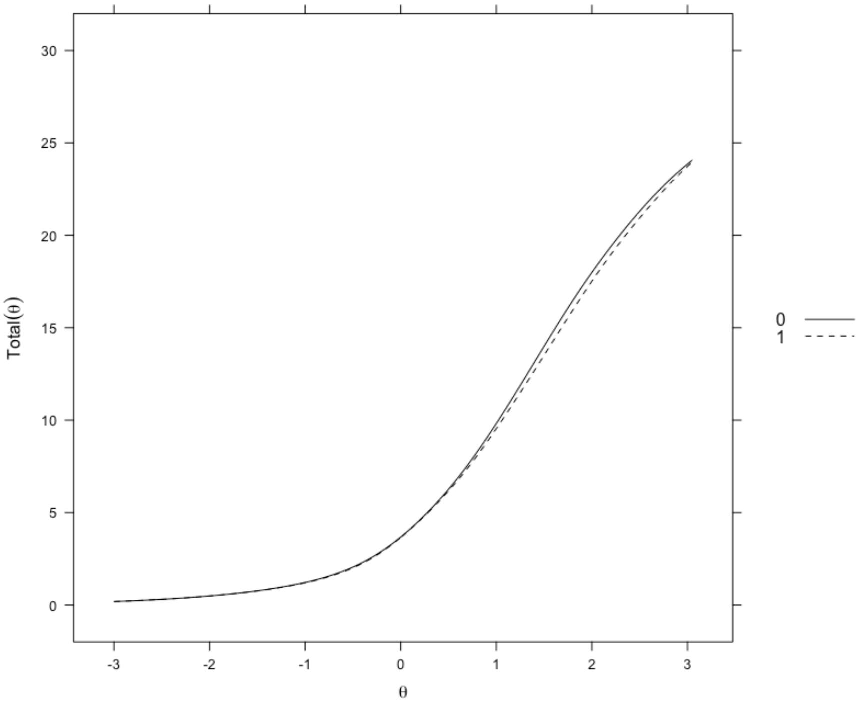

b) Expected item score functions for reference (≥65 years) and focal (35-44 years) groups

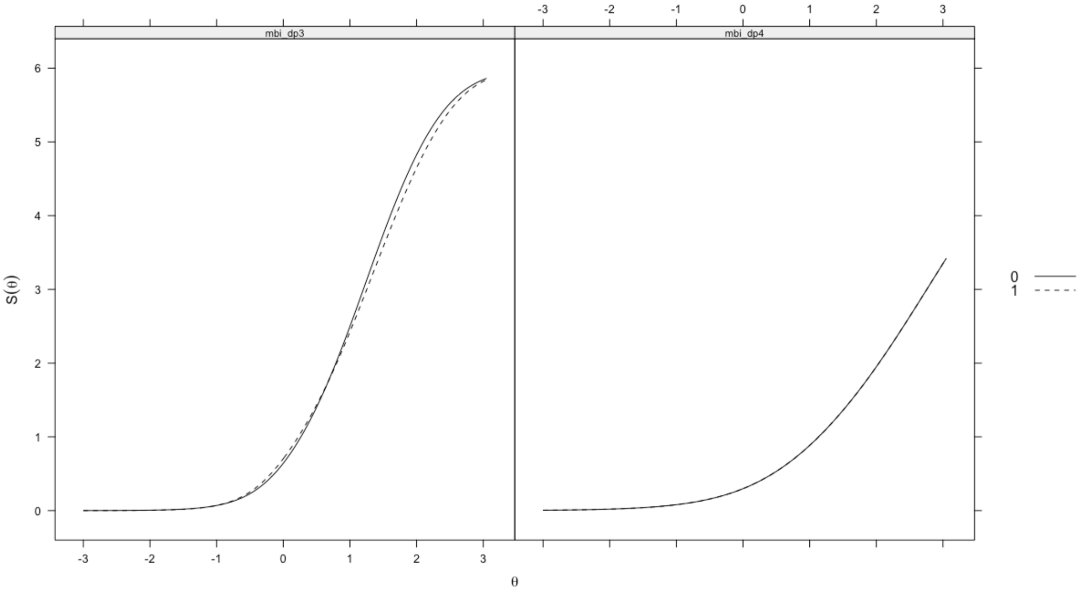

Table 3.23 Multi-group IRT item parameter estimates and standard errors (SE) by age group (reference: ≥65 years; focal: 35-44 years) – DP subscale

	**Reference group item parameter estimates**	**Reference group SE**	**Focal group item parameter estimates**	**Focal group SE**
mbi_dp1.a	1.60	0.08	1.60	0.08
mbi_dp1.b1	0.52	0.05	0.52	0.05
mbi_dp1.b2	1.29	0.06	1.29	0.06
mbi_dp1.b3	1.82	0.08	1.82	0.08
mbi_dp1.b4	2.32	0.10	2.32	0.10
mbi_dp1.b5	2.78	0.13	2.78	0.13
mbi_dp1.b6	4.26	0.23	4.26	0.23
mbi_dp2.a	3.47	0.27	4.29	0.43
mbi_dp2.b1	0.02	0.04	0.04	0.07
mbi_dp2.b2	0.72	0.04	0.74	0.06
mbi_dp2.b3	1.05	0.05	1.13	0.06
mbi_dp2.b4	1.47	0.06	1.58	0.08
mbi_dp2.b5	1.69	0.07	1.97	0.09
mbi_dp2.b6	2.50	0.12	2.75	0.14
mbi_dp3.a	2.58	0.16	2.84	0.21
mbi_dp3.b1	0.14	0.04	-0.02	0.07

mbi_dp3.b2	0.77	0.05	0.69	0.06
mbi_dp3.b3	1.06	0.05	1.09	0.06
mbi_dp3.b4	1.36	0.06	1.45	0.07
mbi_dp3.b5	1.64	0.07	1.80	0.09
mbi_dp3.b6	2.16	0.10	2.33	0.11
mbi_dp4.a	1.43	0.09	1.43	0.09
mbi_dp4.b1	0.99	0.06	0.99	0.06
mbi_dp4.b2	1.99	0.10	1.99	0.10
mbi_dp4.b3	2.57	0.12	2.57	0.12
mbi_dp4.b4	3.00	0.15	3.00	0.15
mbi_dp4.b5	3.49	0.18	3.49	0.18
mbi_dp4.b6	4.45	0.25	4.45	0.25
mbi_dp5.a	1.05	0.06	1.05	0.06
mbi_dp5.b1	-1.15	0.09	-1.15	0.09
mbi_dp5.b2	0.39	0.05	0.39	0.05
mbi_dp5.b3	1.13	0.07	1.13	0.07
mbi_dp5.b4	1.75	0.09	1.75	0.09
mbi_dp5.b5	2.35	0.11	2.35	0.11
mbi_dp5.b6	3.69	0.18	3.69	0.18
Latent Mean	0.00	NA	0.94	0.06
Latent Variance	1.00	NA	0.92	0.10

Figure 3.24 Differential item and test functioning by age group (≥65 years and 45-54 years) – DP subscale

a) Expected total score functions for reference (≥65 years) and focal (45-54 years) groups

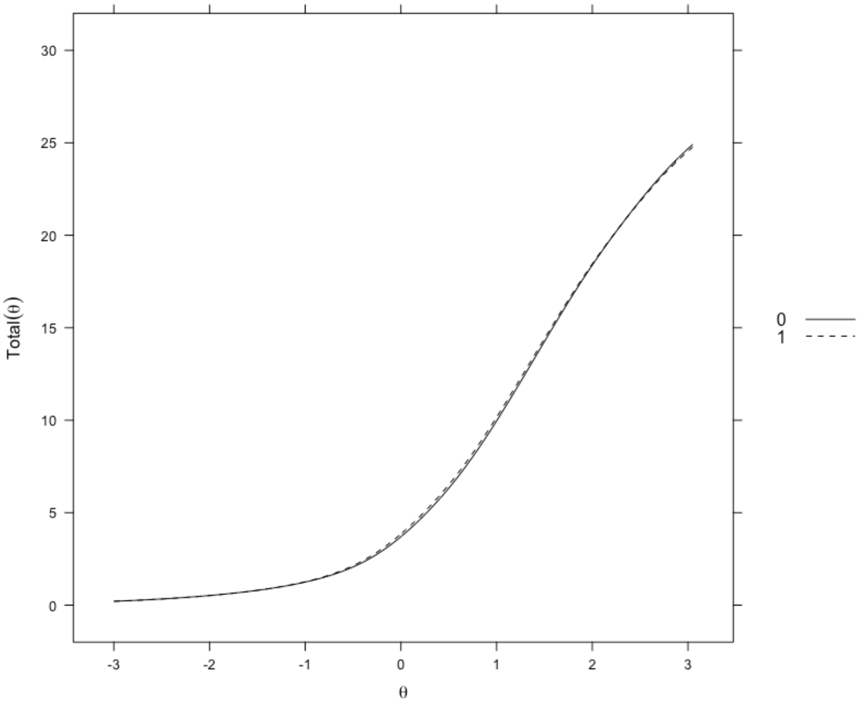

b) Expected item score functions for reference (≥65 years) and focal (45-54 years) groups

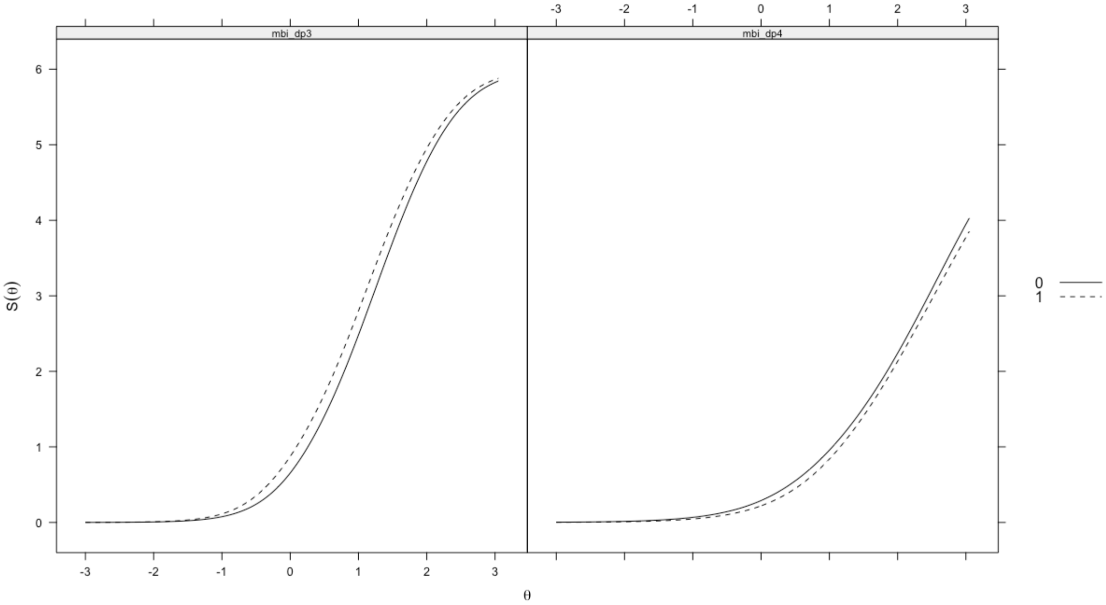

Table 3.24 Multi-group IRT item parameter estimates and standard errors (SE) by age group (reference: ≥65 years; focal: 45-54 years) – DP subscale

	**Reference group item parameter estimates**	**Reference group SE**	**Focal group item parameter estimates**	**Focal group SE**
mbi_dp1.a	1.74	0.08	1.74	0.08
mbi_dp1.b1	0.52	0.04	0.52	0.04
mbi_dp1.b2	1.30	0.06	1.30	0.06
mbi_dp1.b3	1.77	0.07	1.77	0.07
mbi_dp1.b4	2.25	0.09	2.25	0.09
mbi_dp1.b5	2.68	0.11	2.68	0.11
mbi_dp1.b6	3.81	0.17	3.81	0.17
mbi_dp2.a	3.81	0.23	3.81	0.23
mbi_dp2.b1	0.02	0.04	0.02	0.04
mbi_dp2.b2	0.72	0.04	0.72	0.04
mbi_dp2.b3	1.05	0.04	1.05	0.04
mbi_dp2.b4	1.43	0.05	1.43	0.05
mbi_dp2.b5	1.77	0.06	1.77	0.06
mbi_dp2.b6	2.60	0.10	2.60	0.10
mbi_dp3.a	2.51	0.15	2.62	0.16
mbi_dp3.b1	0.14	0.04	-0.11	0.05
mbi_dp3.b2	0.77	0.05	0.61	0.05
mbi_dp3.b3	1.07	0.05	0.95	0.05
mbi_dp3.b4	1.37	0.06	1.23	0.06
mbi_dp3.b5	1.65	0.07	1.58	0.07
mbi_dp3.b6	2.18	0.10	2.14	0.09
mbi_dp4.a	1.59	0.11	1.65	0.12
mbi_dp4.b1	0.89	0.06	1.13	0.06
mbi_dp4.b2	1.90	0.11	1.83	0.09
mbi_dp4.b3	2.39	0.14	2.31	0.11
mbi_dp4.b4	2.71	0.16	2.78	0.14
mbi_dp4.b5	2.94	0.18	3.16	0.16
mbi_dp4.b6	3.63	0.25	3.98	0.23
mbi_dp5.a	1.03	0.05	1.03	0.05
mbi_dp5.b1	-1.26	0.09	-1.26	0.09
mbi_dp5.b2	0.33	0.05	0.33	0.05
mbi_dp5.b3	1.00	0.06	1.00	0.06
mbi_dp5.b4	1.64	0.08	1.64	0.08

Brady et al. (2021)

mbi_dp5.b5	2.27	0.10	2.27	0.10
mbi_dp5.b6	3.60	0.17	3.60	0.17
Latent Mean	0.00	NA	0.69	0.05
Latent Variance	1.00	NA	0.97	0.08

Figure 3.25 Differential item and test functioning by age (≥65 years and 55-64 years) – DP subscale

a) Expected total score functions for reference (≥65 years) and focal (55-64 years) groups

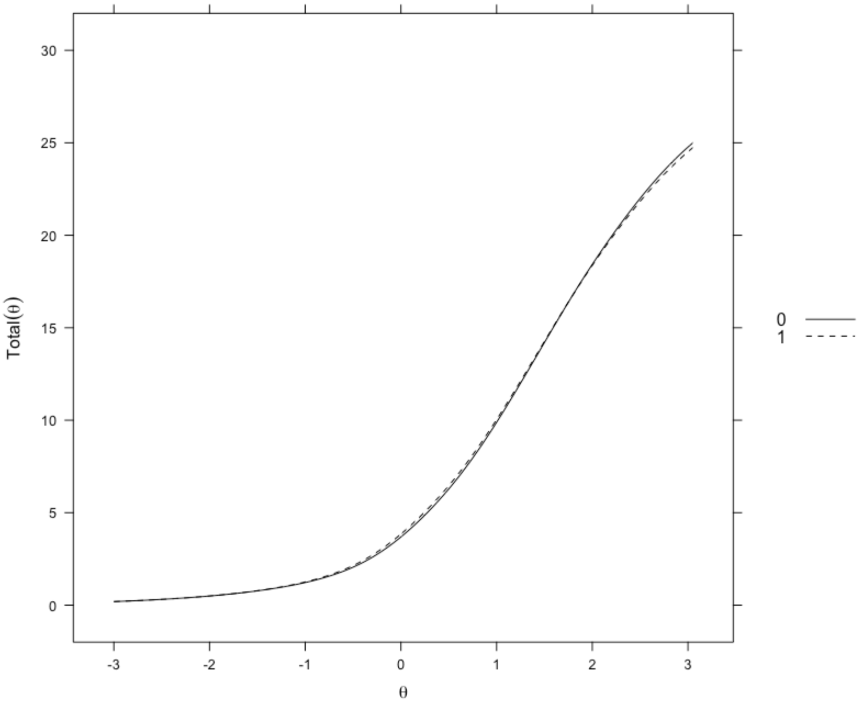

b) Expected item score functions for reference (≥65 years) and focal (55-64 years) groups

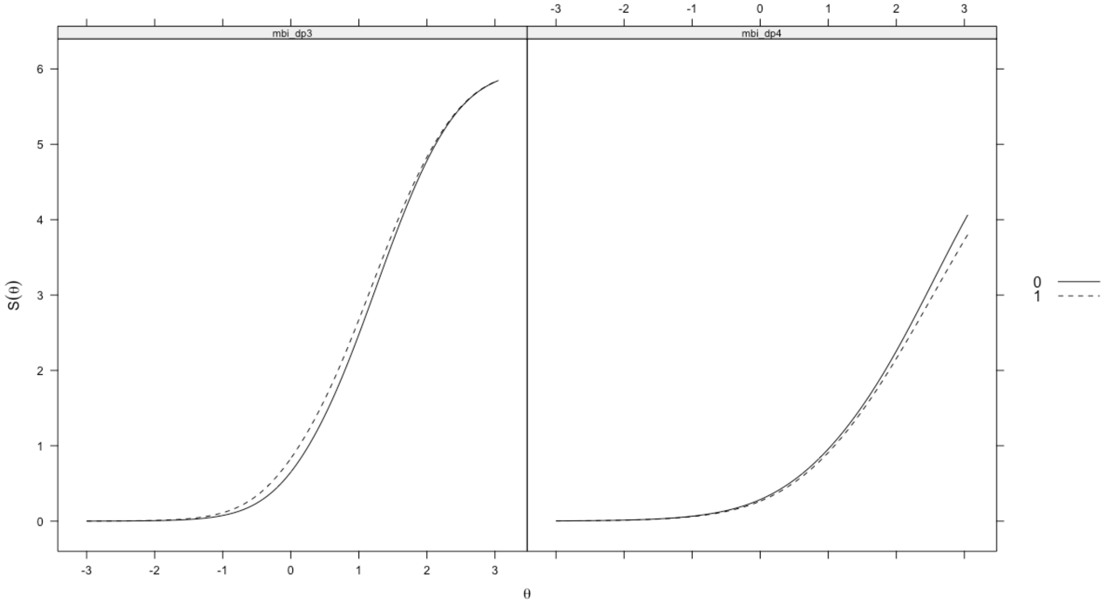

Table 3.25 Multi-group IRT item parameter estimates and standard errors (SE) by age group (reference: ≥65 years; focal: 55-64 years) – DP subscale

	Reference group item parameter estimates	Reference group SE	Focal group item parameter estimates	Focal group SE
mbi_dp1.a	1.71	0.08	1.71	0.08
mbi_dp1.b1	0.53	0.04	0.53	0.04
mbi_dp1.b2	1.30	0.05	1.30	0.05
mbi_dp1.b3	1.76	0.07	1.76	0.07
mbi_dp1.b4	2.22	0.08	2.22	0.08
mbi_dp1.b5	2.61	0.10	2.61	0.10
mbi_dp1.b6	3.85	0.17	3.85	0.17
mbi_dp2.a	3.61	0.20	3.61	0.20
mbi_dp2.b1	0.01	0.04	0.01	0.04
mbi_dp2.b2	0.74	0.04	0.74	0.04
mbi_dp2.b3	1.09	0.04	1.09	0.04
mbi_dp2.b4	1.48	0.05	1.48	0.05
mbi_dp2.b5	1.76	0.06	1.76	0.06
mbi_dp2.b6	2.53	0.09	2.53	0.09
mbi_dp3.a	2.52	0.15	2.53	0.14
mbi_dp3.b1	0.14	0.04	-0.06	0.05

mbi_dp3.b2	0.78	0.05	0.65	0.04
mbi_dp3.b3	1.07	0.05	0.99	0.05
mbi_dp3.b4	1.38	0.06	1.32	0.06
mbi_dp3.b5	1.65	0.07	1.64	0.07
mbi_dp3.b6	2.18	0.10	2.20	0.09
mbi_dp4.a	1.60	0.12	1.58	0.10
mbi_dp4.b1	0.89	0.06	1.01	0.05
mbi_dp4.b2	1.89	0.11	1.83	0.08
mbi_dp4.b3	2.38	0.14	2.33	0.11
mbi_dp4.b4	2.70	0.16	2.79	0.13
mbi_dp4.b5	2.92	0.18	3.20	0.16
mbi_dp4.b6	3.60	0.25	4.07	0.23
mbi_dp5.a	1.06	0.05	1.06	0.05
mbi_dp5.b1	-1.22	0.08	-1.22	0.08
mbi_dp5.b2	0.35	0.05	0.35	0.05
mbi_dp5.b3	1.01	0.06	1.01	0.06
mbi_dp5.b4	1.66	0.08	1.66	0.08
mbi_dp5.b5	2.30	0.10	2.30	0.10
mbi_dp5.b6	3.55	0.16	3.55	0.16
Latent Mean	0.00	NA	0.51	0.04
Latent Variance	1.00	NA	0.93	0.07

Figure 3.26 Differential item and test functioning by specialty group (General Internal Medicine and Anesthesiology) – DP subscale

a) Expected total score functions for reference (General Internal Medicine) and focal (Anesthesiology) groups

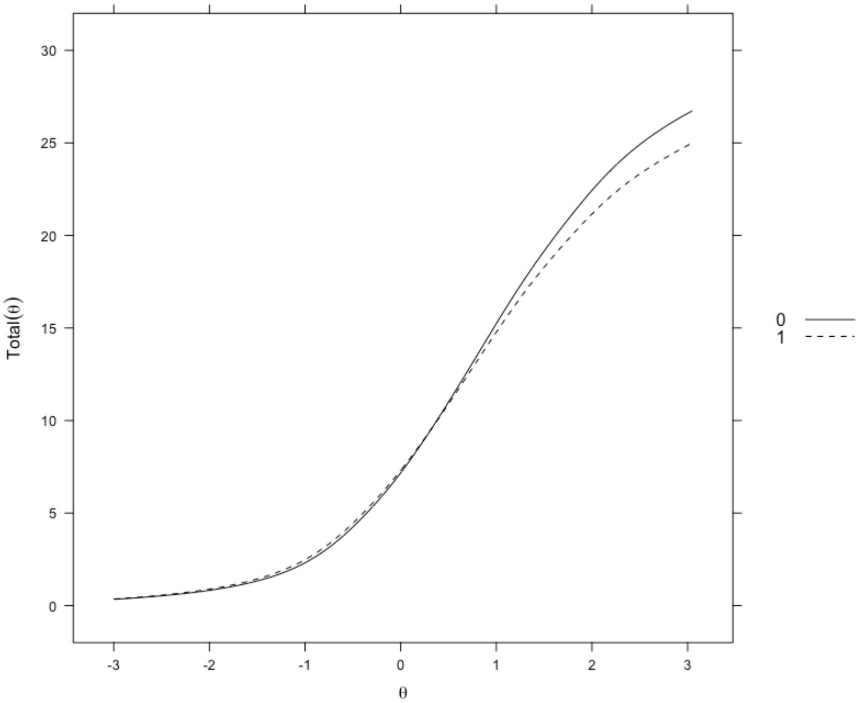

b) Expected item score functions for reference (General Internal Medicine) and focal (Anesthesiology) groups

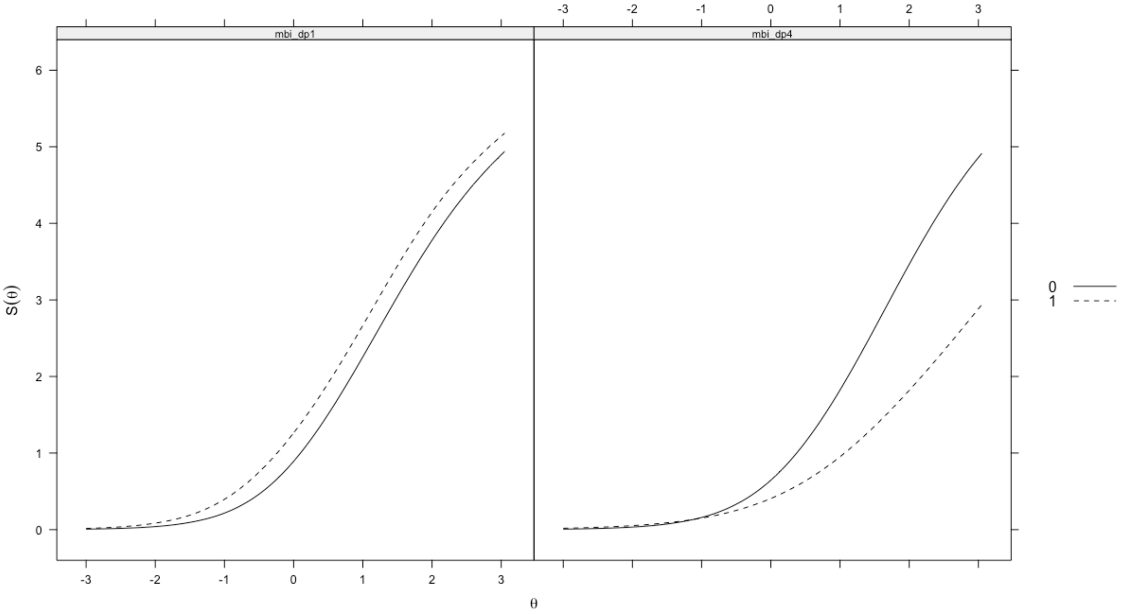

Table 3.26 Multi-group IRT item parameter estimates and standard errors (SE) by specialty group (reference: General Internal Medicine; focal: Anesthesiology) – DP subscale

	**Reference group item parameter estimates**	**Reference group SE**	**Focal group item parameter estimates**	**Focal group SE**
mbi_dp1.a	1.75	0.15	1.75	0.15
mbi_dp1.b1	-0.08	0.07	-0.08	0.07
mbi_dp1.b2	0.64	0.08	0.64	0.08
mbi_dp1.b3	1.10	0.10	1.10	0.10
mbi_dp1.b4	1.63	0.13	1.63	0.13
mbi_dp1.b5	2.16	0.17	2.16	0.17
mbi_dp1.b6	3.52	0.35	3.52	0.35
mbi_dp2.a	4.34	0.55	4.34	0.55
mbi_dp2.b1	-0.51	0.07	-0.51	0.07
mbi_dp2.b2	0.11	0.06	0.11	0.06
mbi_dp2.b3	0.49	0.06	0.49	0.06
mbi_dp2.b4	0.87	0.07	0.87	0.07
mbi_dp2.b5	1.19	0.08	1.19	0.08
mbi_dp2.b6	1.93	0.12	1.93	0.12
mbi_dp3.a	2.26	0.20	2.75	0.38
mbi_dp3.b1	-0.74	0.09	-0.53	0.11
mbi_dp3.b2	-0.10	0.07	0.03	0.09
mbi_dp3.b3	0.23	0.07	0.42	0.09
mbi_dp3.b4	0.56	0.08	0.73	0.10
mbi_dp3.b5	0.94	0.09	1.13	0.12
mbi_dp3.b6	1.60	0.12	1.62	0.17
mbi_dp4.a	1.62	0.18	1.11	0.25
mbi_dp4.b1	0.41	0.09	1.26	0.26
mbi_dp4.b2	1.05	0.12	1.94	0.39
mbi_dp4.b3	1.53	0.15	2.73	0.57
mbi_dp4.b4	1.87	0.18	3.82	0.85
mbi_dp4.b5	2.34	0.22	4.31	1.00
mbi_dp4.b6	3.26	0.35	4.69	1.13
mbi_dp5.a	0.98	0.10	0.98	0.10
mbi_dp5.b1	-1.64	0.18	-1.64	0.18
mbi_dp5.b2	-0.01	0.10	-0.01	0.10
mbi_dp5.b3	0.69	0.12	0.69	0.12
mbi_dp5.b4	1.26	0.15	1.26	0.15

Brady et al. (2021)

mbi_dp5.b5	1.93	0.20	1.93	0.20
mbi_dp5.b6	3.21	0.33	3.21	0.33
Latent Mean	0.00	NA	0.03	0.09
Latent Variance	1.00	NA	0.89	0.15

Figure 3.27 Differential item and test functioning by specialty group (General Internal Medicine and Emergency Medicine) – DP subscale

a) Expected total score functions for reference (General Internal Medicine) and focal (Emergency Medicine) groups

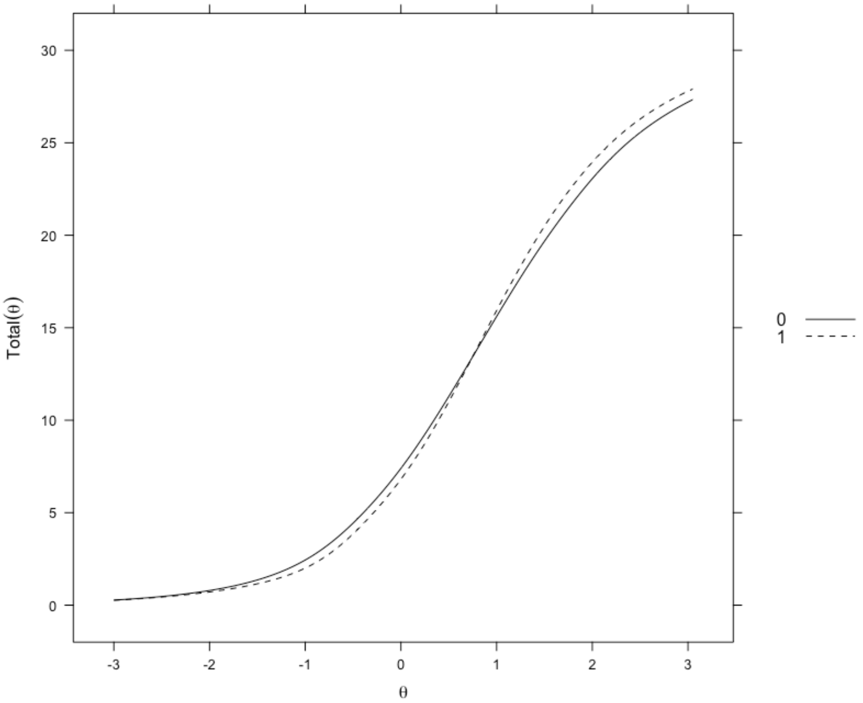

b) Expected item score functions for reference (General Internal Medicine) and focal (Emergency Medicine) groups

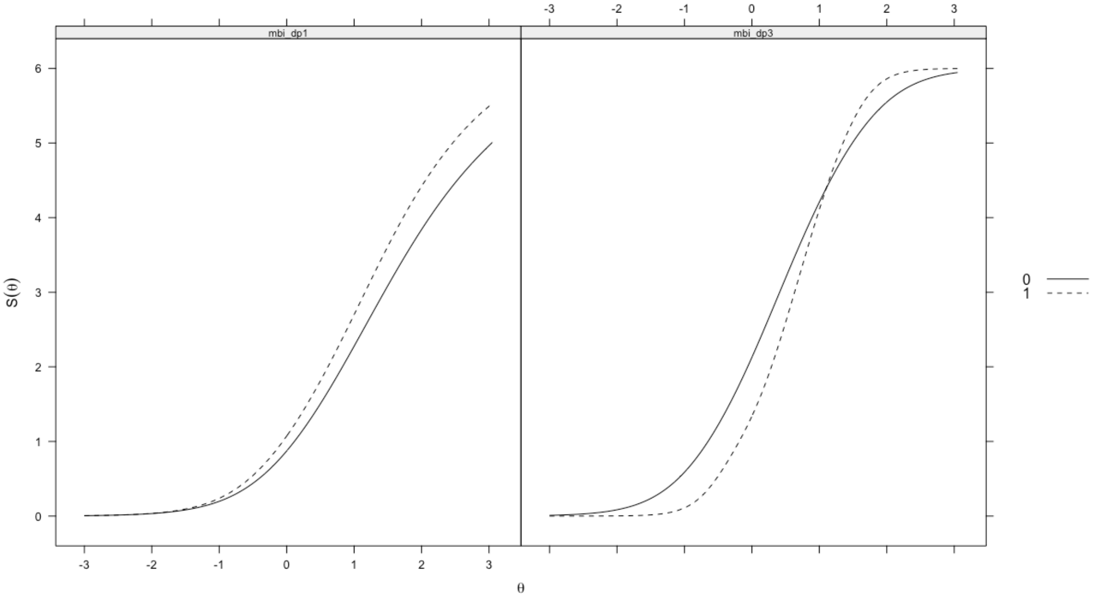

Table 3.27 Multi-group IRT item parameter estimates and standard errors (SE) by specialty group (reference: General Internal Medicine; focal: Emergency Medicine) – DP subscale

	**Reference group item parameter estimates**	**Reference group SE**	**Focal group item parameter estimates**	**Focal group SE**
mbi_dp1.a	1.86	0.19	2.01	0.22
mbi_dp1.b1	0.04	0.08	-0.17	0.11
mbi_dp1.b2	0.70	0.09	0.51	0.09
mbi_dp1.b3	1.10	0.11	0.95	0.10
mbi_dp1.b4	1.64	0.14	1.37	0.12
mbi_dp1.b5	2.24	0.19	1.75	0.14
mbi_dp1.b6	3.50	0.40	2.74	0.23
mbi_dp2.a	3.80	0.36	3.80	0.36
mbi_dp2.b1	-0.53	0.07	-0.53	0.07
mbi_dp2.b2	0.18	0.06	0.18	0.06
mbi_dp2.b3	0.52	0.06	0.52	0.06
mbi_dp2.b4	0.92	0.07	0.92	0.07
mbi_dp2.b5	1.26	0.08	1.26	0.08
mbi_dp2.b6	1.95	0.12	1.95	0.12
mbi_dp3.a	2.22	0.20	4.09	0.52
mbi_dp3.b1	-0.74	0.09	-0.46	0.10

mbi_dp3.b2	-0.10	0.07	0.21	0.08
mbi_dp3.b3	0.23	0.07	0.50	0.08
mbi_dp3.b4	0.57	0.08	0.78	0.08
mbi_dp3.b5	0.94	0.09	1.07	0.09
mbi_dp3.b6	1.61	0.12	1.49	0.11
mbi_dp4.a	1.68	0.14	1.68	0.14
mbi_dp4.b1	0.31	0.07	0.31	0.07
mbi_dp4.b2	1.01	0.09	1.01	0.09
mbi_dp4.b3	1.47	0.11	1.47	0.11
mbi_dp4.b4	1.84	0.13	1.84	0.13
mbi_dp4.b5	2.20	0.15	2.20	0.15
mbi_dp4.b6	3.20	0.24	3.20	0.24
mbi_dp5.a	1.14	0.10	1.14	0.10
mbi_dp5.b1	-1.60	0.16	-1.60	0.16
mbi_dp5.b2	-0.25	0.09	-0.25	0.09
mbi_dp5.b3	0.32	0.09	0.32	0.09
mbi_dp5.b4	0.87	0.10	0.87	0.10
mbi_dp5.b5	1.38	0.12	1.38	0.12
mbi_dp5.b6	2.50	0.19	2.50	0.19
Latent Mean	0.00	NA	0.64	0.08
Latent Variance	1.00	NA	0.86	0.12

Figure 3.28 Differential item and test functioning by specialty group (General Internal Medicine and Family Medicine) – DP subscale

a) Expected total score functions for reference (General Internal Medicine) and focal (Family Medicine) groups

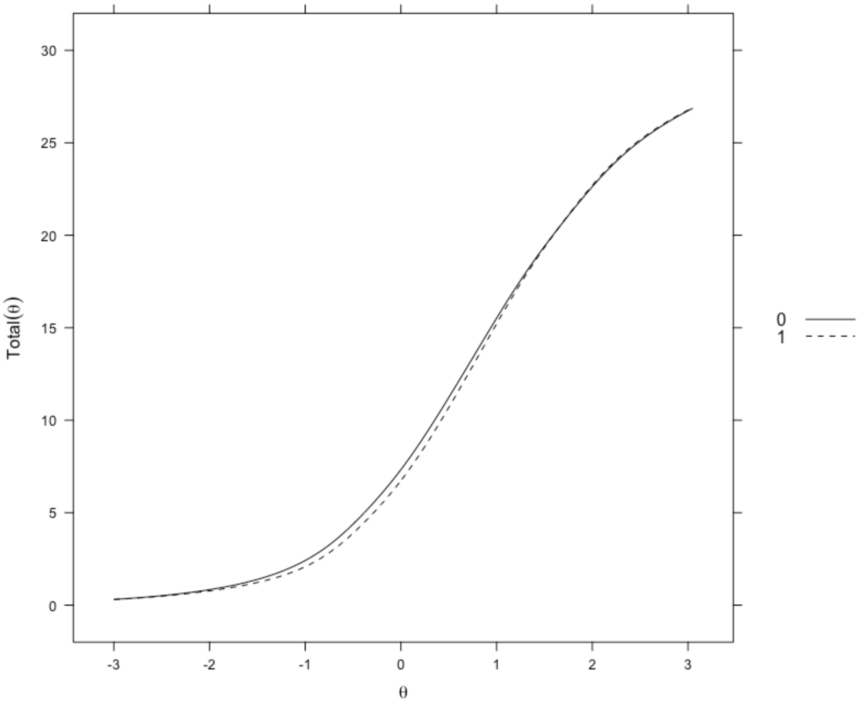

b) Expected item score functions for reference (General Internal Medicine) and focal (Family Medicine) groups

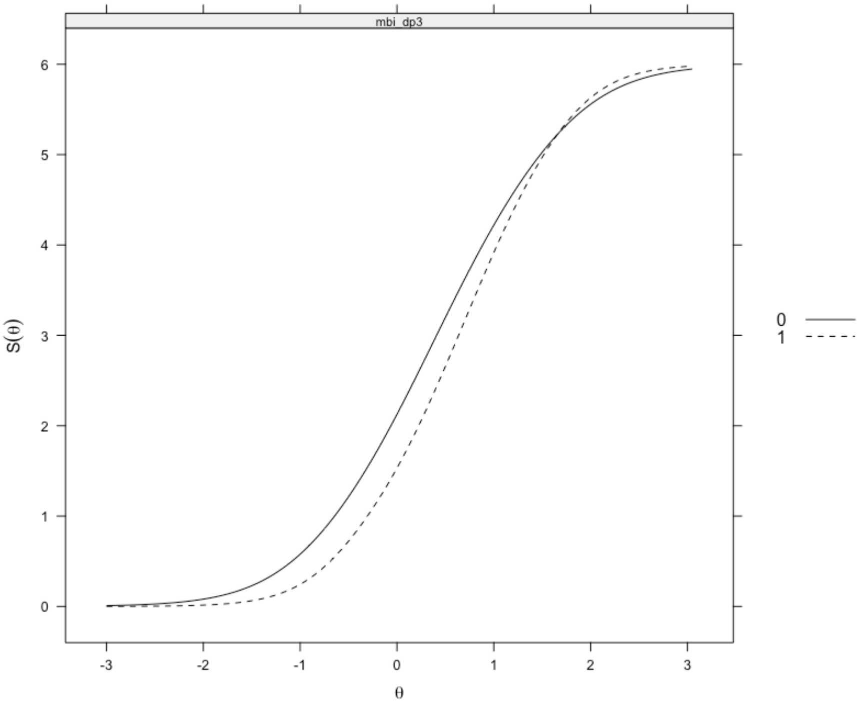

Table 3.28 Multi-group IRT item parameter estimates and standard errors (SE) by specialty group (reference: General Internal Medicine; focal: Family Medicine) – DP subscale

	**Reference group item parameter estimates**	**Reference group SE**	**Focal group item parameter estimates**	**Focal group SE**
mbi_dp1.a	1.79	0.14	1.79	0.14
mbi_dp1.b1	0.04	0.07	0.04	0.07
mbi_dp1.b2	0.74	0.08	0.74	0.08
mbi_dp1.b3	1.18	0.09	1.18	0.09
mbi_dp1.b4	1.66	0.12	1.66	0.12
mbi_dp1.b5	2.16	0.15	2.16	0.15
mbi_dp1.b6	3.75	0.34	3.75	0.34
mbi_dp2.a	4.52	0.48	4.52	0.48
mbi_dp2.b1	-0.51	0.06	-0.51	0.06
mbi_dp2.b2	0.16	0.06	0.16	0.06
mbi_dp2.b3	0.47	0.06	0.47	0.06
mbi_dp2.b4	0.85	0.07	0.85	0.07
mbi_dp2.b5	1.17	0.08	1.17	0.08
mbi_dp2.b6	1.96	0.11	1.96	0.11

mbi_dp3.a	2.24	0.20	2.98	0.28
mbi_dp3.b1	-0.74	0.09	-0.52	0.09
mbi_dp3.b2	-0.10	0.07	0.15	0.07
mbi_dp3.b3	0.23	0.07	0.49	0.07
mbi_dp3.b4	0.56	0.08	0.82	0.08
mbi_dp3.b5	0.94	0.09	1.11	0.09
mbi_dp3.b6	1.60	0.12	1.65	0.12
mbi_dp4.a	1.54	0.13	1.54	0.13
mbi_dp4.b1	0.31	0.07	0.31	0.07
mbi_dp4.b2	1.09	0.10	1.09	0.10
mbi_dp4.b3	1.61	0.12	1.61	0.12
mbi_dp4.b4	2.03	0.15	2.03	0.15
mbi_dp4.b5	2.43	0.18	2.43	0.18
mbi_dp4.b6	3.35	0.28	3.35	0.28
mbi_dp5.a	1.09	0.09	1.09	0.09
mbi_dp5.b1	-1.71	0.16	-1.71	0.16
mbi_dp5.b2	-0.18	0.08	-0.18	0.08
mbi_dp5.b3	0.41	0.09	0.41	0.09
mbi_dp5.b4	0.97	0.10	0.97	0.10
mbi_dp5.b5	1.50	0.13	1.50	0.13
mbi_dp5.b6	2.82	0.23	2.82	0.23
Latent Mean	0.00	NA	0.18	0.07
Latent Variance	1.00	NA	0.79	0.10

Figure 3.29 Differential item and test functioning by specialty group (General Internal Medicine and General Pediatrics) – DP subscale

a) Expected total score functions for reference (General Internal Medicine) and focal (General Pediatrics) groups

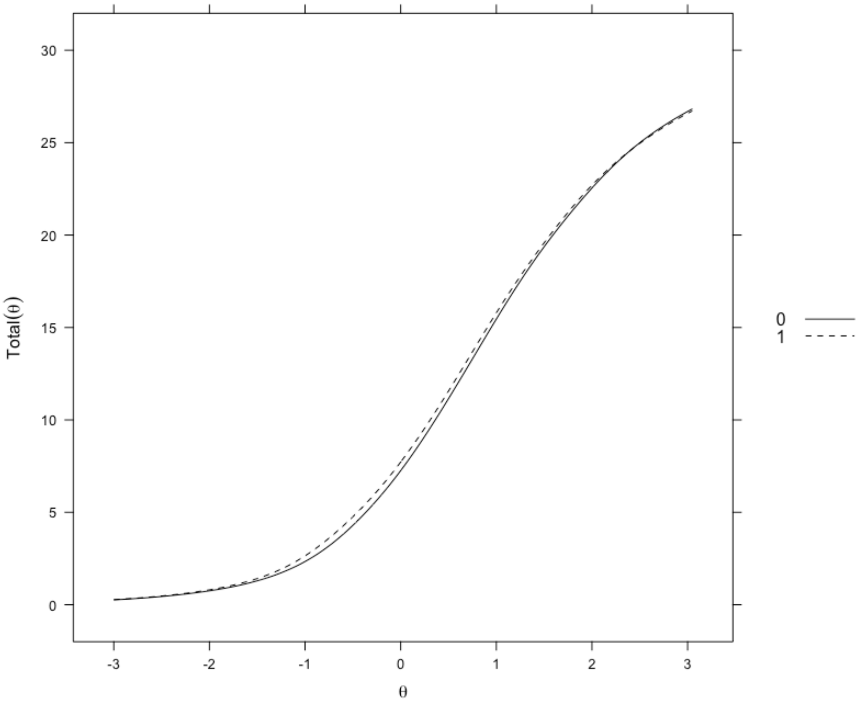

b) Expected item score functions for reference (General Internal Medicine) and focal (General Pediatrics) groups

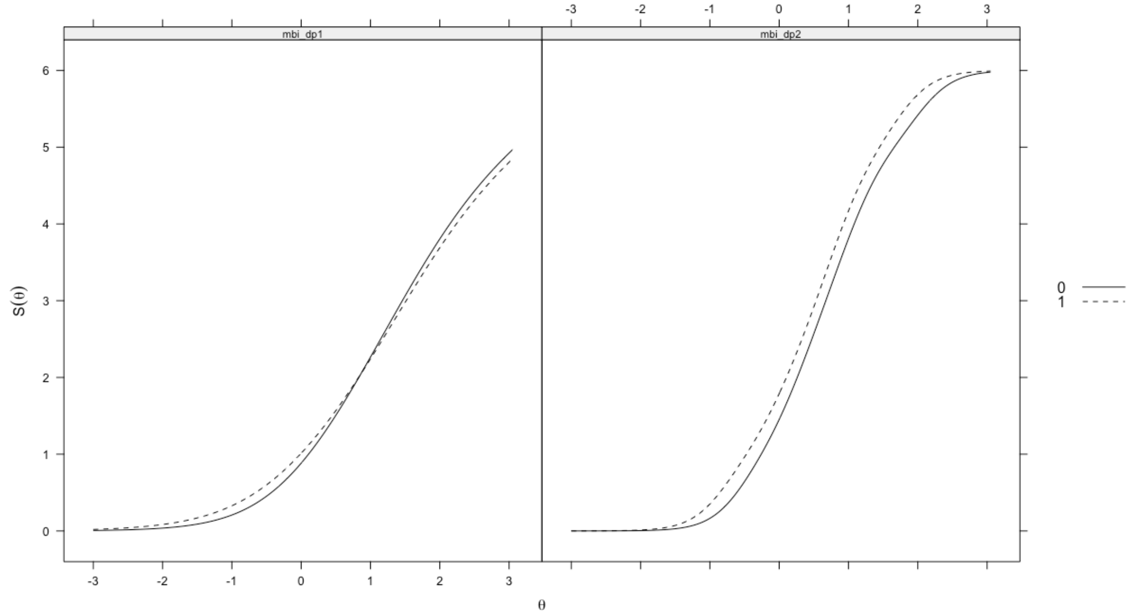

Table 3.29 Multi-group IRT item parameter estimates and standard errors (SE) by specialty group (reference: General Internal Medicine; focal: General Pediatrics) – DP subscale

	**Reference group item parameter estimates**	**Reference group SE**	**Focal group item parameter estimates**	**Focal group SE**
mbi_dp1.a	1.82	0.19	1.48	0.22
mbi_dp1.b1	0.03	0.08	-0.07	0.11
mbi_dp1.b2	0.71	0.09	0.80	0.17
mbi_dp1.b3	1.11	0.11	1.28	0.22
mbi_dp1.b4	1.66	0.15	1.66	0.27
mbi_dp1.b5	2.27	0.20	2.18	0.34
mbi_dp1.b6	3.56	0.41	3.66	0.69
mbi_dp2.a	3.74	0.48	3.73	0.63
mbi_dp2.b1	-0.52	0.07	-0.79	0.10
mbi_dp2.b2	0.12	0.07	-0.07	0.08
mbi_dp2.b3	0.49	0.07	0.39	0.09
mbi_dp2.b4	0.88	0.08	0.73	0.11
mbi_dp2.b5	1.18	0.09	1.00	0.13
mbi_dp2.b6	2.02	0.14	1.74	0.23
mbi_dp3.a	2.44	0.19	2.44	0.19
mbi_dp3.b1	-0.77	0.08	-0.77	0.08

mbi_dp3.b2	-0.07	0.07	-0.07	0.07
mbi_dp3.b3	0.28	0.07	0.28	0.07
mbi_dp3.b4	0.58	0.07	0.58	0.07
mbi_dp3.b5	0.91	0.08	0.91	0.08
mbi_dp3.b6	1.49	0.11	1.49	0.11
mbi_dp4.a	1.55	0.15	1.55	0.15
mbi_dp4.b1	0.40	0.08	0.40	0.08
mbi_dp4.b2	1.12	0.11	1.12	0.11
mbi_dp4.b3	1.60	0.15	1.60	0.15
mbi_dp4.b4	1.98	0.17	1.98	0.17
mbi_dp4.b5	2.61	0.23	2.61	0.23
mbi_dp4.b6	3.48	0.35	3.48	0.35
mbi_dp5.a	1.14	0.10	1.14	0.10
mbi_dp5.b1	-1.60	0.15	-1.60	0.15
mbi_dp5.b2	-0.16	0.09	-0.16	0.09
mbi_dp5.b3	0.43	0.10	0.43	0.10
mbi_dp5.b4	0.99	0.12	0.99	0.12
mbi_dp5.b5	1.55	0.16	1.55	0.16
mbi_dp5.b6	2.87	0.27	2.87	0.27
Latent Mean	0.00	NA	-0.41	0.09
Latent Variance	1.00	NA	0.87	0.15

Figure 3.30 Differential item and test functioning by specialty group (General Internal Medicine and General Surgery) – DP subscale

Brady et al. (2021)

a) Expected total score functions for reference (General Internal Medicine) and focal (General Surgery) groups

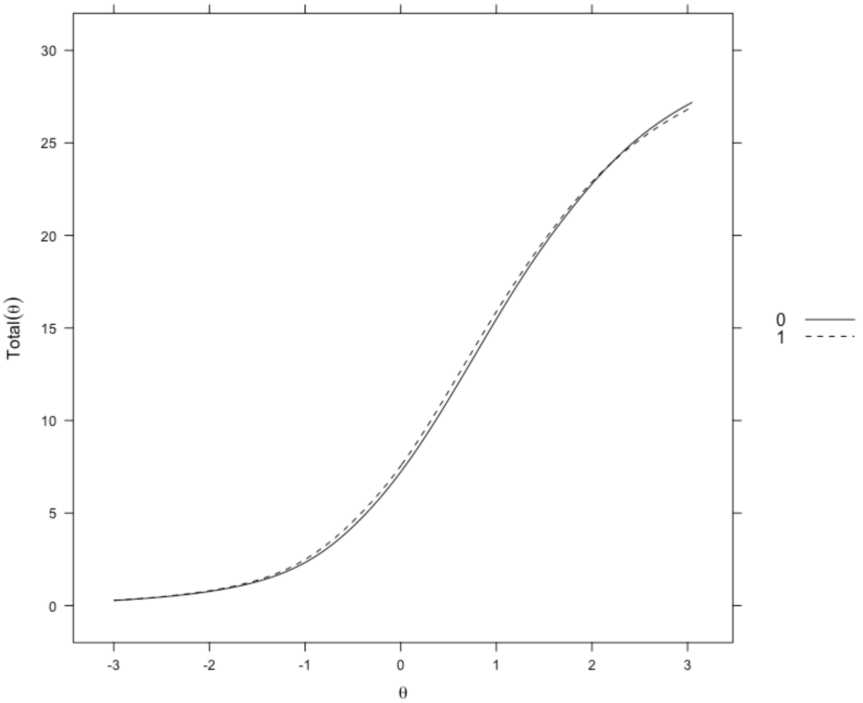

a) Expected item score functions for reference (General Internal Medicine) and focal (General Surgery) groups

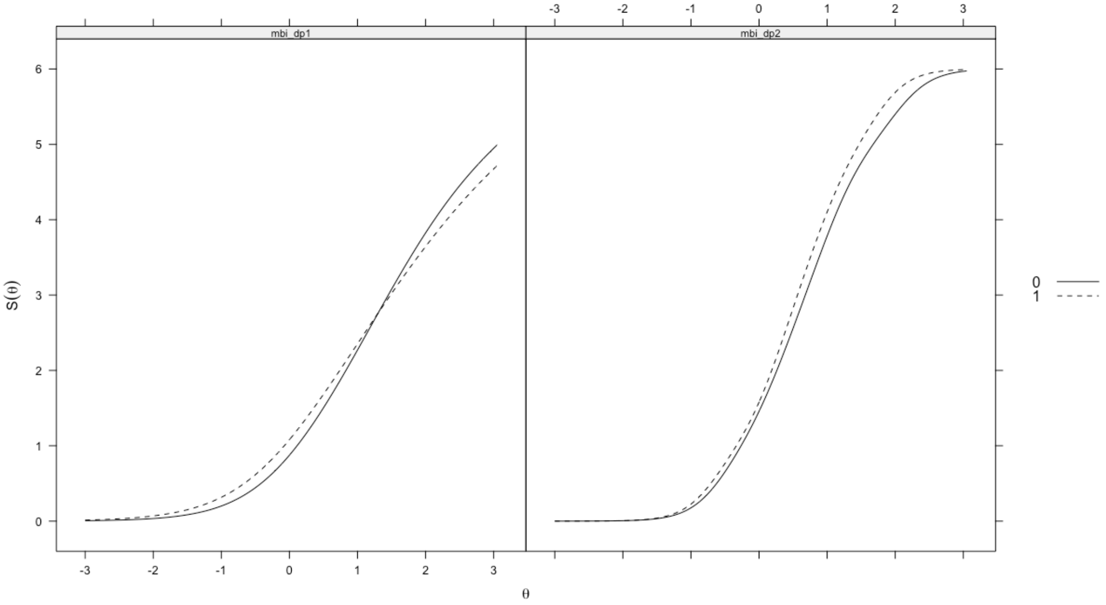

Table 3.30 Multi-group IRT item parameter estimates and standard errors (SE) by specialty group (reference: General Internal Medicine; focal: General Surgery) – DP subscale

Brady et al. (2021)

	**Reference group item parameter estimates**	**Reference group SE**	**Focal group item parameter estimates**	**Focal group SE**
mbi_dp1.a	1.84	0.19	1.64	0.27
mbi_dp1.b1	0.04	0.08	-0.10	0.12
mbi_dp1.b2	0.70	0.09	0.49	0.13
mbi_dp1.b3	1.10	0.11	1.01	0.17
mbi_dp1.b4	1.65	0.14	1.77	0.26
mbi_dp1.b5	2.26	0.20	2.56	0.39
mbi_dp1.b6	3.53	0.41	3.93	0.80
mbi_dp2.a	3.61	0.44	3.82	0.72
mbi_dp2.b1	-0.53	0.07	-0.65	0.11
mbi_dp2.b2	0.13	0.07	0.13	0.09
mbi_dp2.b3	0.49	0.07	0.37	0.09
mbi_dp2.b4	0.88	0.08	0.72	0.11
mbi_dp2.b5	1.18	0.09	1.08	0.13
mbi_dp2.b6	2.03	0.14	1.74	0.20
mbi_dp3.a	2.47	0.20	2.47	0.20
mbi_dp3.b1	-0.77	0.08	-0.77	0.08
mbi_dp3.b2	-0.06	0.07	-0.06	0.07
mbi_dp3.b3	0.28	0.07	0.28	0.07
mbi_dp3.b4	0.57	0.07	0.57	0.07
mbi_dp3.b5	0.92	0.08	0.92	0.08
mbi_dp3.b6	1.52	0.11	1.52	0.11
mbi_dp4.a	1.73	0.17	1.73	0.17
mbi_dp4.b1	0.38	0.08	0.38	0.08
mbi_dp4.b2	1.08	0.11	1.08	0.11
mbi_dp4.b3	1.61	0.14	1.61	0.14
mbi_dp4.b4	1.91	0.16	1.91	0.16
mbi_dp4.b5	2.41	0.20	2.41	0.20
mbi_dp4.b6	3.14	0.29	3.14	0.29
mbi_dp5.a	1.15	0.11	1.15	0.11
mbi_dp5.b1	-1.73	0.17	-1.73	0.17
mbi_dp5.b2	-0.18	0.09	-0.18	0.09
mbi_dp5.b3	0.43	0.10	0.43	0.10
mbi_dp5.b4	0.96	0.12	0.96	0.12
mbi_dp5.b5	1.56	0.16	1.56	0.16

Brady et al. (2021)

mbi_dp5.b6	2.69	0.25	2.69	0.25
Latent Mean	0.00	NA	-0.05	0.09
Latent Variance	1.00	NA	0.83	0.15

Figure 3.31 Differential item and test functioning by specialty group (General Internal Medicine and General Surgery Subspecialty) – DP subscale

b) Expected total score functions for reference (General Internal Medicine) and focal (General Surgery Subspecialty) groups

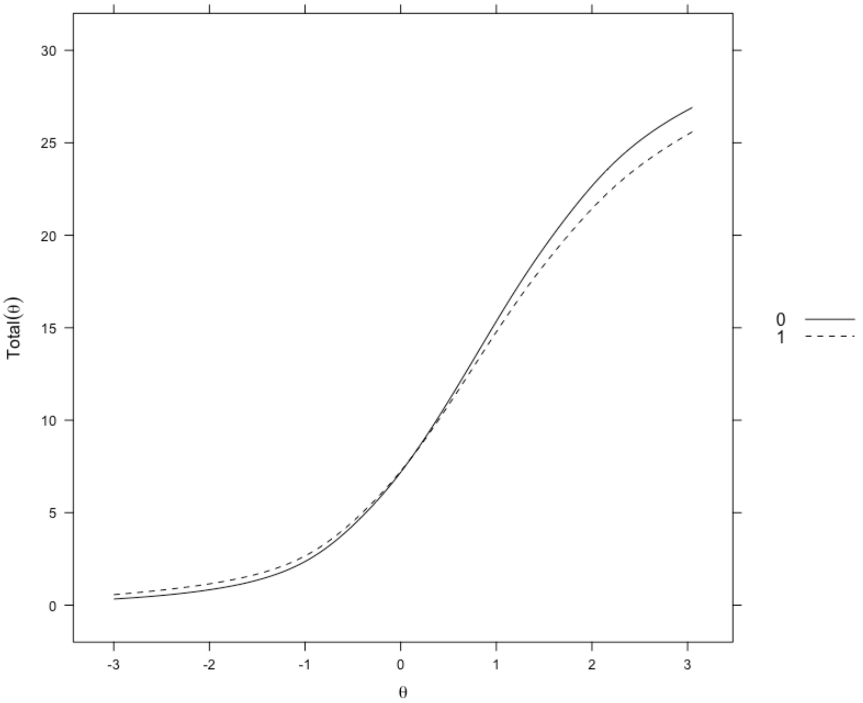

b) Expected item score functions for reference (General Internal Medicine) and focal (General Surgery Subspecialty) groups

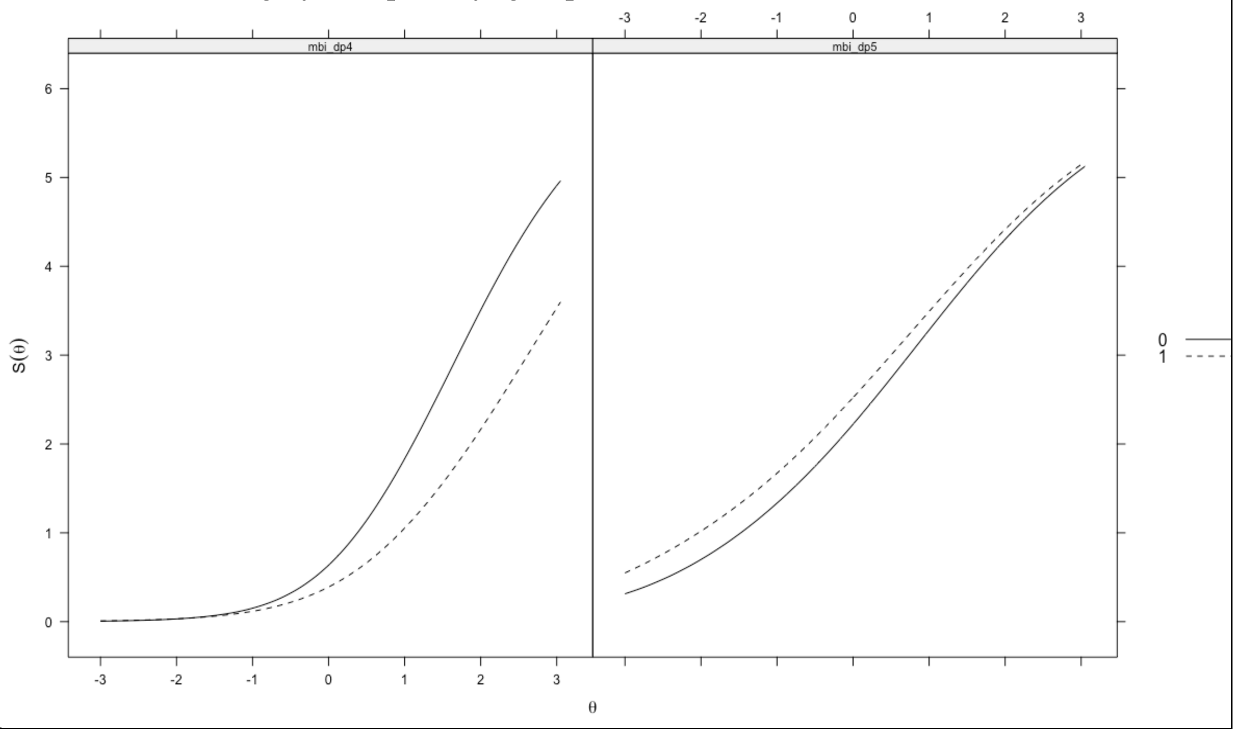

Table 3.31 Multi-group IRT item parameter estimates and standard errors (SE) by specialty group (reference: General Internal Medicine; focal: General Surgery Subspecialty) – DP subscale

	Reference group item parameter estimates	Reference group SE	Focal group item parameter estimates	Focal group SE
mbi_dp1.a	1.76	0.15	1.76	0.15
mbi_dp1.b1	0.08	0.07	0.08	0.07
mbi_dp1.b2	0.74	0.08	0.74	0.08
mbi_dp1.b3	1.20	0.10	1.20	0.10
mbi_dp1.b4	1.71	0.13	1.71	0.13
mbi_dp1.b5	2.32	0.18	2.32	0.18
mbi_dp1.b6	3.73	0.38	3.73	0.38
mbi_dp2.a	4.03	0.46	4.03	0.46
mbi_dp2.b1	-0.58	0.07	-0.58	0.07
mbi_dp2.b2	0.13	0.06	0.13	0.06
mbi_dp2.b3	0.49	0.06	0.49	0.06
mbi_dp2.b4	0.86	0.07	0.86	0.07
mbi_dp2.b5	1.18	0.08	1.18	0.08
mbi_dp2.b6	1.90	0.12	1.90	0.12

mbi_dp3.a	2.36	0.18	2.36	0.18
mbi_dp3.b1	-0.67	0.08	-0.67	0.08
mbi_dp3.b2	-0.08	0.06	-0.08	0.06
mbi_dp3.b3	0.26	0.07	0.26	0.07
mbi_dp3.b4	0.61	0.07	0.61	0.07
mbi_dp3.b5	0.96	0.08	0.96	0.08
mbi_dp3.b6	1.57	0.11	1.57	0.11
mbi_dp4.a	1.65	0.18	1.34	0.21
mbi_dp4.b1	0.41	0.09	0.88	0.15
mbi_dp4.b2	1.04	0.11	1.79	0.25
mbi_dp4.b3	1.52	0.15	2.42	0.34
mbi_dp4.b4	1.85	0.17	2.91	0.41
mbi_dp4.b5	2.32	0.22	3.35	0.50
mbi_dp4.b6	3.21	0.34	4.31	0.76
mbi_dp5.a	1.04	0.12	1.00	0.14
mbi_dp5.b1	-1.74	0.21	-2.59	0.35
mbi_dp5.b2	-0.16	0.11	-0.56	0.14
mbi_dp5.b3	0.49	0.12	0.14	0.13
mbi_dp5.b4	1.06	0.15	0.96	0.18
mbi_dp5.b5	1.66	0.20	1.69	0.26
mbi_dp5.b6	2.91	0.33	2.77	0.40
Latent Mean	0.00	NA	-0.16	0.08
Latent Variance	1.00	NA	0.93	0.13

Figure 3.32 Differential item and test functioning by specialty group (General Internal Medicine and Internal Medicine Subspecialty) – DP subscale

a) Expected total score functions for reference (General Internal Medicine) and focal (Internal Medicine Subspecialty) groups

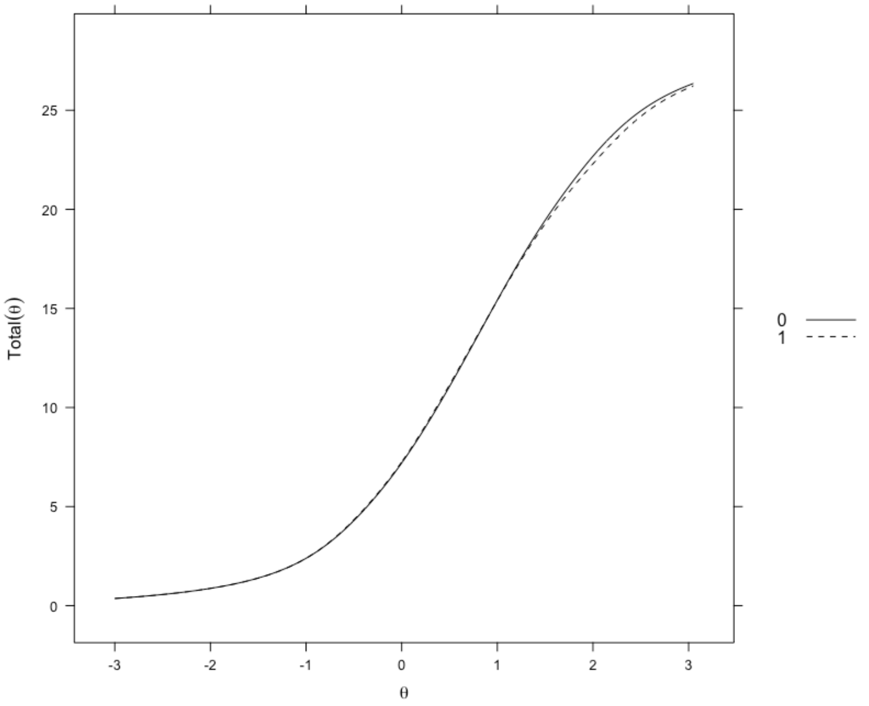

b) Expected item score functions for reference (General Internal Medicine) and focal (Internal Medicine Subspecialty) groups

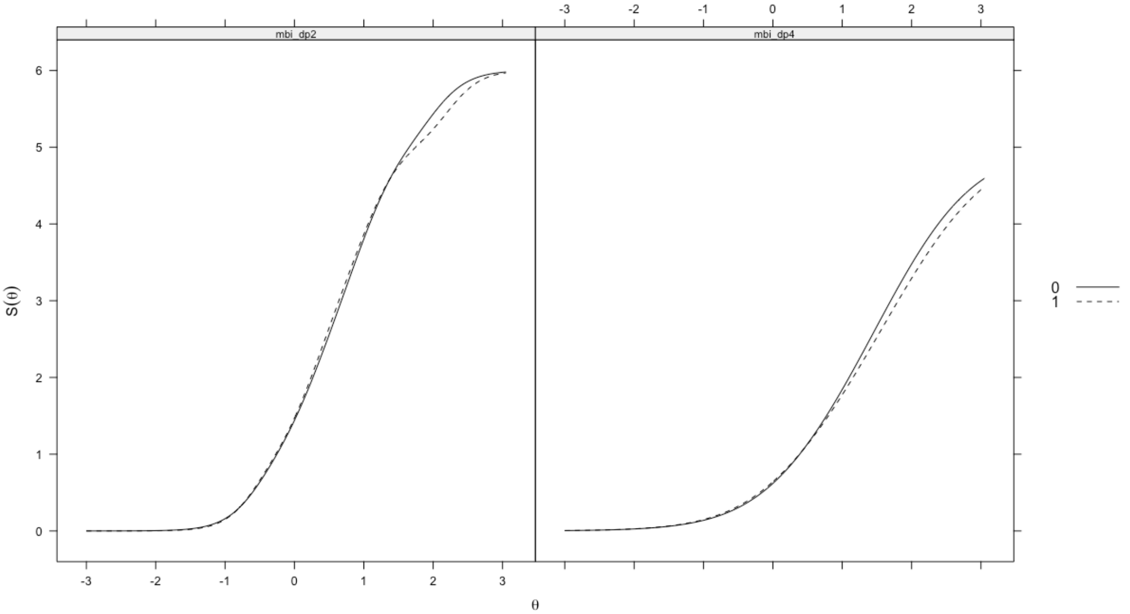

Brady et al. (2021)

Table 3.32 Multi-group IRT item parameter estimates and standard errors (SE) by specialty group (reference: General Internal Medicine; focal: Internal Medicine Subspecialty) – DP subscale

	**Reference group item parameter estimates**	**Reference group SE**	**Focal group item parameter estimates**	**Focal group SE**
mbi_dp1.a	1.90	0.14	1.90	0.14
mbi_dp1.b1	-0.07	0.07	-0.07	0.07
mbi_dp1.b2	0.65	0.07	0.65	0.07
mbi_dp1.b3	1.04	0.09	1.04	0.09
mbi_dp1.b4	1.49	0.11	1.49	0.11
mbi_dp1.b5	1.97	0.14	1.97	0.14
mbi_dp2.a	3.77	0.48	4.20	0.52
mbi_dp2.b1	-0.52	0.07	-0.56	0.08
mbi_dp2.b2	0.13	0.07	0.12	0.07
mbi_dp2.b3	0.49	0.07	0.42	0.07
mbi_dp2.b4	0.88	0.08	0.84	0.09
mbi_dp2.b5	1.18	0.09	1.20	0.11
mbi_dp2.b6	2.01	0.14	2.23	0.20
mbi_dp3.a	2.37	0.16	2.37	0.16
mbi_dp3.b1	-0.72	0.08	-0.72	0.08
mbi_dp3.b2	-0.03	0.06	-0.03	0.06
mbi_dp3.b3	0.31	0.06	0.31	0.06
mbi_dp3.b4	0.67	0.07	0.67	0.07
mbi_dp3.b5	1.03	0.08	1.03	0.08
mbi_dp3.b6	1.60	0.11	1.60	0.11
mbi_dp4.a	1.72	0.19	1.76	0.18
mbi_dp4.b1	0.40	0.09	0.24	0.08
mbi_dp4.b2	1.01	0.11	1.03	0.12
mbi_dp4.b3	1.48	0.14	1.55	0.15
mbi_dp4.b4	1.80	0.17	1.95	0.19
mbi_dp4.b5	2.24	0.21	2.54	0.25
mbi_dp5.a	1.01	0.09	1.01	0.09
mbi_dp5.b1	-1.83	0.16	-1.83	0.16
mbi_dp5.b2	-0.23	0.08	-0.23	0.08
mbi_dp5.b3	0.51	0.09	0.51	0.09
mbi_dp5.b4	1.14	0.12	1.14	0.12

Brady et al. (2021)

mbi_dp5.b5	1.77	0.16	1.77	0.16
mbi_dp5.b6	3.14	0.27	3.14	0.27
Latent Mean	0.00	NA	-0.20	0.07
Latent Variance	1.00	NA	0.96	0.13

Figure 3.33 Differential item and test functioning by specialty group (General Internal Medicine and Neurology) – DP subscale

a) Expected total score functions for reference (General Internal Medicine) and focal (Neurology) groups

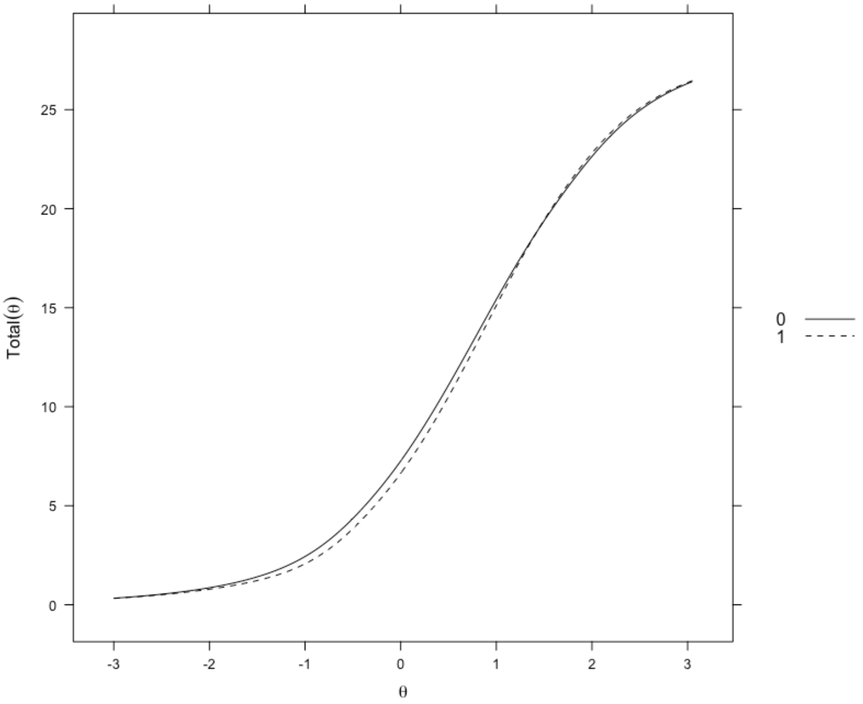

b) Expected item score functions for reference (General Internal Medicine) and focal (Neurology) groups

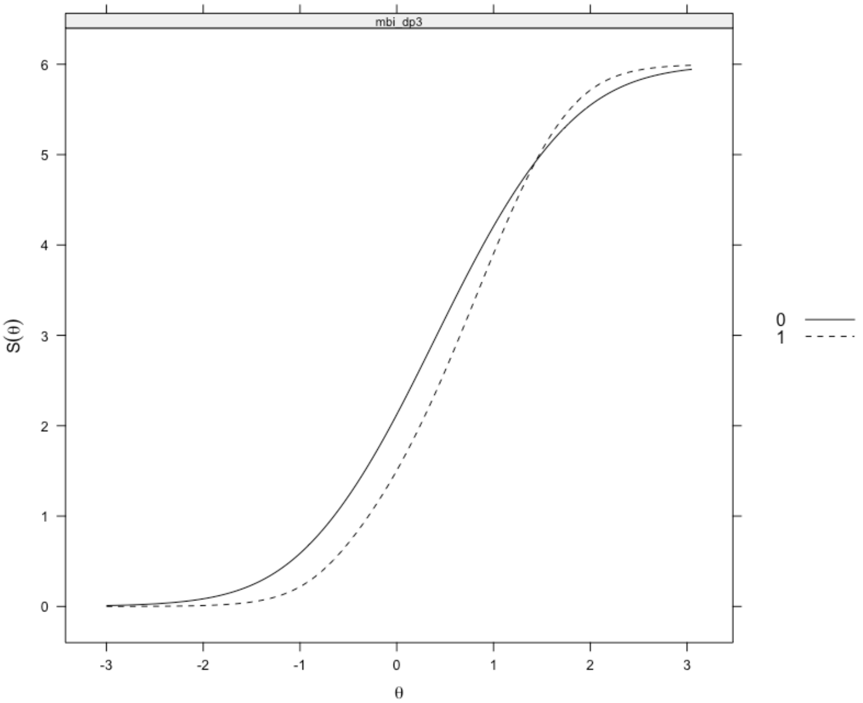

Table 3.33 Multi-group IRT item parameter estimates and standard errors (SE) by specialty group (reference: General Internal Medicine; focal: Neurology) – DP subscale

	**Reference group item parameter estimates**	**Reference group SE**	**Focal group item parameter estimates**	**Focal group SE**
mbi_dp1.a	1.92	0.17	1.92	0.17
mbi_dp1.b1	0.00	0.07	0.00	0.07
mbi_dp1.b2	0.72	0.08	0.72	0.08
mbi_dp1.b3	1.10	0.10	1.10	0.10
mbi_dp1.b4	1.60	0.12	1.60	0.12
mbi_dp1.b5	2.12	0.16	2.12	0.16
mbi_dp2.a	3.83	0.40	3.83	0.40
mbi_dp2.b1	-0.51	0.07	-0.51	0.07
mbi_dp2.b2	0.16	0.06	0.16	0.06
mbi_dp2.b3	0.53	0.06	0.53	0.06
mbi_dp2.b4	0.88	0.07	0.88	0.07
mbi_dp2.b5	1.16	0.08	1.16	0.08
mbi_dp2.b6	1.95	0.12	1.95	0.12
mbi_dp3.a	2.22	0.20	3.24	0.45

mbi_dp3.b1	-0.74	0.09	-0.53	0.10
mbi_dp3.b2	-0.10	0.07	0.11	0.08
mbi_dp3.b3	0.23	0.07	0.51	0.09
mbi_dp3.b4	0.57	0.08	0.87	0.10
mbi_dp3.b5	0.94	0.09	1.13	0.12
mbi_dp3.b6	1.60	0.13	1.56	0.15
mbi_dp4.a	1.71	0.16	1.71	0.16
mbi_dp4.b1	0.39	0.08	0.39	0.08
mbi_dp4.b2	1.12	0.10	1.12	0.10
mbi_dp4.b3	1.59	0.13	1.59	0.13
mbi_dp4.b4	1.91	0.15	1.91	0.15
mbi_dp4.b5	2.33	0.19	2.33	0.19
mbi_dp5.a	1.10	0.10	1.10	0.10
mbi_dp5.b1	-1.83	0.18	-1.83	0.18
mbi_dp5.b2	-0.28	0.10	-0.28	0.10
mbi_dp5.b3	0.38	0.10	0.38	0.10
mbi_dp5.b4	0.97	0.12	0.97	0.12
mbi_dp5.b5	1.64	0.16	1.64	0.16
mbi_dp5.b6	2.87	0.27	2.87	0.27
Latent Mean	0.00	NA	0.10	0.09
Latent Variance	1.00	NA	0.87	0.14

Figure 3.34 Differential item and test functioning by specialty group (General Internal Medicine and Obstetrics and Gynecology) – DP subscale

a) Expected total score functions for reference (General Internal Medicine) and focal (Obstetrics and Gynecology) groups

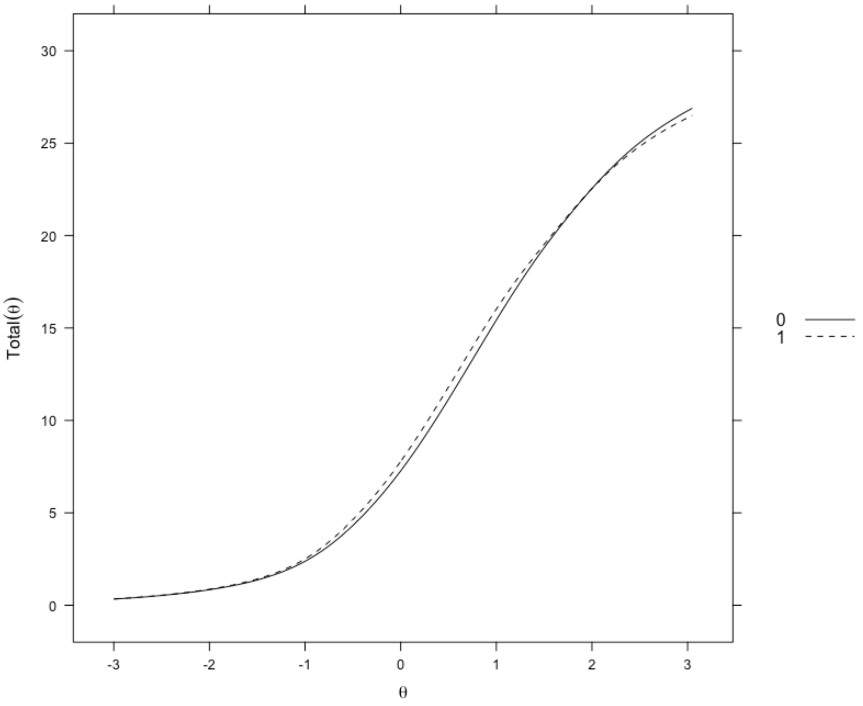

b) Expected item score functions for reference (General Internal Medicine) and focal (Obstetrics and Gynecology) groups

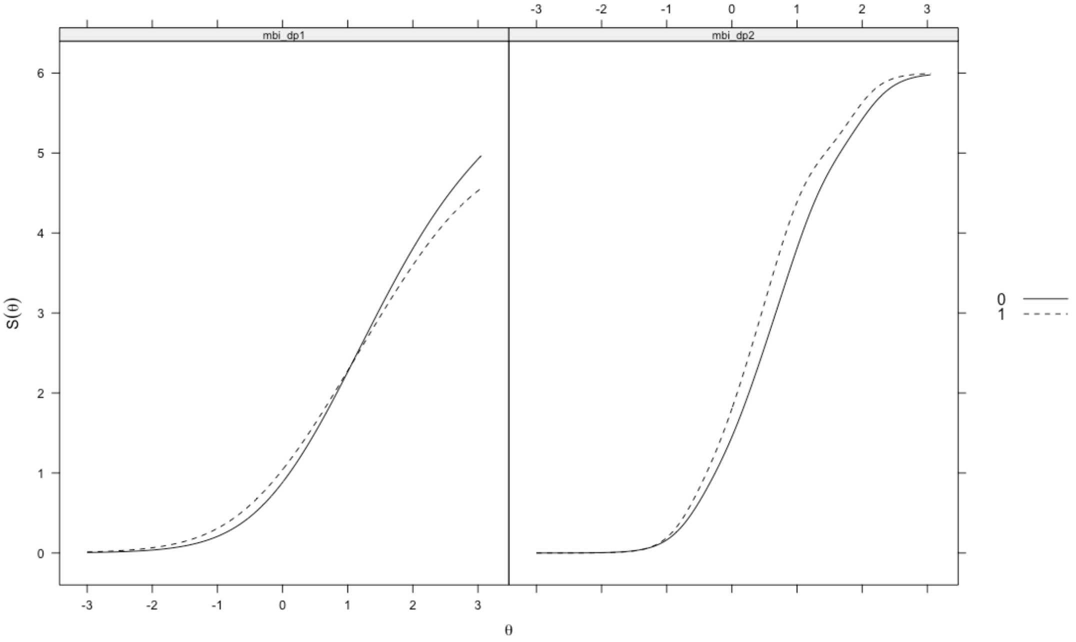

Brady et al. (2021)

Table 3.34 Multi-group IRT item parameter estimates and standard errors (SE) by specialty group (reference: General Internal Medicine; focal: Obstetrics and Gynecology) – DP subscale

	**Reference group item parameter estimates**	**Reference group SE**	**Focal group item parameter estimates**	**Focal group SE**
mbi_dp1.a	1.82	0.19	1.68	0.25
mbi_dp1.b1	0.04	0.08	-0.19	0.11
mbi_dp1.b2	0.71	0.09	0.60	0.13
mbi_dp1.b3	1.11	0.11	1.11	0.17
mbi_dp1.b4	1.66	0.15	1.81	0.26
mbi_dp1.b5	2.27	0.20	2.47	0.36
mbi_dp1.b6	3.56	0.41	8.67	30.74
mbi_dp2.a	3.74	0.48	4.30	0.83
mbi_dp2.b1	-0.52	0.07	-0.63	0.10
mbi_dp2.b2	0.12	0.07	-0.06	0.08
mbi_dp2.b3	0.49	0.07	0.29	0.09
mbi_dp2.b4	0.88	0.08	0.64	0.10
mbi_dp2.b5	1.18	0.09	0.91	0.12
mbi_dp2.b6	2.02	0.14	1.86	0.22
mbi_dp3.a	2.51	0.20	2.51	0.20
mbi_dp3.b1	-0.73	0.08	-0.73	0.08
mbi_dp3.b2	-0.09	0.07	-0.09	0.07
mbi_dp3.b3	0.26	0.07	0.26	0.07
mbi_dp3.b4	0.55	0.07	0.55	0.07
mbi_dp3.b5	0.91	0.08	0.91	0.08
mbi_dp3.b6	1.48	0.11	1.48	0.11
mbi_dp4.a	1.62	0.16	1.62	0.16
mbi_dp4.b1	0.44	0.08	0.44	0.08
mbi_dp4.b2	1.16	0.11	1.16	0.11
mbi_dp4.b3	1.67	0.15	1.67	0.15
mbi_dp4.b4	2.03	0.18	2.03	0.18
mbi_dp4.b5	2.45	0.21	2.45	0.21
mbi_dp4.b6	3.35	0.33	3.35	0.33
mbi_dp5.a	1.07	0.10	1.07	0.10
mbi_dp5.b1	-1.82	0.18	-1.82	0.18
mbi_dp5.b2	-0.22	0.09	-0.22	0.09
mbi_dp5.b3	0.43	0.10	0.43	0.10

Brady et al. (2021)

mbi_dp5.b4	1.02	0.13	1.02	0.13
mbi_dp5.b5	1.64	0.17	1.64	0.17
mbi_dp5.b6	2.84	0.27	2.84	0.27
Latent Mean	0.00	NA	-0.11	0.09
Latent Variance	1.00	NA	0.82	0.15

Figure 3.35 Differential item and test functioning by specialty group (General Internal Medicine and Ophthalmology) – DP subscale

a) Expected total score functions for reference (General Internal Medicine) and focal (Ophthalmology) groups

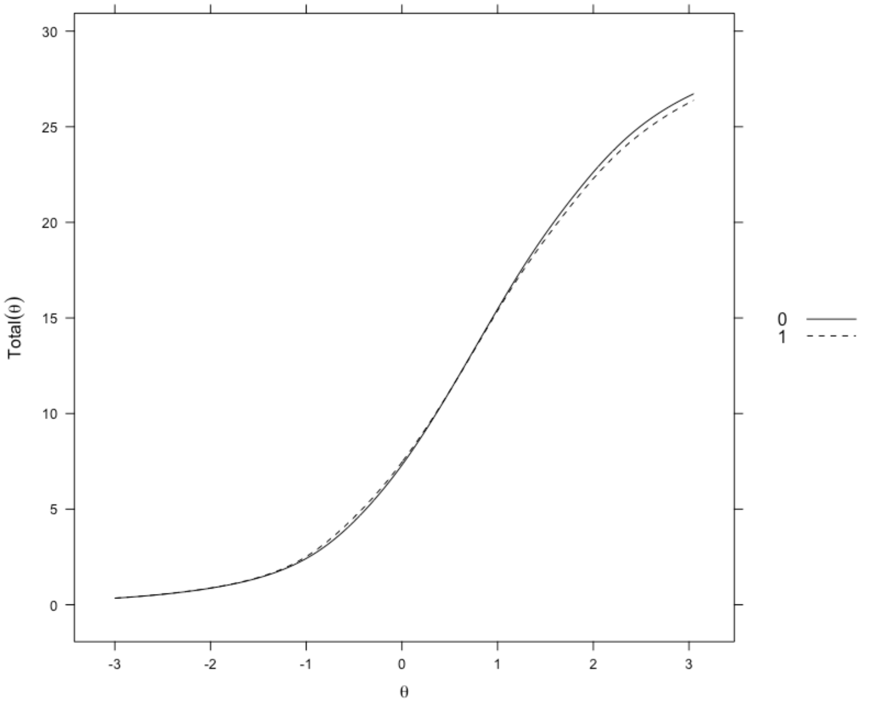

b) Expected item score functions for reference (General Internal Medicine) and focal (Ophthalmology) groups

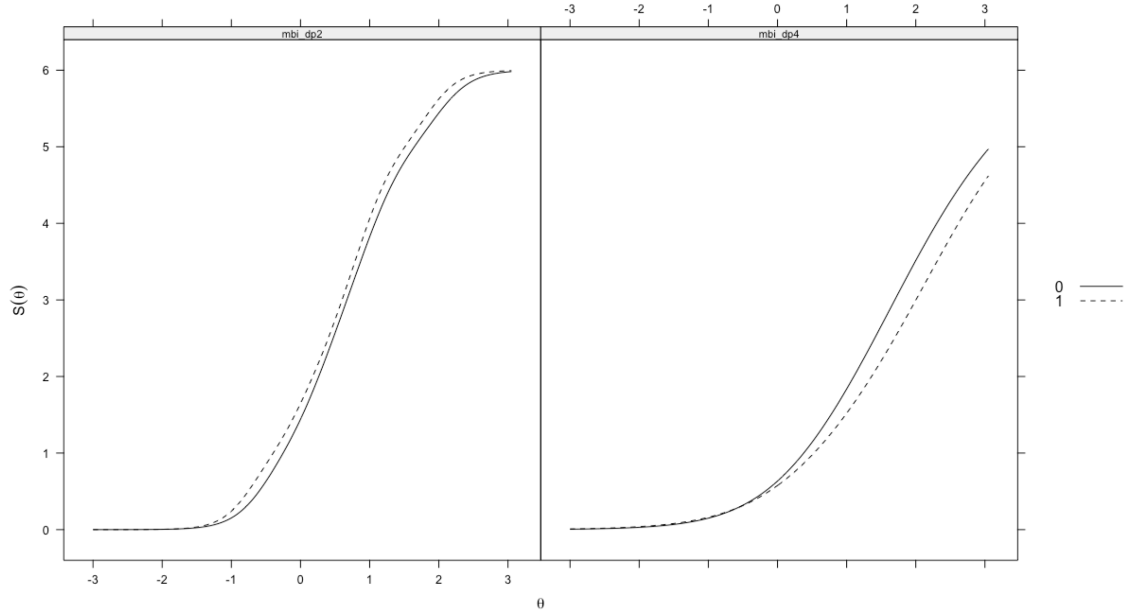

Table 3.35 Multi-group IRT item parameter estimates and standard errors (SE) by specialty group (reference: General Internal Medicine; focal: Ophthalmology) – DP subscale

	**Reference group item parameter estimates**	**Reference group SE**	**Focal group item parameter estimates**	**Focal group SE**
mbi_dp1.a	1.69	0.15	1.69	0.15
mbi_dp1.b1	-0.04	0.08	-0.04	0.08
mbi_dp1.b2	0.66	0.09	0.66	0.09
mbi_dp1.b3	1.13	0.11	1.13	0.11
mbi_dp1.b4	1.69	0.14	1.69	0.14
mbi_dp1.b5	2.15	0.17	2.15	0.17
mbi_dp2.a	3.86	0.52	4.33	0.99
mbi_dp2.b1	-0.52	0.07	-0.71	0.11
mbi_dp2.b2	0.12	0.06	-0.03	0.09
mbi_dp2.b3	0.48	0.07	0.46	0.10
mbi_dp2.b4	0.87	0.08	0.81	0.12
mbi_dp2.b5	1.17	0.09	1.05	0.13
mbi_dp2.b6	2.00	0.14	1.86	0.21
mbi_dp3.a	2.35	0.19	2.35	0.19
mbi_dp3.b1	-0.67	0.08	-0.67	0.08
mbi_dp3.b2	-0.05	0.07	-0.05	0.07

mbi_dp3.b3	0.29	0.07	0.29	0.07
mbi_dp3.b4	0.61	0.07	0.61	0.07
mbi_dp3.b5	0.94	0.08	0.94	0.08
mbi_dp3.b6	1.58	0.11	1.58	0.11
mbi_dp4.a	1.66	0.19	1.49	0.24
mbi_dp4.b1	0.41	0.09	0.42	0.14
mbi_dp4.b2	1.04	0.11	1.37	0.22
mbi_dp4.b3	1.51	0.15	1.95	0.29
mbi_dp4.b4	1.84	0.17	2.23	0.33
mbi_dp4.b5	2.31	0.22	2.60	0.38
mbi_dp4.b6	3.21	0.35	3.20	0.49
mbi_dp5.a	1.07	0.10	1.07	0.10
mbi_dp5.b1	-1.85	0.18	-1.85	0.18
mbi_dp5.b2	-0.23	0.10	-0.23	0.10
mbi_dp5.b3	0.42	0.10	0.42	0.10
mbi_dp5.b4	1.09	0.13	1.09	0.13
mbi_dp5.b5	1.68	0.17	1.68	0.17
mbi_dp5.b6	2.94	0.28	2.94	0.28
Latent Mean	0.00	NA	-0.21	0.11
Latent Variance	1.00	NA	1.28	0.23

Figure 3.36 Differential item and test functioning by specialty group (General Internal Medicine and Orthopedic Surgery) – DP subscale

a) Expected total score functions for reference (General Internal Medicine) and focal (Orthopedic Surgery) groups

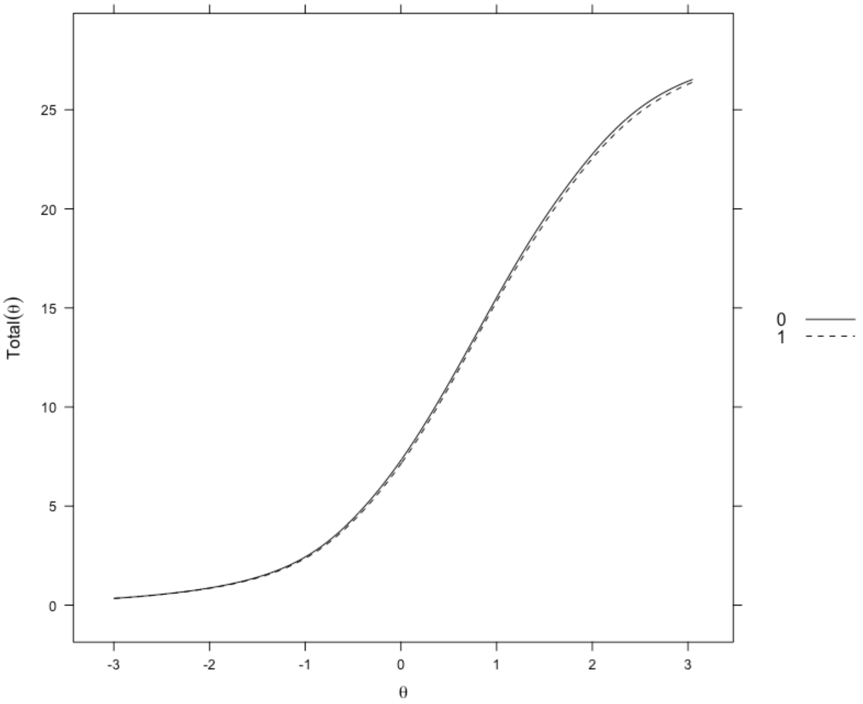

b) Expected item score functions for reference (General Internal Medicine) and focal (Orthopedic Surgery) groups

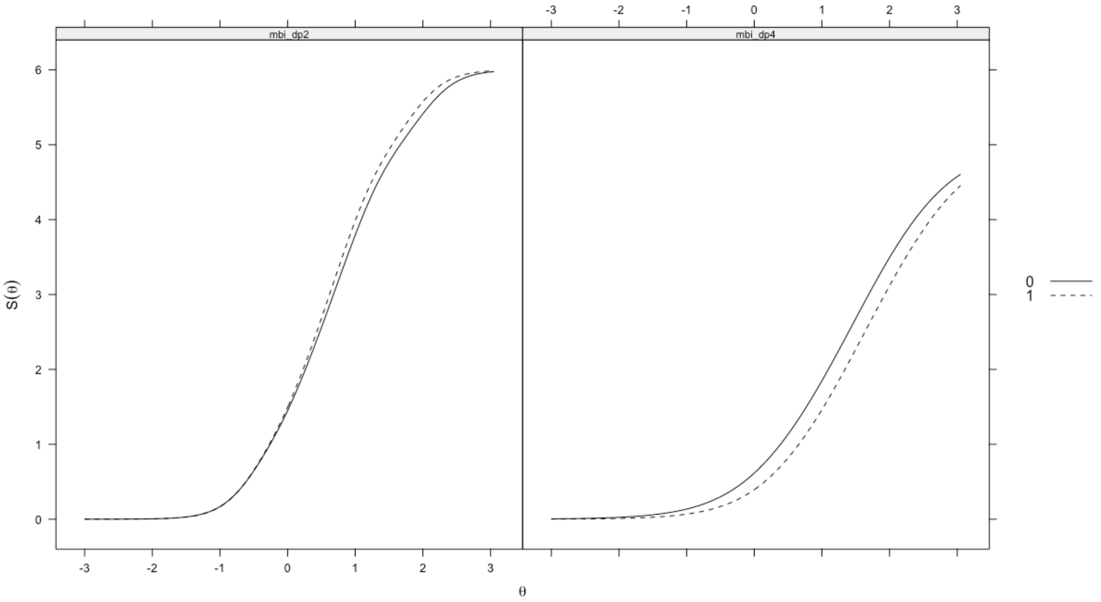

Table 3.36 Multi-group IRT item parameter estimates and standard errors (SE) by specialty group (reference: General Internal Medicine; focal: Orthopedic Surgery) – DP subscale

	Reference group item parameter estimates	Reference group SE	Focal group item parameter estimates	Focal group SE
mbi_dp1.a	1.91	0.17	1.91	0.17
mbi_dp1.b1	0.01	0.07	0.01	0.07
mbi_dp1.b2	0.70	0.08	0.70	0.08
mbi_dp1.b3	1.10	0.10	1.10	0.10
mbi_dp1.b4	1.62	0.12	1.62	0.12
mbi_dp1.b5	2.17	0.16	2.17	0.16
mbi_dp2.a	3.69	0.48	3.72	0.70
mbi_dp2.b1	-0.52	0.07	-0.52	0.11
mbi_dp2.b2	0.13	0.07	0.11	0.09
mbi_dp2.b3	0.49	0.07	0.46	0.10
mbi_dp2.b4	0.88	0.08	0.80	0.11
mbi_dp2.b5	1.18	0.09	1.08	0.12
mbi_dp2.b6	2.02	0.14	1.87	0.19
mbi_dp3.a	2.30	0.19	2.30	0.19
mbi_dp3.b1	-0.70	0.08	-0.70	0.08
mbi_dp3.b2	-0.08	0.07	-0.08	0.07

mbi_dp3.b3	0.28	0.07	0.28	0.07
mbi_dp3.b4	0.63	0.07	0.63	0.07
mbi_dp3.b5	0.97	0.08	0.97	0.08
mbi_dp3.b6	1.64	0.12	1.64	0.12
mbi_dp4.a	1.74	0.20	1.93	0.32
mbi_dp4.b1	0.40	0.09	0.58	0.12
mbi_dp4.b2	1.01	0.11	1.26	0.17
mbi_dp4.b3	1.47	0.14	1.59	0.20
mbi_dp4.b4	1.79	0.17	2.08	0.26
mbi_dp4.b5	2.24	0.21	2.66	0.35
mbi_dp5.a	1.07	0.10	1.07	0.10
mbi_dp5.b1	-1.84	0.19	-1.84	0.19
mbi_dp5.b2	-0.32	0.10	-0.32	0.10
mbi_dp5.b3	0.38	0.10	0.38	0.10
mbi_dp5.b4	0.99	0.12	0.99	0.12
mbi_dp5.b5	1.49	0.15	1.49	0.15
mbi_dp5.b6	2.67	0.25	2.67	0.25
Latent Mean	0.00	NA	0.13	0.10
Latent Variance	1.00	NA	0.98	0.17

Figure 3.37 Differential item and test functioning by specialty group (General Internal Medicine and Pediatric Subspecialty) – DP subscale

a) Expected total score functions for reference (General Internal Medicine) and focal (Pediatric Subspecialty) groups

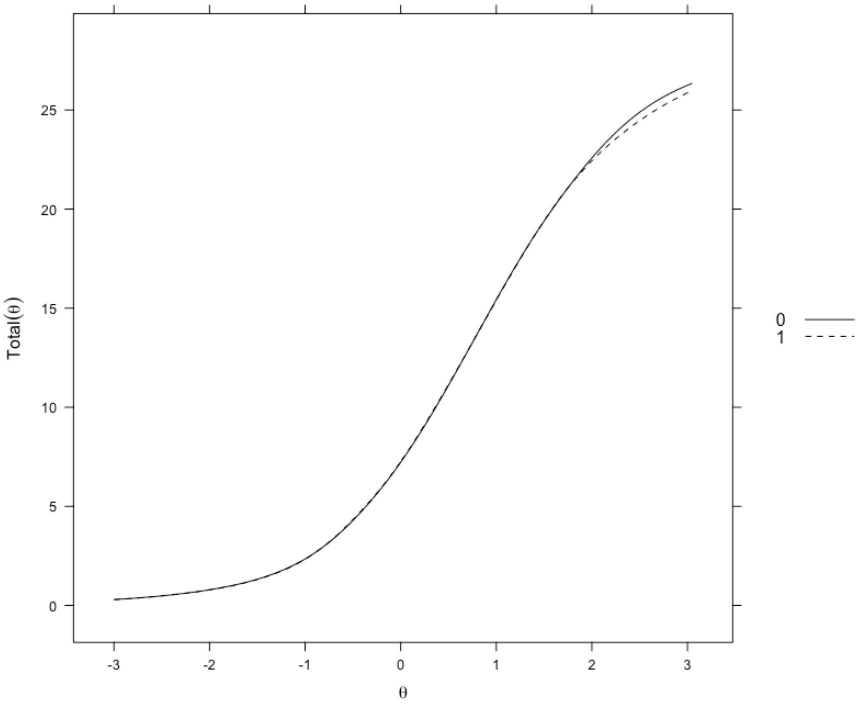

b) Expected item score functions for reference (General Internal Medicine) and focal (Pediatric Subspecialty) groups

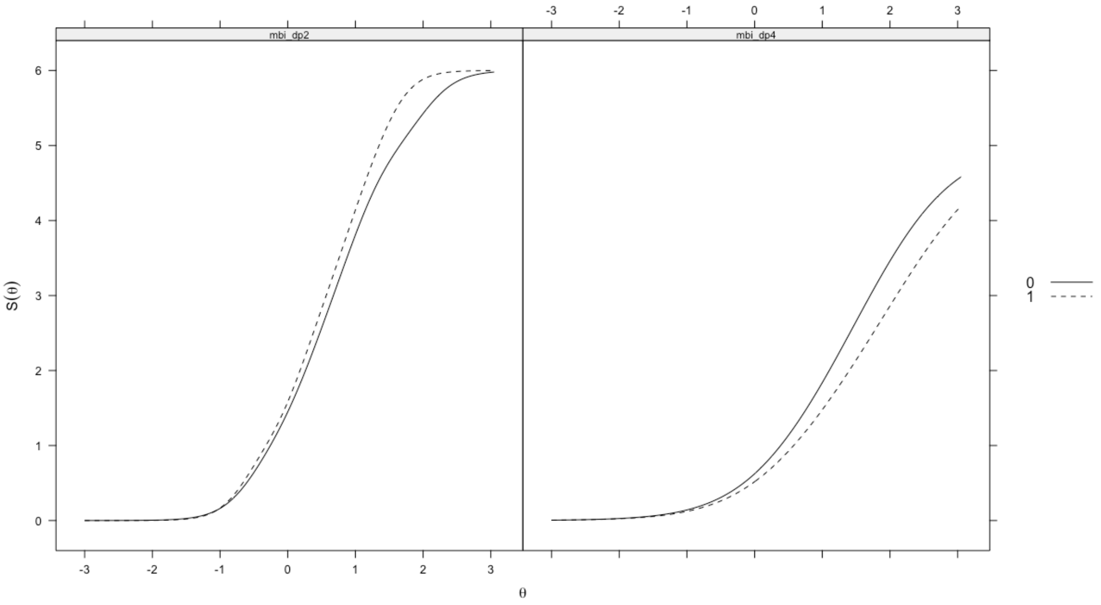

Brady et al. (2021)

Table 3.37 Multi-group IRT item parameter estimates and standard errors (SE) by specialty group (reference: General Internal Medicine; focal: Pediatric Subspecialty) – DP subscale

	**Reference group item parameter estimates**	**Reference group SE**	**Focal group item parameter estimates**	**Focal group SE**
mbi_dp1.a	1.73	0.15	1.73	0.15
mbi_dp1.b1	-0.09	0.07	-0.09	0.07
mbi_dp1.b2	0.66	0.08	0.66	0.08
mbi_dp1.b3	1.10	0.10	1.10	0.10
mbi_dp1.b4	1.67	0.13	1.67	0.13
mbi_dp1.b5	2.20	0.18	2.20	0.18
mbi_dp2.a	3.76	0.48	4.47	0.86
mbi_dp2.b1	-0.52	0.07	-0.62	0.09
mbi_dp2.b2	0.12	0.07	0.06	0.08
mbi_dp2.b3	0.49	0.07	0.36	0.09
mbi_dp2.b4	0.88	0.08	0.77	0.11
mbi_dp2.b5	1.18	0.09	1.11	0.14
mbi_dp2.b6	2.01	0.14	1.50	0.19
mbi_dp3.a	2.43	0.19	2.43	0.19
mbi_dp3.b1	-0.72	0.08	-0.72	0.08
mbi_dp3.b2	-0.03	0.07	-0.03	0.07
mbi_dp3.b3	0.29	0.07	0.29	0.07
mbi_dp3.b4	0.63	0.07	0.63	0.07
mbi_dp3.b5	0.96	0.08	0.96	0.08
mbi_dp3.b6	1.54	0.11	1.54	0.11
mbi_dp4.a	1.70	0.19	1.67	0.27
mbi_dp4.b1	0.40	0.09	0.43	0.13
mbi_dp4.b2	1.02	0.11	1.23	0.21
mbi_dp4.b3	1.48	0.14	1.79	0.29
mbi_dp4.b4	1.81	0.17	2.36	0.39
mbi_dp4.b5	2.26	0.21	2.81	0.49
mbi_dp5.a	1.09	0.10	1.09	0.10
mbi_dp5.b1	-1.67	0.16	-1.67	0.16
mbi_dp5.b2	-0.19	0.09	-0.19	0.09
mbi_dp5.b3	0.48	0.10	0.48	0.10
mbi_dp5.b4	1.10	0.13	1.10	0.13
mbi_dp5.b5	1.72	0.18	1.72	0.18

Brady et al. (2021)

mbi_dp5.b6	2.93	0.28	2.93	0.28
Latent Mean	0.00	NA	-0.35	0.09
Latent Variance	1.00	NA	0.77	0.13

Figure 3.38 Differential item and test functioning by specialty group (General Internal Medicine and Physical Medicine and Rehabilitation/Preventive Medicine/Occupational Medicine) – DP subscale

a) Expected total score functions for reference (General Internal Medicine) and focal (Physical Medicine and Rehabilitation/Preventive Medicine/Occupational Medicine) groups

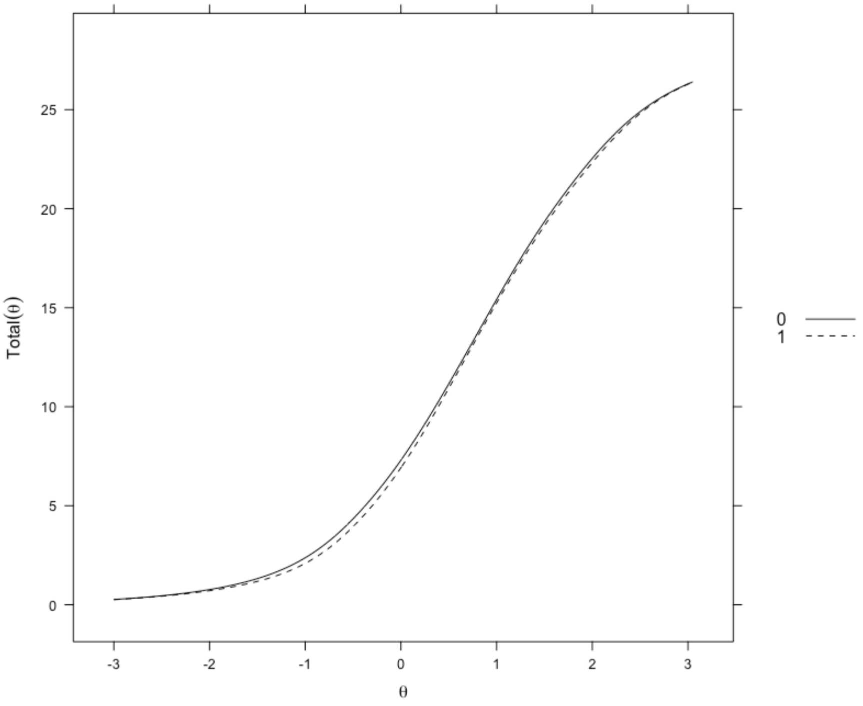

b) Expected item score functions for reference (General Internal Medicine) and focal (Physical Medicine and Rehabilitation/Preventive Medicine/Occupational Medicine) groups

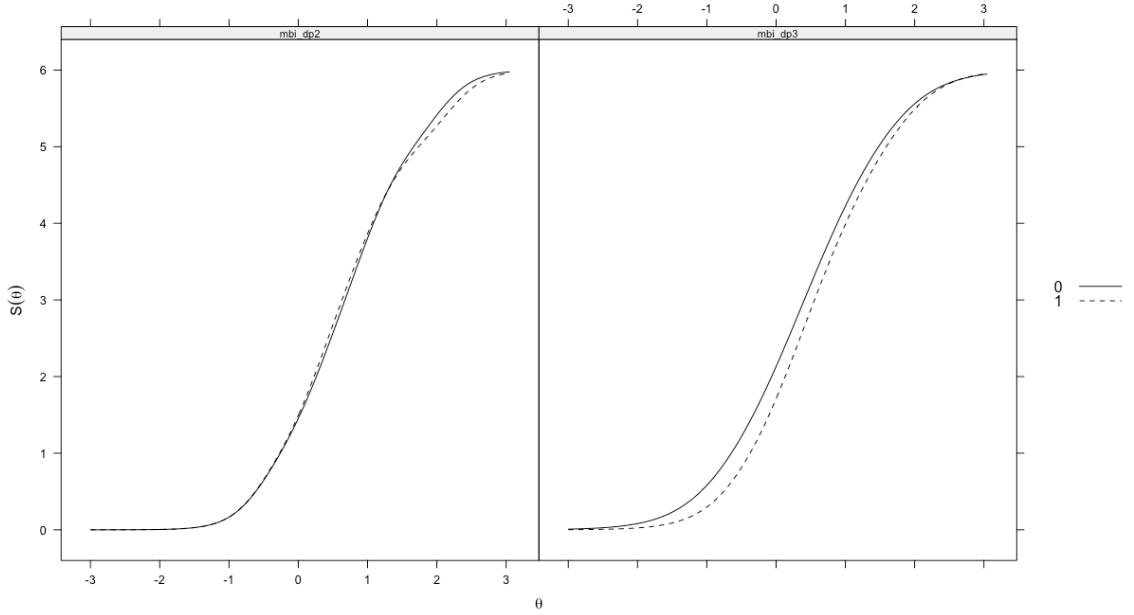

Table 3.38 Multi-group IRT item parameter estimates and standard errors (SE) by specialty group (reference: General Internal Medicine; focal: Physical Medicine and Rehabilitation/Preventive Medicine/Occupational Medicine) – DP subscale

	**Reference group item parameter estimates**	**Reference group SE**	**Focal group item parameter estimates**	**Focal group SE**
mbi_dp1.a	1.78	0.16	1.78	0.16
mbi_dp1.b1	0.05	0.07	0.05	0.07
mbi_dp1.b2	0.73	0.09	0.73	0.09
mbi_dp1.b3	1.16	0.10	1.16	0.10
mbi_dp1.b4	1.61	0.13	1.61	0.13
mbi_dp1.b5	2.19	0.17	2.19	0.17
mbi_dp2.a	3.73	0.49	3.77	0.68
mbi_dp2.b1	-0.53	0.07	-0.53	0.12
mbi_dp2.b2	0.12	0.07	0.14	0.09
mbi_dp2.b3	0.49	0.07	0.39	0.10
mbi_dp2.b4	0.88	0.08	0.82	0.11
mbi_dp2.b5	1.18	0.09	1.20	0.14
mbi_dp2.b6	2.02	0.14	2.18	0.25
mbi_dp3.a	2.24	0.21	2.63	0.37

mbi_dp3.b1	-0.74	0.09	-0.47	0.12
mbi_dp3.b2	-0.10	0.07	0.11	0.10
mbi_dp3.b3	0.23	0.07	0.35	0.10
mbi_dp3.b4	0.56	0.08	0.63	0.11
mbi_dp3.b5	0.94	0.09	1.14	0.14
mbi_dp3.b6	1.60	0.12	1.80	0.21
mbi_dp4.a	1.67	0.17	1.67	0.17
mbi_dp4.b1	0.36	0.08	0.36	0.08
mbi_dp4.b2	1.11	0.11	1.11	0.11
mbi_dp4.b3	1.52	0.13	1.52	0.13
mbi_dp4.b4	1.85	0.16	1.85	0.16
mbi_dp4.b5	2.36	0.20	2.36	0.20
mbi_dp5.a	1.14	0.11	1.14	0.11
mbi_dp5.b1	-1.61	0.16	-1.61	0.16
mbi_dp5.b2	-0.17	0.09	-0.17	0.09
mbi_dp5.b3	0.44	0.10	0.44	0.10
mbi_dp5.b4	1.00	0.12	1.00	0.12
mbi_dp5.b5	1.60	0.16	1.60	0.16
mbi_dp5.b6	2.75	0.25	2.75	0.25
Latent Mean	0.00	NA	-0.06	0.10
Latent Variance	1.00	NA	1.08	0.21

Figure 3.39 Differential item and test functioning by specialty group (General Internal Medicine and Psychiatry) – DP subscale

a) Expected total score functions for reference (General Internal Medicine) and focal (Psychiatry) groups

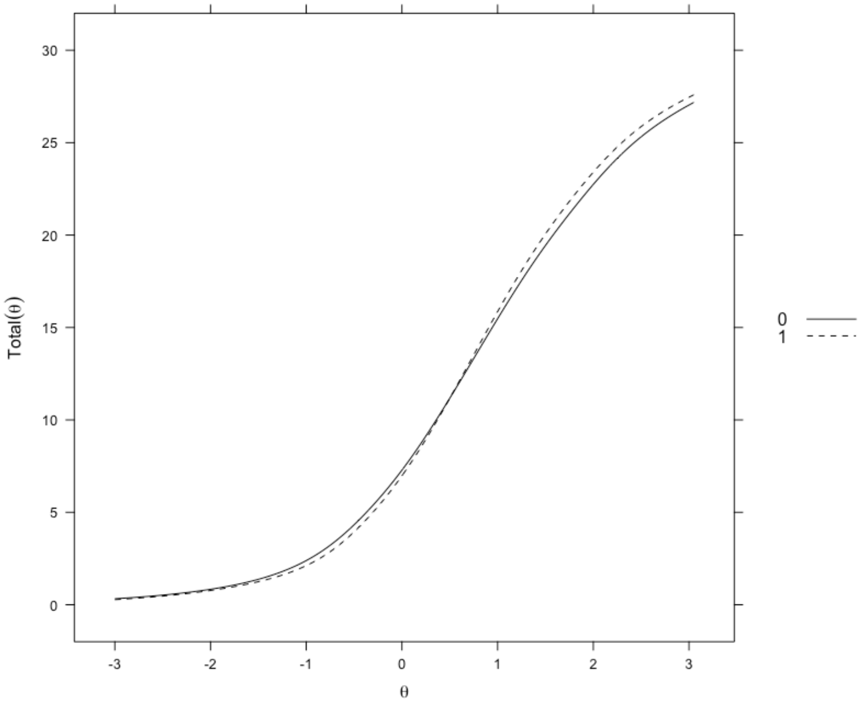

b) Expected item score functions for reference (General Internal Medicine) and focal (Psychiatry) groups

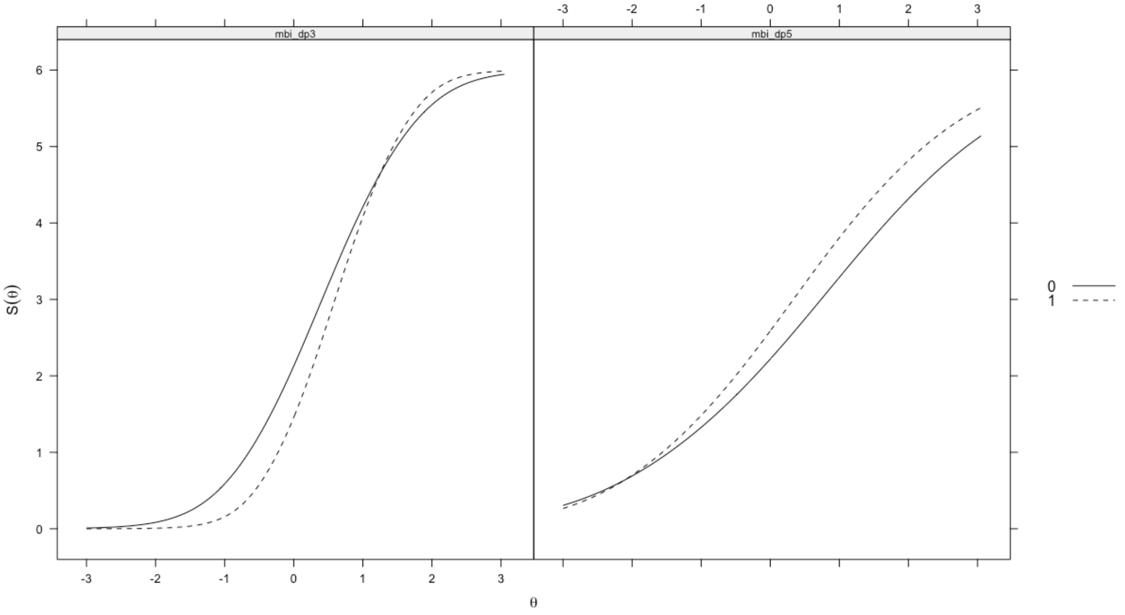

Brady et al. (2021)

Table 3.39 Multi-group IRT item parameter estimates and standard errors (SE) by specialty group (reference: General Internal Medicine; focal: Psychiatry) – DP subscale

	**Reference group item parameter estimates**	**Reference group SE**	**Focal group item parameter estimates**	**Focal group SE**
mbi_dp1.a	1.92	0.15	1.92	0.15
mbi_dp1.b1	-0.03	0.07	-0.03	0.07
mbi_dp1.b2	0.69	0.08	0.69	0.08
mbi_dp1.b3	1.09	0.09	1.09	0.09
mbi_dp1.b4	1.60	0.11	1.60	0.11
mbi_dp1.b5	2.10	0.15	2.10	0.15
mbi_dp1.b6	3.32	0.28	3.32	0.28
mbi_dp2.a	4.07	0.39	4.07	0.39
mbi_dp2.b1	-0.50	0.07	-0.50	0.07
mbi_dp2.b2	0.15	0.06	0.15	0.06
mbi_dp2.b3	0.48	0.06	0.48	0.06
mbi_dp2.b4	0.85	0.07	0.85	0.07
mbi_dp2.b5	1.21	0.08	1.21	0.08
mbi_dp2.b6	2.03	0.12	2.03	0.12
mbi_dp3.a	2.22	0.20	3.17	0.32
mbi_dp3.b1	-0.74	0.09	-0.37	0.08
mbi_dp3.b2	-0.10	0.07	0.18	0.07
mbi_dp3.b3	0.23	0.07	0.43	0.07
mbi_dp3.b4	0.56	0.08	0.77	0.08
mbi_dp3.b5	0.94	0.09	1.01	0.09
mbi_dp3.b6	1.61	0.12	1.60	0.13
mbi_dp4.a	1.72	0.14	1.72	0.14
mbi_dp4.b1	0.33	0.07	0.33	0.07
mbi_dp4.b2	1.05	0.09	1.05	0.09
mbi_dp4.b3	1.51	0.12	1.51	0.12
mbi_dp4.b4	1.92	0.14	1.92	0.14
mbi_dp4.b5	2.30	0.17	2.30	0.17
mbi_dp4.b6	3.24	0.28	3.24	0.28
mbi_dp5.a	1.05	0.12	1.24	0.14
mbi_dp5.b1	-1.73	0.20	-1.78	0.20
mbi_dp5.b2	-0.15	0.11	-0.47	0.11
mbi_dp5.b3	0.49	0.12	0.03	0.10
mbi_dp5.b4	1.05	0.15	0.62	0.11

mbi_dp5.b5	1.65	0.20	1.16	0.15
mbi_dp5.b6	2.90	0.32	2.44	0.27
Latent Mean	0.00	NA	-0.14	0.07
Latent Variance	1.00	NA	0.83	0.11

Figure 3.40 Differential item and test functioning by specialty group (General Internal Medicine and Radiology) – DP subscale

a) Expected total score functions for reference (General Internal Medicine) and focal (Radiology) groups

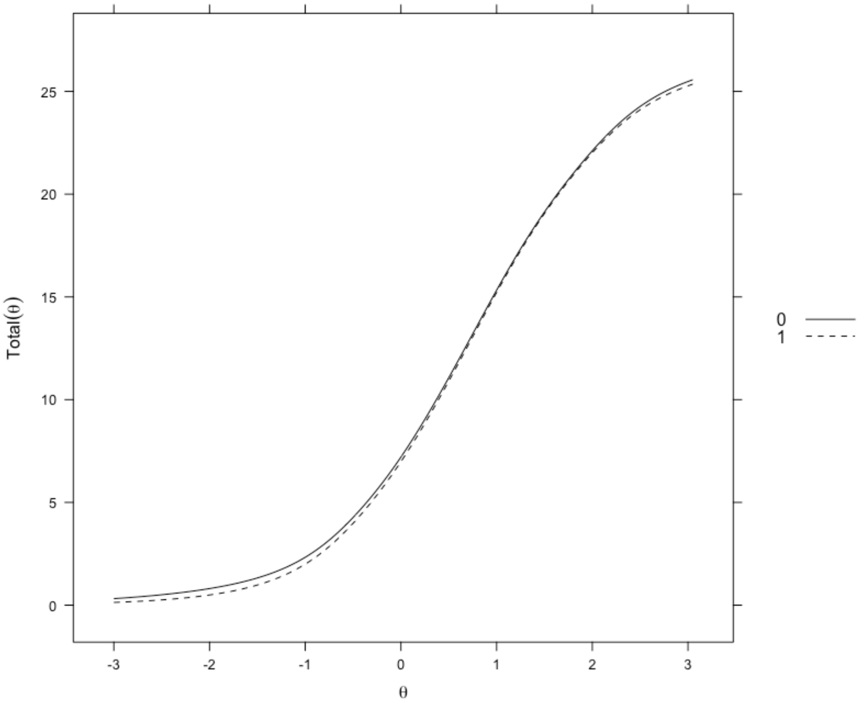

b) Expected item score functions for reference (General Internal Medicine) and focal (Radiology) groups

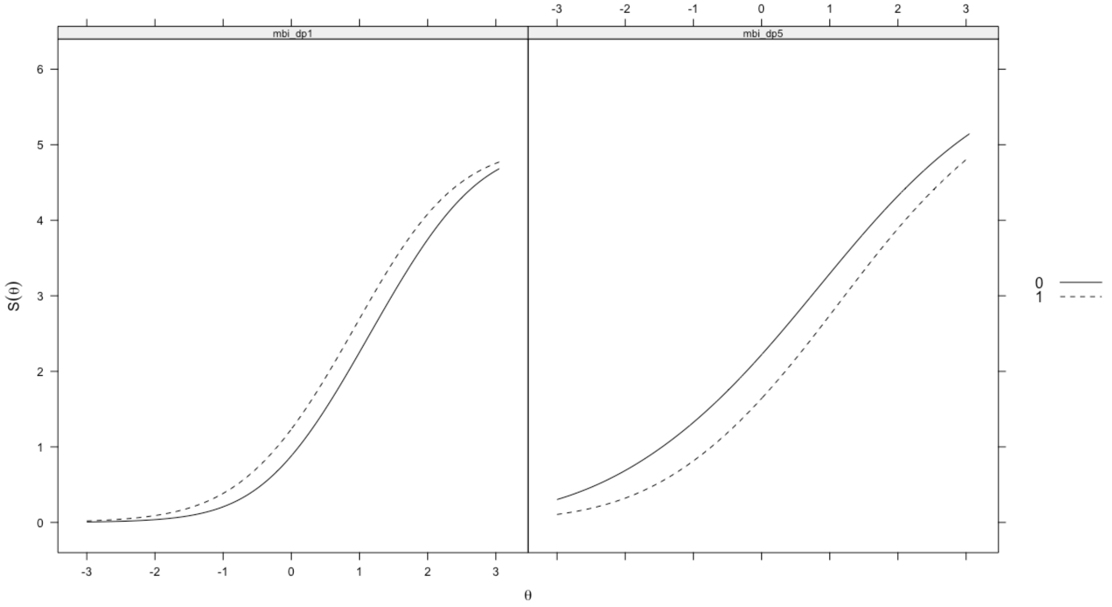

Table 3.40 Multi-group IRT item parameter estimates and standard errors (SE) by specialty group (reference: General Internal Medicine; focal: Radiology) – DP subscale

	**Reference group item parameter estimates**	**Reference group SE**	**Focal group item parameter estimates**	**Focal group SE**
mbi_dp1.a	1.82	0.19	1.57	0.23
mbi_dp1.b1	0.03	0.08	-0.20	0.12
mbi_dp1.b2	0.71	0.09	0.58	0.13
mbi_dp1.b3	1.11	0.11	0.91	0.15
mbi_dp1.b4	1.66	0.15	1.27	0.19
mbi_dp1.b5	2.28	0.20	1.73	0.25
mbi_dp2.a	3.83	0.43	3.83	0.43
mbi_dp2.b1	-0.55	0.07	-0.55	0.07
mbi_dp2.b2	0.10	0.06	0.10	0.06
mbi_dp2.b3	0.47	0.06	0.47	0.06
mbi_dp2.b4	0.84	0.07	0.84	0.07
mbi_dp2.b5	1.16	0.08	1.16	0.08
mbi_dp2.b6	2.07	0.13	2.07	0.13
mbi_dp3.a	2.41	0.19	2.41	0.19
mbi_dp3.b1	-0.72	0.08	-0.72	0.08
mbi_dp3.b2	-0.11	0.07	-0.11	0.07

mbi_dp3.b3	0.24	0.07	0.24	0.07
mbi_dp3.b4	0.57	0.07	0.57	0.07
mbi_dp3.b5	0.95	0.08	0.95	0.08
mbi_dp3.b6	1.55	0.11	1.55	0.11
mbi_dp4.a	1.83	0.18	1.83	0.18
mbi_dp4.b1	0.44	0.08	0.44	0.08
mbi_dp4.b2	1.05	0.10	1.05	0.10
mbi_dp4.b3	1.53	0.13	1.53	0.13
mbi_dp4.b4	1.90	0.15	1.90	0.15
mbi_dp5.a	1.05	0.12	1.24	0.19
mbi_dp5.b1	-1.72	0.20	-0.97	0.18
mbi_dp5.b2	-0.16	0.11	0.27	0.14
mbi_dp5.b3	0.49	0.12	0.96	0.18
mbi_dp5.b4	1.05	0.15	1.46	0.24
mbi_dp5.b5	1.64	0.19	2.11	0.32
mbi_dp5.b6	2.89	0.32	3.70	0.62
Latent Mean	0.00	NA	-0.18	0.09
Latent Variance	1.00	NA	1.02	0.17

Figure 3.41 Differential item and test functioning by gender (Male and Female) – PA subscale

a) Expected total score functions for reference (Male) and focal (Female) groups

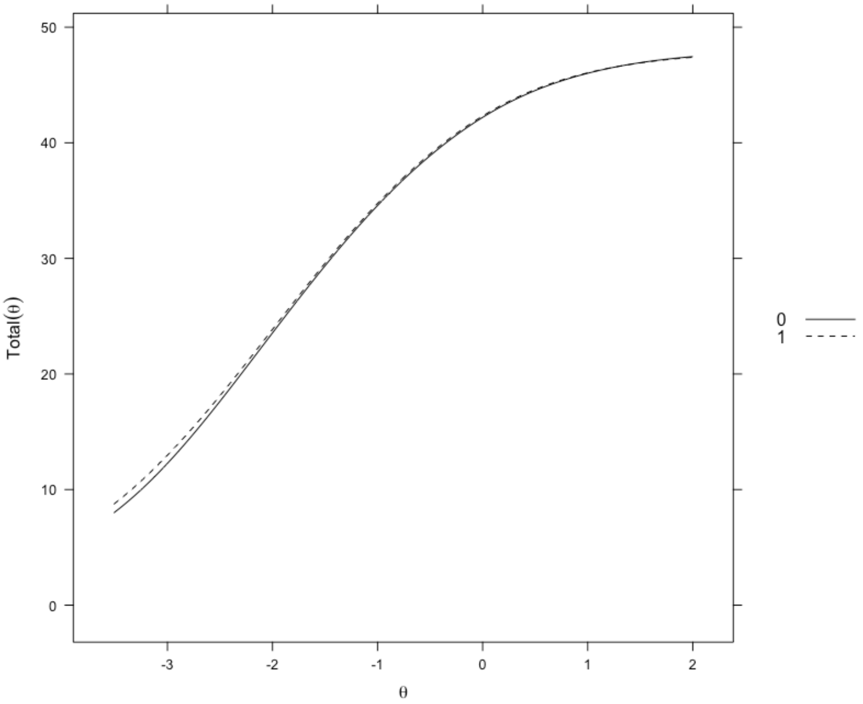

b) Expected item score functions for reference (Male) and focal (Female) groups

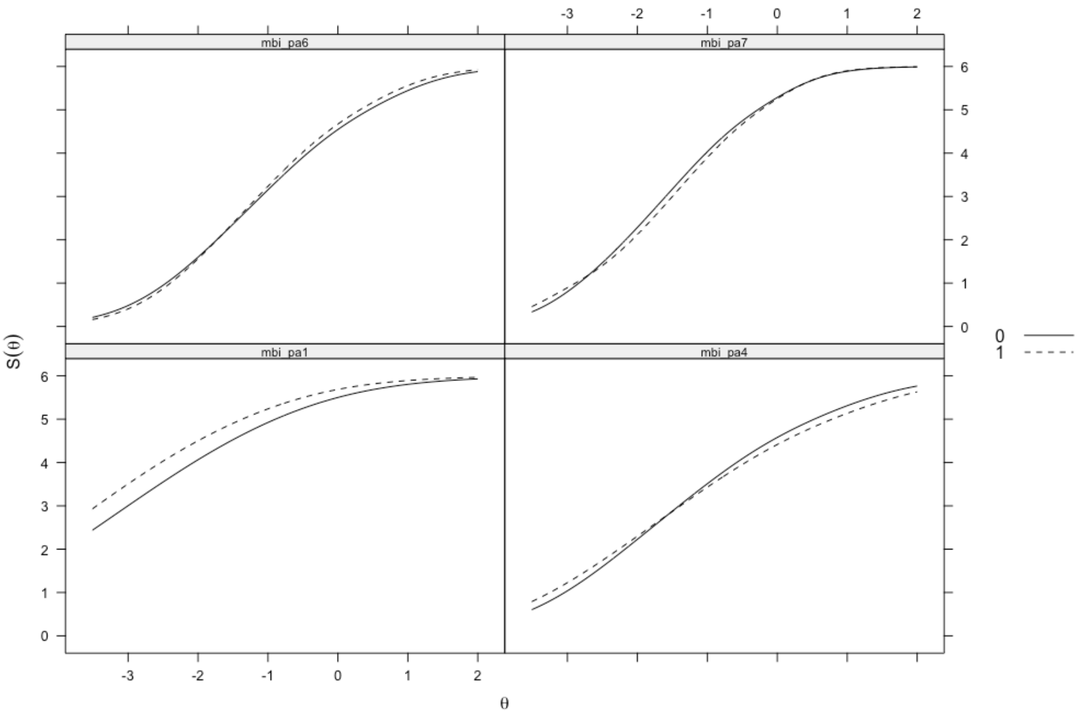

Table 3.41 Multi-group IRT item parameter estimates and standard errors (SE) by gender (reference: Male; focal: Female) – PA subscale

	**Reference group item parameter estimates**	**Reference group SE**	**Focal group item parameter estimates**	**Focal group SE**
mbi_pa1.a	1.10	0.05	1.19	0.08
mbi_pa1.b1	-4.85	0.23	-5.20	0.41
mbi_pa1.b2	-4.10	0.18	-4.52	0.32
mbi_pa1.b3	-3.50	0.14	-3.98	0.26
mbi_pa1.b4	-2.58	0.10	-3.07	0.18
mbi_pa1.b5	-2.02	0.08	-2.44	0.14
mbi_pa1.b6	-0.73	0.04	-1.06	0.06
mbi_pa2.a	1.65	0.06	1.65	0.06
mbi_pa2.b1	-3.50	0.11	-3.50	0.11
mbi_pa2.b2	-3.22	0.10	-3.22	0.10
mbi_pa2.b3	-2.96	0.09	-2.96	0.09
mbi_pa2.b4	-2.49	0.07	-2.49	0.07
mbi_pa2.b5	-2.03	0.06	-2.03	0.06
mbi_pa2.b6	-0.85	0.03	-0.85	0.03
mbi_pa3.a	2.56	0.08	2.56	0.08
mbi_pa3.b1	-3.04	0.09	-3.04	0.09
mbi_pa3.b2	-2.50	0.06	-2.50	0.06
mbi_pa3.b3	-2.13	0.05	-2.13	0.05
mbi_pa3.b4	-1.59	0.04	-1.59	0.04
mbi_pa3.b5	-1.20	0.03	-1.20	0.03
mbi_pa3.b6	-0.31	0.02	-0.31	0.02
mbi_pa4.a	1.52	0.05	1.36	0.08
mbi_pa4.b1	-3.03	0.10	-3.26	0.17
mbi_pa4.b2	-2.47	0.08	-2.64	0.13
mbi_pa4.b3	-1.85	0.06	-1.84	0.08
mbi_pa4.b4	-1.01	0.04	-0.97	0.05
mbi_pa4.b5	-0.50	0.03	-0.33	0.04
mbi_pa4.b6	1.08	0.04	1.39	0.09
mbi_pa5.a	1.90	0.06	1.90	0.06
mbi_pa5.b1	-2.94	0.08	-2.94	0.08
mbi_pa5.b2	-2.59	0.07	-2.59	0.07
mbi_pa5.b3	-2.31	0.06	-2.31	0.06
mbi_pa5.b4	-1.84	0.05	-1.84	0.05

mbi_pa5.b5	-1.48	0.04	-1.48	0.04
mbi_pa5.b6	-0.47	0.02	-0.47	0.02
mbi_pa6.a	1.95	0.06	2.11	0.11
mbi_pa6.b1	-2.58	0.07	-2.51	0.10
mbi_pa6.b2	-1.93	0.05	-1.91	0.07
mbi_pa6.b3	-1.43	0.04	-1.46	0.05
mbi_pa6.b4	-0.79	0.03	-0.88	0.04
mbi_pa6.b5	-0.33	0.03	-0.40	0.04
mbi_pa6.b6	0.89	0.03	0.74	0.06
mbi_pa7.a	2.49	0.08	2.69	0.14
mbi_pa7.b1	-3.08	0.10	-3.38	0.21
mbi_pa7.b2	-2.38	0.06	-2.24	0.08
mbi_pa7.b3	-1.86	0.05	-1.75	0.06
mbi_pa7.b4	-1.31	0.03	-1.23	0.04
mbi_pa7.b5	-0.89	0.03	-0.78	0.04
mbi_pa7.b6	0.11	0.02	0.13	0.04
mbi_pa8.a	1.23	0.04	1.23	0.04
mbi_pa8.b1	-4.89	0.20	-4.89	0.20
mbi_pa8.b2	-3.85	0.13	-3.85	0.13
mbi_pa8.b3	-3.08	0.10	-3.08	0.10
mbi_pa8.b4	-2.24	0.07	-2.24	0.07
mbi_pa8.b5	-1.59	0.05	-1.59	0.05
mbi_pa8.b6	-0.17	0.03	-0.17	0.03
Latent Mean	0.00	NA	-0.21	0.03
Latent Variance	1.00	NA	0.86	0.06

Figure 3.42 Differential item and test functioning by gender (≥65 and <35 years) – PA subscale

a) Expected total score functions for reference (≥65 years) and focal (<35 years) groups

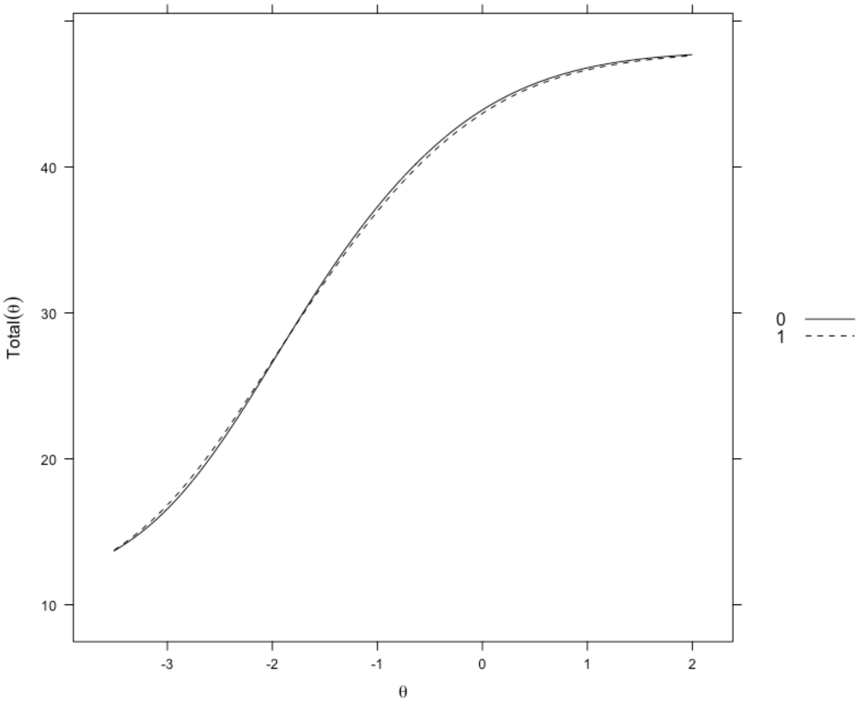

b) Expected item score functions for reference (≥65 years) and focal (<35 years) groups

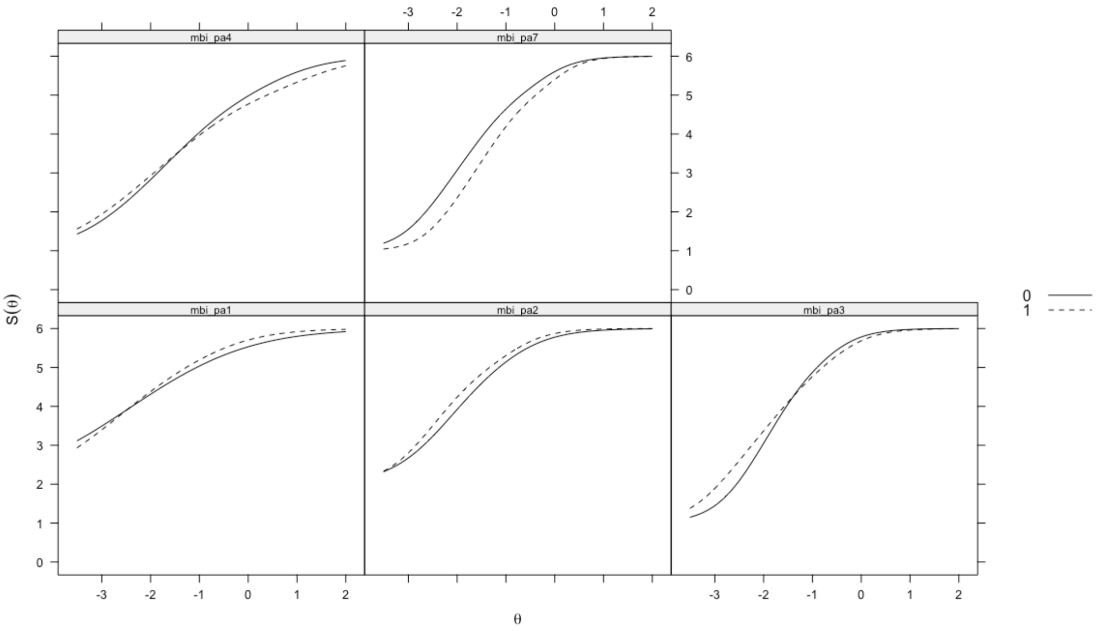

Table 3.42 Multi-group IRT item parameter estimates and standard errors (SE) by gender (reference: ≥65 years; focal: <35 years) – PA subscale

	**Reference group item parameter estimates**	**Reference group SE**	**Focal group item parameter estimates**	**Focal group SE**
mbi_pa1.a	0.98	0.09	1.43	0.23
mbi_pa1.b1	-3.50	0.29	-3.55	0.46
mbi_pa1.b2	-2.66	0.21	-2.83	0.33
mbi_pa1.b3	-2.27	0.18	-2.15	0.23
mbi_pa1.b4	-1.03	0.10	-0.86	0.11
mbi_pa2.a	1.77	0.13	2.31	0.33
mbi_pa2.b1	-2.58	0.15	-2.93	0.30
mbi_pa2.b2	-2.23	0.13	-2.51	0.23
mbi_pa2.b3	-1.88	0.10	-2.01	0.16
mbi_pa2.b4	-0.94	0.06	-0.89	0.08
mbi_pa3.a	2.39	0.16	2.32	0.31
mbi_pa3.b1	-2.48	0.13	-3.01	0.31
mbi_pa3.b2	-2.24	0.11	-2.62	0.24
mbi_pa3.b3	-1.78	0.08	-1.91	0.15
mbi_pa3.b4	-1.52	0.07	-1.33	0.10
mbi_pa3.b5	-0.66	0.05	-0.46	0.08
mbi_pa4.a	1.56	0.10	1.54	0.21
mbi_pa4.b1	-2.84	0.17	-3.11	0.34
mbi_pa4.b2	-2.18	0.12	-2.38	0.23
mbi_pa4.b3	-1.38	0.08	-1.48	0.13
mbi_pa4.b4	-1.02	0.07	-0.74	0.10
mbi_pa4.b5	0.56	0.06	1.20	0.24
mbi_pa5.a	2.03	0.13	2.03	0.13
mbi_pa5.b1	-2.42	0.12	-2.42	0.12
mbi_pa5.b2	-2.02	0.10	-2.02	0.10
mbi_pa5.b3	-1.68	0.08	-1.68	0.08
mbi_pa5.b4	-0.70	0.05	-0.70	0.05
mbi_pa6.a	2.04	0.11	2.04	0.11
mbi_pa6.b1	-2.64	0.13	-2.64	0.13
mbi_pa6.b2	-2.19	0.10	-2.19	0.10
mbi_pa6.b3	-1.82	0.08	-1.82	0.08
mbi_pa6.b4	-1.21	0.06	-1.21	0.06
mbi_pa6.b5	-0.76	0.05	-0.76	0.05

Brady et al. (2021)

mbi_pa6.b6	0.41	0.05	0.41	0.05
mbi_pa7.a	2.46	0.16	2.95	0.37
mbi_pa7.b1	-2.71	0.14	-2.37	0.19
mbi_pa7.b2	-2.33	0.11	-1.88	0.13
mbi_pa7.b3	-1.75	0.08	-1.41	0.10
mbi_pa7.b4	-1.32	0.06	-0.94	0.08
mbi_pa7.b5	-0.27	0.04	0.03	0.09
mbi_pa8.a	1.35	0.09	1.35	0.09
mbi_pa8.b1	-3.45	0.21	-3.45	0.21
mbi_pa8.b2	-2.94	0.17	-2.94	0.17
mbi_pa8.b3	-2.28	0.13	-2.28	0.13
mbi_pa8.b4	-1.73	0.10	-1.73	0.10
mbi_pa8.b5	-0.41	0.05	-0.41	0.05
Latent Mean	0.00	NA	-0.61	0.07
Latent Variance	1.00	NA	0.67	0.10

Figure 3.43 Differential item and test functioning by gender (≥65 and 35-44 years) – PA subscale

a) Expected total score functions for reference (≥65 years) and focal (35-44 years) groups

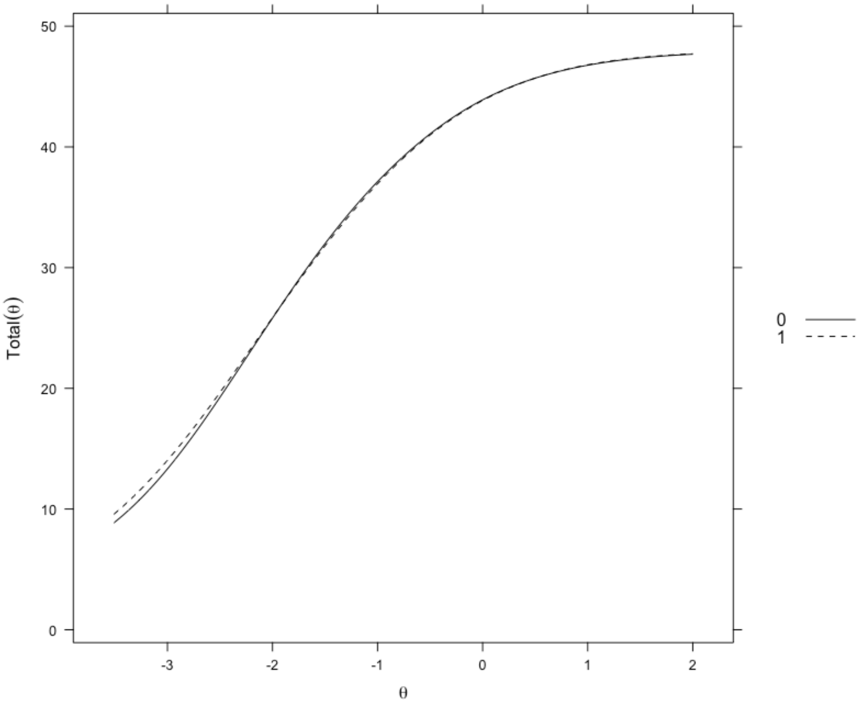

b) Expected item score functions for reference (≥65 years) and focal (35-44 years) groups

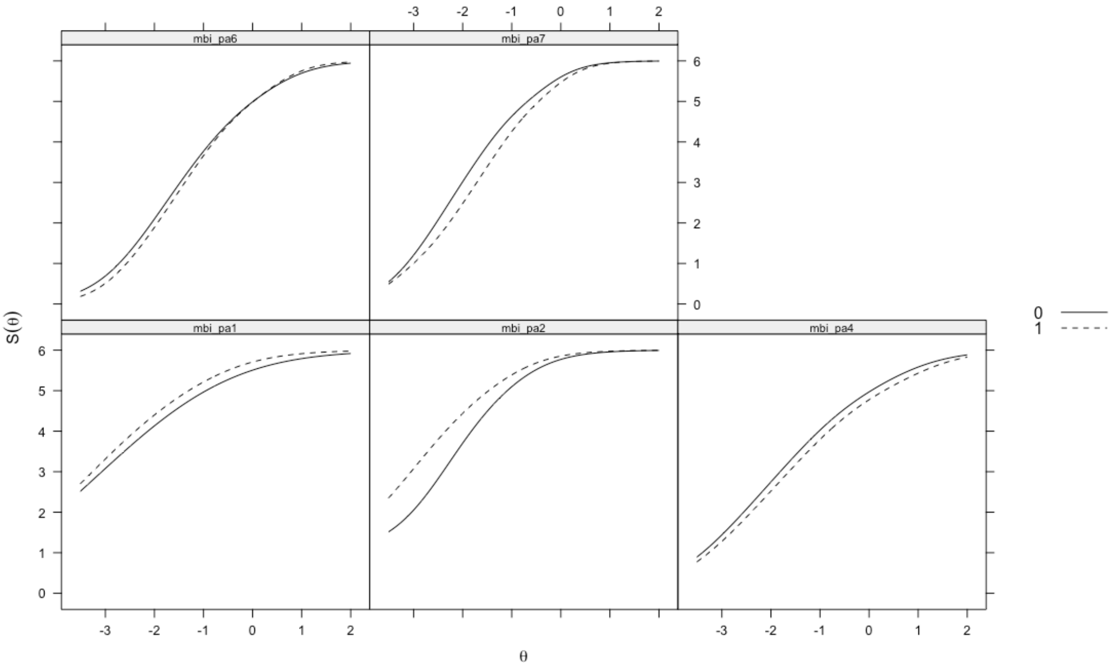

Table 3.43 Multi-group IRT item parameter estimates and standard errors (SE) by gender (reference: ≥65 years; focal: 35-44 years) – PA subscale

	**Reference group item parameter estimates**	**Reference group SE**	**Focal group item parameter estimates**	**Focal group SE**
mbi_pa1.a	0.98	0.09	1.35	0.12
mbi_pa1.b1	-4.78	0.43	-5.07	0.49
mbi_pa1.b2	-4.07	0.34	-4.25	0.35
mbi_pa1.b3	-3.50	0.28	-3.80	0.29
mbi_pa1.b4	-2.65	0.21	-2.88	0.19
mbi_pa1.b5	-2.26	0.18	-2.24	0.14
mbi_pa1.b6	-1.02	0.10	-0.95	0.07
mbi_pa2.a	1.78	0.13	1.80	0.15
mbi_pa2.b1	-2.74	0.16	-3.76	0.28
mbi_pa2.b2	-2.58	0.15	-3.35	0.23
mbi_pa2.b3	-2.23	0.12	-2.77	0.17
mbi_pa2.b4	-1.88	0.10	-2.21	0.12
mbi_pa2.b5	-0.93	0.06	-1.12	0.06
mbi_pa3.a	2.71	0.15	2.71	0.15
mbi_pa3.b1	-3.05	0.14	-3.05	0.14

mbi_pa3.b2	-2.54	0.10	-2.54	0.10
mbi_pa3.b3	-2.24	0.09	-2.24	0.09
mbi_pa3.b4	-1.75	0.07	-1.75	0.07
mbi_pa3.b5	-1.39	0.06	-1.39	0.06
mbi_pa3.b6	-0.57	0.04	-0.57	0.04
mbi_pa4.a	1.53	0.09	1.61	0.12
mbi_pa4.b1	-3.34	0.21	-3.35	0.22
mbi_pa4.b2	-2.88	0.17	-2.73	0.16
mbi_pa4.b3	-2.21	0.12	-2.00	0.11
mbi_pa4.b4	-1.40	0.08	-1.28	0.07
mbi_pa4.b5	-1.03	0.07	-0.72	0.06
mbi_pa4.b6	0.56	0.06	0.93	0.12
mbi_pa5.a	2.12	0.12	2.12	0.12
mbi_pa5.b1	-2.93	0.13	-2.93	0.13
mbi_pa5.b2	-2.65	0.11	-2.65	0.11
mbi_pa5.b3	-2.44	0.10	-2.44	0.10
mbi_pa5.b4	-2.03	0.08	-2.03	0.08
mbi_pa5.b5	-1.71	0.07	-1.71	0.07
mbi_pa5.b6	-0.73	0.04	-0.73	0.04
mbi_pa6.a	1.89	0.11	2.39	0.17
mbi_pa6.b1	-2.69	0.14	-2.73	0.15
mbi_pa6.b2	-2.19	0.11	-2.13	0.10
mbi_pa6.b3	-1.85	0.09	-1.66	0.08
mbi_pa6.b4	-1.22	0.07	-1.13	0.06
mbi_pa6.b5	-0.79	0.05	-0.66	0.05
mbi_pa6.b6	0.41	0.05	0.47	0.08
mbi_pa7.a	2.47	0.16	2.71	0.20
mbi_pa7.b1	-3.28	0.21	-3.39	0.24
mbi_pa7.b2	-2.70	0.14	-2.46	0.12
mbi_pa7.b3	-2.33	0.11	-1.98	0.09
mbi_pa7.b4	-1.75	0.08	-1.43	0.07
mbi_pa7.b5	-1.31	0.06	-1.01	0.05
mbi_pa7.b6	-0.26	0.04	-0.08	0.06
mbi_pa8.a	1.35	0.08	1.35	0.08
mbi_pa8.b1	-3.77	0.20	-3.77	0.20
mbi_pa8.b2	-3.07	0.15	-3.07	0.15
mbi_pa8.b3	-2.34	0.11	-2.34	0.11

Brady et al. (2021)

mbi_pa8.b4	-1.75	0.08	-1.75	0.08
mbi_pa8.b5	-0.44	0.05	-0.44	0.05
Latent Mean	0.00	NA	-0.65	0.05
Latent Variance	1.00	NA	0.67	0.07

Figure 3.44 Differential item and test functioning by gender (≥65 and 45-54 years) – PA subscale

a) Expected total score functions for reference (≥65 years) and focal (45-54 years) groups

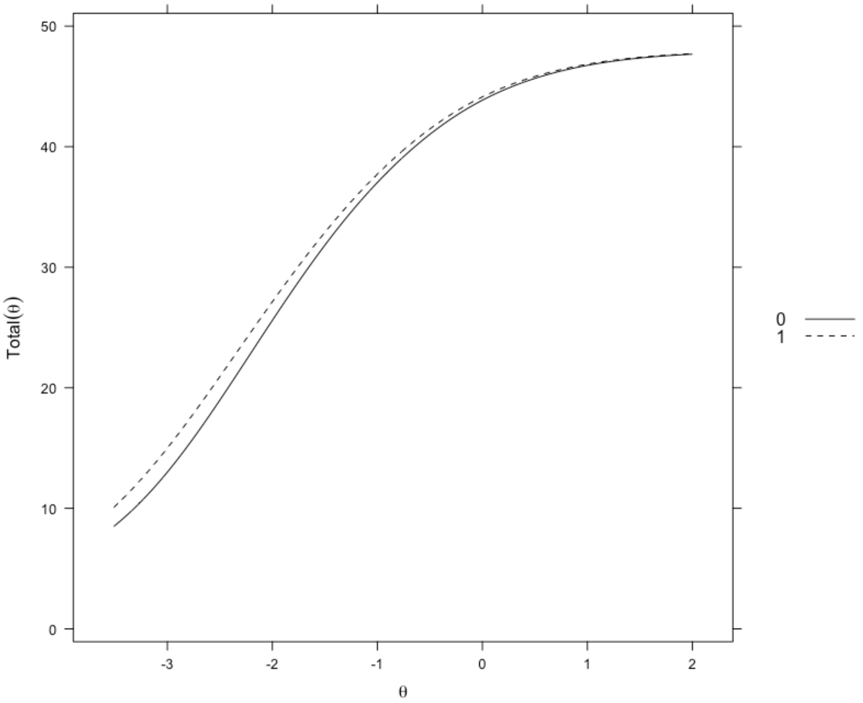

b) Expected item score functions for reference (≥65 years) and focal (45-54 years) groups

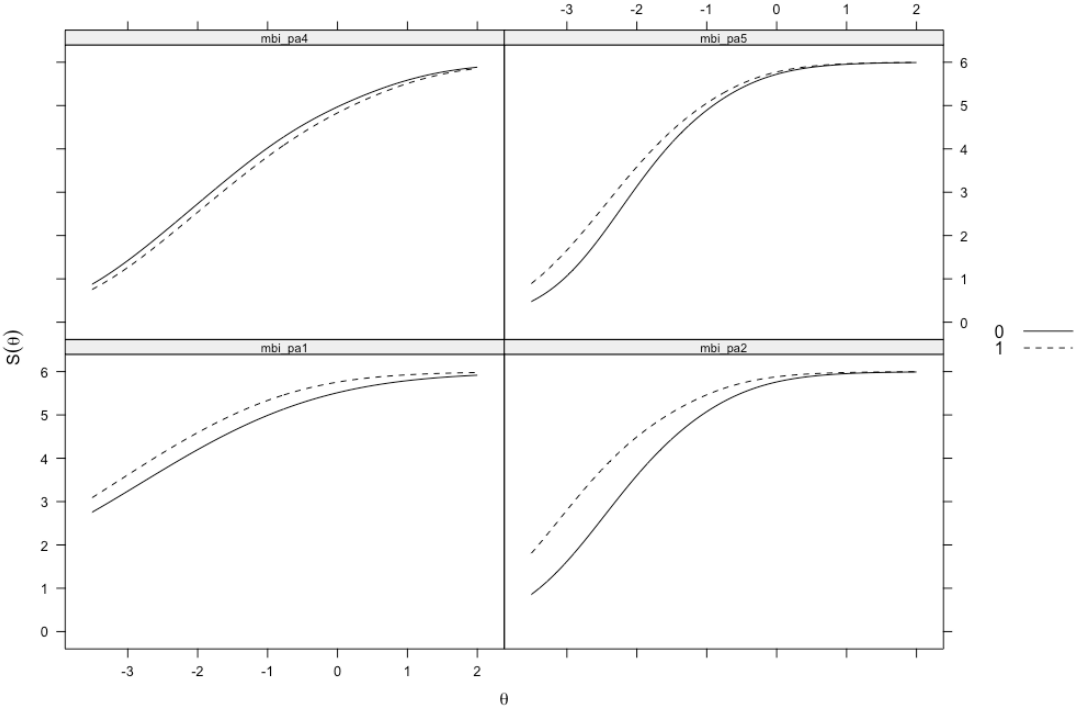

Table 3.44 Multi-group IRT item parameter estimates and standard errors (SE) by gender (reference: ≥65 years; focal: 45-54 years) – PA subscale

	**Reference group item parameter estimates**	**Reference group SE**	**Focal group item parameter estimates**	**Focal group SE**
mbi_pa1.a	0.97	0.09	1.29	0.11
mbi_pa1.b1	-4.09	0.35	-4.73	0.39
mbi_pa1.b2	-3.51	0.29	-4.07	0.30
mbi_pa1.b3	-2.66	0.21	-3.00	0.20
mbi_pa1.b4	-2.27	0.18	-2.43	0.15
mbi_pa1.b5	-1.03	0.10	-1.21	0.08
mbi_pa2.a	1.75	0.13	1.82	0.14
mbi_pa2.b1	-3.01	0.19	-3.71	0.24
mbi_pa2.b2	-2.78	0.17	-3.48	0.21
mbi_pa2.b3	-2.62	0.15	-3.22	0.19
mbi_pa2.b4	-2.26	0.13	-2.81	0.15
mbi_pa2.b5	-1.90	0.10	-2.43	0.13
mbi_pa2.b6	-0.94	0.06	-1.24	0.07
mbi_pa3.a	2.67	0.14	2.67	0.14

mbi_pa3.b1	-3.05	0.13	-3.05	0.13
mbi_pa3.b2	-2.65	0.11	-2.65	0.11
mbi_pa3.b3	-2.33	0.09	-2.33	0.09
mbi_pa3.b4	-1.83	0.07	-1.83	0.07
mbi_pa3.b5	-1.50	0.06	-1.50	0.06
mbi_pa3.b6	-0.66	0.04	-0.66	0.04
mbi_pa4.a	1.54	0.09	1.54	0.10
mbi_pa4.b1	-3.32	0.21	-3.20	0.18
mbi_pa4.b2	-2.87	0.17	-2.70	0.14
mbi_pa4.b3	-2.20	0.12	-2.11	0.11
mbi_pa4.b4	-1.39	0.08	-1.27	0.07
mbi_pa4.b5	-1.02	0.07	-0.74	0.06
mbi_pa4.b6	0.56	0.06	0.69	0.09
mbi_pa5.a	1.92	0.13	1.97	0.14
mbi_pa5.b1	-2.67	0.15	-3.39	0.20
mbi_pa5.b2	-2.46	0.13	-2.87	0.15
mbi_pa5.b3	-2.38	0.13	-2.54	0.13
mbi_pa5.b4	-2.02	0.10	-2.16	0.10
mbi_pa5.b5	-1.74	0.09	-1.78	0.08
mbi_pa5.b6	-0.73	0.05	-0.80	0.05
mbi_pa6.a	1.99	0.09	1.99	0.09
mbi_pa6.b1	-2.79	0.11	-2.79	0.11
mbi_pa6.b2	-2.18	0.08	-2.18	0.08
mbi_pa6.b3	-1.77	0.07	-1.77	0.07
mbi_pa6.b4	-1.15	0.05	-1.15	0.05
mbi_pa6.b5	-0.72	0.04	-0.72	0.04
mbi_pa6.b6	0.43	0.05	0.43	0.05
mbi_pa7.a	2.59	0.13	2.59	0.13
mbi_pa7.b1	-3.23	0.15	-3.23	0.15
mbi_pa7.b2	-2.54	0.10	-2.54	0.10
mbi_pa7.b3	-2.14	0.08	-2.14	0.08
mbi_pa7.b4	-1.64	0.06	-1.64	0.06
mbi_pa7.b5	-1.21	0.05	-1.21	0.05
mbi_pa7.b6	-0.27	0.04	-0.27	0.04
mbi_pa8.a	1.22	0.07	1.22	0.07
mbi_pa8.b1	-3.89	0.21	-3.89	0.21
mbi_pa8.b2	-3.27	0.16	-3.27	0.16

Brady et al. (2021)

mbi_pa8.b3	-2.51	0.12	-2.51	0.12
mbi_pa8.b4	-1.90	0.09	-1.90	0.09
mbi_pa8.b5	-0.50	0.05	-0.50	0.05
Latent Mean	0.00	NA	-0.55	0.04
Latent Variance	1.00	NA	0.76	0.07

Figure 3.45 Differential item and test functioning by gender (≥65 and 55-64 years) – PA subscale

a) Expected total score functions for reference (≥65 years) and focal (55-64 years) groups

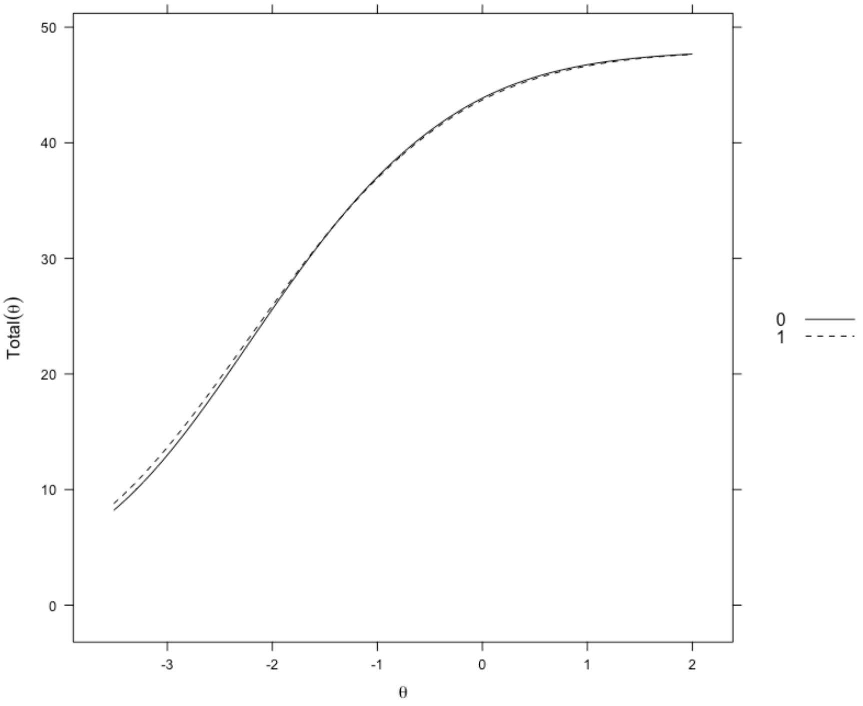

b) Expected item score functions for reference (≥65 years) and focal (55-64 years) groups

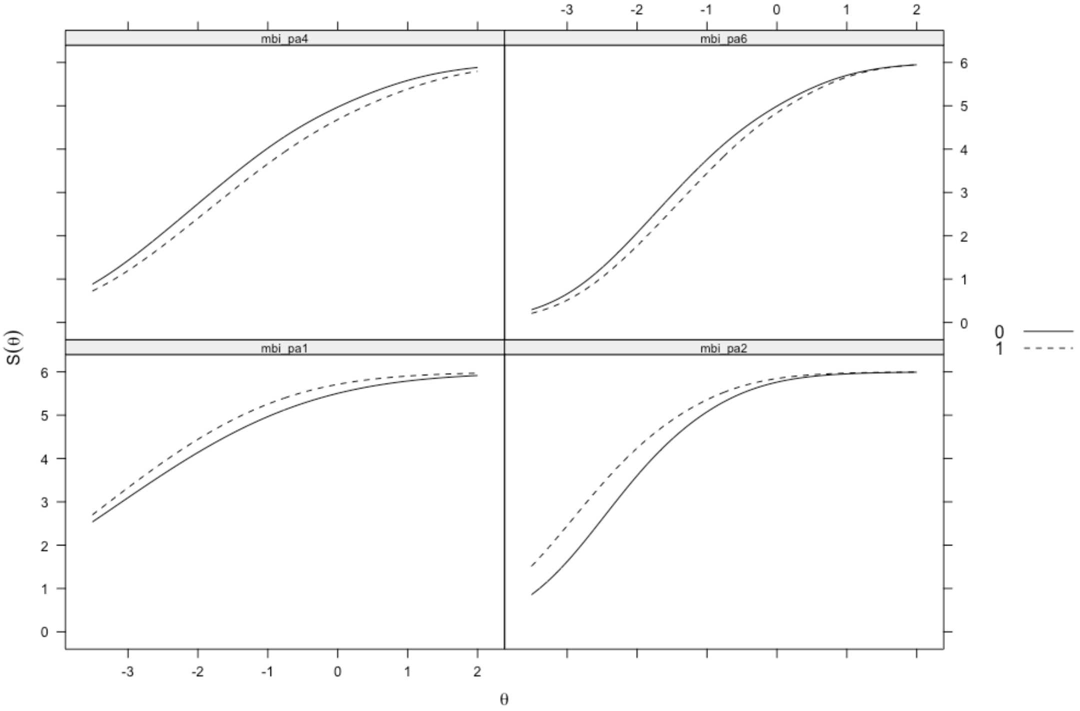

Table 3.45 Multi-group IRT item parameter estimates and standard errors (SE) by gender (reference: ≥65 years; focal: 55-64 years) – PA subscale

	**Reference group item parameter estimates**	**Reference group SE**	**Focal group item parameter estimates**	**Focal group SE**
mbi_pa1.a	0.97	0.08	1.19	0.09
mbi_pa1.b1	-4.80	0.43	-4.89	0.36
mbi_pa1.b2	-4.09	0.35	-4.23	0.29
mbi_pa1.b3	-3.51	0.29	-3.70	0.24
mbi_pa1.b4	-2.66	0.21	-2.90	0.18
mbi_pa1.b5	-2.27	0.18	-2.40	0.14
mbi_pa1.b6	-1.03	0.10	-1.27	0.08
mbi_pa2.a	1.75	0.13	1.76	0.12
mbi_pa2.b1	-3.01	0.19	-3.58	0.21
mbi_pa2.b2	-2.78	0.17	-3.27	0.18
mbi_pa2.b3	-2.62	0.15	-3.04	0.16
mbi_pa2.b4	-2.26	0.13	-2.60	0.13
mbi_pa2.b5	-1.90	0.10	-2.24	0.11
mbi_pa2.b6	-0.94	0.06	-1.16	0.06

Brady et al. (2021)

mbi_pa3.a	2.56	0.13	2.56	0.13
mbi_pa3.b1	-3.17	0.14	-3.17	0.14
mbi_pa3.b2	-2.68	0.11	-2.68	0.11
mbi_pa3.b3	-2.33	0.09	-2.33	0.09
mbi_pa3.b4	-1.85	0.07	-1.85	0.07
mbi_pa3.b5	-1.53	0.06	-1.53	0.06
mbi_pa3.b6	-0.64	0.04	-0.64	0.04
mbi_pa4.a	1.54	0.09	1.48	0.09
mbi_pa4.b1	-3.33	0.21	-3.18	0.17
mbi_pa4.b2	-2.87	0.17	-2.59	0.13
mbi_pa4.b3	-2.20	0.12	-1.99	0.10
mbi_pa4.b4	-1.39	0.08	-1.16	0.06
mbi_pa4.b5	-1.03	0.07	-0.61	0.05
mbi_pa4.b6	0.56	0.06	0.93	0.09
mbi_pa5.a	1.93	0.10	1.93	0.10
mbi_pa5.b1	-2.90	0.12	-2.90	0.12
mbi_pa5.b2	-2.66	0.11	-2.66	0.11
mbi_pa5.b3	-2.45	0.10	-2.45	0.10
mbi_pa5.b4	-2.01	0.08	-2.01	0.08
mbi_pa5.b5	-1.71	0.07	-1.71	0.07
mbi_pa5.b6	-0.77	0.04	-0.77	0.04
mbi_pa6.a	1.92	0.11	2.10	0.12
mbi_pa6.b1	-2.66	0.14	-2.66	0.12
mbi_pa6.b2	-2.18	0.11	-2.05	0.09
mbi_pa6.b3	-1.84	0.09	-1.56	0.07
mbi_pa6.b4	-1.21	0.07	-0.97	0.05
mbi_pa6.b5	-0.78	0.05	-0.57	0.05
mbi_pa6.b6	0.41	0.05	0.55	0.06
mbi_pa7.a	2.66	0.12	2.66	0.12
mbi_pa7.b1	-3.33	0.15	-3.33	0.15
mbi_pa7.b2	-2.60	0.10	-2.60	0.10
mbi_pa7.b3	-2.08	0.08	-2.08	0.08
mbi_pa7.b4	-1.59	0.06	-1.59	0.06
mbi_pa7.b5	-1.20	0.05	-1.20	0.05
mbi_pa7.b6	-0.24	0.04	-0.24	0.04
mbi_pa8.a	1.29	0.07	1.29	0.07
mbi_pa8.b1	-4.52	0.25	-4.52	0.25

Brady et al. (2021)

mbi_pa8.b2	-3.80	0.19	-3.80	0.19
mbi_pa8.b3	-3.15	0.15	-3.15	0.15
mbi_pa8.b4	-2.39	0.11	-2.39	0.11
mbi_pa8.b5	-1.81	0.08	-1.81	0.08
mbi_pa8.b6	-0.47	0.05	-0.47	0.05
Latent Mean	0.00	NA	-0.31	0.04
Latent Variance	1.00	NA	0.87	0.07

Figure 3.46 Differential item and test functioning by specialty (General Internal Medicine and Anesthesiology) – PA subscale

a) Expected total score functions for reference (General Internal Medicine) and focal (Anesthesiology) groups

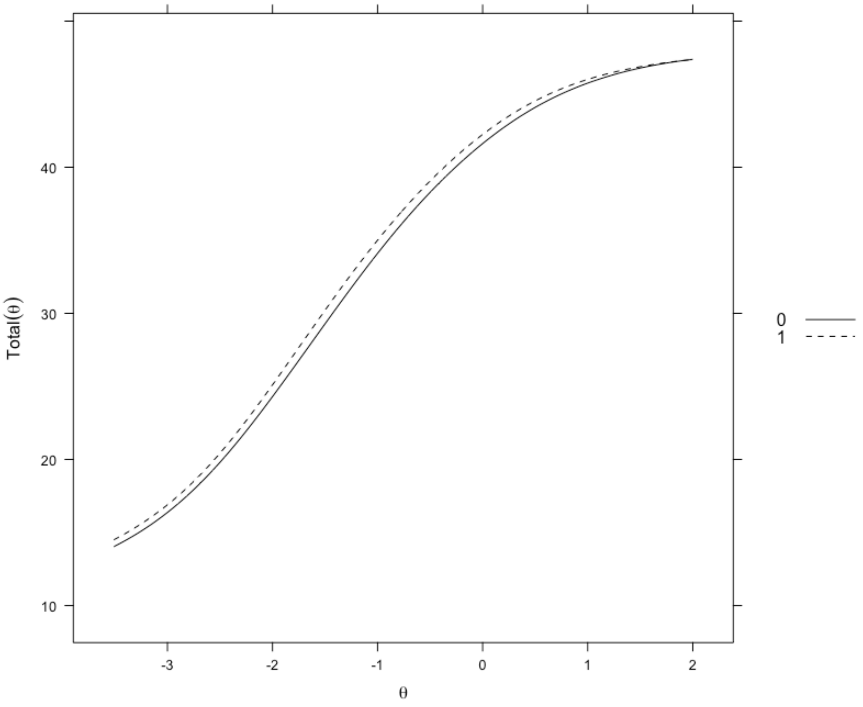

b) Expected item score functions for reference (General Internal Medicine) and focal (Anesthesiology) groups

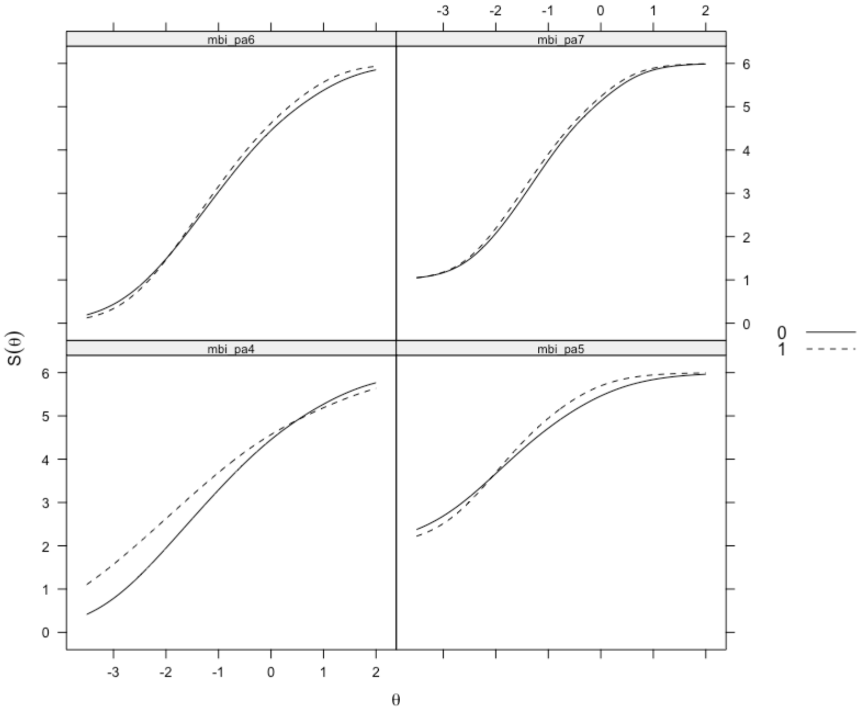

Table 3.46 Multi-group IRT item parameter estimates and standard errors (SE) by gender (reference: General Internal Medicine; focal: Anesthesiology) – PA subscale

	**Reference group item parameter estimates**	**Reference group SE**	**Focal group item parameter estimates**	**Focal group SE**
mbi_pa1.a	0.98	0.12	0.98	0.12
mbi_pa1.b1	-4.53	0.54	-4.53	0.54
mbi_pa1.b2	-3.22	0.35	-3.22	0.35
mbi_pa1.b3	-2.46	0.26	-2.46	0.26
mbi_pa1.b4	-0.96	0.13	-0.96	0.13
mbi_pa2.a	1.62	0.17	1.62	0.17
mbi_pa2.b1	-2.52	0.22	-2.52	0.22
mbi_pa2.b2	-2.12	0.18	-2.12	0.18
mbi_pa2.b3	-0.86	0.10	-0.86	0.10
mbi_pa3.a	2.44	0.22	2.44	0.22
mbi_pa3.b1	-2.35	0.17	-2.35	0.17
mbi_pa3.b2	-1.98	0.14	-1.98	0.14
mbi_pa3.b3	-1.45	0.10	-1.45	0.10
mbi_pa3.b4	-1.07	0.09	-1.07	0.09

Brady et al. (2021)

mbi_pa3.b5	-0.13	0.07	-0.13	0.07
mbi_pa4.a	1.60	0.15	1.30	0.20
mbi_pa4.b1	-2.74	0.26	-3.78	0.57
mbi_pa4.b2	-2.26	0.20	-2.87	0.39
mbi_pa4.b3	-1.67	0.15	-2.16	0.28
mbi_pa4.b4	-0.86	0.10	-1.19	0.18
mbi_pa4.b5	-0.29	0.09	-0.61	0.15
mbi_pa4.b6	1.12	0.12	1.39	0.28
mbi_pa5.a	1.50	0.17	1.93	0.31
mbi_pa5.b1	-2.63	0.26	-2.53	0.30
mbi_pa5.b2	-2.06	0.20	-2.04	0.23
mbi_pa5.b3	-1.59	0.16	-1.74	0.20
mbi_pa5.b4	-0.29	0.09	-0.58	0.12
mbi_pa6.a	1.86	0.17	2.20	0.32
mbi_pa6.b1	-2.47	0.21	-2.43	0.27
mbi_pa6.b2	-1.79	0.15	-1.85	0.20
mbi_pa6.b3	-1.40	0.12	-1.46	0.16
mbi_pa6.b4	-0.72	0.09	-0.84	0.12
mbi_pa6.b5	-0.28	0.08	-0.28	0.11
mbi_pa6.b6	0.96	0.10	0.73	0.17
mbi_pa7.a	2.54	0.25	2.63	0.40
mbi_pa7.b1	-2.23	0.17	-2.26	0.24
mbi_pa7.b2	-1.64	0.12	-1.78	0.18
mbi_pa7.b3	-1.18	0.10	-1.27	0.14
mbi_pa7.b4	-0.71	0.08	-0.75	0.11
mbi_pa7.b5	0.27	0.07	0.15	0.12
mbi_pa8.a	1.22	0.13	1.22	0.13
mbi_pa8.b1	-3.65	0.37	-3.65	0.37
mbi_pa8.b2	-2.90	0.28	-2.90	0.28
mbi_pa8.b3	-2.18	0.20	-2.18	0.20
mbi_pa8.b4	-1.66	0.16	-1.66	0.16
mbi_pa8.b5	-0.12	0.09	-0.12	0.09
Latent Mean	0.00	NA	-0.27	0.11
Latent Variance	1.00	NA	1.12	0.22

Figure 3.47 Differential item and test functioning by specialty (General Internal Medicine and Emergency Medicine) – PA subscale

a) Expected total score functions for reference (General Internal Medicine) and focal (Emergency Medicine) groups

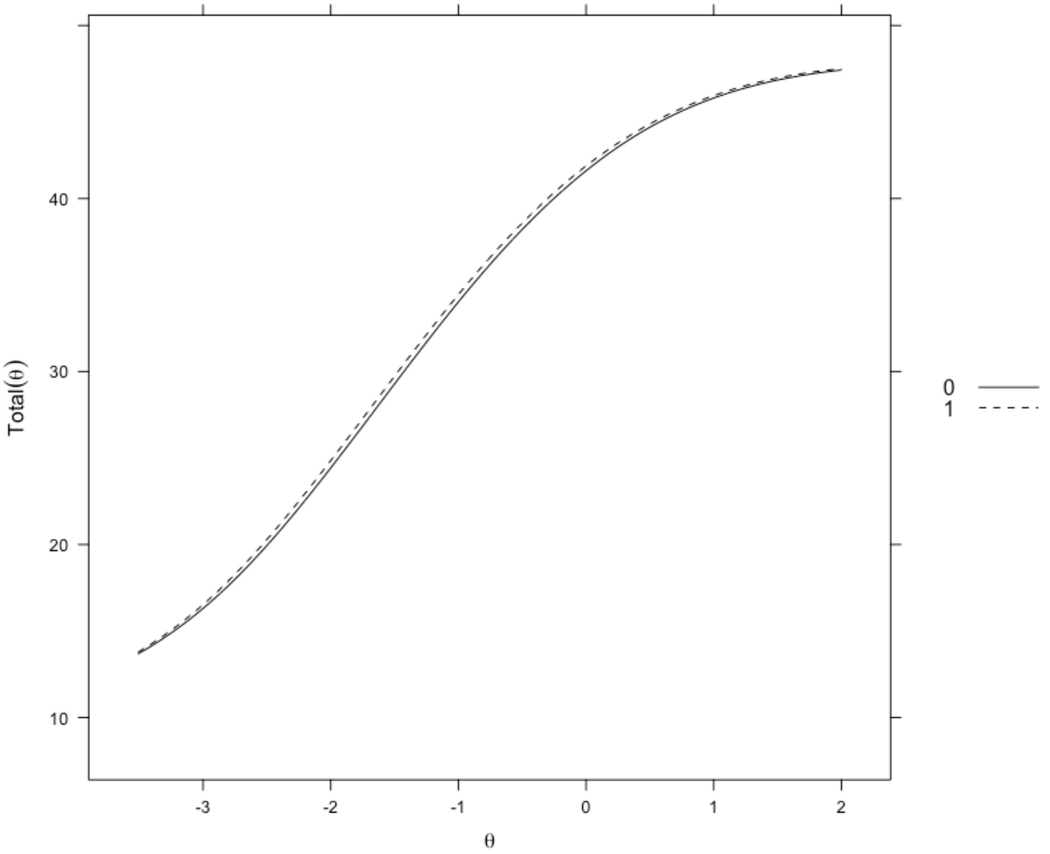

b) Expected item score functions for reference (General Internal Medicine) and focal (Emergency Medicine) groups

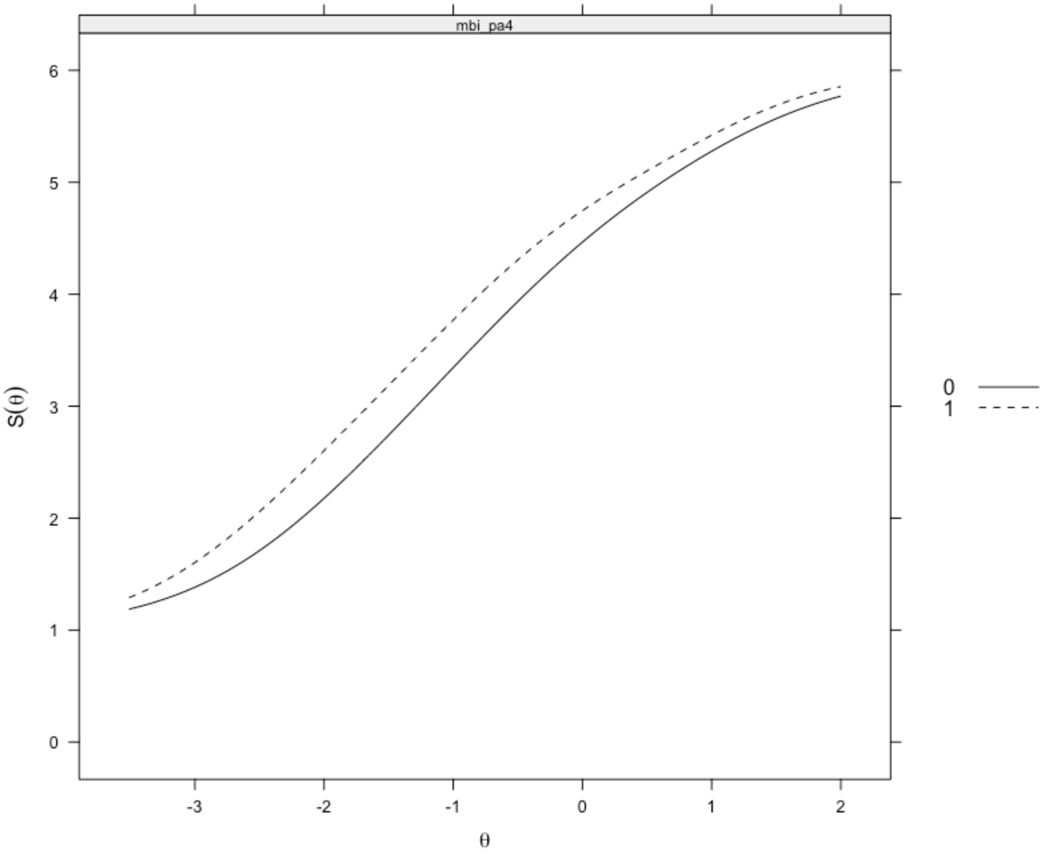

Table 3.47 Multi-group IRT item parameter estimates and standard errors (SE) by gender (reference: General Internal Medicine; focal: Emergency Medicine) – PA subscale

	**Reference group item parameter estimates**	**Reference group SE**	**Focal group item parameter estimates**	**Focal group SE**
mbi_pa1.a	0.97	0.11	0.97	0.11
mbi_pa1.b1	-4.36	0.47	-4.36	0.47
mbi_pa1.b2	-3.02	0.30	-3.02	0.30
mbi_pa1.b3	-2.34	0.23	-2.34	0.23
mbi_pa1.b4	-0.90	0.12	-0.90	0.12
mbi_pa2.a	1.57	0.15	1.57	0.15
mbi_pa2.b1	-2.63	0.20	-2.63	0.20
mbi_pa2.b2	-2.10	0.16	-2.10	0.16
mbi_pa2.b3	-0.76	0.08	-0.76	0.08
mbi_pa3.a	2.33	0.19	2.33	0.19

mbi_pa3.b1	-2.80	0.20	-2.80	0.20
mbi_pa3.b2	-2.14	0.14	-2.14	0.14
mbi_pa3.b3	-1.45	0.10	-1.45	0.10
mbi_pa3.b4	-1.04	0.08	-1.04	0.08
mbi_pa3.b5	-0.15	0.07	-0.15	0.07
mbi_pa4.a	1.62	0.15	1.91	0.21
mbi_pa4.b1	-2.27	0.20	-2.83	0.26
mbi_pa4.b2	-1.67	0.15	-2.06	0.17
mbi_pa4.b3	-0.85	0.10	-1.22	0.12
mbi_pa4.b4	-0.28	0.09	-0.54	0.09
mbi_pa4.b5	1.11	0.12	1.02	0.15
mbi_pa5.a	1.59	0.13	1.59	0.13
mbi_pa5.b1	-3.48	0.28	-3.48	0.28
mbi_pa5.b2	-2.70	0.20	-2.70	0.20
mbi_pa5.b3	-2.41	0.17	-2.41	0.17
mbi_pa5.b4	-1.82	0.13	-1.82	0.13
mbi_pa5.b5	-1.38	0.11	-1.38	0.11
mbi_pa5.b6	-0.25	0.08	-0.25	0.08
mbi_pa6.a	2.07	0.15	2.07	0.15
mbi_pa6.b1	-2.49	0.16	-2.49	0.16
mbi_pa6.b2	-1.81	0.12	-1.81	0.12
mbi_pa6.b3	-1.35	0.09	-1.35	0.09
mbi_pa6.b4	-0.68	0.07	-0.68	0.07
mbi_pa6.b5	-0.23	0.07	-0.23	0.07
mbi_pa6.b6	0.93	0.09	0.93	0.09
mbi_pa7.a	2.50	0.20	2.50	0.20
mbi_pa7.b1	-2.32	0.15	-2.32	0.15
mbi_pa7.b2	-1.71	0.11	-1.71	0.11
mbi_pa7.b3	-1.21	0.09	-1.21	0.09
mbi_pa7.b4	-0.69	0.07	-0.69	0.07
mbi_pa7.b5	0.26	0.07	0.26	0.07
mbi_pa8.a	1.21	0.11	1.21	0.11
mbi_pa8.b1	-3.97	0.37	-3.97	0.37
mbi_pa8.b2	-3.24	0.28	-3.24	0.28
mbi_pa8.b3	-2.38	0.20	-2.38	0.20
mbi_pa8.b4	-1.83	0.16	-1.83	0.16
mbi_pa8.b5	-0.32	0.09	-0.32	0.09

Brady et al. (2021)

Latent Mean	0.00	NA	-0.35	0.08
Latent Variance	1.00	NA	1.05	0.14

Figure 3.48 Differential item and test functioning by specialty (General Internal Medicine and Family Medicine) – PA subscale

a) Expected total score functions for reference (General Internal Medicine) and focal (Family Medicine) groups

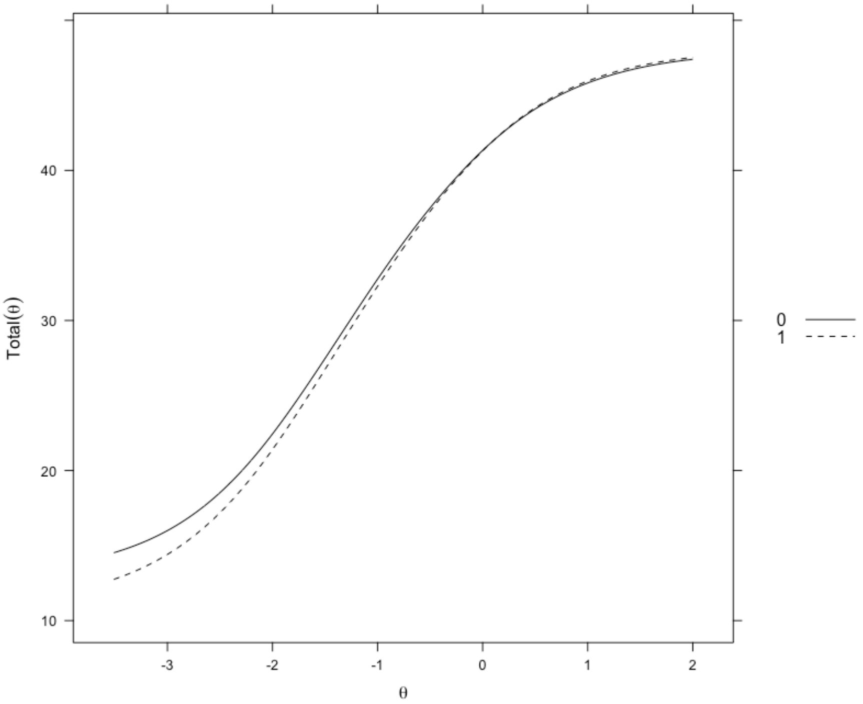

b) Expected item score functions for reference (General Internal Medicine) and focal (Family Medicine) groups

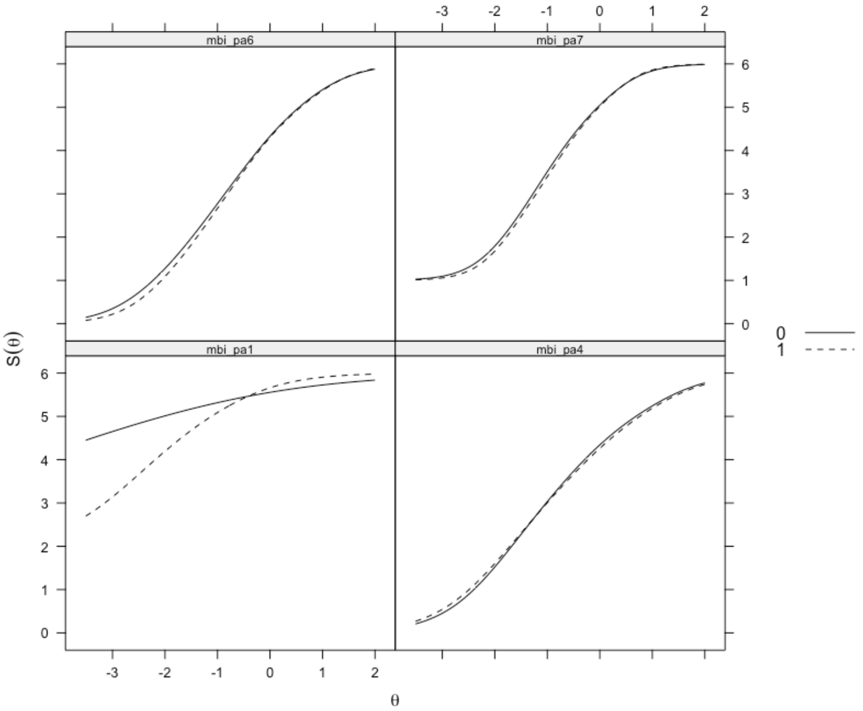

Table 3.48 Multi-group IRT item parameter estimates and standard errors (SE) by gender (reference: General Internal Medicine; focal: Family Medicine) – PA subscale

	**Reference group item parameter estimates**	**Reference group SE**	**Focal group item parameter estimates**	**Focal group SE**
mbi_pa1.a	0.75	0.14	1.02	0.15
mbi_pa1.b1	-5.61	1.02	-4.02	0.60
mbi_pa1.b2	-3.91	0.67	-3.21	0.45
mbi_pa1.b3	-3.07	0.51	-2.55	0.35
mbi_pa1.b4	-1.27	0.24	-0.90	0.16
mbi_pa2.a	1.58	0.14	1.58	0.14
mbi_pa2.b1	-2.94	0.25	-2.94	0.25
mbi_pa2.b2	-2.48	0.20	-2.48	0.20
mbi_pa2.b3	-1.98	0.16	-1.98	0.16
mbi_pa2.b4	-0.71	0.08	-0.71	0.08
mbi_pa3.a	2.65	0.23	2.65	0.23
mbi_pa3.b1	-2.08	0.14	-2.08	0.14
mbi_pa3.b2	-1.45	0.10	-1.45	0.10
mbi_pa3.b3	-1.03	0.08	-1.03	0.08

Brady et al. (2021)

mbi_pa3.b4	-0.13	0.07	-0.13	0.07
mbi_pa4.a	1.59	0.15	1.25	0.15
mbi_pa4.b1	-2.75	0.26	-3.19	0.39
mbi_pa4.b2	-2.28	0.20	-2.63	0.31
mbi_pa4.b3	-1.68	0.15	-1.87	0.22
mbi_pa4.b4	-0.86	0.10	-0.86	0.13
mbi_pa4.b5	-0.29	0.09	-0.23	0.11
mbi_pa4.b6	1.12	0.12	1.50	0.19
mbi_pa5.a	1.61	0.14	1.61	0.14
mbi_pa5.b1	-2.63	0.21	-2.63	0.21
mbi_pa5.b2	-1.94	0.15	-1.94	0.15
mbi_pa5.b3	-1.54	0.12	-1.54	0.12
mbi_pa5.b4	-0.34	0.08	-0.34	0.08
mbi_pa6.a	1.84	0.17	1.76	0.20
mbi_pa6.b1	-2.49	0.21	-2.65	0.29
mbi_pa6.b2	-1.80	0.15	-1.84	0.19
mbi_pa6.b3	-1.40	0.12	-1.26	0.14
mbi_pa6.b4	-0.72	0.09	-0.58	0.10
mbi_pa6.b5	-0.28	0.08	-0.16	0.09
mbi_pa6.b6	0.96	0.10	1.21	0.16
mbi_pa7.a	2.55	0.25	2.44	0.29
mbi_pa7.b1	-2.23	0.17	-2.21	0.22
mbi_pa7.b2	-1.63	0.12	-1.72	0.17
mbi_pa7.b3	-1.17	0.10	-1.10	0.12
mbi_pa7.b4	-0.70	0.08	-0.70	0.09
mbi_pa7.b5	0.26	0.07	0.36	0.10
mbi_pa8.a	1.31	0.12	1.31	0.12
mbi_pa8.b1	-3.04	0.27	-3.04	0.27
mbi_pa8.b2	-2.16	0.19	-2.16	0.19
mbi_pa8.b3	-1.62	0.14	-1.62	0.14
mbi_pa8.b4	-0.13	0.08	-0.13	0.08
Latent Mean	0.00	NA	0.20	0.09
Latent Variance	1.00	NA	1.08	0.17

Figure 3.49 Differential item and test functioning by specialty (General Internal Medicine and General Pediatrics) – PA subscale

a) Expected total score functions for reference (General Internal Medicine) and focal (General Pediatrics) groups

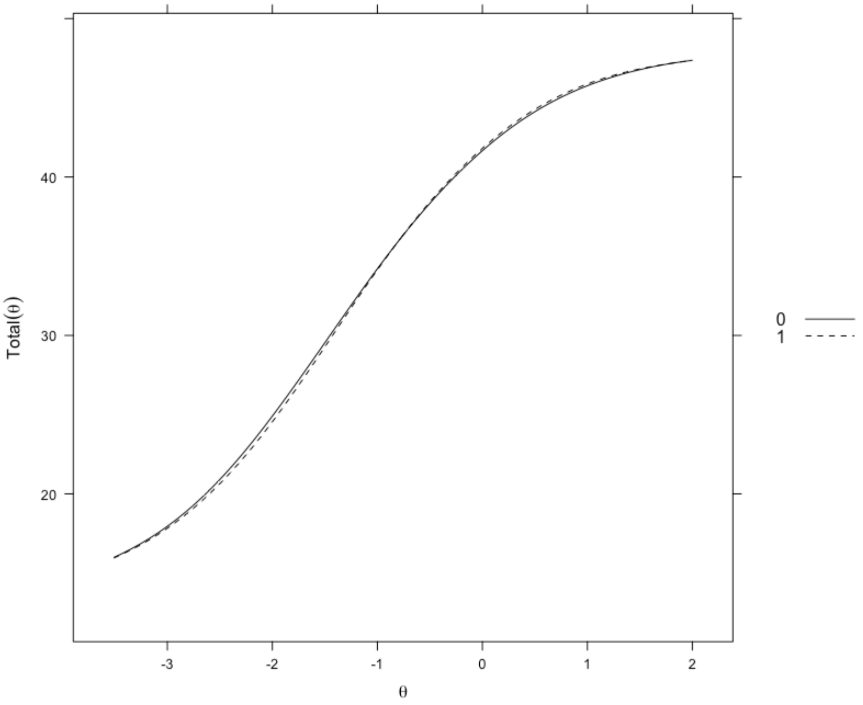

b) Expected item score functions for reference (General Internal Medicine) and focal (General Pediatrics) groups

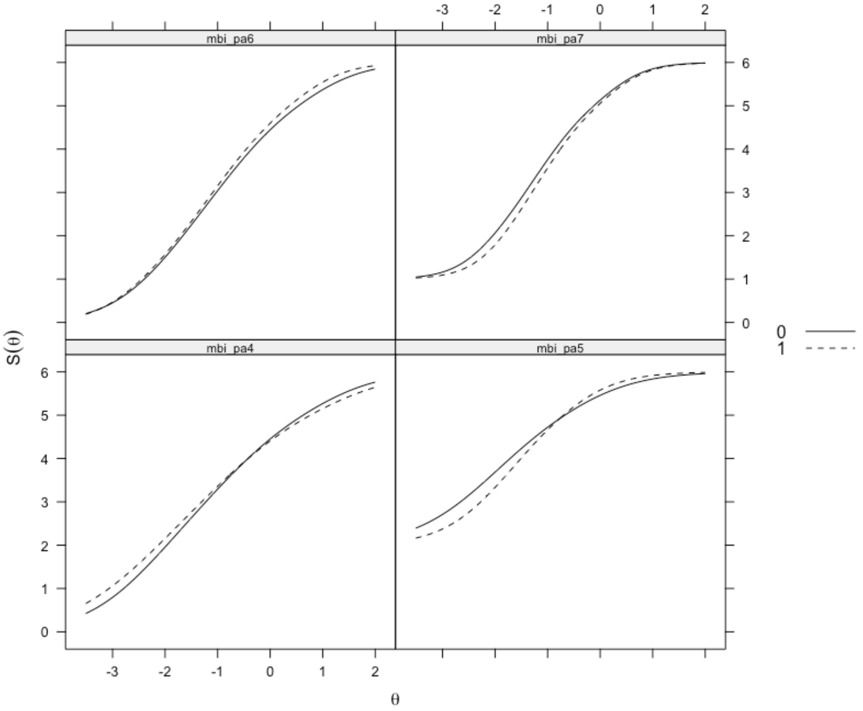

Table 3.49 Multi-group IRT item parameter estimates and standard errors (SE) by gender (reference: General Internal Medicine; focal: General Pediatrics) – PA subscale

	**Reference group item parameter estimates**	**Reference group SE**	**Focal group item parameter estimates**	**Focal group SE**
mbi_pa1.a	0.84	0.11	0.84	0.11
mbi_pa1.b1	-3.68	0.46	-3.68	0.46
mbi_pa1.b2	-2.86	0.35	-2.86	0.35
mbi_pa1.b3	-1.24	0.17	-1.24	0.17
mbi_pa2.a	1.64	0.16	1.64	0.16
mbi_pa2.b1	-2.51	0.21	-2.51	0.21
mbi_pa2.b2	-2.05	0.17	-2.05	0.17
mbi_pa2.b3	-0.81	0.09	-0.81	0.09
mbi_pa3.a	2.64	0.23	2.64	0.23
mbi_pa3.b1	-2.01	0.14	-2.01	0.14
mbi_pa3.b2	-1.37	0.10	-1.37	0.10
mbi_pa3.b3	-1.01	0.08	-1.01	0.08
mbi_pa3.b4	-0.14	0.07	-0.14	0.07
mbi_pa4.a	1.59	0.15	1.32	0.18

mbi_pa4.b1	-2.75	0.26	-2.88	0.38
mbi_pa4.b2	-2.27	0.20	-2.53	0.32
mbi_pa4.b3	-1.68	0.15	-1.72	0.22
mbi_pa4.b4	-0.86	0.10	-0.94	0.14
mbi_pa4.b5	-0.29	0.09	-0.39	0.12
mbi_pa4.b6	1.12	0.12	1.30	0.21
mbi_pa5.a	1.48	0.17	1.84	0.26
mbi_pa5.b1	-2.66	0.27	-2.33	0.27
mbi_pa5.b2	-2.08	0.20	-1.74	0.20
mbi_pa5.b3	-1.60	0.16	-1.39	0.16
mbi_pa5.b4	-0.29	0.09	-0.47	0.11
mbi_pa6.a	1.84	0.17	2.17	0.28
mbi_pa6.b1	-2.49	0.21	-2.70	0.32
mbi_pa6.b2	-1.80	0.15	-1.92	0.21
mbi_pa6.b3	-1.40	0.12	-1.33	0.15
mbi_pa6.b4	-0.72	0.09	-0.88	0.11
mbi_pa6.b5	-0.28	0.08	-0.25	0.10
mbi_pa6.b6	0.96	0.10	0.75	0.14
mbi_pa7.a	2.57	0.26	2.53	0.34
mbi_pa7.b1	-2.23	0.17	-1.90	0.20
mbi_pa7.b2	-1.63	0.12	-1.52	0.16
mbi_pa7.b3	-1.17	0.10	-1.09	0.12
mbi_pa7.b4	-0.70	0.08	-0.62	0.10
mbi_pa7.b5	0.26	0.07	0.31	0.11
mbi_pa8.a	1.25	0.12	1.25	0.12
mbi_pa8.b1	-3.01	0.28	-3.01	0.28
mbi_pa8.b2	-2.17	0.20	-2.17	0.20
mbi_pa8.b3	-1.58	0.15	-1.58	0.15
mbi_pa8.b4	-0.10	0.09	-0.10	0.09
Latent Mean	0.00	NA	0.05	0.10
Latent Variance	1.00	NA	1.08	0.20

Figure 3.50 Differential item and test functioning by specialty (General Internal Medicine and General Surgery) – PA subscale

a) Expected total score functions for reference (General Internal Medicine) and focal (General Surgery) groups

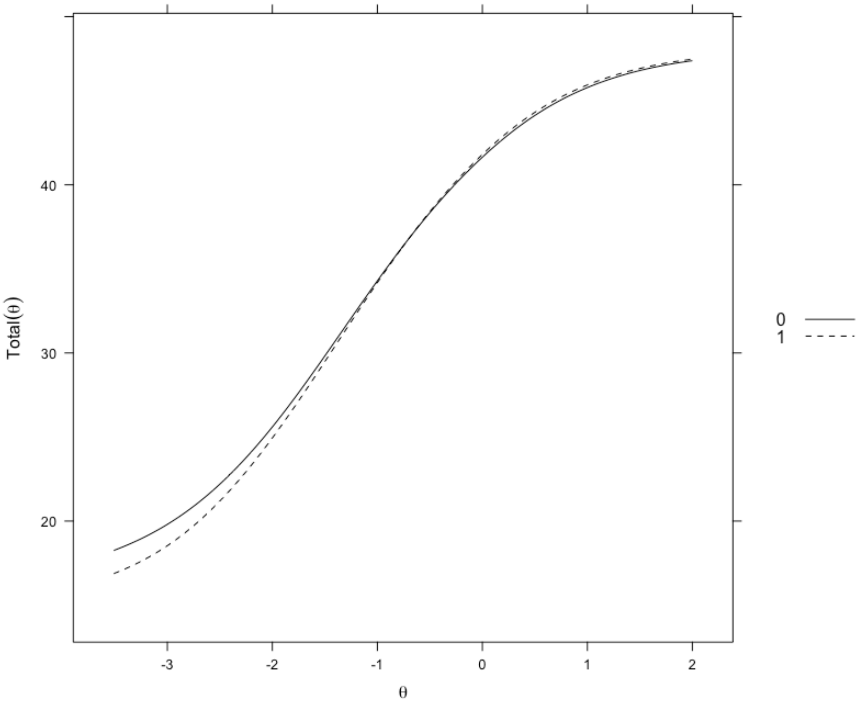

b) Expected item score functions for reference (General Internal Medicine) and focal (General Surgery) groups

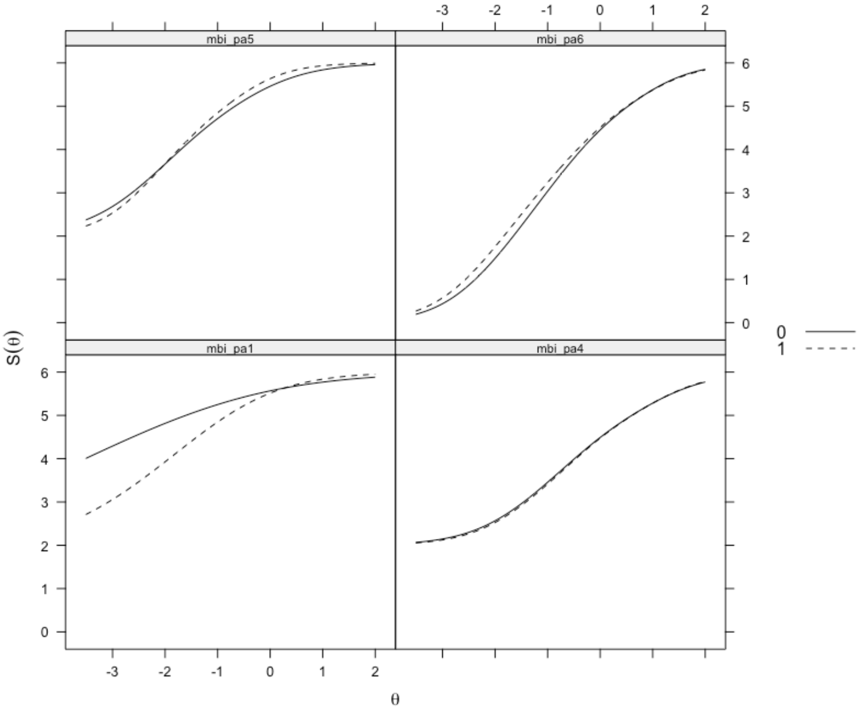

Table 3.50 Multi-group IRT item parameter estimates and standard errors (SE) by gender (reference: General Internal Medicine; focal: General Surgery) – PA subscale

	**Reference group item parameter estimates**	**Reference group SE**	**Focal group item parameter estimates**	**Focal group SE**
mbi_pa1.a	0.74	0.13	1.26	0.25
mbi_pa1.b1	-5.69	1.05	-3.35	0.63
mbi_pa1.b2	-3.97	0.69	-2.11	0.36
mbi_pa1.b3	-3.11	0.53	-1.63	0.28
mbi_pa1.b4	-1.29	0.24	-0.70	0.16
mbi_pa2.a	1.71	0.17	1.71	0.17
mbi_pa2.b1	-2.32	0.20	-2.32	0.20
mbi_pa2.b2	-1.98	0.16	-1.98	0.16
mbi_pa2.b3	-0.72	0.09	-0.72	0.09
mbi_pa3.a	2.25	0.20	2.25	0.20
mbi_pa3.b1	-2.11	0.15	-2.11	0.15
mbi_pa3.b2	-1.48	0.11	-1.48	0.11
mbi_pa3.b3	-1.06	0.09	-1.06	0.09
mbi_pa3.b4	-0.14	0.07	-0.14	0.07

Brady et al. (2021)

mbi_pa4.a	1.61	0.16	1.72	0.25
mbi_pa4.b1	-1.66	0.15	-1.67	0.22
mbi_pa4.b2	-0.85	0.10	-0.79	0.13
mbi_pa4.b3	-0.28	0.09	-0.26	0.11
mbi_pa4.b4	1.11	0.12	1.14	0.19
mbi_pa5.a	1.50	0.17	1.97	0.33
mbi_pa5.b1	-2.63	0.26	-2.59	0.38
mbi_pa5.b2	-2.06	0.20	-2.19	0.30
mbi_pa5.b3	-1.59	0.16	-1.50	0.20
mbi_pa5.b4	-0.29	0.09	-0.44	0.11
mbi_pa6.a	1.86	0.17	1.77	0.26
mbi_pa6.b1	-2.48	0.21	-2.54	0.36
mbi_pa6.b2	-1.79	0.15	-2.02	0.26
mbi_pa6.b3	-1.40	0.12	-1.68	0.21
mbi_pa6.b4	-0.72	0.09	-0.80	0.13
mbi_pa6.b5	-0.28	0.08	-0.32	0.11
mbi_pa6.b6	0.95	0.10	0.96	0.17
mbi_pa7.a	2.82	0.26	2.82	0.26
mbi_pa7.b1	-1.66	0.12	-1.66	0.12
mbi_pa7.b2	-1.19	0.09	-1.19	0.09
mbi_pa7.b3	-0.72	0.07	-0.72	0.07
mbi_pa7.b4	0.24	0.07	0.24	0.07
mbi_pa8.a	1.45	0.14	1.45	0.14
mbi_pa8.b1	-2.65	0.23	-2.65	0.23
mbi_pa8.b2	-1.89	0.16	-1.89	0.16
mbi_pa8.b3	-1.36	0.12	-1.36	0.12
mbi_pa8.b4	-0.06	0.08	-0.06	0.08
Latent Mean	0.00	NA	0.00	0.09
Latent Variance	1.00	NA	0.82	0.15

Figure 3.51 Differential item and test functioning by specialty (General Internal Medicine and General Surgery Subspecialty) – PA subscale

a) Expected total score functions for reference (General Internal Medicine) and focal (General Surgery Subspecialty) groups

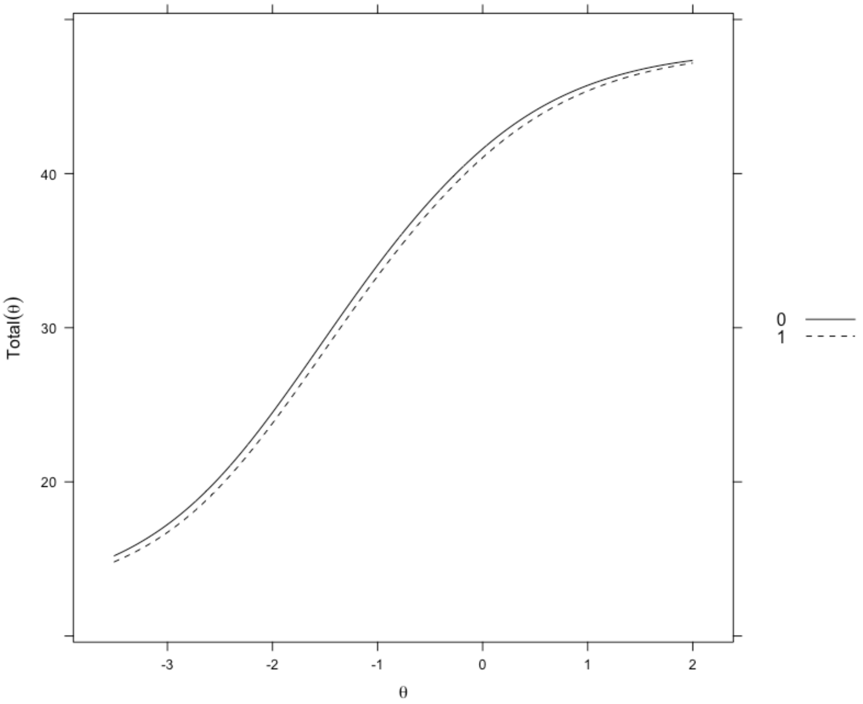

b) Expected item score functions for reference (General Internal Medicine) and focal (General Surgery Subspecialty) groups

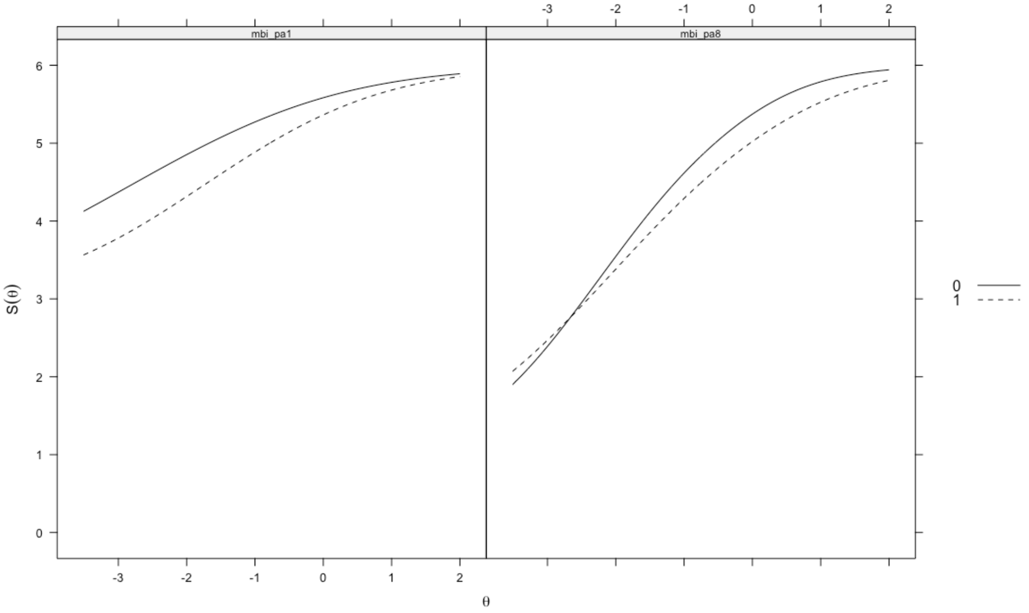

Table 3.51 Multi-group IRT item parameter estimates and standard errors (SE) by gender (reference: General Internal Medicine; focal: General Surgery Subspecialty) – PA subscale

	**Reference group item parameter estimates**	**Reference group SE**	**Focal group item parameter estimates**	**Focal group SE**
mbi_pa1.a	0.77	0.14	0.89	0.16
mbi_pa1.b1	-3.85	0.65	-2.59	0.46
mbi_pa1.b2	-3.03	0.50	-1.90	0.35
mbi_pa1.b3	-1.25	0.23	-0.51	0.17
mbi_pa2.a	1.64	0.16	1.64	0.16
mbi_pa2.b1	-2.27	0.19	-2.27	0.19
mbi_pa2.b2	-1.88	0.15	-1.88	0.15
mbi_pa2.b3	-0.70	0.09	-0.70	0.09
mbi_pa3.a	2.60	0.23	2.60	0.23
mbi_pa3.b1	-2.06	0.14	-2.06	0.14
mbi_pa3.b2	-1.47	0.10	-1.47	0.10
mbi_pa3.b3	-1.04	0.08	-1.04	0.08
mbi_pa3.b4	-0.15	0.06	-0.15	0.06
mbi_pa4.a	1.55	0.12	1.55	0.12
mbi_pa4.b1	-2.69	0.22	-2.69	0.22
mbi_pa4.b2	-2.23	0.17	-2.23	0.17
mbi_pa4.b3	-1.60	0.13	-1.60	0.13
mbi_pa4.b4	-0.79	0.09	-0.79	0.09
mbi_pa4.b5	-0.26	0.08	-0.26	0.08
mbi_pa4.b6	1.18	0.11	1.18	0.11
mbi_pa5.a	1.65	0.15	1.65	0.15
mbi_pa5.b1	-2.32	0.19	-2.32	0.19
mbi_pa5.b2	-1.85	0.15	-1.85	0.15
mbi_pa5.b3	-1.45	0.12	-1.45	0.12
mbi_pa5.b4	-0.32	0.08	-0.32	0.08
mbi_pa6.a	1.82	0.14	1.82	0.14
mbi_pa6.b1	-2.57	0.19	-2.57	0.19
mbi_pa6.b2	-1.81	0.13	-1.81	0.13
mbi_pa6.b3	-1.35	0.11	-1.35	0.11
mbi_pa6.b4	-0.70	0.08	-0.70	0.08
mbi_pa6.b5	-0.29	0.07	-0.29	0.07
mbi_pa6.b6	0.97	0.09	0.97	0.09
mbi_pa7.a	2.36	0.20	2.36	0.20

mbi_pa7.b1	-2.37	0.17	-2.37	0.17
mbi_pa7.b2	-1.79	0.12	-1.79	0.12
mbi_pa7.b3	-1.27	0.10	-1.27	0.10
mbi_pa7.b4	-0.76	0.08	-0.76	0.08
mbi_pa7.b5	0.24	0.07	0.24	0.07
mbi_pa8.a	1.40	0.16	1.09	0.16
mbi_pa8.b1	-3.39	0.38	-3.71	0.59
mbi_pa8.b2	-2.78	0.29	-2.61	0.39
mbi_pa8.b3	-2.00	0.20	-1.89	0.29
mbi_pa8.b4	-1.56	0.16	-1.29	0.22
mbi_pa8.b5	-0.18	0.09	0.35	0.13
Latent Mean	0.00	NA	0.18	0.08
Latent Variance	1.00	NA	0.94	0.13

Figure 3.52 Differential item and test functioning by specialty (General Internal Medicine and Internal Medicine Subspecialty) – PA subscale

a) Expected total score functions for reference (General Internal Medicine) and focal (Internal Medicine Subspecialty) groups

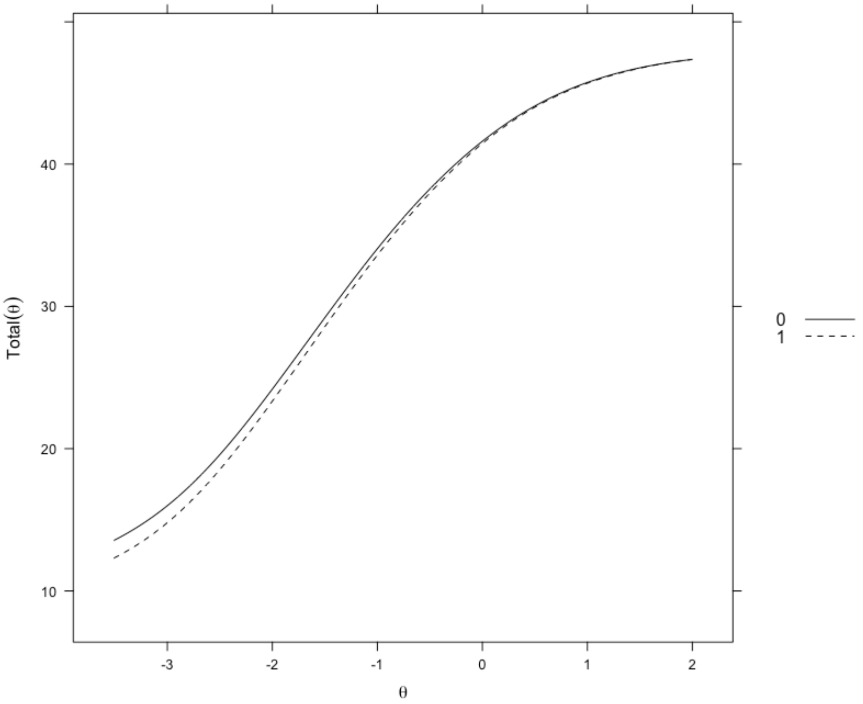

b) Expected item score functions for reference (General Internal Medicine) and focal (Internal Medicine Subspecialty) groups

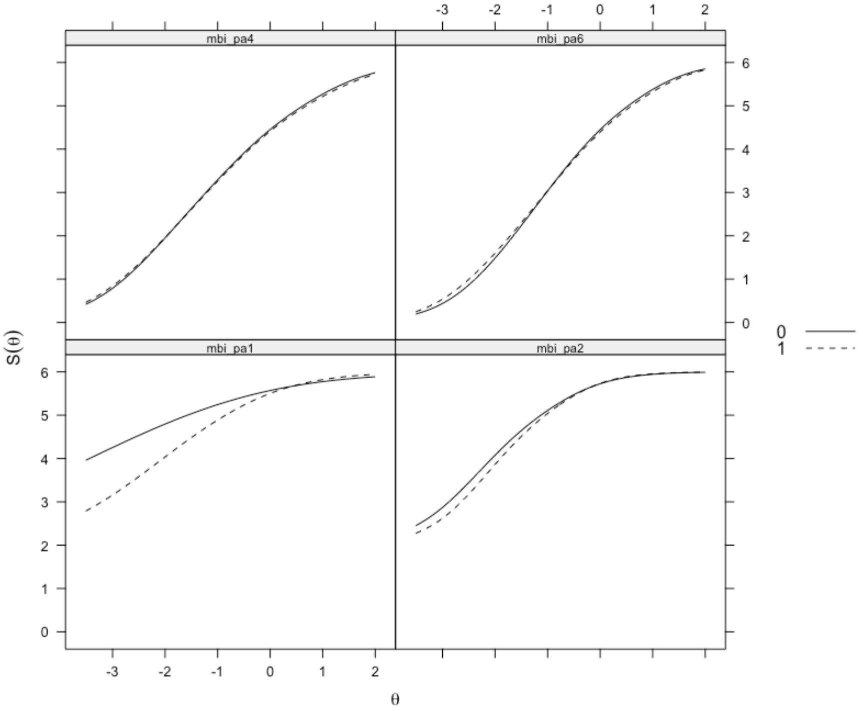

Table 3.52 Multi-group IRT item parameter estimates and standard errors (SE) by gender (reference: General Internal Medicine; focal: Internal Medicine Subspecialty) – PA subscale

	**Reference group item parameter estimates**	**Reference group SE**	**Focal group item parameter estimates**	**Focal group SE**
mbi_pa1.a	0.76	0.14	1.21	0.14
mbi_pa1.b1	-5.56	1.01	-3.35	0.39
mbi_pa1.b2	-3.88	0.66	-2.36	0.26
mbi_pa1.b3	-3.04	0.51	-1.79	0.20
mbi_pa1.b4	-1.26	0.23	-0.60	0.10
mbi_pa2.a	1.75	0.20	1.96	0.21
mbi_pa2.b1	-2.90	0.29	-2.68	0.26
mbi_pa2.b2	-2.37	0.22	-2.22	0.20
mbi_pa2.b3	-1.99	0.18	-1.80	0.16
mbi_pa2.b4	-0.71	0.10	-0.65	0.09
mbi_pa3.a	2.44	0.19	2.44	0.19
mbi_pa3.b1	-2.53	0.17	-2.53	0.17
mbi_pa3.b2	-2.03	0.13	-2.03	0.13
mbi_pa3.b3	-1.45	0.10	-1.45	0.10

Brady et al. (2021)

mbi_pa3.b4	-1.10	0.08	-1.10	0.08
mbi_pa3.b5	-0.15	0.06	-0.15	0.06
mbi_pa4.a	1.60	0.15	1.58	0.15
mbi_pa4.b1	-2.74	0.26	-2.92	0.29
mbi_pa4.b2	-2.27	0.20	-2.19	0.20
mbi_pa4.b3	-1.67	0.15	-1.68	0.16
mbi_pa4.b4	-0.86	0.10	-0.76	0.10
mbi_pa4.b5	-0.29	0.09	-0.32	0.08
mbi_pa4.b6	1.12	0.12	1.24	0.14
mbi_pa5.a	1.60	0.13	1.60	0.13
mbi_pa5.b1	-2.53	0.19	-2.53	0.19
mbi_pa5.b2	-2.03	0.15	-2.03	0.15
mbi_pa5.b3	-1.63	0.12	-1.63	0.12
mbi_pa5.b4	-0.31	0.07	-0.31	0.07
mbi_pa6.a	1.86	0.17	1.87	0.17
mbi_pa6.b1	-2.47	0.21	-2.68	0.25
mbi_pa6.b2	-1.79	0.15	-1.92	0.17
mbi_pa6.b3	-1.40	0.12	-1.40	0.13
mbi_pa6.b4	-0.72	0.09	-0.66	0.09
mbi_pa6.b5	-0.28	0.08	-0.22	0.08
mbi_pa6.b6	0.96	0.10	1.05	0.12
mbi_pa7.a	2.48	0.19	2.48	0.19
mbi_pa7.b1	-2.25	0.15	-2.25	0.15
mbi_pa7.b2	-1.66	0.11	-1.66	0.11
mbi_pa7.b3	-1.17	0.09	-1.17	0.09
mbi_pa7.b4	-0.75	0.07	-0.75	0.07
mbi_pa7.b5	0.26	0.07	0.26	0.07
mbi_pa8.a	1.31	0.11	1.31	0.11
mbi_pa8.b1	-3.73	0.33	-3.73	0.33
mbi_pa8.b2	-2.78	0.23	-2.78	0.23
mbi_pa8.b3	-1.96	0.16	-1.96	0.16
mbi_pa8.b4	-1.42	0.12	-1.42	0.12
mbi_pa8.b5	-0.07	0.07	-0.07	0.07
Latent Mean	0.00	NA	0.09	0.07
Latent Variance	1.00	NA	0.85	0.11

Brady et al. (2021)

Figure 3.53 Differential item and test functioning by specialty (General Internal Medicine and Neurology) – PA subscale

a) Expected total score functions for reference (General Internal Medicine) and focal (Neurology) groups

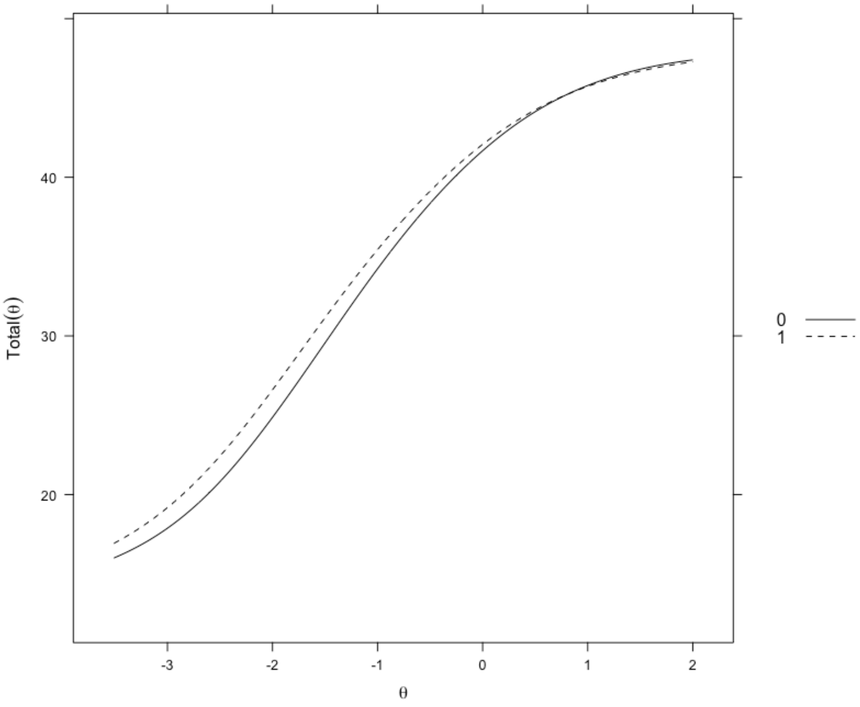

b) Expected item score functions for reference (General Internal Medicine) and focal (Neurology) groups

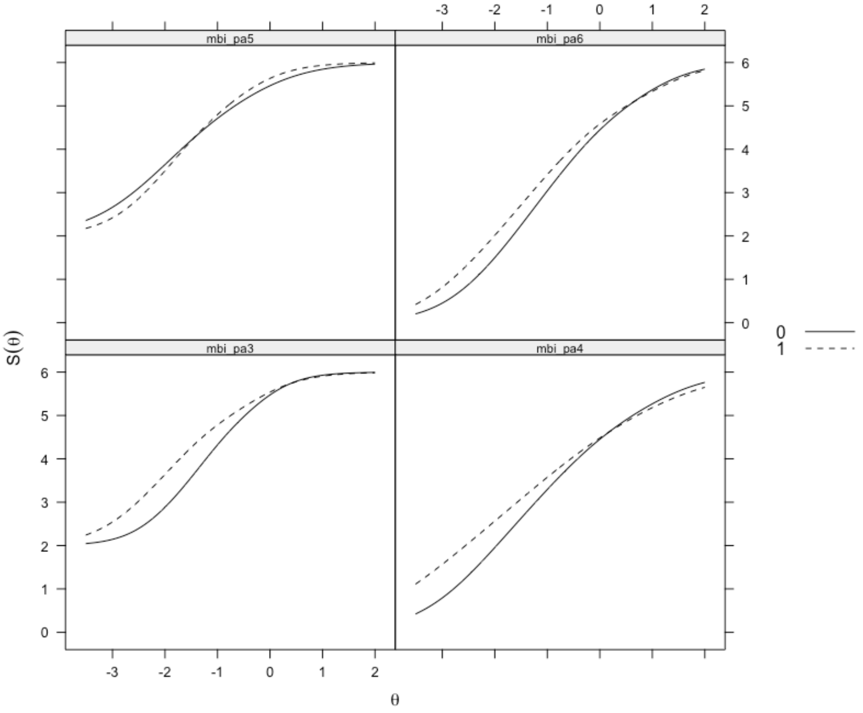

Table 3.53 Multi-group IRT item parameter estimates and standard errors (SE) by gender (reference: General Internal Medicine; focal: Neurology) – PA subscale

	**Reference group item parameter estimates**	**Reference group SE**	**Focal group item parameter estimates**	**Focal group SE**
mbi_pa1.a	0.91	0.12	0.91	0.12
mbi_pa1.b1	-4.47	0.56	-4.47	0.56
mbi_pa1.b2	-3.36	0.40	-3.36	0.40
mbi_pa1.b3	-2.63	0.31	-2.63	0.31
mbi_pa1.b4	-1.00	0.14	-1.00	0.14
mbi_pa2.a	1.82	0.18	1.82	0.18
mbi_pa2.b1	-2.35	0.19	-2.35	0.19
mbi_pa2.b2	-1.91	0.15	-1.91	0.15
mbi_pa2.b3	-0.67	0.08	-0.67	0.08
mbi_pa3.a	2.38	0.25	1.97	0.31
mbi_pa3.b1	-2.12	0.17	-2.70	0.34
mbi_pa3.b2	-1.44	0.12	-2.02	0.24
mbi_pa3.b3	-1.05	0.10	-1.58	0.19
mbi_pa3.b4	-0.15	0.07	-0.22	0.11

Brady et al. (2021)

mbi_pa4.a	1.59	0.15	1.25	0.19
mbi_pa4.b1	-2.75	0.26	-3.61	0.55
mbi_pa4.b2	-2.27	0.20	-3.10	0.44
mbi_pa4.b3	-1.68	0.15	-2.04	0.28
mbi_pa4.b4	-0.86	0.10	-1.09	0.18
mbi_pa4.b5	-0.29	0.09	-0.35	0.14
mbi_pa4.b6	1.12	0.12	1.20	0.24
mbi_pa5.a	1.52	0.17	1.97	0.32
mbi_pa5.b1	-2.61	0.26	-2.44	0.30
mbi_pa5.b2	-2.05	0.20	-1.95	0.23
mbi_pa5.b3	-1.58	0.16	-1.51	0.18
mbi_pa5.b4	-0.29	0.09	-0.46	0.11
mbi_pa6.a	1.84	0.17	1.85	0.26
mbi_pa6.b1	-2.49	0.21	-3.02	0.39
mbi_pa6.b2	-1.80	0.15	-2.25	0.26
mbi_pa6.b3	-1.40	0.12	-1.66	0.19
mbi_pa6.b4	-0.72	0.09	-0.98	0.14
mbi_pa6.b5	-0.28	0.08	-0.40	0.12
mbi_pa6.b6	0.96	0.10	1.13	0.19
mbi_pa7.a	2.36	0.21	2.36	0.21
mbi_pa7.b1	-2.24	0.16	-2.24	0.16
mbi_pa7.b2	-1.70	0.12	-1.70	0.12
mbi_pa7.b3	-1.25	0.10	-1.25	0.10
mbi_pa7.b4	-0.81	0.08	-0.81	0.08
mbi_pa7.b5	0.27	0.07	0.27	0.07
mbi_pa8.a	1.44	0.14	1.44	0.14
mbi_pa8.b1	-2.03	0.18	-2.03	0.18
mbi_pa8.b2	-1.51	0.14	-1.51	0.14
mbi_pa8.b3	-0.15	0.08	-0.15	0.08
Latent Mean	0.00	NA	-0.08	0.10
Latent Variance	1.00	NA	1.13	0.21

Figure 3.54 Differential item and test functioning by specialty (General Internal Medicine and Obstetrics and Gynecology) – PA subscale

a) Expected total score functions for reference (General Internal Medicine) and focal (Obstetrics and Gynecology) groups

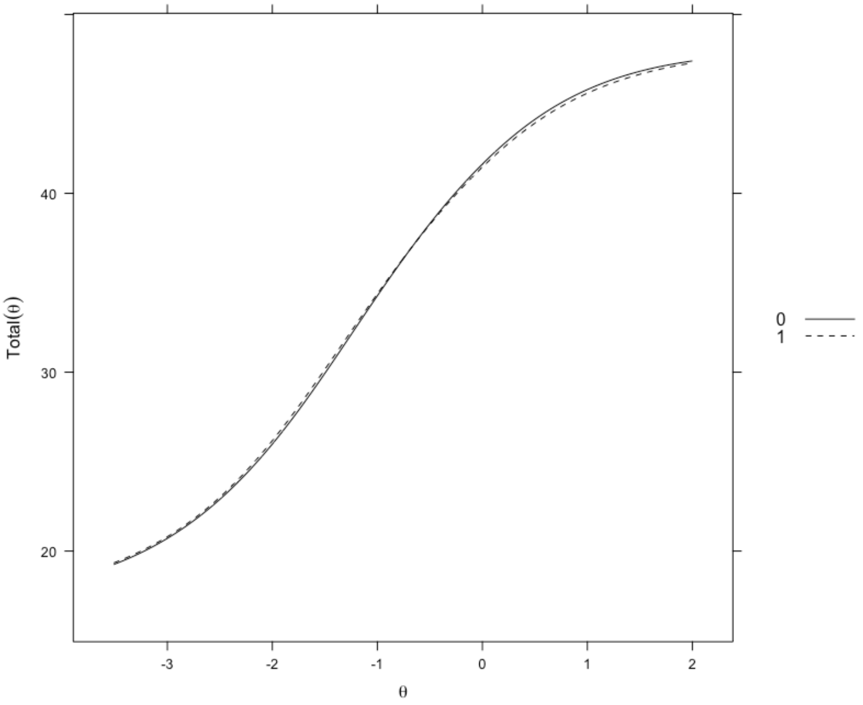

b) Expected item score functions for reference (General Internal Medicine) and focal (Obstetrics and Gynecology) groups

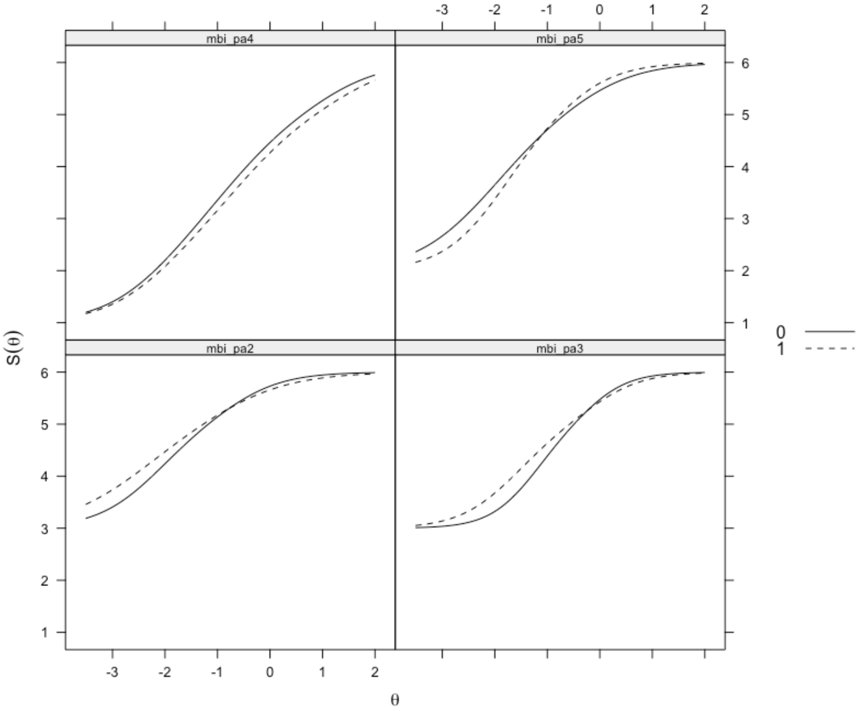

Table 3.54 Multi-group IRT item parameter estimates and standard errors (SE) by gender (reference: General Internal Medicine; focal: Obstetrics and Gynecology) – PA subscale

	Reference group item parameter estimates	Reference group SE	Focal group item parameter estimates	Focal group SE
mbi_pa1.a	0.89	0.11	0.89	0.11
mbi_pa1.b1	-3.29	0.39	-3.29	0.39
mbi_pa1.b2	-2.65	0.31	-2.65	0.31
mbi_pa1.b3	-0.93	0.14	-0.93	0.14
mbi_pa2.a	1.75	0.20	1.29	0.23
mbi_pa2.b1	-2.36	0.22	-2.72	0.43
mbi_pa2.b2	-1.98	0.18	-2.23	0.35
mbi_pa2.b3	-0.71	0.10	-0.82	0.17
mbi_pa3.a	2.36	0.25	2.00	0.29
mbi_pa3.b1	-1.45	0.12	-1.92	0.23
mbi_pa3.b2	-1.05	0.10	-1.29	0.17
mbi_pa3.b3	-0.14	0.07	-0.05	0.10
mbi_pa4.a	1.59	0.15	1.62	0.21
mbi_pa4.b1	-2.29	0.21	-2.30	0.29

mbi_pa4.b2	-1.68	0.15	-1.52	0.20
mbi_pa4.b3	-0.86	0.11	-0.66	0.13
mbi_pa4.b4	-0.29	0.09	-0.09	0.11
mbi_pa4.b5	1.12	0.12	1.46	0.19
mbi_pa5.a	1.52	0.17	1.81	0.28
mbi_pa5.b1	-2.62	0.26	-2.16	0.28
mbi_pa5.b2	-2.05	0.20	-1.87	0.24
mbi_pa5.b3	-1.57	0.16	-1.53	0.20
mbi_pa5.b4	-0.28	0.09	-0.51	0.12
mbi_pa6.a	2.00	0.16	2.00	0.16
mbi_pa6.b1	-1.85	0.14	-1.85	0.14
mbi_pa6.b2	-1.37	0.11	-1.37	0.11
mbi_pa6.b3	-0.67	0.08	-0.67	0.08
mbi_pa6.b4	-0.21	0.07	-0.21	0.07
mbi_pa6.b5	0.92	0.09	0.92	0.09
mbi_pa7.a	2.70	0.24	2.70	0.24
mbi_pa7.b1	-1.64	0.12	-1.64	0.12
mbi_pa7.b2	-1.15	0.09	-1.15	0.09
mbi_pa7.b3	-0.71	0.07	-0.71	0.07
mbi_pa7.b4	0.23	0.07	0.23	0.07
mbi_pa8.a	1.26	0.12	1.26	0.12
mbi_pa8.b1	-2.97	0.28	-2.97	0.28
mbi_pa8.b2	-2.08	0.19	-2.08	0.19
mbi_pa8.b3	-1.58	0.15	-1.58	0.15
mbi_pa8.b4	-0.14	0.09	-0.14	0.09
Latent Mean	0.00	NA	0.21	0.10
Latent Variance	1.00	NA	1.19	0.20

Figure 3.55 Differential item and test functioning by specialty (General Internal Medicine and Ophthalmology) – PA subscale

a) Expected total score functions for reference (General Internal Medicine) and focal (Ophthalmology) groups

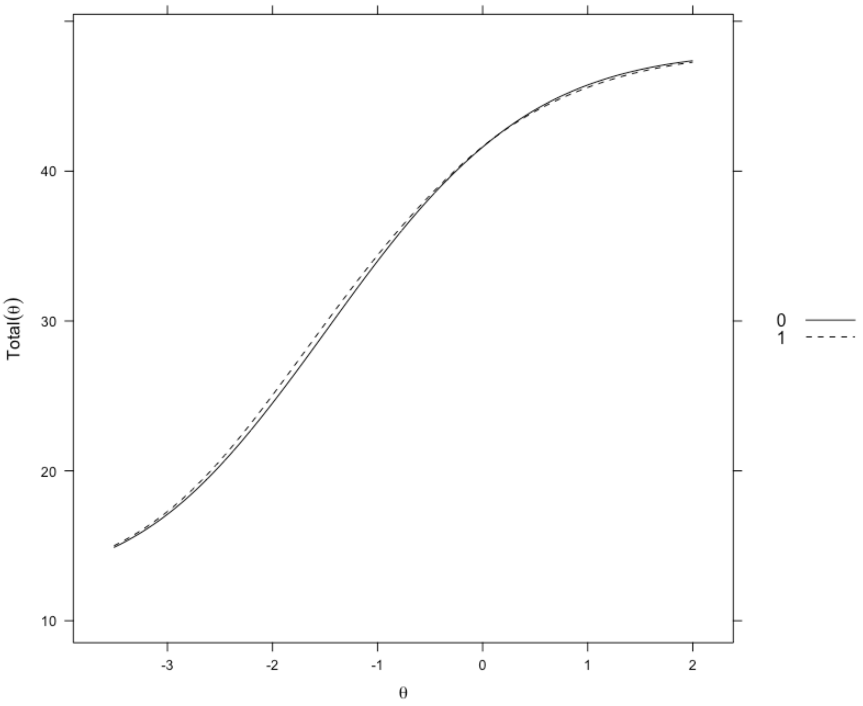

b) Expected item score functions for reference (General Internal Medicine) and focal (Ophthalmology) groups

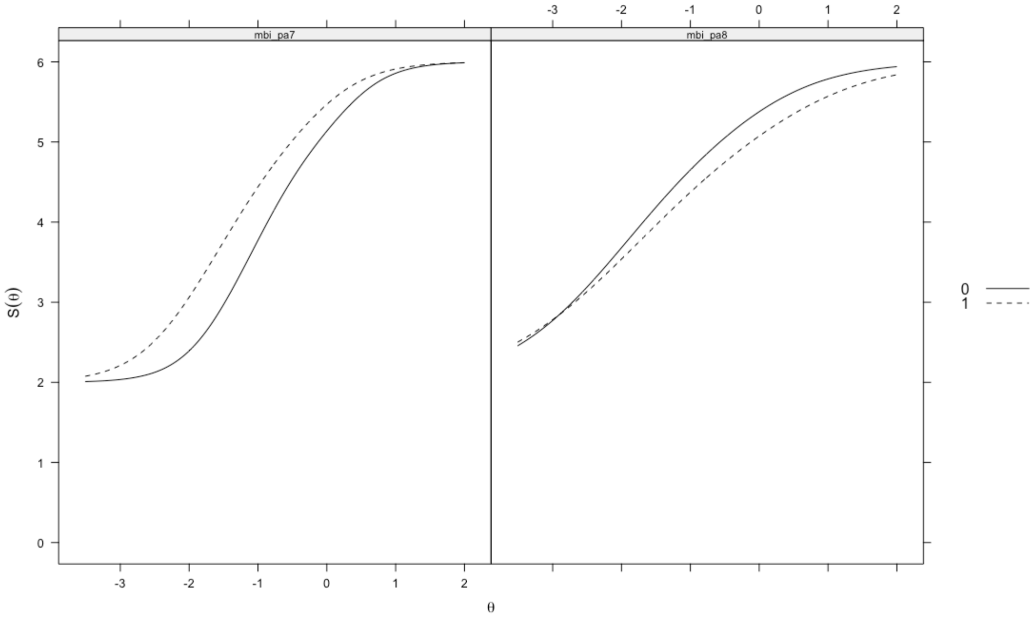

Table 3.55 Multi-group IRT item parameter estimates and standard errors (SE) by gender (reference: General Internal Medicine; focal: Ophthalmology) – PA subscale

	**Reference group item parameter estimates**	**Reference group SE**	**Focal group item parameter estimates**	**Focal group SE**
mbi_pa1.a	0.77	0.11	0.77	0.11
mbi_pa1.b1	-4.99	0.73	-4.99	0.73
mbi_pa1.b2	-3.76	0.53	-3.76	0.53
mbi_pa1.b3	-2.99	0.42	-2.99	0.42
mbi_pa1.b4	-1.19	0.19	-1.19	0.19
mbi_pa2.a	1.51	0.15	1.51	0.15
mbi_pa2.b1	-2.95	0.27	-2.95	0.27
mbi_pa2.b2	-2.55	0.22	-2.55	0.22
mbi_pa2.b3	-2.08	0.18	-2.08	0.18
mbi_pa2.b4	-0.74	0.09	-0.74	0.09
mbi_pa3.a	2.35	0.21	2.35	0.21
mbi_pa3.b1	-2.53	0.19	-2.53	0.19
mbi_pa3.b2	-2.15	0.15	-2.15	0.15
mbi_pa3.b3	-1.48	0.11	-1.48	0.11
mbi_pa3.b4	-1.09	0.09	-1.09	0.09
mbi_pa3.b5	-0.23	0.07	-0.23	0.07
mbi_pa4.a	1.64	0.14	1.64	0.14
mbi_pa4.b1	-2.64	0.22	-2.64	0.22
mbi_pa4.b2	-2.13	0.17	-2.13	0.17
mbi_pa4.b3	-1.59	0.13	-1.59	0.13
mbi_pa4.b4	-0.79	0.09	-0.79	0.09
mbi_pa4.b5	-0.26	0.08	-0.26	0.08
mbi_pa4.b6	1.19	0.11	1.19	0.11
mbi_pa5.a	1.61	0.15	1.61	0.15
mbi_pa5.b1	-2.55	0.22	-2.55	0.22
mbi_pa5.b2	-2.01	0.17	-2.01	0.17
mbi_pa5.b3	-1.49	0.13	-1.49	0.13
mbi_pa5.b4	-0.32	0.08	-0.32	0.08
mbi_pa6.a	1.91	0.15	1.91	0.15
mbi_pa6.b1	-2.45	0.19	-2.45	0.19
mbi_pa6.b2	-1.80	0.13	-1.80	0.13
mbi_pa6.b3	-1.34	0.11	-1.34	0.11
mbi_pa6.b4	-0.73	0.08	-0.73	0.08

Brady et al. (2021)

mbi_pa6.b5	-0.27	0.07	-0.27	0.07
mbi_pa6.b6	0.96	0.09	0.96	0.09
mbi_pa7.a	2.62	0.27	2.16	0.35
mbi_pa7.b1	-1.60	0.12	-2.15	0.29
mbi_pa7.b2	-1.15	0.10	-1.68	0.22
mbi_pa7.b3	-0.70	0.08	-1.12	0.16
mbi_pa7.b4	0.26	0.07	-0.16	0.11
mbi_pa8.a	1.38	0.16	1.19	0.21
mbi_pa8.b1	-2.79	0.29	-2.77	0.48
mbi_pa8.b2	-2.01	0.20	-1.95	0.34
mbi_pa8.b3	-1.56	0.16	-1.21	0.24
mbi_pa8.b4	-0.18	0.09	0.37	0.15
Latent Mean	0.00	NA	0.23	0.10
Latent Variance	1.00	NA	1.04	0.17

Figure 3.56 Differential item and test functioning by specialty (General Internal Medicine and Orthopedic Surgery) – PA subscale

a) Expected total score functions for reference (General Internal Medicine) and focal (Orthopedic Surgery) groups

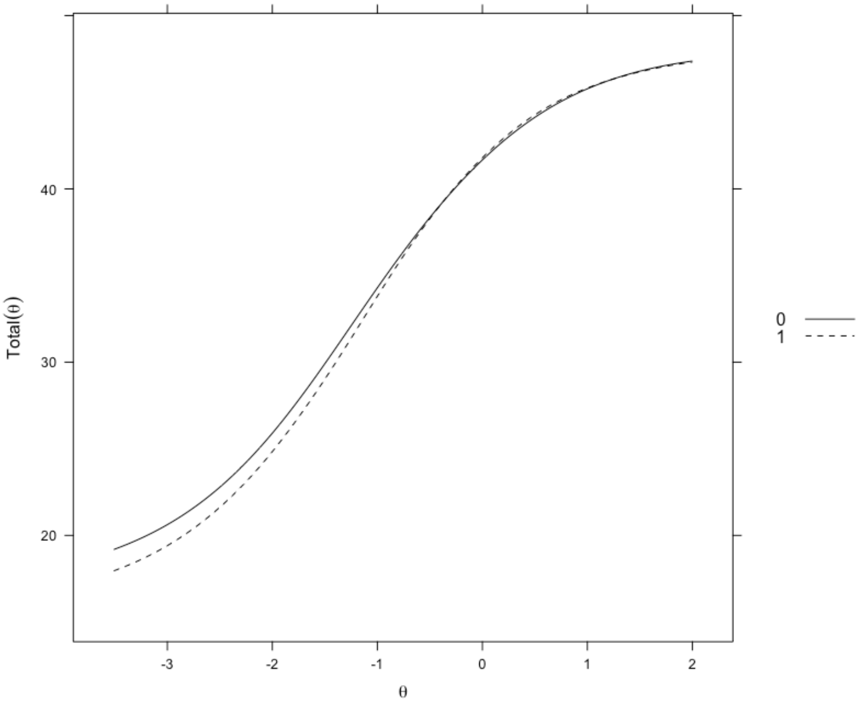

b) Expected item score functions for reference (General Internal Medicine) and focal (Orthopedic Surgery) groups

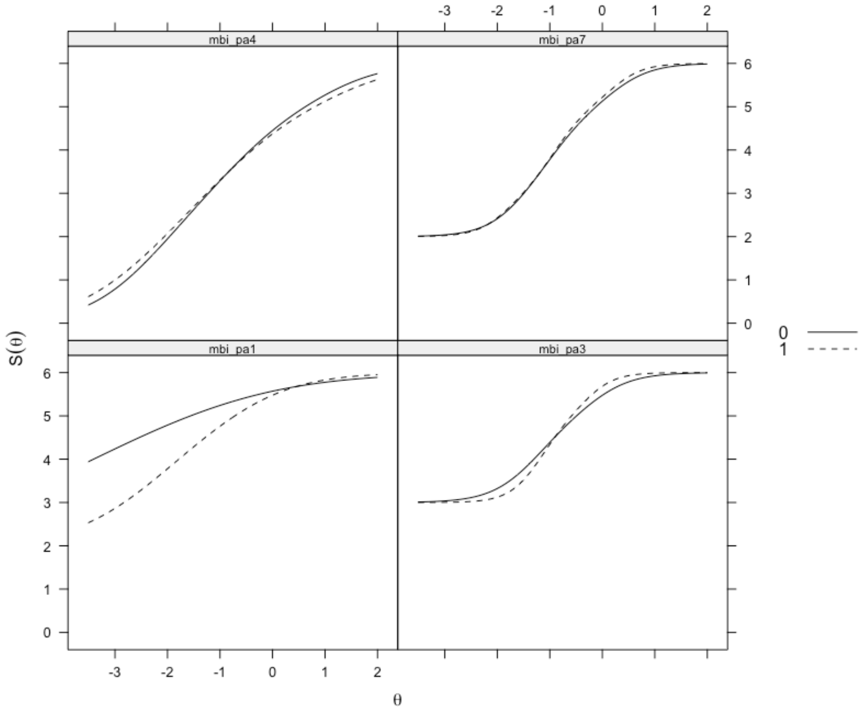

Table 3.56 Multi-group IRT item parameter estimates and standard errors (SE) by gender (reference: General Internal Medicine; focal: Orthopedic Surgery) – PA subscale

	**Reference group item parameter estimates**	**Reference group SE**	**Focal group item parameter estimates**	**Focal group SE**
mbi_pa1.a	0.77	0.14	1.32	0.26
mbi_pa1.b1	-5.51	0.99	-2.90	0.55
mbi_pa1.b2	-3.84	0.64	-2.10	0.39
mbi_pa1.b3	-3.01	0.50	-1.54	0.29
mbi_pa1.b4	-1.24	0.23	-0.55	0.16
mbi_pa2.a	1.93	0.19	1.93	0.19
mbi_pa2.b1	-2.24	0.18	-2.24	0.18
mbi_pa2.b2	-1.94	0.16	-1.94	0.16
mbi_pa2.b3	-0.71	0.09	-0.71	0.09
mbi_pa3.a	2.35	0.25	3.43	0.67
mbi_pa3.b1	-1.45	0.12	-1.31	0.18
mbi_pa3.b2	-1.05	0.10	-1.02	0.15
mbi_pa3.b3	-0.15	0.07	-0.28	0.10

Brady et al. (2021)

mbi_pa4.a	1.60	0.15	1.34	0.22
mbi_pa4.b1	-2.74	0.26	-2.97	0.52
mbi_pa4.b2	-2.26	0.20	-2.27	0.38
mbi_pa4.b3	-1.67	0.15	-1.68	0.29
mbi_pa4.b4	-0.86	0.10	-0.93	0.19
mbi_pa4.b5	-0.29	0.09	-0.36	0.15
mbi_pa4.b6	1.12	0.12	1.37	0.23
mbi_pa5.a	1.66	0.16	1.66	0.16
mbi_pa5.b1	-1.92	0.16	-1.92	0.16
mbi_pa5.b2	-1.49	0.13	-1.49	0.13
mbi_pa5.b3	-0.29	0.08	-0.29	0.08
mbi_pa6.a	1.91	0.16	1.91	0.16
mbi_pa6.b1	-1.79	0.14	-1.79	0.14
mbi_pa6.b2	-1.44	0.12	-1.44	0.12
mbi_pa6.b3	-0.73	0.08	-0.73	0.08
mbi_pa6.b4	-0.28	0.07	-0.28	0.07
mbi_pa6.b5	0.95	0.10	0.95	0.10
mbi_pa7.a	2.55	0.26	3.26	0.56
mbi_pa7.b1	-1.61	0.13	-1.84	0.25
mbi_pa7.b2	-1.16	0.10	-1.03	0.15
mbi_pa7.b3	-0.70	0.08	-0.77	0.12
mbi_pa7.b4	0.26	0.07	0.22	0.10
mbi_pa8.a	1.26	0.13	1.26	0.13
mbi_pa8.b1	-2.82	0.28	-2.82	0.28
mbi_pa8.b2	-2.01	0.19	-2.01	0.19
mbi_pa8.b3	-1.51	0.15	-1.51	0.15
mbi_pa8.b4	-0.11	0.09	-0.11	0.09
Latent Mean	0.00	NA	0.21	0.10
Latent Variance	1.00	NA	0.82	0.16

Figure 3.57 Differential item and test functioning by specialty (General Internal Medicine and Pediatric Subspecialty) – PA subscale

a) Expected total score functions for reference (General Internal Medicine) and focal (Pediatric Subspecialty) groups

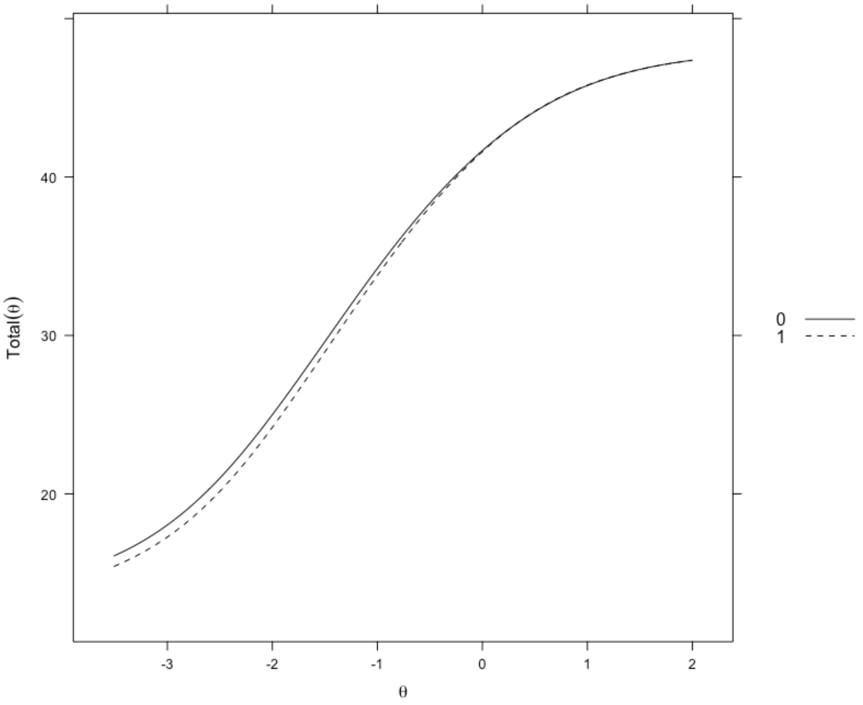

b) Expected item score functions for reference (General Internal Medicine) and focal (Pediatric Subspecialty) groups

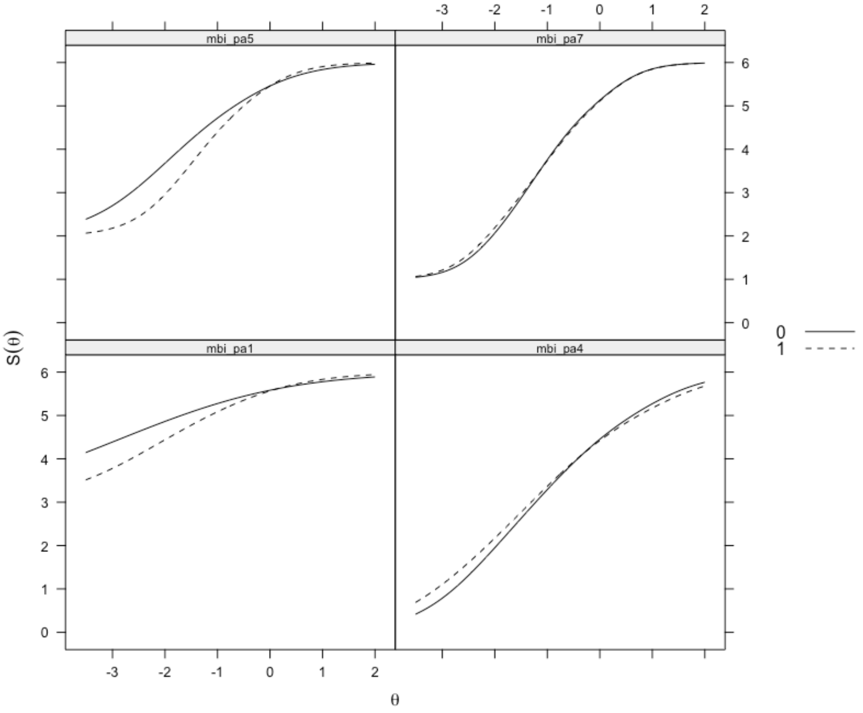

Table 3.57 Multi-group IRT item parameter estimates and standard errors (SE) by gender (reference: General Internal Medicine; focal: Pediatric Subspecialty) – PA subscale

	**Reference group item parameter estimates**	**Reference group SE**	**Focal group item parameter estimates**	**Focal group SE**
mbi_pa1.a	0.75	0.14	1.15	0.20
mbi_pa1.b1	-3.90	0.66	-2.74	0.43
mbi_pa1.b2	-3.06	0.51	-2.22	0.34
mbi_pa1.b3	-1.27	0.24	-0.65	0.15
mbi_pa2.a	1.60	0.16	1.60	0.16
mbi_pa2.b1	-2.44	0.20	-2.44	0.20
mbi_pa2.b2	-1.91	0.16	-1.91	0.16
mbi_pa2.b3	-0.64	0.08	-0.64	0.08
mbi_pa3.a	2.53	0.22	2.53	0.22
mbi_pa3.b1	-2.04	0.14	-2.04	0.14
mbi_pa3.b2	-1.42	0.10	-1.42	0.10
mbi_pa3.b3	-0.99	0.08	-0.99	0.08
mbi_pa3.b4	-0.15	0.07	-0.15	0.07
mbi_pa4.a	1.60	0.15	1.48	0.20

mbi_pa4.b1	-2.74	0.26	-3.31	0.47
mbi_pa4.b2	-2.27	0.20	-2.44	0.31
mbi_pa4.b3	-1.67	0.15	-1.68	0.21
mbi_pa4.b4	-0.86	0.10	-0.97	0.14
mbi_pa4.b5	-0.29	0.09	-0.28	0.11
mbi_pa4.b6	1.12	0.12	1.33	0.20
mbi_pa5.a	1.49	0.17	2.11	0.29
mbi_pa5.b1	-2.65	0.26	-2.01	0.23
mbi_pa5.b2	-2.07	0.20	-1.56	0.17
mbi_pa5.b3	-1.60	0.16	-1.18	0.14
mbi_pa5.b4	-0.29	0.09	-0.17	0.10
mbi_pa6.a	1.90	0.15	1.90	0.15
mbi_pa6.b1	-2.59	0.19	-2.59	0.19
mbi_pa6.b2	-1.92	0.14	-1.92	0.14
mbi_pa6.b3	-1.44	0.11	-1.44	0.11
mbi_pa6.b4	-0.82	0.08	-0.82	0.08
mbi_pa6.b5	-0.34	0.07	-0.34	0.07
mbi_pa6.b6	0.89	0.09	0.89	0.09
mbi_pa7.a	2.57	0.26	2.52	0.33
mbi_pa7.b1	-2.23	0.17	-2.34	0.26
mbi_pa7.b2	-1.63	0.12	-1.77	0.18
mbi_pa7.b3	-1.17	0.10	-1.11	0.12
mbi_pa7.b4	-0.70	0.08	-0.64	0.10
mbi_pa7.b5	0.26	0.07	0.25	0.10
mbi_pa8.a	1.27	0.12	1.27	0.12
mbi_pa8.b1	-3.02	0.28	-3.02	0.28
mbi_pa8.b2	-2.16	0.20	-2.16	0.20
mbi_pa8.b3	-1.61	0.15	-1.61	0.15
mbi_pa8.b4	-0.12	0.08	-0.12	0.08
Latent Mean	0.00	NA	0.03	0.09
Latent Variance	1.00	NA	1.01	0.17

Figure 3.58 Differential item and test functioning by specialty (General Internal Medicine and Physical Medicine and Rehabilitation/Preventive Medicine/Occupational Medicine) – PA subscale

a) Expected total score functions for reference (General Internal Medicine) and focal (Physical Medicine and Rehabilitation/Preventive Medicine/Occupational Medicine) groups

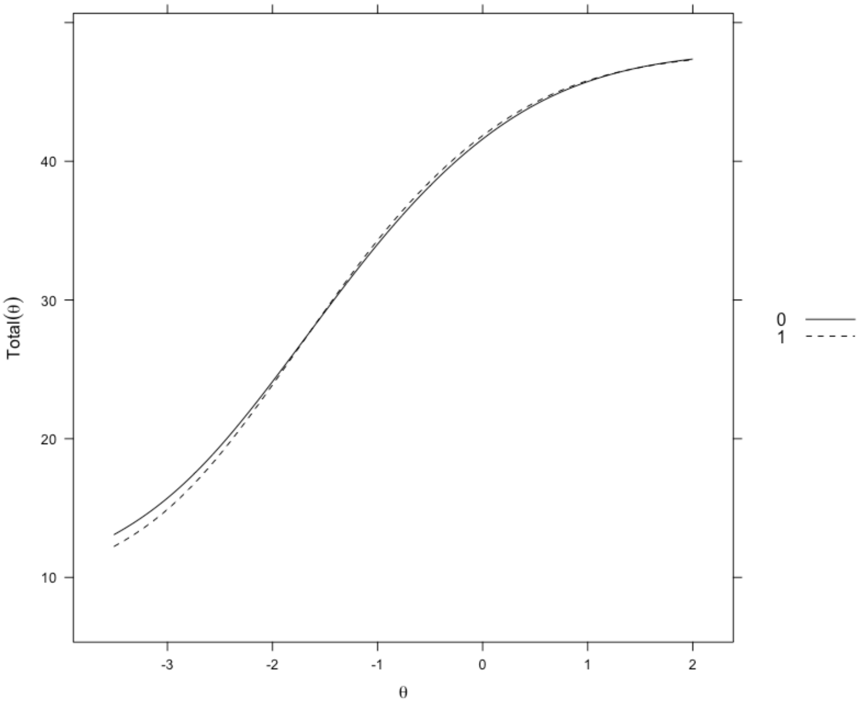

b) Expected item score functions for reference (General Internal Medicine) and focal (Physical Medicine and Rehabilitation/Preventive Medicine/Occupational Medicine) groups

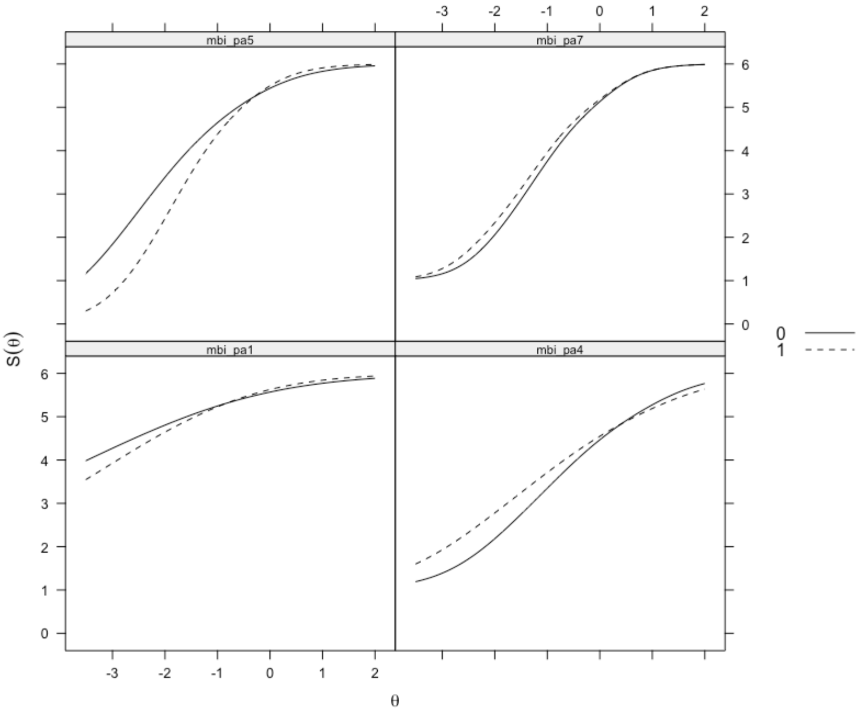

Table 3.58 Multi-group IRT item parameter estimates and standard errors (SE) by gender (reference: General Internal Medicine; focal: Physical Medicine and Rehabilitation/Preventive Medicine/Occupational Medicine) – PA subscale

	**Reference group item parameter estimates**	**Reference group SE**	**Focal group item parameter estimates**	**Focal group SE**
mbi_pa1.a	0.75	0.14	0.99	0.19
mbi_pa1.b1	-5.62	1.03	-4.31	0.79
mbi_pa1.b2	-3.92	0.67	-3.46	0.59
mbi_pa1.b3	-3.07	0.52	-2.59	0.43
mbi_pa1.b4	-1.27	0.24	-1.06	0.21
mbi_pa2.a	1.64	0.16	1.64	0.16
mbi_pa2.b1	-2.76	0.23	-2.76	0.23
mbi_pa2.b2	-2.28	0.18	-2.28	0.18
mbi_pa2.b3	-1.86	0.15	-1.86	0.15
mbi_pa2.b4	-0.71	0.09	-0.71	0.09
mbi_pa3.a	2.48	0.22	2.48	0.22
mbi_pa3.b1	-2.51	0.18	-2.51	0.18

mbi_pa3.b2	-2.10	0.14	-2.10	0.14
mbi_pa3.b3	-1.49	0.11	-1.49	0.11
mbi_pa3.b4	-1.11	0.09	-1.11	0.09
mbi_pa3.b5	-0.19	0.07	-0.19	0.07
mbi_pa4.a	1.60	0.15	1.22	0.18
mbi_pa4.b1	-2.27	0.20	-2.88	0.40
mbi_pa4.b2	-1.67	0.15	-2.36	0.32
mbi_pa4.b3	-0.86	0.10	-1.18	0.18
mbi_pa4.b4	-0.29	0.09	-0.49	0.14
mbi_pa4.b5	1.12	0.12	1.26	0.22
mbi_pa5.a	1.47	0.16	2.01	0.28
mbi_pa5.b1	-3.42	0.37	-2.61	0.29
mbi_pa5.b2	-2.88	0.29	-2.32	0.25
mbi_pa5.b3	-2.70	0.27	-1.95	0.20
mbi_pa5.b4	-2.10	0.20	-1.57	0.17
mbi_pa5.b5	-1.62	0.16	-1.34	0.15
mbi_pa5.b6	-0.30	0.09	-0.27	0.11
mbi_pa6.a	1.94	0.15	1.94	0.15
mbi_pa6.b1	-2.57	0.19	-2.57	0.19
mbi_pa6.b2	-1.84	0.13	-1.84	0.13
mbi_pa6.b3	-1.34	0.10	-1.34	0.10
mbi_pa6.b4	-0.74	0.08	-0.74	0.08
mbi_pa6.b5	-0.28	0.07	-0.28	0.07
mbi_pa6.b6	0.95	0.10	0.95	0.10
mbi_pa7.a	2.57	0.26	2.56	0.34
mbi_pa7.b1	-2.22	0.17	-2.50	0.26
mbi_pa7.b2	-1.63	0.12	-1.81	0.18
mbi_pa7.b3	-1.17	0.10	-1.22	0.13
mbi_pa7.b4	-0.70	0.08	-0.87	0.11
mbi_pa7.b5	0.26	0.07	0.27	0.11
mbi_pa8.a	1.32	0.13	1.32	0.13
mbi_pa8.b1	-3.40	0.32	-3.40	0.32
mbi_pa8.b2	-2.85	0.25	-2.85	0.25
mbi_pa8.b3	-2.10	0.18	-2.10	0.18
mbi_pa8.b4	-1.50	0.14	-1.50	0.14
mbi_pa8.b5	-0.11	0.08	-0.11	0.08
Latent Mean	0.00	NA	-0.04	0.10

Brady et al. (2021)

Latent Variance	1.00	NA	1.19	0.20

Figure 3.59 Differential item and test functioning by specialty (General Internal Medicine and Psychiatry) – PA subscale

a) Expected total score functions for reference (General Internal Medicine) and focal (Psychiatry) groups

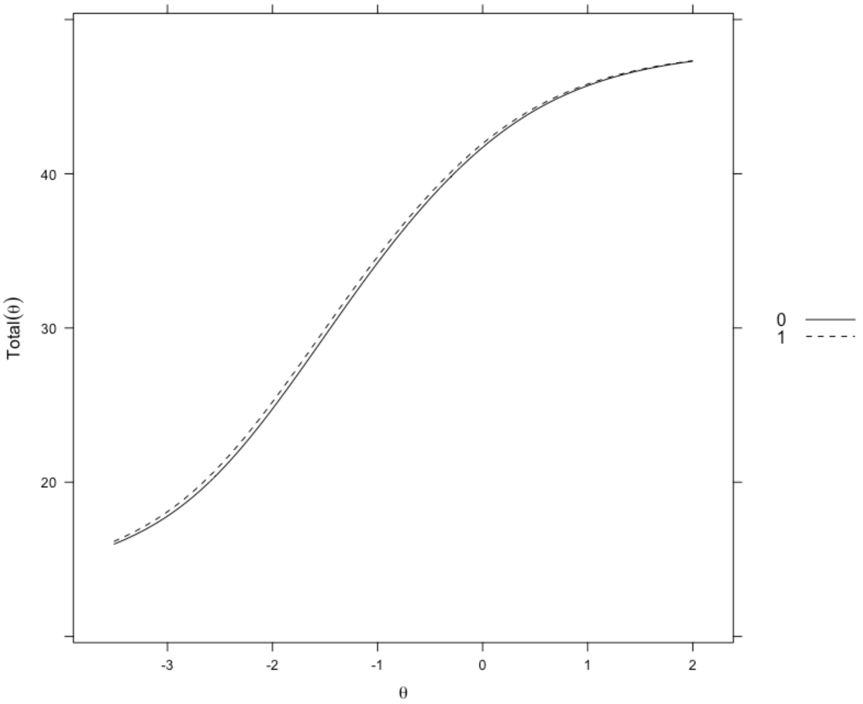

b) Expected item score functions for reference (General Internal Medicine) and focal (Psychiatry) groups

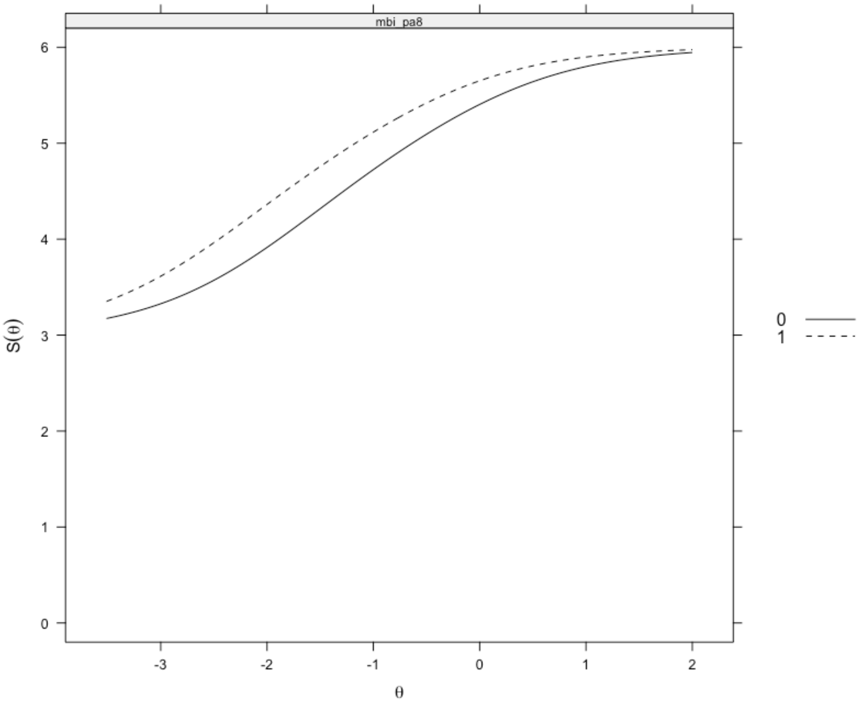

Table 3.59 Multi-group IRT item parameter estimates and standard errors (SE) by gender (reference: General Internal Medicine; focal: Psychiatry) – PA subscale

	**Reference group item parameter estimates**	**Reference group SE**	**Focal group item parameter estimates**	**Focal group SE**
mbi_pa1.a	0.83	0.10	0.83	0.10
mbi_pa1.b1	-5.56	0.71	-5.56	0.71
mbi_pa1.b2	-4.86	0.60	-4.86	0.60
mbi_pa1.b3	-3.63	0.42	-3.63	0.42
mbi_pa1.b4	-2.88	0.33	-2.88	0.33
mbi_pa1.b5	-1.30	0.17	-1.30	0.17
mbi_pa2.a	1.87	0.16	1.87	0.16
mbi_pa2.b1	-2.22	0.16	-2.22	0.16
mbi_pa2.b2	-1.91	0.14	-1.91	0.14
mbi_pa2.b3	-0.71	0.08	-0.71	0.08
mbi_pa3.a	2.52	0.20	2.52	0.20
mbi_pa3.b1	-2.51	0.17	-2.51	0.17
mbi_pa3.b2	-2.13	0.14	-2.13	0.14
mbi_pa3.b3	-1.49	0.10	-1.49	0.10

Brady et al. (2021)

mbi_pa3.b4	-1.16	0.09	-1.16	0.09
mbi_pa3.b5	-0.24	0.06	-0.24	0.06
mbi_pa4.a	1.44	0.11	1.44	0.11
mbi_pa4.b1	-2.99	0.23	-2.99	0.23
mbi_pa4.b2	-2.44	0.18	-2.44	0.18
mbi_pa4.b3	-1.64	0.13	-1.64	0.13
mbi_pa4.b4	-0.89	0.09	-0.89	0.09
mbi_pa4.b5	-0.35	0.08	-0.35	0.08
mbi_pa4.b6	1.31	0.11	1.31	0.11
mbi_pa5.a	1.89	0.16	1.89	0.16
mbi_pa5.b1	-1.77	0.13	-1.77	0.13
mbi_pa5.b2	-1.41	0.11	-1.41	0.11
mbi_pa5.b3	-0.30	0.07	-0.30	0.07
mbi_pa6.a	1.77	0.13	1.77	0.13
mbi_pa6.b1	-2.50	0.18	-2.50	0.18
mbi_pa6.b2	-1.82	0.13	-1.82	0.13
mbi_pa6.b3	-1.42	0.10	-1.42	0.10
mbi_pa6.b4	-0.67	0.08	-0.67	0.08
mbi_pa6.b5	-0.23	0.07	-0.23	0.07
mbi_pa6.b6	1.04	0.09	1.04	0.09
mbi_pa7.a	2.45	0.19	2.45	0.19
mbi_pa7.b1	-2.13	0.14	-2.13	0.14
mbi_pa7.b2	-1.60	0.11	-1.60	0.11
mbi_pa7.b3	-1.13	0.08	-1.13	0.08
mbi_pa7.b4	-0.68	0.07	-0.68	0.07
mbi_pa7.b5	0.30	0.07	0.30	0.07
mbi_pa8.a	1.41	0.16	1.43	0.18
mbi_pa8.b1	-1.99	0.20	-2.65	0.30
mbi_pa8.b2	-1.55	0.16	-2.05	0.23
mbi_pa8.b3	-0.18	0.09	-0.68	0.11
Latent Mean	0.00	NA	0.14	0.07
Latent Variance	1.00	NA	0.98	0.12

Figure 3.60 Differential item and test functioning by specialty (General Internal Medicine and Radiology) – PA subscale

a) Expected total score functions for reference (General Internal Medicine) and focal (Radiology) groups

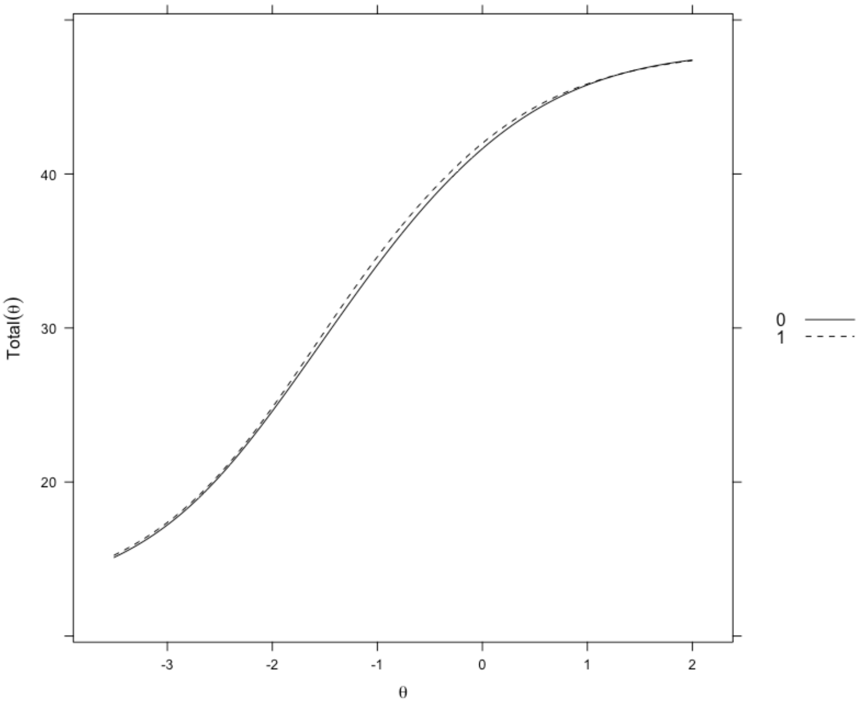

b) Expected item score functions for reference (General Internal Medicine) and focal (Radiology) groups

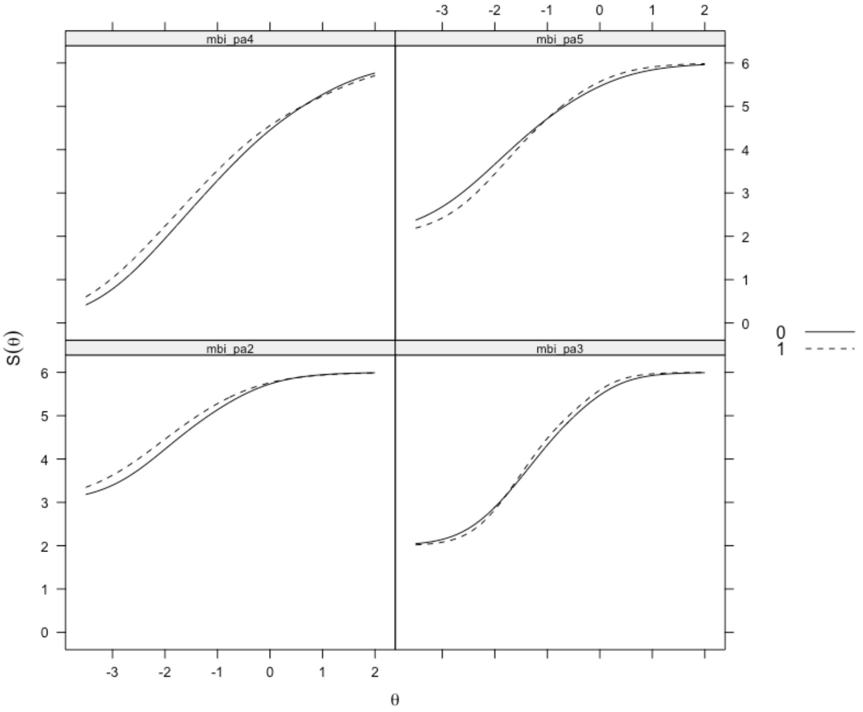

Table 3.60 Multi-group IRT item parameter estimates and standard errors (SE) by gender (reference: General Internal Medicine; focal: Radiology) – PA subscale

	**Reference group item parameter estimates**	**Reference group SE**	**Focal group item parameter estimates**	**Focal group SE**
mbi_pa1.a	0.96	0.12	0.96	0.12
mbi_pa1.b1	-3.05	0.34	-3.05	0.34
mbi_pa1.b2	-2.48	0.27	-2.48	0.27
mbi_pa1.b3	-0.87	0.12	-0.87	0.12
mbi_pa2.a	1.77	0.21	1.46	0.27
mbi_pa2.b1	-2.34	0.22	-2.51	0.34
mbi_pa2.b2	-1.97	0.18	-2.21	0.29
mbi_pa2.b3	-0.71	0.10	-1.06	0.15
mbi_pa3.a	2.38	0.25	2.86	0.43
mbi_pa3.b1	-2.12	0.17	-2.03	0.19
mbi_pa3.b2	-1.44	0.12	-1.56	0.15
mbi_pa3.b3	-1.05	0.10	-1.14	0.12
mbi_pa3.b4	-0.15	0.07	-0.20	0.10
mbi_pa4.a	1.60	0.15	1.51	0.22
mbi_pa4.b1	-2.74	0.26	-2.97	0.39

mbi_pa4.b2	-2.27	0.20	-2.53	0.31
mbi_pa4.b3	-1.67	0.15	-1.80	0.21
mbi_pa4.b4	-0.86	0.10	-1.08	0.14
mbi_pa4.b5	-0.29	0.09	-0.53	0.12
mbi_pa4.b6	1.12	0.12	1.30	0.23
mbi_pa5.a	1.51	0.17	1.78	0.28
mbi_pa5.b1	-2.63	0.26	-2.25	0.26
mbi_pa5.b2	-2.06	0.20	-2.00	0.22
mbi_pa5.b3	-1.59	0.16	-1.46	0.17
mbi_pa5.b4	-0.29	0.09	-0.43	0.11
mbi_pa6.a	1.95	0.15	1.95	0.15
mbi_pa6.b1	-2.49	0.18	-2.49	0.18
mbi_pa6.b2	-1.85	0.13	-1.85	0.13
mbi_pa6.b3	-1.42	0.11	-1.42	0.11
mbi_pa6.b4	-0.76	0.08	-0.76	0.08
mbi_pa6.b5	-0.29	0.07	-0.29	0.07
mbi_pa6.b6	0.95	0.10	0.95	0.10
mbi_pa7.a	2.54	0.22	2.54	0.22
mbi_pa7.b1	-2.29	0.16	-2.29	0.16
mbi_pa7.b2	-1.68	0.12	-1.68	0.12
mbi_pa7.b3	-1.17	0.09	-1.17	0.09
mbi_pa7.b4	-0.73	0.07	-0.73	0.07
mbi_pa7.b5	0.23	0.07	0.23	0.07
mbi_pa8.a	1.27	0.12	1.27	0.12
mbi_pa8.b1	-3.64	0.35	-3.64	0.35
mbi_pa8.b2	-2.98	0.27	-2.98	0.27
mbi_pa8.b3	-2.13	0.19	-2.13	0.19
mbi_pa8.b4	-1.51	0.14	-1.51	0.14
mbi_pa8.b5	-0.08	0.09	-0.08	0.09
Latent Mean	0.00	NA	-0.34	0.09
Latent Variance	1.00	NA	0.93	0.16

Table 4. Detailed LRT and Item-Level sDRF statistic DIF Detection Results: EE Subscale [a]

			**Likelihood ratio test for DIF detection using a forward approach [b]**				***Item-Level Signed DRF (sDRF) [c]***			
DIF Grouping Variable (Reference, n)	***Focal group (n)***	***Item***	***AIC difference***	***X^2 (df)***	***p-value***	***B-H adjusted p-value***	***sDRF statistic (95% CI)***	***X^2 (df)***	***p-value***	***B-H adjusted p-value***
**Sex** (male, n = 4078)	Female (n = 2005)	EE1	-12.98	24.98 (6)	**0.0003**	**0.0007**	-0.01 (-0.06, 0.04)	0.20 (1)	0.6573	0.7194
		EE2	-15.54	27.54 (6)	**0.0001**	**0.0003**	-0.01 (-0.04, 0.07)	0.13 (1)	0.7194	0.7194
		EE3	--	--	--	--	--	--	--	--
		EE5	--	--	--	--	--	--	--	--
		EE6	-21.38	33.38 (6)	**<0.0000**	**<0.0000**	0.13 (0.07, 0.19)	17.75 (1)	**<0.0000**	**0.0001**
		EE7	-37.03	49.03 (6)	**<0.0000**	**<0.0000**	0.20 (0.13, 0.27)	30.89 (1)	**<0.0000**	**<0.0000**
		EE4EE8	--	--	--	--	--	--	--	--
		EE9	--	--	--	--	--	--	--	--
**Age Category** (≥65 years, n = 1258)	<35 years (n = 313)	EE1	-13.04	25.04 (6)	**0.0003**	**0.0007**	0.04 (-0.09, 0.16)	0.30 (1)	0.5859	0.5859
		EE2	--	--	--	--	--	--	--	--
		EE3	-34.45	46.45 (6)	**<0.0000**	**<0.0000**	-0.31 (-0.44, -0.18)	21.95 (1)	**<0.0000**	**<0.0000**
		EE5	--	--	--	--	--	--	--	--
		EE6	-37.47	49.47 (6)	**<0.0000**	**<0.0000**	0.31 (0.18, 0.45)	19.63 (1)	**<0.0000**	**<0.0000**
		EE7	-31.08	43.08 (6)	**<0.0000**	**<0.0000**	0.18 (0.03, 0.34)	5.51 (1)	**0.0189**	**0.0252**
		EE4EE8	--	--	--	--	--	--	--	--
		EE9	--	--	--	--	--	--	--	--
	35-44 years (n = 1167)	EE1	-3.20	15.20 (6)	**0.0187**	**0.0375**	<0.01 (-0.08, 0.08)	0.01 (1)	0.9182	0.9182
		EE2	--	--	--	--	--	--	--	--
		EE3	-57.16	69.16 (6)	**<0.0000**	**<0.0000**	-0.33 (-0.42, -0.23)	46.33 (1)	**<0.0000**	**<0.0000**
		EE5	--	--	--	--	--	--	--	--
		EE6	-42.66	54.66 (6)	**<0.0000**	**<0.0000**	0.13 (0.03, 0.23)	6.95 (1)	**0.0084**	**0.0167**
		EE7	-22.97	34.97 (6)	**<0.0000**	**<0.0000**	0.03 (-0.08, 0.14)	0.37 (1)	0.5437	0.7250
		EE4EE8	--	--	--	--	--	--	--	--
		EE9	--	--	--	--	--	--	--	--
	45-54 years (n = 1328)	EE1	--	--	--	--	--	--	--	--
		EE2	--	--	--	--	--	--	--	--
		EE3	-6.69	18.69 (6)	**0.0047**	**0.0094**	-0.13 (-0.22, -0.04)	7.44 (1)	**0.0064**	**0.0085**
		EE5	--	--	--	--	--	--	--	--
		EE6	-9.30	21.30 (6)	**0.0016**	**0.0043**	0.11 (0.01, 0.20)	5.06 (1)	**0.0246**	**0.0246**
		EE7	-25.71	37.71 (6)	**<0.0000**	**<0.0000**	-0.16 (-0.27, -0.06)	8.92 (1)	**0.0028**	**0.0056**
		EE4EE8	--	--	--	--	--	--	--	--

Brady et al. (2021)

		EE9	-15.90	27.90 (6)	**0.0001**	**0.0004**	0.16 (0.06, 0.26)	9.06 (1)	**0.0026**	**0.0056**
	55-64 years (n = 2013)	EE1	--	--	--	--	--	--	--	--
		EE2	--	--	--	--	--	--	--	--
		EE3	--	--	--	--	--	--	--	--
		EE5	--	--	--	--	--	--	--	--
		EE6	--	--	--	--	--	--	--	--
		EE7	-16.47	28.47 (6)	**<0.0000**	**0.0006**	-0.18 (-0.28, 0.08)	11.60 (1)	**0.0007**	**0.0007**
		EE4EE8	--	--	--	--	--	--	--	--
		EE9	--	--	--	--	--	--	--	--
**Specialty**	Anesthesiology (n = 219)	EE1	5.27	6.73 (6)	0.3470	0.9252	0.07 (-0.06, 0.22)	1.08 (1)	0.2978	0.3225
		EE2	--	--	--	--	--	--	--	--
		EE3	--	--	--	--	--	--	--	--
		EE5	2.78	9.22 (6)	0.1617	0.6470	-0.09 (-0.25, 0.07)	1.19 (1)	0.2758	0.3225
		EE6	--	--	--	--	--	--	--	--
		EE7	-11.00	23.00 (6)	**0.0008**	**0.0064**	0.10 (-0.10, 0.29)	0.98 (1)	0.3225	0.3225
		EE4EE8	--	--	--	--	--	--	--	--
		EE9	6.74	5.26 (6)	0.5104	1.0000	-0.13 (-0.36, 0.10)	1.25 (1)	0.2640	0.3225
	Emergency medicine (n = 320)	EE1	--	--	--	--	--	--	--	--
		EE2	--	--	--	--	--	--	--	--
		EE3	--	--	--	--	--	--	--	--
		EE5	--	--	--	--	--	--	--	--
		EE6	--	--	--	--	--	--	--	--
		EE7	-18.96	30.96 (6)	**<0.0000**	**0.0002**	0.01 (-0.16, 0.18)	0.01 (1)	0.9179	0.9179
		EE4EE8	-15.07	27.07 (12)	**0.0001**	**0.0006**	-0.43 (-0.60, -0.24)	21.64 (1)	**<0.0000**	**<0.0000**
		EE9	--	--	--	--	--	--	--	--
	Family medicine (n = 494)	EE1	2.09	9.91 (6)	0.1285	0.5140	-0.10 (-0.22, 0.02)	2.96 (1)	0.0854	0.3414
		EE2	--	--	--	--	--	--	--	--
		EE3	--	--	--	--	--	--	--	--
		EE5	5.72	6.28 (6)	0.3929	1.0000	-0.02 (-0.15, 0.10)	0.12 (1)	0.7316	0.7316
		EE6	--	--	--	--	--	--	--	--
		EE7	-4.99	16.99 (6)	**0.0093**	0.0746	-0.08 (-0.23, 0.08)	0.91 (1)	0.3394	0.6485
		EE4EE8	--	--	--	--	--	--	--	--
		EE9	11.86	0.14 (6)	0.9999	1.0000	-0.06 (-0.23, 0.12)	0.48 (1)	0.4864	0.6485
	GIM (R) (n = 424); General Pediatrics (n = 338) (F)	EE1	8.46	3.54 (6)	0.7389	1.0000	-0.04 (-0.16, 0.10)	0.33 (1)	0.5682	0.5682
		EE2	--	--	--	--	--	--	--	--
		EE3	--	--	--	--	--	--	--	--
		EE5	--	--	--	--	--	--	--	--
		EE6	-3.26	15.26 (6)	**0.0183**	0.1465	0.33 (0.16, 0.50)	15.10 (1)	**0.0001**	**0.0004**
		EE7	1.59	10.41 (6)	0.1086	0.4343	0.26 (0.09, 0.45)	8.00 (1)	**0.0047**	**0.0094**

		EE4EE8	--	--	--	--	--	--	--	--
		EE9	5.99	6.01 (6)	0.4216	1.0000	0.07 (-0.11, 0.25)	0.63 (1)	0.4273	0.5682
	GIM (R) (n = 424); General Surgery (F) (n = 230)	EE1	--	--	--	--	--	--	--	--
		EE2	-3.91	15.91 (6)	**0.0142**	0.0570	0.15 (0.01, 0.30)	4.30 (1)	**0.0382**	**0.0382**
		EE3	--	--	--	--	--	--	--	--
		EE5	--	--	--	--	--	--	--	--
		EE6	--	--	--	--	--	--	--	--
		EE7	--	--	--	--	--	--	--	--
		EE4EE8	--	--	--	--	--	--	--	--
		EE9	-5.35	17.35 (6)	**0.0081**	0.0570	-0.25 (-0.43,-0.05)	6.48 (1)	**0.0109**	**0.0218**
	GIM (R) (n = 424); General surgery subspecialty (F) (n = 350)	EE1	8.10	3.90 (6)	0.6908	1.0000	0.11 (-0.03, 0.25)	2.37 (1)	0.1235	0.3570
		EE2	-0.87	12.87 (6)	**0.0452**	0.3616	0.10 (-0.04, 0.25)	1.81 (1)	0.1785	0.3570
		EE3	--	--	--	--	--	--	--	--
		EE5	8.97	3.03 (6)	0.8049	1.0000	-0.05 (-0.20, 0.10)	0.51 (1)	0.4739	0.6319
		EE6	--	--	--	--	--	--	--	--
		EE7	8.68	3.32 (6)	0.7677	1.0000	0.01 (-0.19, 0.21)	0.02 (1)	0.8998	0.8998
		EE4EE8	--	--	--	--	--	--	--	--
		EE9	--	--	--	--	--	--	--	--
	GIM (R) (n = 424); Internal medicine subspecialty (F) (n = 711)	EE1	--	--	--	--	--	--	--	--
		EE2	--	--	--	--	--	--	--	--
		EE3	10.02	1.98 (6)	0.9217	1.0000	0.02 (-0.12, 0.17)	0.06 (1)	0.8116	0.9991
		EE5	10.19	1.81 (6)	0.9363	1.0000	0.00 (-0.13, 0.13)	0.13 (1)	0.9991	0.9991
		EE6	6.44	5.56 (6)	0.4740	1.0000	0.16 (0.00, 0.31)	4.16 (1)	**0.0414**	0.1657
		EE7	--	--	--	--	--	--	--	--
		EE4EE8	--	--	--	--	--	--	--	--
		EE9	6.64	5.36 (6)	0.4991	1.0000	0.04 (-0.12, 0.21)	0.19 (1)	0.6620	0.9991
	Neurology(F) (n = 221)	EE1	--	--	--	--	--	--	--	--
		EE2	--	--	--	--	--	--	--	--
		EE3	6.19	5.81 (6)	0.4453	1.0000	-0.07 (-0.25, 0.11)	0.56 (1)	0.4557	0.4557
		EE5	6.26	5.74 (6)	0.4531	1.0000	-0.13 (-0.30, 0.02)	2.89 (1)	0.0892	0.3183
		EE6	--	--	--	--	--	--	--	--
		EE7	3.34	8.66 (6)	0.1934	1.0000	-0.13 (0.33, 0.07)	1.65 (1)	0.1995	0.3183
		EE4EE8	--	--	--	--	--	--	--	--
		EE9	7.75	4.25 (6)	0.6425	1.0000	-0.13 (-0.33, 0.08)	1.39 (1)	0.2387	0.3183
	Obstetrics and gynecology (F) (n = 267)	EE1	7.99	4.01 (6)	0.6755	1.0000	-0.06 (-0.20, 0.08)	0.80 (1)	0.3716	0.6331
		EE2	6.24	5.76 (6)	0.4509	1.0000	-0.01 (-0.15, 0.15)	0.01 (1)	0.9328	0.9328
		EE3	--	--	--	--	--	--	--	--
		EE5	--	--	--	--	--	--	--	--
		EE6	--	--	--	--	--	--	--	--
		EE7	-5.81	17.81 (6)	**0.0067**	0.0537	0.08 (-0.14, 0.27)	0.54 (1)	0.4616	0.6331

Brady et al. (2021)

		EE4EE8	--	--	--	--	--	--	--	--
		EE9	4.35	7.65 (6)	0.2651	1.0000	-0.07 (-0.28, 0.11)	0.51 (1)	0.4748	0.6331
	Ophthalmology (F) (n = 219)	EE1	11.73	0.27 (6)	0.9996	1.0000	-0.02 (-0.17, 0.14)	0.07 (1)	0.7975	0.7875
		EE2	5.86	6.14 (6)	0.4072	1.0000	0.11 (-0.06, 0.28)	1.59 (1)	0.2067	0.4134
		EE3	6.60	5.40 (6)	0.4931	1.0000	0.13 (-0.06, 0.32)	1.80 (1)	0.1801	0.4134
		EE5	--	--	--	--	--	--	--	--
		EE6	--	--	--	--	--	--	--	--
		EE7	--	--	--	--	--	--	--	--
		EE4EE8	--	--	--	--	--	--	--	--
		EE9	4.51	7.49 (6)	0.2781	1.0000	-0.04 (-0.25, 0.16)	0.16 (1)	0.6877	0.7975
	Orthopedic surgery) (n = 219)	EE1	--	--	--	--	--	--	--	--
		EE2	9.66	2.34 (6)	0.8861	1.0000	0.00 (-0.15, 0.16)	<0.01 (1)	0.9524	0.9937
		EE3	--	--	--	--	--	--	--	--
		EE5	--	--	--	--	--	--	--	--
		EE6	8.37	3.63 (6)	0.7263	1.0000	-0.04 (-0.23, 0.16)	0.19 (1)	0.6595	0.9937
		EE7	10.01	1.99 (6)	0.9206	1.0000	-0.16 (-0.37, 0.06)	2.16 (1)	0.1417	0.5667
		EE4EE8	--	--	--	--	--	--	--	--
		EE9	9.54	2.46 (6)	0.8729	1.0000	0.00 (-0.22, 0.22)	<0.01 (1)	0.9937	0.9937
	Pediatric subspecialty (F) (n = 293)	EE1	--	--	--	--	--	--	--	--
		EE2	--	--	--	--	--	--	--	--
		EE3	--	--	--	--	--	--	--	--
		EE5	--	--	--	--	--	--	--	--
		EE6	-14.10	26.10 (6)	**0.0002**	**0.0017**	0.21 (0.05, 0.38)	6.32 (1)	**0.0120**	**0.0120**
		EE7	--	--	--	--	--	--	--	--
		EE4EE8	--	--	--	--	--	--	--	--
		EE9	--	--	--	--	--	--	--	--
	GIM (R) (n =424); Physical medicine and rehabilitation/preventive medicine/occupational medicine (F) (n = 267)	EE1	--	--	--	--	--	--	--	--
		EE2	--	--	--	--	--	--	--	--
		EE3	--	--	--	--	--	--	--	--
		EE5	--	--	--	--	--	--	--	--
		EE6	-16.46	28.46 (6)	**0.0001**	**0.0006**	0.15 (-0.01, 0.30)	3.21 (1)	0.0733	0.0733
		EE7	--	--	--	--	--	--	--	--
		EE4EE8	--	--	--	--	--	--	--	--
		EE9	--	--	--	--	--	--	--	--
	GIM (n = 424) (R); Psychiatry (n = 525) (F)	EE1	--	--	--	--	--	--	--	--
		EE2	--	--	--	--	--	--	--	--
		EE3	--	--	--	--	--	--	--	--
		EE5	--	--	--	--	--	--	--	--
		EE6	-15.82	27.82 (6)	**0.0001**	**0.0008**	0.12 (-0.03, 0.27)	2.72 (1)	0.0990	0.0990
		EE7	--	--	--	--	--	--	--	--

Brady et al. (2021)

		EE4EE8	--	--	--	--	--	--	--	--
		EE9	--	--	--	--	--	--	--	--
	GIM (R) (n =424) ; Radiology (F) (n = 230)	EE1	8.28	3.72 (6)	0.7148	1.0000	0.07 (-0.07, 0.22)	0.86 (1)	0.3542	0.4427
		EE2	2.59	9.41 (6)	0.1520	0.6181	0.06 (-0.09, 0.21)	0.61 (1)	0.4336	0.4427
		EE3	9.37	2.63 (6)	0.8535	1.0000	0.07 (-0.12, 0.26)	0.59 (1)	0.4427	0.4427
		EE5	--	--	--	--	--	--	--	--
		EE6	--	--	--	--	--	--	--	--
		EE7	-9.29	21.29 (6)	**0.0016**	**0.0130**	0.08 (-0.12, 0.30)	0.65 (1)	0.4215	0.4427
		EE4EE8	--	--	--	--	--	--	--	--
		EE9	--	--	--	--	--	--	--	--

[a] *"--" indicates that the corresponding item was used as an anchor item. Bolded p-values are significant at p < 0.05.* [b] *If no DIF was detected in the LRT using an iterative, backward all-other anchor item selection approach, item EE4EE8 was selected as an initial anchor to identify items with the lowest AIC difference in an initial LRT using a forward anchor item selection approach. Then items from the initial LRT using the forward approach with the lowest AIC difference were added as anchors and DIF was retested in studied items. Item EE4EE8 was selected as an initial anchor as it showed minimal DIF throughout DIF testing.*
[c] *theta integration range for the sDRF statistic was -3.00 to 3.00.*

Table 5. Detailed LRT and Item-Level sDRF statistic DIF Detection Results: DP Subscale [a]

			**Likelihood ratio test for DIF detection using a forward approach** [b]				***Item-Level Signed DRF (sDRF)*** [c]			
DIF Grouping Variable (Reference group, n)	***Focal group (n)***	***Item***	***AIC difference***	***X² (df)***	***p-value***	***B-H adjusted p-value***	***sDRF statistic (95% CI)***	***X² (df)***	***p-value***	***B-H adjusted p-value***
**Sex** Male (n = 4178)	Female (n = 2032)	DP1	-6.13	18.13 (6)	**0.0059**	**0.0296**	0.15 (0.08,0.22)	18.16 (1)	**<0.0000**	**<0.0000**
		DP2	-1.85	13.85 (6)	**0.0314**	0.0784	-0.05 (-0.13, 0.01)	2.62 (1)	0.1105	0.1105
		DP3	--	--	--	--	--	--	--	--
		DP4	--	--	--	--	--	--	--	--
		DP5	--	--	--	--	--	--	--	--
**Age Category** (≥65 years, n = 1303)	<35 years (n = 309)	DP1	--	--	--	--	--	--	--	--
		DP2	--	--	--	--	--	--	--	--
		DP3	-12.02	24.02 (6)	**0.0005**	**0.0026**	0.02 (-0.14, 0.16)	0.08 (1)	0.7786	0.7786
		DP4	-8.11	20.11 (6)	**0.0026**	**0.0066**	0.04 (-0.11, 0.14)	0.57 (1)	0.5729	0.7786
		DP5	--	--	--	--	--	--	--	--
	35-44 years (n = 1170)	DP1	--	--	--	--	--	--	--	--
		DP2	-10.55	22.55 (6)	**0.0010**	**0.0024**	0.15 (0.00, 0.27)	4.61 (1)	**0.0318**	0.0636

Brady et al. (2021)

		DP3	-12.99	24.99 (6)	**0.0003**	**0.0017**	0.03 (-0.12, 0.17)	0.15 (1)	0.7006	0.7006
		DP4	--	--	--	--	--	--	--	--
		DP5	--	--	--	--	--	--	--	--
	45-54 years (n = 1345)	DP1	--	--	--	--	--	--	--	--
		DP2	--	--	--	--	--	--	--	--
		DP3	-13.76	25.76 (6)	**0.0002**	**0.0006**	-0.20 (-0.31, -0.08)	11.76 (1)	**0.0006**	**0.0012**
		DP4	-15.68	27.68 (6)	**0.0001**	**0.0005**	0.08 (-0.01, 0.18)	2.65 (1)	0.1034	0.1034
		DP5	--	--	--	--	--	--	--	--
	55-64 years (n = 2083)	DP1	--	--	--	--	--	--	--	--
		DP2	--	--	--	--	--	--	--	--
		DP3	-9.61	21.61 (6)	**0.0014**	**0.0071**	-0.14 (-0.24, 0.03)	6.63 (1)	**0.0100**	**0.0201**
		DP4	-3.44	15.44 (6)	**0.0171**	**0.0427**	0.04 (-0.04, 0.12)	0.71 (1)	0.3982	0.3982
		DP5	--	--	--	--	--	--	--	--
**Specialty** (GIM, n = 438)	Anesthesiology (n = 224)	DP1	-6.11	18.11 (6)	**0.0060**	**0.0299**	-0.31 (-0.52, -0.09)	8.16 (1)	**0.0043**	**0.0043**
		DP2	--	--	--	--	--	--	--	--
		DP3	--	--	--	--	--	--	--	--
		DP4	-3.44	15.44 (6)	**0.0171**	**0.0428**	0.40 (0.19, 0.56)	17.52 (1)	**<0.0000**	**<0.0001**
		DP5	--	--	--	--	--	--	--	--
	Emergency medicine (n = 331)	DP1	4.31	7.69 (6)	0.2617	0.6542	-0.27 (-0.48, -0.07)	6.47 (1)	**0.0110**	**0.0110**
		DP2	--	--	--	--	--	--	--	--
		DP3	-20.60	32.60 (6)	**<0.0000**	**<0.0000**	0.38 (0.17, 0.59)	13.04 (1)	**0.0003**	**0.0006**
		DP4	--	--	--	--	--	--	--	--
		DP5	--	--	--	--	--	--	--	--
	Family Medicine (n = 508)	DP1	--	--	--	--	--	--	--	--
		DP2	--	--	--	--	--	--	--	--
		DP3	-13.19	25.19 (6)	**0.0003**	**0.0016**	0.40 (0.21, 0.58)	18.76 (1)	**<0.0000**	**<0.0000**
		DP4	--	--	--	--	--	--	--	--
		DP5	--	--	--	--	--	--	--	--
	General pediatrics (n = 346)	DP1	4.88	7.12 (6)	0.3095	0.7739	-0.08 (-0.30, 0.10)	0.62 (1)	0.4323	0.4323
		DP2	-1.32	13.32 (6)	**0.0383**	0.1915	-0.27 (-0.46, -0.06)	7.42 (1)	**0.0064**	**0.0129**
		DP3	--	--	--	--	--	--	--	--
		DP4	--	--	--	--	--	--	--	--
		DP5	--	--	--	--	--	--	--	--
	General surgery (n = 239)	DP1	6.24	5.76 (6)	0.4503	1.0000	-0.12 (-0.34, 0.08)	1.38 (1)	0.2409	0.2409
		DP2	4.19	7.81 (6)	0.2521	1.0000	-0.16 (-0.38, 0.05)	2.14 (1)	0.1437	0.2409
		DP3	--	--	--	--	--	--	--	--
		DP4	--	--	--	--	--	--	--	--
		DP5	--	--	--	--	--	--	--	--
		DP1	--	--	--	--	--	--	--	--

	General surgery subspecialty (n = 355)	DP2	--	--	--	--	--	--	--	--
		DP3	--	--	--	--	--	--	--	--
		DP4	3.09	8.91 (6)	0.1786	0.4464	0.35 (0.20, 0.49)	18.97 (1)	**<0.0000**	**<0.0000**
		DP5	-3.79	15.79 (6)	**0.0149**	0.0746	-0.28 (-0.52, -0.05)	5.52 (1)	**0.0189**	**0.0189**
	Internal medicine subspecialty (n = 730)	DP1	--	--	--	--	--	--	--	--
		DP2	6.41	5.59 (6)	0.4710	1.0000	-0.02 (-0.17, 0.13)	0.09 (1)	0.7601	0.8712
		DP3	--	--	--	--	--	--	--	--
		DP4	2.46	7.54 (5)	0.1838	0.9188	0.01 (-0.13, 0.14)	0.03 (1)	0.8712	0.8712
		DP5	--	--	--	--	--	--	--	--
	Neurology (n = 234)	DP1	--	--	--	--	--	--	--	--
		DP2	--	--	--	--	--	--	--	--
		DP3	-10.61	22.61 (6)	**0.0009**	**0.0047**	0.41 (0.19, 0.63)	13.72 (1)	**0.0002**	**0.0002**
		DP4	--	--	--	--	--	--	--	--
		DP5	--	--	--	--	--	--	--	--
	Obstetrics and gynecology (n = 270)	DP1	5.90	4.10 (5)	0.5350	1.0000	-0.09 (-0.30, 0.10)	0.85 (1)	0.3552	0.3552
		DP2	4.84	7.16 (6)	0.3063	1.0000	-0.29 (-0.49, -0.08)	8.06 (1)	**0.0045**	**0.0091**
		DP3	--	--	--	--	--	--	--	--
		DP4	--	--	--	--	--	--	--	--
		DP5	--	--	--	--	--	--	--	--
	Ophthalmology (n = 226)	DP1	--	--	--	--	--	--	--	--
		DP2	5.07	6.93 (6)	0.3275	1.0000	-0.16 (-0.38, 0.07)	1.89 (1)	0.1689	0.2842
		DP3	--	--	--	--	--	--	--	--
		DP4	7.05	4.95 (6)	0.5498	1.0000	0.12 (-0.11, 0.33)	1.15 (1)	0.2842	0.2842
		DP5	--	--	--	--	--	--	--	--
	Orthopedic surgery (n = 224)	DP1	--	--	--	--	--	--	--	--
		DP2	11.65	0.35 (6)	0.9992	1.0000	0.07 (0.04, 0.35)	0.43 (2)	0.8065	0.8065
		DP3	--	--	--	--	--	--	--	--
		DP4	2.41	7.59 (5)	0.1801	0.9006	0.23 (0.08, 0.42)	5.03 (1)	**0.0249**	**0.0498**
		DP5	--	--	--	--	--	--	--	--
	Pediatric subspecialty (n = 301)	DP1	--	--	--	--	--	--	--	--
		DP2	1.79	10.21 (6)	0.1161	0.5804	-0.16 (-0.36, 0.06)	2.08 (1)	0.1489	0.1489
		DP3	--	--	--	--	--	--	--	--
		DP4	5.47	4.53 (5)	0.4759	1.0000	0.15 (-0.03, 0.31)	2.91 (1)	0.0881	0.1489
		DP5	--	--	--	--	--	--	--	--
	Physical medicine and rehabilitation/ preventive medicine/	DP1	--	--	--	--	--	--	--	--
		DP2	7.93	4.07 (6)	0.6674	1.0000	-0.03 (-0.25, 0.21)	0.06 (1)	0.8140	0.8140
		DP3	1.38	10.62 (6)	0.1008	0.5041	0.30 (0.03, 0.57)	4.61 (1)	**0.0317**	0.0634
		DP4	--	--	--	--	--	--	--	--
		DP5	--	--	--	--	--	--	--	--

Brady et al. (2021)

	occupational medicine (n = 263)									
	Psychiatry (n = 531)	DP1	--	--	--	--	--	--	--	--
		DP2	--	--	--	--	--	--	--	--
		DP3	-17.89	29.89 (6)	**<0.0000**	**0.0002**	0.42 (0.24, 0.61)	19.50 (1)	**<0.0000**	**<0.0000**
		DP4	--	--	--	--	--	--	--	--
		DP5	-0.27	12.27 (6)	0.0562	0.1405	-0.33 (-0.54, -0.12)	9.04 (1)	**<0.0026**	**<0.0026**
	Radiology (n = 240)	DP1	-5.67	15.67 (5)	**0.0079**	**0.0196**	-0.31 (-0.52, -0.07)	7.90 (1)	**0.0049**	**0.0049**
		DP2	--	--	--	--	--	--	--	--
		DP3	--	--	--	--	--	--	--	--
		DP4	--	--	--	--	--	--	--	--
		DP5	-10.07	22.07 (6)	**0.0012**	**0.0059**	0.53 (0.29, 0.78)	17.81 (1)	**<0.0000**	**<0.0000**

[a] "--" indicates that the corresponding item was used as a final anchor item. Reference and focal group are denoted by "R" and "F", respectively. Bolded p-values are significant at $p < 0.05$. [b] If no DIF was detected in the LRT using an iterative, backward all-other anchor item selection approach, item DP5 was selected as an initial anchor to identify items with the lowest AIC difference in an initial LRT using a forward anchor item selection approach. Then items from the initial LRT using the forward approach with the lowest AIC difference were added as anchors and DIF was retested in studied items. Item DP5 was selected as an initial anchor as it showed minimal DIF throughout DIF testing. Items with sparse item response categories (≤5 respondents) were collapsed prior to multi-group IRT model estimation. [c] theta integration range for the sDRF statistic was -3.05 to 3.00.

Table 6. Detailed LRT and Item-Level sDRF statistic DIF Detection Results: DP Subscale [a]

			Likelihood ratio test for DIF detection using a forward approach [b]				*Item-Level Signed DRF (sDRF)* [c]			
DIF Grouping Variable (Reference group, n)	***Focal group (n)***	***Item***	***AIC difference***	***X^2 (df)***	***p-value***	***B-H adjusted p-value***	***sDRF statistic (95% CI)***	***X^2 (df)***	***p-value***	***B-H adjusted p-value***
**Sex:** (Male, n = 4048)	Female (n = 1973)	PA1	-36.16	48.16 (6)	**<0.0000**	**<0.0000**	-0.21 (-0.26, -0.16)	67.84 (1)	**<0.0000**	**<0.0000**
		PA2	--	--	--	--	--	--	--	--
		PA3	--	--	--	--	--	--	--	--
		PA4	-12.96	24.96 (6)	**0.0003**	**0.0014**	0.13 (0.06, 0.20)	12.54 (1)	**0.0004**	**0.0008**
		PA5	--	--	--	--	--	--	--	--
		PA6	-0.15	12.15 (6)	0.0587	0.1175	-0.10 (-0.17, -0.02)	6.96 (1)	**0.0083**	**0.0111**
		PA7	-1.15	13.15 (6)	**0.0408**	0.1088	0.05 (-0.01, 0.11)	3.34 (1)	0.0676	0.0676
		PA8	--	--	--	--	--	--	--	--
	<35 years (n = 307)	PA1	-19.03	27.03 (4)	**<0.0000**	**<0.0000**	-0.14 (-0.24, -0.02)	7.44 (1)	**0.0064**	**0.0106**

Brady et al. (2021)

**Age Category** (≥65 years, n = 1222)		PA2	-9.91	17.91 (4)	**0.0013**	**0.0026**	-0.11 (-0.18, -0.03)	7.50 (1)	**0.0062**	**0.0106**
		PA3	-14.38	24.38 (5)	**0.0002**	**0.0005**	0.05 (-0.05, 0.16)	0.95 (1)	0.3285	0.3285
		PA4	-24.76	34.76 (5)	**<0.0000**	**<0.0000**	0.16 (0.02, 0.31)	4.54 (1)	**0.0331**	**0.0414**
		PA5	--	--	--	--	--	--	--	--
		PA6	--	--	--	--	--	--	--	--
		PA7	-1.74	11.74 (5)	**0.0386**	0.0618	0.25 (0.14, 0.38)	17.16 (1)	**<0.0000**	**0.0002**
		PA8	--	--	--	--	--	--	--	--
	35-44 years (n = 1158)	PA1	-42.62	54.62 (6)	**<0.0000**	**<0.0000**	-0.21 (-0.30, -0.12)	19.69 (1)	**<0.0000**	**<0.0000**
		PA2	-11.27	21.27 (5)	**0.0007**	**0.0014**	-0.20 (-0.29, -0.13)	24.41 (1)	**<0.0000**	**<0.0000**
		PA3	--	--	--	--	--	--	--	--
		PA4	-26.04	38.04 (6)	**<0.0000**	**<0.0000**	0.20 (0.08, 0.31)	11.78 (1)	**0.0006**	**0.0007**
		PA5	--	--	--	--	--	--	--	--
		PA6	-17.66	29.66 (6)	**<0.0000**	**0.0001**	0.05 (-0.07, 0.16)	0.66 (1)	0.4162	0.4162
		PA7	-1.91	13.91 (6)	**0.0306**	**0.0490**	0.23 (0.14, 0.32)	26.10 (1)	**<0.0000**	**<0.0000**
		PA8	--	--	--	--	--	--	--	--
	45-54 years (n = 1318)	PA1	-43.92	53.92 (6)	**<0.0000**	**<0.0000**	-0.27 (-0.35, -0.18)	36.89 (1)	**<0.0000**	**<0.0000**
		PA2	-8.11	20.11 (6)	**0.0026**	**0.0070**	-0.26 (-0.34, -0.18)	38.89 (1)	**<0.0000**	**<0.0000**
		PA3	--	--	--	--	--	--	--	--
		PA4	-7.44	19.44 (6)	**0.0035**	**0.0070**	0.15 (0.03, 0.26)	7.75 (1)	**0.0054**	**0.0072**
		PA5	-16.26	28.26 (6)	**<0.0000**	**0.0003**	-0.12 (-0.21, -0.02)	6.07 (1)	**0.0137**	**0.0137**
		PA6	--	--	--	--	--	--	--	--
		PA7	--	--	--	--	--	--	--	--
		PA8	--	--	--	--	--	--	--	--
	55-64 years (n = 2013)	PA1	-20.64	32.64 (6)	**<0.0000**	**<0.0000**	-0.22 (-0.31, -0.13)	24.70 (1)	**<0.0000**	**<0.0000**
		PA2	1.22	10.78 (6)	0.0954	0.1908	-0.17 (-0.25, -0.10)	19.96 (1)	**<0.0000**	**<0.0000**
		PA3	--	--	--	--	--	--	--	--
		PA4	-39.28	51.28 (6)	**<0.0000**	**<0.0000**	0.29 (0.20, 0.39)	36.62 (1)	**<0.0000**	**<0.0000**
		PA5	--	--	--	--	--	--	--	--
		PA6	-12.64	24.64 (6)	**0.0004**	**0.0011**	0.19 (0.09, 0.28)	15.49 (1)	**0.0001**	**0.0001**
		PA7	--	--	--	--	--	--	--	--
		PA8	--	--	--	--	--	--	--	--
**Specialty** GIM (n = 427)	Anesthesiology (n =215)	PA1	--	--	--	--	--	--	--	--
		PA2	--	--	--	--	--	--	--	--
		PA3	--	--	--	--	--	--	--	--
		PA4	8.21	3.79 (6)	0.7055	1.0000	-0.18 (-0.40, 0.04)	2.66 (1)	0.1030	0.2060
		PA5	-0.33	8.33 (4)	0.0803	0.6428	-0.18 (-0.32, -0.02)	5.53 (1)	**0.0186**	0.0746
		PA6	6.40	5.60 (6)	0.4691	1.0000	-0.14 (-0.38, 0.08)	1.51 (1)	0.2196	0.2928
		PA7	8.07	1.93 (5)	0.8592	1.0000	-0.10 (-0.26, 0.09)	1.06 (1)	0.3026	0.3026
		PA8	--	--	--	--	--	--	--	--
		PA1	--	--	--	--	--	--	--	--

	Emergency medicine (n=334)	PA2	--	--	--	--	--	--	--	--
		PA3	--	--	--	--	--	--	--	--
		PA4	-8.09	18.09 (5)	**0.0028**	**0.0226**	-0.30 (-0.46, -0.14)	13.51 (1)	**0.0002**	**0.0002**
		PA5	--	--	--	--	--	--	--	--
		PA6	--	--	--	--	--	--	--	--
		PA7	--	--	--	--	--	--	--	--
		PA8	--	--	--	--	--	--	--	--
	Family Medicine (F) (n = 497)	PA1	4.18	3.82 (4)	0.4314	1.0000	-0.03 (-0.11, 0.11)	0.31 (1)	0.5792	0.6302
		PA2	--	--	--	--	--	--	--	--
		PA3	--	--	--	--	--	--	--	--
		PA4	9.22	2.78 (6)	0.8354	1.0000	0.06 (-0.07, 0.30)	0.37 (1)	0.5454	0.6302
		PA5	--	--	--	--	--	--	--	--
		PA6	5.48	6.52 (6)	0.3675	1.0000	0.05 (-0.09, 0.18)	0.25 (1)	0.6153	0.6302
		PA7	6.11	3.89 (5)	0.5657	1.0000	0.03 (-0.09, 0.18)	0.23 (1)	0.6302	0.6302
		PA8	--	--	--	--	--	--	--	--
	General pediatrics (n = 337) (F)	PA1	--	--	--	--	--	--	--	--
		PA2	--	--	--	--	--	--	--	--
		PA3	--	--	--	--	--	--	--	--
		PA4	7.27	4.73 (6)	0.5784	1.0000	0.03 (-0.17, 0.22)	0.07 (1)	0.7940	0.7940
		PA5	3.16	4.84 (4)	0.3036	1.0000	-0.04 (-0.16, 0.10)	0.26 (1)	0.6126	0.7940
		PA6	-3.93	15.93 (6)	**0.0141**	0.1129	-0.14 (-0.33, 0.06)	2.00 (1)	0.1578	0.4921
		PA7	7.27	2.73 (5)	0.7422	1.0000	0.10 (-0.07, 0.25)	1.35 (1)	0.2461	0.4921
		PA8	--	--	--	--	--	--	--	--
	General surgery (n = 240)	PA1	6.78	1.22 (4)	0.8745	1.0000	0.15 (-0.01, 0.29)	3.60 (1)	0.0577	0.1153
		PA2	--	--	--	--	--	--	--	--
		PA3	--	--	--	--	--	--	--	--
		PA4	7.04	0.96 (4)	0.9156	1.0000	0.02 (-0.17, 0.19)	0.04 (1)	0.8349	0.8349
		PA5	-0.69	8.69 (4)	0.0694	0.5550	-0.13 (-0.26, 0.01)	3.79 (1)	0.0515	0.1153
		PA6	9.31	2.69 (6)	0.8470	1.0000	-0.09 (-0.29, 0.10)	0.70 (1)	0.4018	0.5357
		PA7	--	--	--	--	--	--	--	--
		PA8	--	--	--	--	--	--	--	--
	General surgery subspecialty (n = 339)	PA1	-4.51	10.51 (6)	**0.0147**	0.0588	0.23 (0.10, 0.36)	11.86 (1)	**0.0006**	**0.0006**
		PA2	--	--	--	--	--	--	--	--
		PA3	--	--	--	--	--	--	--	--
		PA4	--	--	--	--	--	--	--	--
		PA5	--	--	--	--	--	--	--	--
		PA6	--	--	--	--	--	--	--	--
		PA7	--	--	--	--	--	--	--	--
		PA8	-11.02	21.02 (6)	**0.0008**	**0.0064**	0.30 (0.14, 0.46)	12.69 (1)	**0.0004**	**0.0006**
		PA1	5.67	2.33 (4)	0.6755	1.0000	0.13 (0.01, 0.23)	5.67 (1)	**0.0173**	0.0691

Brady et al. (2021)

	Internal medicine subspecialty (n = 712)	PA2	7.54	0.46 (4)	0.9777	1.0000	0.02 (-0.07, 0.10)	0.20 (1)	0.6572	0.6792
		PA3	--	--	--	--	--	--	--	--
		PA4	5.68	6.32 (6)	0.3885	1.0000	0.04 (-0.11, 0.19)	0.26 (1)	0.6070	0.6792
		PA5	--	--	--	--	--	--	--	--
		PA6	8.94	3.06 (6)	0.8015	1.0000	0.03 (-0.13, 0.19)	0.17 (1)	0.6792	0.6792
		PA7	--	--	--	--	--	--	--	--
		PA8	--	--	--	--	--	--	--	--
	Neurology (n = 226)	PA1	--	--	--	--	--	--	--	--
		PA2	--	--	--	--	--	--	--	--
		PA3	5.05	2.95 (4)	0.5667	1.00	-0.19 (-0.33, -0.05)	6.80 (1)	**0.0091**	**0.0366**
		PA4	8.94	3.06 (6)	0.8018	1.00	-0.10 (-0.32, 0.14)	0.75 (1)	0.3870	0.3870
		PA5	3.85	4.15 (4)	0.3859	1.00	-0.10 (-0.24, 0.04)	1.83 (1)	0.1764	0.2352
		PA6	6.37	5.63 (6)	0.4662	1.00	-0.17 (-0.39, 0.04)	2.53 (1)	0.1118	0.2236
		PA7	--	--	--	--	--	--	--	--
		PA8	--	--	--	--	--	--	--	--
	Obstetrics and gynecology (n = 270)	PA1	--	--	--	--	--	--	--	--
		PA2	3.63	2.37 (3)	0.5001	1.0000	0.02 (-0.08, 0.13)	0.18 (1)	0.6732	0.6732
		PA3	3.20	2.80 (3)	0.4239	1.0000	-0.05 (-0.17, 0.08)	0.55 (1)	0.4598	0.6131
		PA4	3.37	6.63 (5)	0.2498	0.9991	0.18 (0.01, 0.37)	3.55 (1)	0.0595	0.2380
		PA5	1.45	6.55 (4)	0.1617	0.9991	-0.07 (-0.21, 0.08)	0.84 (1)	0.3596	0.6131
		PA6	--	--	--	--	--	--	--	--
		PA7	--	--	--	--	--	--	--	--
		PA8	--	--	--	--	--	--	--	--
	Ophthalmology (n = 218)	PA1	--	--	--	--	--	--	--	--
		PA2	--	--	--	--	--	--	--	--
		PA3	--	--	--	--	--	--	--	--
		PA4	--	--	--	--	--	--	--	--
		PA5	--	--	--	--	--	--	--	--
		PA6	--	--	--	--	--	--	--	--
		PA7	-9.78	17.78 (4)	**0.0014**	**0.0109**	-0.33 (-0.47, -0.17)	20.02 (1)	**<0.0000**	**<0.0000**
		PA8	-7.18	15.18 (4)	**0.0043**	**0.0174**	0.25 (0.09, 0.41)	9.39 (1)	**0.0022**	**0.0022**
	Orthopedic Surgery (n = 217)	PA1	6.25	1.75 (4)	0.7821	1.0000	0.17 (0.02, 0.34)	4.41 (1)	**0.0357**	0.1427
		PA2	--	--	--	--	--	--	--	--
		PA3	0.66	5.34 (3)	0.1487	0.5949	-0.08 (-0.21, 0.06)	1.45 (1)	0.2289	0.4578
		PA4	8.91	3.09 (6)	0.7969	1.0000	0.07 (-0.17, 0.30)	0.34 (1)	0.5577	0.5577
		PA5	--	--	--	--	--	--	--	--
		PA6	--	--	--	--	--	--	--	--
		PA7	-3.22	11.22 (4)	**0.0242**	0.1935	-0.07 (-0.23, 0.08)	0.86 (1)	0.3551	0.4734
		PA8	--	--	--	--	--	--	--	--
		PA1	2.03	3.97 (3)	0.2643	1.0000	0.06 (-0.06, 0.19)	0.97 (1)	0.3251	0.6501

Brady et al. (2021)

Pediatric subspecialty (n = 291)	PA2	--	--	--	--	--	--	--	--
	PA3	--	--	--	--	--	--	--	--
	PA4	7.91	4.09 (6)	0.6650	1.0000	0.01 (-0.18, 0.21)	0.01 (1)	0.9117	0.9302
	PA5	7.24	0.76 (4)	0.9436	1.0000	0.10 (-0.05, 0.23)	1.95 (1)	0.1623	0.6490
	PA6	--	--	--	--	--	--	--	--
	PA7	7.14	2.86 (5)	0.7222	1.0000	0.01 (-0.13, 0.17)	0.01 (1)	0.9302	0.9302
	PA8	--	--	--	--	--	--	--	--
Physical medicine and rehabilitation/preventive medicine/occupational medicine (n = 250)	PA1	5.50	2.50 (4)	0.6446	1.0000	-0.03 (-0.15, 0.10)	0.16 (1)	0.6933	0.6933
	PA2	--	--	--	--	--	--	--	--
	PA3	--	--	--	--	--	--	--	--
	PA4	7.08	2.92 (5)	0.7120	1.0000	-0.13 (-0.32, 0.07)	1.70 (1)	0.1928	0.4665
	PA5	6.50	5.50 (6)	0.4819	1.0000	0.09 (-0.08, 0.29)	0.87 (1)	0.3499	0.4665
	PA6	--	--	--	--	--	--	--	--
	PA7	6.38	3.62 (5)	0.6049	1.0000	-0.09 (-0.24, 0.08)	1.20 (1)	0.2726	0.4665
	PA8	--	--	--	--	--	--	--	--
Psychiatry (n = 505)	PA1	--	--	--	--	--	--	--	--
	PA2	--	--	--	--	--	--	--	--
	PA3	--	--	--	--	--	--	--	--
	PA4	--	--	--	--	--	--	--	--
	PA5	--	--	--	--	--	--	--	--
	PA6	--	--	--	--	--	--	--	--
	PA7	--	--	--	--	--	--	--	--
	PA8	-14.25	20.25 (3)	**0.0002**	**0.0012**	-0.24 (-0.33, -0.14)	23.10 (1)	**<0.0000**	**<0.0000**
Radiology (n = 229)	PA1	--	--	--	--	--	--	--	--
	PA2	0.88	5.12 (3)	0.1629	0.9883	-0.07 (-0.18, 0.06)	1.18 (1)	0.2782	0.3709
	PA3	3.85	4.15 (4)	0.3858	1.0000	-0.09 (-0.23, 0.06)	1.58 (1)	0.2091	0.3709
	PA4	7.64	4.36 (6)	0.6276	1.0000	-0.12 (-0.32, 0.09)	1.24 (1)	0.2659	0.3709
	PA5	2.58	5.42 (4)	0.2471	0.9883	-0.04 (-0.19, 0.12)	0.27 (1)	0.6031	0.6031
	PA6	--	--	--	--	--	--	--	--
	PA7	--	--	--	--	--	--	--	--
	PA8	--	--	--	--	--	--	--	--

[a] *"--" indicates that the corresponding item was used as a final anchor item. Reference and focal group are denoted by "R" and "F", respectively. Bolded p-values are significant at $p < 0.05$.* [b] *If no DIF was detected in the LRT using an iterative, backward all-other anchor item selection approach, item PA8 was selected as an initial anchor to identify items with the lowest AIC difference in an initial LRT using a forward anchor item selection approach. Then items from the initial LRT using the forward approach with the lowest AIC difference were added as anchors and DIF was retested in studied items. Item PA8 was selected as an initial anchor as it showed minimal DIF throughout DIF testing. Items with sparse item response categories (≤5 respondents) were collapsed prior to multi-group IRT model estimation.* [c] *theta integration range for the sDRF statistic was -3.51 to 2.00.*

Supplemental Appendix 4: Detailed DIF Impact Results – Subscale-level signed DRF (sDRF) and unsigned DRF (uDRF) statistics

Table 1. Signed and unsigned subscale-level DRF statistics for EE scale [a]

		Subscale-Level sDRF			*Subscale-Level uDRF*		
DIF Grouping Variable	***Reference group; focal group*** [b]	***sDRF statistic (95% CI)***	***X^2 (df)***	***p-value***	***uDRF statistic (95% CI)***	***X^2 (df)***	***p-value***
**Sex**	Male (R); Female (F)	0.34 (0.19, 0.49)	19.32 (1)	**<0.0000**	0.34 (0.22, 0.49)	19.79 (2)	**<0.0000**
**Age Category**	≥65 years (R); <35 years (F)	-0.22 (-0.14, 0.57)	1.39 (1)	0.2380	0.25 (0.15, 0.62)	2.50 (2)	0.2862
	≥65 years (R); 35-44 years (F)	-0.16 (-0.41, 0.08)	1.71 (1)	0.1913	0.21 (0.11, 0.44)	3.83 (2)	0.1474
	≥65 years (R); 45-54 years (F)	-0.01 (-0.23, 0.21)	0.01 (1)	0.9045	0.15 (0.07, 0.36)	2.25 (2)	0.3247
	≥65 years (R); 55-64 years (F)	-0.18 (-0.27, -0.08)	13.23 (1)	**0.0003**	0.18 (0.10, 0.27)	13.52 (2)	**0.0012**
**Specialty**	GIM (R); Anesthesiology (F)	-0.04 (-0.52, 0.44)	0.03 (1)	0.8615	0.44 (0.18, 0.82)	4.17 (2)	0.1245
	GIM (R); Emergency medicine (F)	-0.42 (-0.69, -0.16)	9.51 (1)	**0.0020**	0.43 (0.23, 0.70)	10.76 (2)	**0.0046**
	GIM (R); Family medicine (F)	-0.27 (-0.64, 0.12)	1.85 (1)	0.1743	0.28 (0.12, 0.66)	2.92 (2)	0.2318
	GIM (R); General pediatrics (F)	0.63 (0.26, 1.03)	10.07 (1)	**0.0015**	0.63 (0.32, 1.04)	10.12 (2)	**0.0063**
	GIM (R); General surgery (F)	-0.10 (-0.37, 0.15)	0.55 (1)	0.4582	0.15 (0.08, 0.39)	1.99 (2)	0.3705
	GIM (R); General surgery subspecialty (F)	0.17 (-0.30, 0.60)	0.57 (1)	0.4521	0.26 (0.10, 0.69)	1.60 (2)	0.4503
	GIM (R); Internal medicine subspecialty (F)	0.21 (-0.17, 0.58)	1.32 (1)	0.2513	0.32 (0.14, 0.64)	4.28 (2)	0.1176
	GIM (R); Neurology (F)	-0.46 (-0.91, 0.01)	3.82 (1)	**0.0494**	0.60 (0.25, 1.03)	7.30 (2)	**0.0259**
	GIM (R); Obstetrics and gynecology (F)	-0.06 (-0.50, 0.40)	0.08 (1)	0.7752	0.12 (0.11, 0.55)	0.55 (2)	0.7606
	GIM (R); Ophthalmology (F)	0.18 (-0.33, 0.67)	0.51 (1)	0.4757	0.19 (0.08, 0.71)	0.67 (2)	0.7165
	GIM (R); Orthopedic surgery (F)	-0.21 (-0.64, 0.24)	0.85 (1)	0.3577	0.30 (0.11, 0.74)	2.01 (2)	0.3667
	GIM (R); Pediatric subspecialty (F)	0.21 (0.05, 0.37)	7.00 (1)	**0.0081**	0.21 (0.07, 0.37)	7.11 (2)	**0.0286**
	GIM (R); Physical medicine and rehabilitation/preventive medicine/occupational medicine (F)	0.15 (-0.01, 0.30)	3.43 (1)	0.0641	0.15 (0.05, 0.31)	3.82 (2)	0.1478
	GIM (R); Psychiatry (F)	0.12 (-0.02, 0.27)	2.75 (1)	0.0973	0.12 (0.05, 0.27)	2.89 (2)	0.2360
	GIM (R); Radiology (F)	0.29 (-0.14, 0.72)	1.68 (1)	0.1956	0.29 (0.12, 0.74)	1.80 (2)	0.4060

[a] theta integration range for the sDIF and uDIF statistics was -3.00 to 3.00; bolded p-values are significant at p <0.05. [b] reference and focal group are denoted by "R" and "F", respectively.

Table 2. Subscale-Level signed and unsigned DRF statistics for DP scale [a]

		Subscale-level signed DRF			*Subscale-Level unsigned DRF*		
DIF Grouping Variable	***Reference group; focal group*** [b]	***sDRF statistic (95% CI)***	***X^2 (df)***	***p-value***	***uDRF statistic (95% CI)***	***X^2 (df)***	***p-value***
**Sex**	Male (R); Female (F)	0.10 (-0.01, 0.21)	2.88 (1)	0.0895	0.13 (0.06, 0.24)	5.89 (2)	0.0525
**Age Category**	≥65 years (R); <35 years (F)	-0.06 (-0.18, 0.25)	0.29 (1)	0.5916	0.06 (0.04, 0.28)	0.32 (2)	0.8521
	≥65 years (R); 35-44 years (F)	-0.17 (-0.09, 0.44)	1.62 (1)	0.2033	0.17 (0.05, 0.45)	1.62 (2)	0.2033
	≥65 years (R); 45-54 years (F)	-0.12 (-0.28, 0.03)	2.33 (1)	0.1267	0.13 (0.04, 0.29)	2.81 (2)	0.2449
	≥65 years (R); 55-64 years (F)	-0.10 (-0.24, 0.04)	2.00 (1)	0.1571	0.12 (0.04, 0.25)	3.23 (2)	0.1985
**Specialty**	GIM (R); Anesthesiology (F)	0.08 (-0.25, 0.36)	0.30 (1)	0.5838	0.27 (0.08, 0.56)	3.26 (2)	0.1962
	GIM (R); Emergency medicine (F)	0.11 (-0.21, 0.40)	0.44 (1)	0.5084	0.48 (0.21, 0.75)	9.92 (2)	**0.0070**
	GIM (R); Family medicine (F)	0.40 (0.22, 0.58)	18.67 (1)	**<0.0000**	0.41 (0.25, 0.59)	19.90 (2)	**<0.0000**
	GIM (R); General pediatrics (F)	-0.35 (-0.66, -0.03)	4.54 (1)	**0.0331**	0.35 (0.14, 0.66)	4.61 (2)	0.0996
	GIM (R); General surgery (F)	-0.29 (-0.65, 0.07)	2.57 (1)	0.1089	0.29 (0.08, 0.65)	2.69 (2)	0.2600
	GIM (R); General surgery subspecialty (F)	0.07 (-0.21, 0.36)	0.22 (1)	0.6379	0.33 (0.12, 0.61)	5.33 (2)	0.0695
	GIM (R); Internal medicine subspecialty (F)	-0.01 (-0.24, 0.22)	0.01 (1)	0.9194	0.05 (0.04, 0.30)	0.38 (2)	0.8279
	GIM (R); Neurology (F)	0.41 (0.18, 0.61)	13.91 (1)	**0.0002**	0.42 (0.22, 0.62)	16.11 (2)	**0.0003**
	GIM (R); Obstetrics and gynecology (F)	-0.38 (-0.72, 0.06)	4.87 (1)	**0.0273**	0.38 (0.14, 0.72)	5.02 (2)	0.0814
	GIM (R); Ophthalmology (F)	-0.04 (-0.41, 0.30)	0.30 (1)	0.8163	0.12 (0.07, 0.54)	0.75 (2)	0.6868
	GIM (R); Orthopedic surgery (F)	0.15 (-0.20, 0.47)	0.76 (1)	0.3828	0.15 (0.06, 0.49)	0.76 (1)	0.3828
	GIM (R); Pediatric subspecialty (F)	-0.01 (-0.33, 0.31)	<0.01 (1)	0.9505	0.03 (0.05, 0.40)	0.07 (2)	0.9666
	GIM (R); Physical medicine and rehabilitation/preventive medicine/occupational medicine (F)	0.27 (-0.17, 0.72)	1.42 (1)	0.2340	0.27 (0.09, 0.74)	1.42 (1)	0.2340
	GIM (R); Psychiatry (F)	0.09 (-0.21, 0.39)	0.37 (1)	0.5408	0.29 (0.12, 0.57)	4.22 (2)	0.1210
	GIM (R); Radiology (F)	0.23 (-0.15, 0.55)	1.64 (1)	0.2009	0.23 (0.09, 0.58)	1.65 (1)	0.2009

[a] theta integration range for the subscale-level sDRF and uDRF statistics was -3.00 to 3.05; bolded p-values are significant at p <0.05. [b] reference and focal group are denoted by "R" and "F", respectively.

Table 3. Subscale-level signed and unsigned DTF statistics for PA scale [a]

		Subscale-level signed DRF			*Subscale-level unsigned DRF*		
DIF Grouping Variable	***Reference group; focal group*** [b]	***sDRF statistic (95% CI)***	***X^2 (df)***	***p-value***	***uDRF statistic (95% CI)***	***X^2 (df)***	***p-value***
**Sex**	Male (R); Female (F)	-0.12 (-0.28, 0.03)	2.55 (1)	0.1104	0.13 (0.04, 0.29)	2.95 (2)	0.2293
**Age Category**	≥65 years (R); <35 years (F)	0.21 (-0.13, 0.56)	1.40 (1)	0.2367	0.22 (0.07, 0.58)	1.67 (2)	0.4329
	35-44 years (R); ≥65 years (F)	0.06 (-0.23, 0.35)	0.18 (1)	0.6682	0.09 (0.05, 0.37)	0.70 (1)	0.7054
	≥65 years (R); 45-54 years (F)	-0.49 (-0.70, -0.29)	21.95 (1)	**<0.0000**	0.49 (0.29, 0.70)	21.95 (1)	**<0.0000**
	≥65 years (R); 55-64 years (F)	0.09 (-0.13, 0.27)	0.81 (1)	0.3693	0.14 (0.04, 0.32)	2.12 (2)	0.3466
**Specialty**	GIM (R); Anesthesiology (F)	-0.60 (-1.10, -0.05)	4.86 (1)	**0.0275**	0.60 (0.17, 1.11)	4.88 (2)	0.0873
	GIM (R); Emergency medicine (F)	-0.30 (-0.44, -0.14)	13.74 (1)	**0.0002**	0.30 (0.15, 0.44)	13.74 (1)	**0.0002**
	GIM (R); Family medicine (F)	0.10 (-0.07, 0.67)	0.29 (1)	0.5893	0.19 (0.10, 0.72)	1.16 (2)	0.5595
	GIM (R); General pediatrics (F)	-0.05 (-0.49, 0.42)	0.05 (1)	0.8249	0.12 (0.07, 0.61)	0.30 (2)	0.8604
	GIM (R); General surgery (F)	-0.05 (-0.44, 0.34)	0.07 (1)	0.7972	0.16 (0.07, 0.55)	0.77 (2)	0.6820
	GIM (R); General surgery subspecialty (F)	0.53 (0.34, 0.74)	25.28 (1)	**<0.0000**	0.53 (0.34, 0.74)	25.58 (1)	**<0.0000**
	GIM (R); Internal medicine subspecialty (F)	0.22 (-0.09, 0.51)	2.15 (1)	0.1427	0.22 (0.06, 0.51)	2.51 (1)	0.1427
	GIM (R); Neurology (F)	-0.55 (-1.05, -0.11)	5.85 (1)	**0.0156**	0.59 (0.25, 1.04)	7.53 (2)	**0.0231**
	GIM (R); Obstetrics and gynecology (F)	0.09 (-0.24, 0.43)	0.29 (1)	0.5892	0.16 (0.06, 0.51)	1.12 (2)	0.5724
	GIM (R); Ophthalmology (F)	-0.07 (-0.31, 0.19)	0.33 (1)	0.5661	0.18 (0.06, 0.42)	2.53 (2)	0.2826
	GIM (R); Orthopedic surgery (F)	0.09 (-0.35, 0.58)	0.14 (1)	0.7039	0.21 (0.10, 0.63)	1.13 (2)	0.5686
	GIM (R); Pediatric subspecialty (F)	0.18 (-0.17, 0.57)	0.85 (1)	0.3556	0.19 (0.07, 0.60)	1.19 (2)	0.5522
	GIM (R); Physical medicine and rehabilitation/preventive medicine/occupational medicine (F)						
	GIM (R); Psychiatry (F)	-0.24 (-0.33, -0.14)	23.29 (1)	**<0.0000**	0.24 (0.14, 0.33)	23.29 (1)	**<0.0000**
	GIM (R); Radiology (F)	-0.31 (-0.69, 0.08)	2.43 (1)	0.1192	0.32 (0.10, 0.72)	2.51 (2)	0.2852

[a] theta integration range for subscale-level sDRF and uDRF statistics was -3.51 to 2.00; bolded p-values are significant at p <0.05. [b] reference and focal group are denoted by "R" and "F", respectively

References

1. Shanafelt TD, West CP, Sinsky C, Trockel M, Tutty M, Satele D, Carlasare L, Dyrbye L: **Changes in Burnout and Satisfaction With Work-Life Integration in Physicians and the General US Working Population Between 2011 and 2017.** 2019.
2. Shanafelt TD, Hasan O, Dyrbye LN, Sinsky C, Satele D, Sloan J, West CP: **Changes in Burnout and Satisfaction With Work-Life Balance in Physicians and the General US Working Population Between 2011 and 2014.** *Mayo Clinic Proceedings* 2015, **90:**1600-1613.
3. Shanafelt TD, Boone S, Tan L, Dyrbye LN, Sotile W, Satele D, West CP, Sloan J, Oreskovich MR: **Burnout and satisfaction with work-life balance among US physicians relative to the general US population.** *Archives of internal medicine* 2012, **172:**1377-1385.
4. Templeton Kea: **Gender-Based Diff erences in Burnout: Issues Faced by Women Physicians.** *NAM (National Academy of Medicine) Perspective* 2019.
5. Maslach C, Jackson SE: **The measurement of experienced burnout.** *Journal of occupational behavior* 1981, **2:**99-113.
6. Maslach C, Jackson SE, Leiter MP: **Maslach Burnout Inventory Manual.** 4th edition: Mind Garden, Inc.; 2017.
7. Breslau J, Javaras KN, Blacker D, Murphy JM, Normand S-LT: **Differential Item Functioning Between Ethnic Groups in the Epidemiological Assessment of Depression.** *The Journal of nervous and mental disease* 2008, **196:**297-306.
8. HealthMeasures: **PROMIS Instrument Development and Scientific Standards Version 2.0.** 2013.
9. Mokkink LB, Terwee CB, Patrick DL, Alonso J, Stratford PW, Knol DL, Bouter LM, de Vet HC: **The COSMIN study reached international consensus on taxonomy, terminology, and definitions of measurement properties for health-related patient-reported outcomes.** *Journal of clinical epidemiology* 2010, **63:**737-745.
10. Brady KJS, Kazis LE, Sheldrick RC, Ni P, Trockel MT: **Selecting Physician Well-Being Measures to Assess Health System Performance and Screen for Distress: Conceptual and Methodological Considerations.** *Current Problems in Pediatric and Adolescent Health Care* 2019.
11. Brady KJS, Ni P, Sheldrick RC, Trockel MT, Shanafelt TD, Rowe SG, Schneider JI, Kazis LE: **Describing the emotional exhaustion, depersonalization, and low personal accomplishment symptoms associated with Maslach Burnout Inventory subscale scores in US physicians: an item response theory analysis.** *J Patient Rep Outcomes* 2020, **4:**42.
12. Teresi JA, Jones RN: **Methodological Issues in Examining Measurement Equivalence in Patient Reported Outcomes Measures: Methods Overview to the Two-Part Series,"Measurement Equivalence of the Patient Reported Outcomes Measurement Information System®(PROMIS®) Short Forms".** *Psychological test and assessment modeling* 2016, **58:**37.
13. Chalmers RP: **Model-based measures for detecting and quantifying response bias.** *Psychometrika* 2018, **83:**696-732.
14. Edelen MO, Thissen D, Teresi JA, Kleinman M, Ocepek-Welikson K: **Identification of differential item functioning using item response theory and the likelihood-based**

model comparison approach: application to the Mini-Mental State Examination. *Medical Care* 2006, **44:**S134-S142.
15. Teresi JA, Ocepek-Welikson K, Kleinman M, Cook KF, Crane PK, Gibbons LE, Morales LS, Orlando-Edelen M, Cella D: **Evaluating measurement equivalence using the item response theory log-likelihood ratio (IRTLR) method to assess differential item functioning (DIF): applications (with illustrations) to measures of physical functioning ability and general distress.** *Quality of Life Research* 2007, **16:**43-68.
16. Fieo R, Ocepek-Welikson K, Kleinman M, Eimicke JP, Crane PK, Cella D, Teresi JA: **Measurement Equivalence of the Patient Reported Outcomes Measurement Information System(®) (PROMIS(®)) Applied Cognition - General Concerns, Short Forms in Ethnically Diverse Groups.** *Psychological test and assessment modeling* 2016, **58:**255-307.
17. Chalmers R, Counsell A, Flora D: **It Might Not Make a Big DIF: Improved Differential Test Functioning Statistics That Account for Sampling Variability.** *Educational and psychological measurement* 2016, **76:**114-140.
18. **R: A language and environment for statistical computing.** [https://www.R-project.org/.]
19. Chalmers P: **mirt: A Multidimensional Item Response Theory Package for the R Environment.** *Journal of Statistical Software* 2012, **48:**1-29.
20. West CP, Dyrbye LN, Shanafelt TD: **Physician Burnout: Contributors, Consequences, and Solutions.** *J Intern Med* 2018.
21. National Academies of Medicine: **Taking Action Against Clinician Burnout: A Systems Approach to Professional Well-Being.** Washington, DC; 2019.
22. Teresi JA: **Different approaches to differential item functioning in health applications. Advantages, disadvantages and some neglected topics.** *Med Care* 2006, **44:**S152-170.
23. Kopf J, Zeileis A, Strobl C: **Anchor Selection Strategies for DIF Analysis: Review, Assessment, and New Approaches.** *Educational and Psychological Measurement* 2015, **75:**22-56.
24. Orlando Edelen MO, Thissen D, Teresi JA, Kleinman M, Ocepek-Welikson K: **Identification of differential item functioning using item response theory and the likelihood-based model comparison approach. Application to the Mini-Mental State Examination.** *Med Care* 2006, **44:**S134-142.
